# Prediction of folding patterns for intrinsic disordered protein

**DOI:** 10.1038/s41598-023-45969-5

**Published:** 2023-11-21

**Authors:** Jiaan Yang, Wen-xiang Cheng, Gang Wu, Sitong Sheng, Peng Zhang

**Affiliations:** 1 Shenzhen Institutes of Advanced Technology, Chinese Academy of Sciences, Shenzhen, 518055 Guangdong China; 2 Micro Biotech, Ltd., Shanghai, 200123 China; 3 School of Basic Medicine, Tongji Medical College, Huazhong University of Science and Technology, Wuhan, 430030 China; 4 HYK High-throughput Biotechnology Institute, Shenzhen, 518057 Guangdong China

**Keywords:** Biophysics, Computational biology and bioinformatics

## Abstract

The conformation flexibility of natural protein causes both complexity and difficulty to understand the relationship between structure and function. The prediction of intrinsically disordered protein primarily is focusing on to disclose the regions with structural flexibility involving relevant biological functions and various diseases. The order of amino acids in protein sequence determines possible conformations, folding flexibility and biological function. Although many methods provided the information of intrinsically disordered protein (IDP), but the results are mainly limited to determine the locations of regions without knowledge of possible folding conformations. Here, the developed protein folding fingerprint adopted the protein folding variation matrix (PFVM) to reveal all possible folding patterns for the intrinsically disordered protein along its sequence. The PFVM integrally exhibited the intrinsically disordered protein with disordering regions, degree of disorder as well as folding pattern. The advantage of PFVM will not only provide rich information for IDP, but also may promote the study of protein folding problem.

## Introduction

The protein intrinsic disorder plays a significant roles in both biological functions and pathological syndromes. The disordered folding conformations are particularly enhanced in proteins implicated in cell signaling, transcription, chromatin remodeling functions or binding affinity^1–4^. Also, a protein with conformation disorder may have multiple partners gaining very different folding structures before and after bound state^5^. Many studies showed that the IDP caused different human diseases, including neurodegenerative, cardiovascular, diabetes, cancer and amyloidosis etc^6,7^. For examples, α-synuclein, tau and amyloid-β proteins leads to Alzheimer disease; α-synuclein results in Parkinson disease; aggregates of PrPSC cause prion diseases; oncoproteins of p53, Mdm2, PTEN, c-Myc, AF4, BRCA1, EWS, Bcl-2, c-Fos, HPV cause cancer diseases^8–10^. In addition, many studies showed that the protein intrinsic disorder linked to functionally important phenomena, such as allosteric regulation and enzyme catalysis etc. Although, many researchers often adopted the concept that a well-defined protein 3D structure fulfilled their biological function, so far more studies revealed that the protein intrinsic disorder played important roles in biological functions^3,11,12^. Of course, the flexibility of folding conformation increases the complicity to comprehend the relationship between protein structure and biological function, and so encounters essential a challenged task to solve the protein folding object.

The scope of disordered regions in protein is indeed needed to be known firstly. In structure aspect, some proteins have fully disordered feature along almost entire sequence as IDP, and some proteins have one or more sections with intrinsic disorder along sequence as IDR. Based on relevant long (> 30 residues), about one third of eukaryotic proteins have intrinsic disorder^13^. The IDRs are often found as flexible loop connecting domains or as the regions of N-terminal and C-terminal in protein. Furthermore, the protein intrinsic disorder may be annotated by the residue according its physicochemical properties under various cell's conditions^14,15^. Regarding the folding order or disorder, the comparative analysis of composition of amino acids in sequence revealed that the residues, such as Ala, Arg, Gly, Gln, Glu, Lys, Pro and Ser, frequently occurred in intrinsic disorder regions. In contrast, the residues, such as Asn, Cys, Ile, Leu, Phe, Val, Trp and Tyr, are more common in folding ordered segments of protein. Generally, the disordered regions in protein are often found to involve polar uncharged amino acids. These polar uncharged amino acids with low hydrophobicity, which have strong electrostatic repulsions due to a higher net charge and lack of driving force for compaction, are usually considered with the IDP and IDR^16–18^. The basic knowledge greatly helped to predict the disorder regions in protein.

The protein intrinsic disorder in some degree may be detected by various experimental approaches, such as X-ray Crystallography^17^, Nuclear Magnetic Resonance (NMR)^19,20^ and Cryo-Electron Microscopy (Cryo-EM)^21^ etc. More than three thousands of unique proteins are experimentally validated to contain disordered regions in Protein Data Bank (PDB)^22^. Over two hundred millions of sequences in MobiDB database have been annotated due to disorder based on missing residues in X-ray crystallographic structures and flexible regions in NMR structures^23^. Also, many date, including over 2300 protein entries, over 4500 pieces of evidence of structural state, state transitions, interactions and functions from more than 2500 scientific publications involving involved IDP and IDR, have been annotated in DisProt database^24^. Also, the protein intrinsic disorder can be predicted by various computational tools, and some of databases have been established^25^. The prediction approaches are classified into four categories. First, the prediction based on physical or chemical properties of residues or prior knowledge of segments of intrinsic protein disorder, such as FoldIndex^26^, NORSp^27^, GlobPlot^28^, CH plot^29^, and DisPredict^30^, PONDR^31^ and SLIDER^32^. Second, the prediction based on inter-residue contact, such as IUPred^33^, FoldUnfold^34^, and Ucon^35^. Third, the prediction based on algorithms trained on structural data sets to determine disorder and order regions in amino acid sequence, such as PrDOS^36^, MobiDB^37^, IDEAL^38^, PONDR^39^, Spritz^40^, DisEMBL^41^, RONN^42^, s2D^43^, MFDp^44^, Disopred3^45^, and D^2^P^2^^46^. Forth, the prediction based on a deep-learning process. Recently the developed Alphafold approach, which achieved protein structure prediction accuracy competitive with that of experimental determination, has tried to define the lower confident regions in protein with lower pLDDT (predicted Local Distance Difference Test) score as IDR^47–51^. Also, some approaches, such as IUPred2A and DEPICTER2 etc., tried to predict the relationship between IDR and disorder biological functions^52–54^. Anyway, for more than two decades, the effort, which focused on developing qualitative prediction for IDP and IDR, has made much progress to expose protein intrinsically disorder.

Despite the progress of intrinsically disorder protein including experiment measurement, prediction approach, database and application, the challenges persist in two aspects. First, although most of approaches for IDP predicted the disordered regions for location in protein, the knowledge of possible changing folding conformations in disorder region are ambiguity. Second, let us assume that the changing folding conformations are known, it is difficult to present or describe various folding patterns for IDP or IDR. With perspective of protein folding problem, in 1969, Cyrus Levinthal already indicated that a protein may have an astronomical number of folding conformations along its amino acid sequence^55^. In practice, these possible conformations with stable status inside physiological cells or under different environment are interested to be known. In order to overcome these hurdles, we developed a novel approach, protein structure fingerprint, completely to reveal the protein folding variations from amino acid sequence^56,57^. A backbone of five consecutive amino acid residues is identified as a universal folden, all possible folding shapes are obtained and then expressed by protein folding shape code (PFSC) which is alphabetic letter description. For any protein, its Protein Folding Variation Matrix (PFVM) assembles all possible local folding variations along amino acid sequence, and it possesses three prominent features. First, the PFVM showed the fluctuation variations with folding patterns and the number of folding shapes along sequence which revealed the protein intrinsic disorder. Second, with digital alphabetic description, the folding fluctuation along sequence is displayed in a matrix, so the intrinsic disorder can be directly observed at a glance and analyzed in detail within PFVM. Third, the most possible folding conformations for any protein can be acquired with using the PFSC alphabetic letters on top rows in PFVM, even an astronomical number of folding conformations can be predicted with using all PFSC letters in PFVM. Therefore, the protein structure fingerprint approach provides a significant means to predict the protein intrinsic disorder, which well presents both disordered regions and folding patterns.

## Materials and methods

The protein structure fingerprint approach firstly defines the protein folding shape code (PFSC) for the backbone of five consecutive amino acid residues, and its folding shapes are described by a set of digital alphabetic letters. Subsequently, a database is created to collect all possible folding shapes in PFSC for any combination of five amino acids from 20 amino acids. Finally, the protein folding variation matrix (PFVM) is obtained according protein sequence with extracting folding shapes from the database. The protein structure fingerprint approach well predicts the intrinsic disorder for a protein, and also explicitly displays possible folding patterns along amino acid sequence in PFVM. Our developed protein structure fingerprint approach has been published in two papers with detail description^56,57^. Here, the approach is briefly summed below description.

### Protein folding shape code (PFSC)

**Figure 1:**
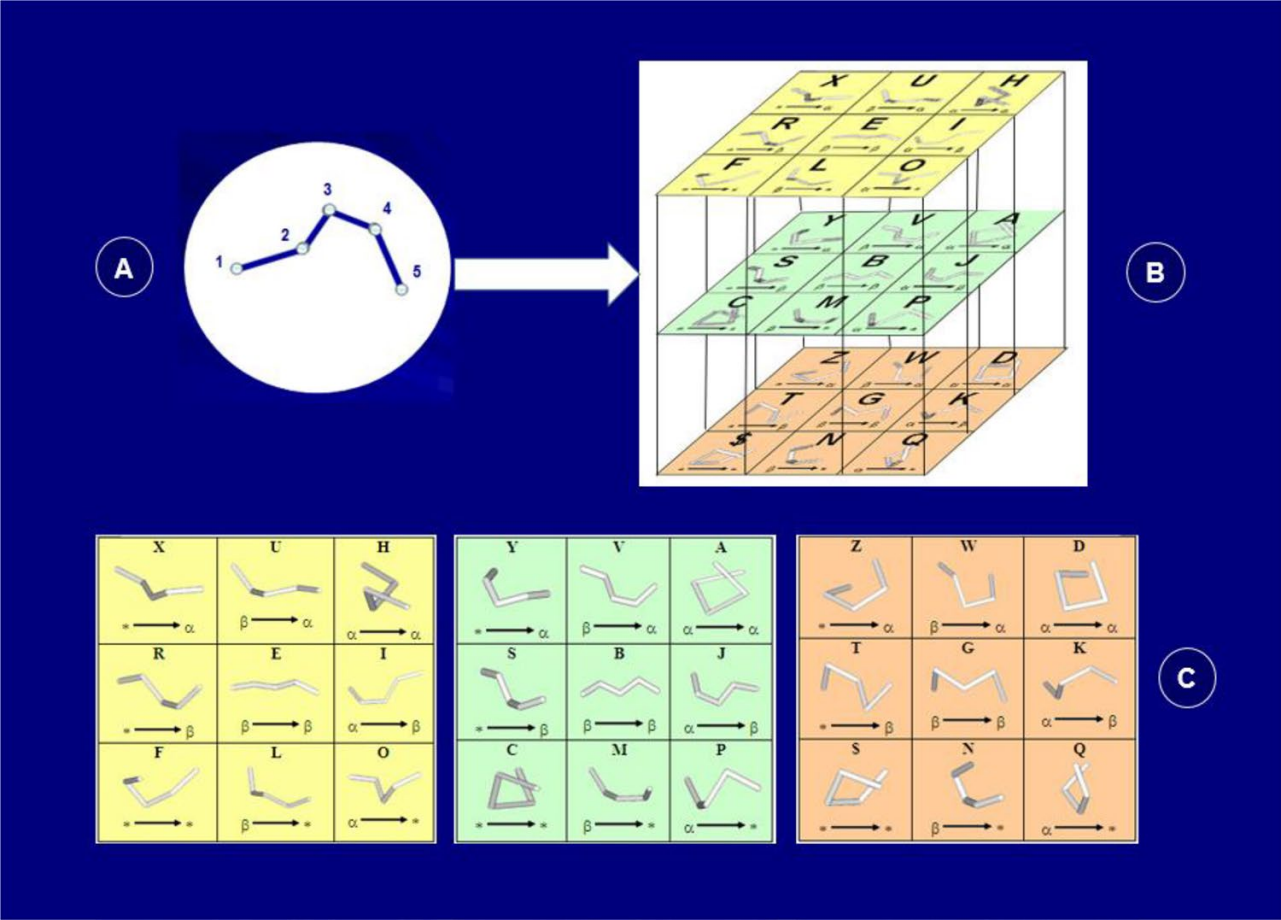
Protein folding shape code (PFSC)^56^. With mathematical derivation from five points connection as ball-and-stick model (**A**), a set of 27 PFSC is obtained covering complete folding space of five amino acids, and described by 26 alphabetic letters and $ symbol. The cubic on top represents the PFSC relationship which each PFSC shares partial folding shape with neighbor one (**B**). Three squares on bottom clearly display both folding shape and alphabetic letter, and each folding shape is a vector (**C**).

In protein structure fingerprint, a folden of five amino acid residues was taken as folding element, and the description of its complete folding space was acquired mathematically. The backbone of five consecutive amino acid residues has two continuous dihedral angles (1-4 and 2-5), which is the smallest element to reveal how a twist is continued or reversed in some degree. First, a folden with five points connection as ball-and-stick (Fig. 1A) was mathematically derived to expose its possible folds. Without biological structure constrain, the folds are freely rotated around each join point with topological uniformity, and all possible folds in geometric space form a complete and continuous aggregation. Second, with matrix transformation, the mathematical equation with initial fifteen dimensional variables for folden of five points was derived to reduce the dimensions into three effective variables. Third, the continuous aggregation of folding geometric space was converted into partitioned quantum description, and then applied to biological protein. Then a set of 27 folding shapes (Fig. 1B and C) is obtained which is able completely to cover various folding patterns for five successive amino acid residues. Fourth, with digital description, these 27 folding shapes are represented by 26 alphabetic letters and “$” symbol. As a set of 27 alphabetic letters is applied to protein systems, it is called as the Protein Folding Shape Code (PFSC)^56^, which together represents the full space of folding shapes of five amino acid residues. Each PFSC letter represents a specific folding pattern. For examples, “A” is for typical alpha-helix; “V, J, Y, P, D and H” contain partial feature of alpha-helix; “B” for typical beta-strand; “E, G, S, M, V and J” contain partial feature of beta-strand and “X, U, R, I, F, L, O, C, Z, W, T, K, $, N and Q” for irregular tertiary folding shape. With alphabetic digital letters, one PFSC code represents a folding pattern of five amino acid residues, and reversely the folding shape of a given 3D coordinate of five amino acid residues can be represented by one PFSC letter. Furthermore, a PFSC string can be converted into its protein 3D conformation in space, and reversely the folding conformation of a given protein 3D structure can be described by a PFSC string. Thus, any protein 3D structure in PDB is able to have a PFSC string description. Therefore, the PFSC is an alphabetic digital presentation for protein conformation, which provides complete description for protein fold without any gap from N-terminal to C-terminal, covering over regular secondary structure fragment as well as tertiary structure fragment.

### PFSC database

**Figure 2:**
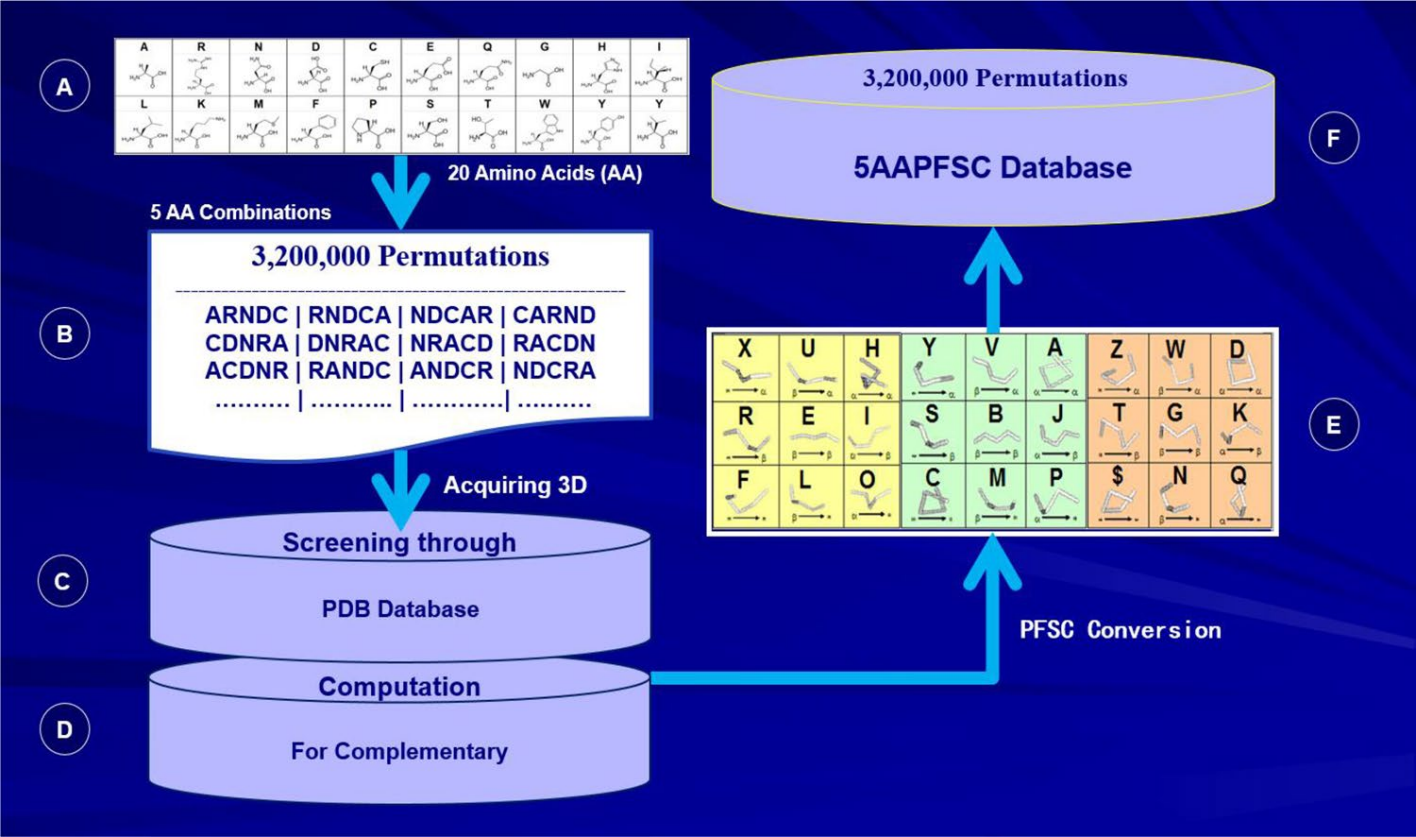
The procedure to construct 5AAPFSC database^57^. From 20 amino acids (**A**), 3,200,000 sets of permutations of five amino acids are generated (**B**). The possible folding shapes for each set of five amino acids can be found from PDB (**C**) and computational simulation calculation (**D**). All 3D folding shapes for each set of five amino acids are converted into PFSC alphabetic letters (**E**) and then stored into 5AAPFSC database (**F**).

A database of folding patterns for any five amino acid residues has been generated, which stored all possible folding shapes with PFSC alphabetic letters for five amino acid residues. With molecular constrain, each set of five amino acid residues may have different type and different number of folding patterns. However, under PFSC protocol, the number of folding patterns is not beyond 27. Thus, a database, which has any combination of five amino acids and contains all possible folding patters for each five amino acids, is needed. First, all potential combinations of any five amino acids should be known. The total numbers of possible permutations for five amino acids from 20 amino acids is 3,200,000 (Fig. 2A and B). The 3D coordinates of about 2,000,000 of permutations of five amino acids are available in protein database (PDB), and these data have been firstly collected through screening PDB (Fig. 2C). For remaining approximately 1,200,000 of permutations of five amino acids, their possible 3D coordinates for folding structures are calculated by molecular dynamics simulations with CHARMM (Chemistry at Harvard Macromolecular Mechanics) force fields (Fig. 2D)^56^. Consequently, the 3D coordinates of all possible folding shapes for five amino acid residues were converted into PFSC code (Fig. 2E). Then, a database is created to store all possible folding shapes in alphabetic letters of PFSC for 3,200,000 sets of five amino acid residues, which is named as 5AAPFSC (Fig. 2F). Hence, the 5AAPFSC database contains all probable combinations of five amino acids, and the folding shapes for each set of five amino acids are stored by PFSC alphabetic letters, including both folding patterns and number of folding variations. Therefore, the 5AAPFSC database has complete folding information for five amino acids^58^.

### Protein folding variation matrix (PFVM)

**Figure 3:**
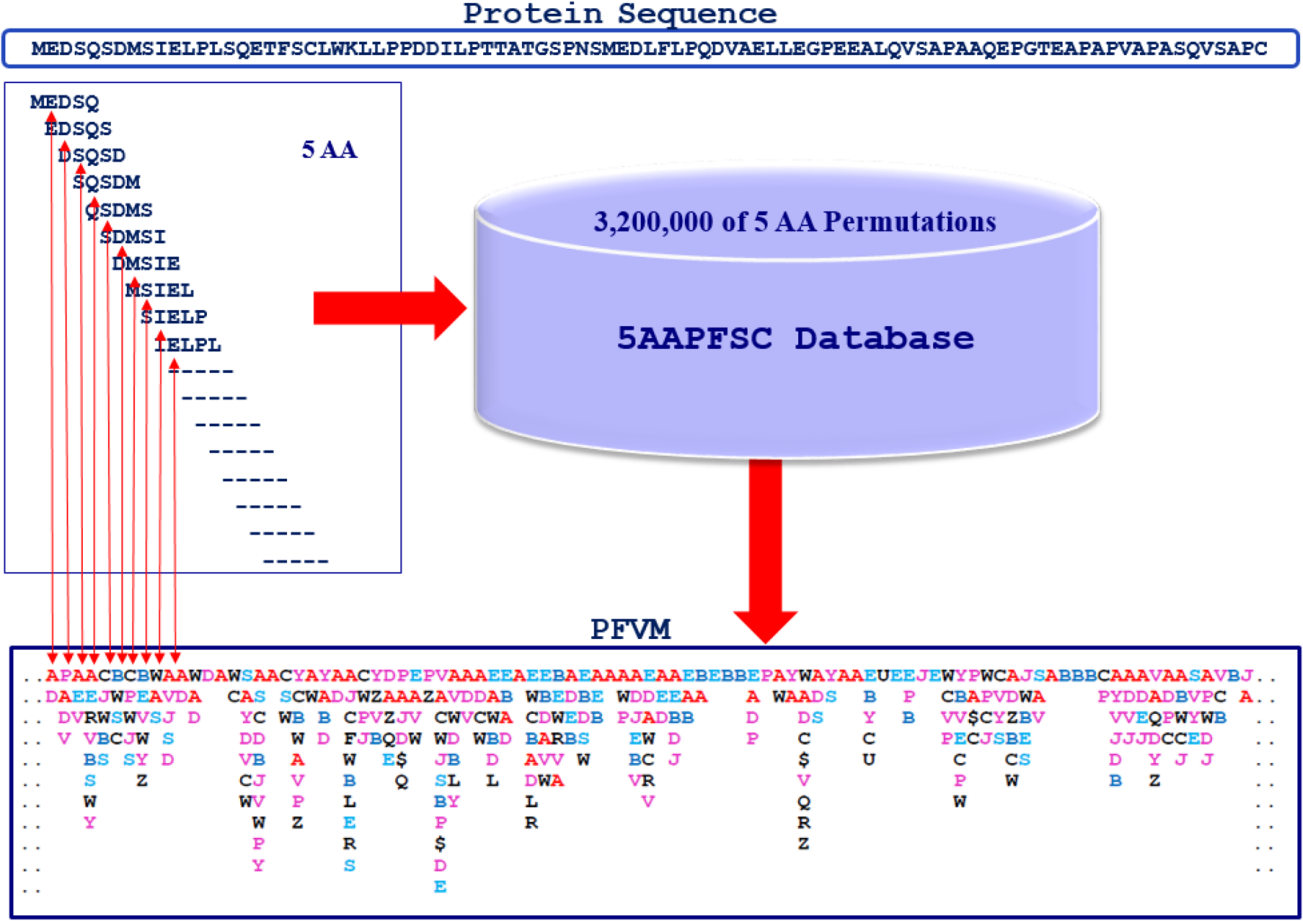
Protein folding variation matrix (PFVM)^57^. With each set of five amino acids from N-terminal to C-terminal along protein sequence, the PFSC alphabetic letters for folding shapes are obtained from 5AAPFSC database, the PFSC alphabetic letters are listed in each column aligning at middle of five amino acids, and then the PFVM is generated.

With protein folding fingerprint, the local folding variations along a protein sequence are assembled into a protein folding variation matrix (PFVM)^57^. The local folding variations for each five successive amino acids along a protein sequence, which are stored by a set of PFSC letters in 5AAPFSC database, are extracted and displayed in a vertical column aligning middle of five amino acids. Starting from the first set of five amino acid residues from N-terminal, each step moves forward by one residue with sharing four amino acids from prior step until C-terminal, and then the PFVM is formed. For example, in Fig. 3, the first set of five amino acids “MEDSQ” has “AD” as PFSC in first column; the second set of five amino acids “EDSQS” has “PADV” as PFSC in second column; the third set of five amino acids “DSQSD” has “AEV” as PFSC in third column; the next set of five amino acids “SQSDM” has “AERVBSWY” as PFSC in corresponding column and so on until the end of C-terminal. Obviously, four features of protein folding variations are exposed by PFVM. First, the PFVM showed that most of five amino acids actually had more than one PFSC letter, but the maximum number of PFSC letters would not be excess more than 27. Second, different set of five amino acids has different folding patterns, which is listed on each column in PFVM. Third, the PFSC letters in each column are ranked, i.e. the most possible folding shapes are on the top in PFVM. The folding shapes of five amino acids, which have higher frequency in PDB database or lower free energy in computation simulation, are considered with most probability in folding patterns and are assembled at the top rank. Fourth, with PFVM, an astronomical number of folding conformations for a protein may be constructed by taking one PFSC letter from each column to form a massive number of PFSC strings. Also, the most possible folding conformations can be constructed by the PFSC letter on top of each columns in PFVM. The procedure, how the local folding variations are assigned from a protein sequence to its PFVM, is illustrated in Fig. 3. Therefore, the PFVM is generated, and the local folding variations for entire protein are revealed according the PFSC letters in each column along sequence. Therefore, the PFVM provides a significant tool to study the protein folding problem as well as the protein intrinsic disorder.

## Results

The features of protein intrinsic disorder are well displayed in PFVM based on folding variations of five amino acid residues, and the folding variations can be observed by extension of longer length of fragment or entire protein. Also, the PFVM has ability to provide rich disorder information for any protein, including to expose both flexible regions and folding patterns. Furthermore, the results of protein intrinsic disorder in PFVM may be compared with various given experiment data and prediction approaches, and the similarity and dissimilarity between PFVM and other methods are discussed. Several proteins with typical intrinsic disorder are presented here, including SUMO1_HUMAN protein as a well-known IPD, Aβ42 protein involving Alzheimer's disease, PAGE4_HUMAN protein relating to biological function and pathogenic molecular mechanism and etc.

### SUMO1_HUMAN

**Figure 4:**
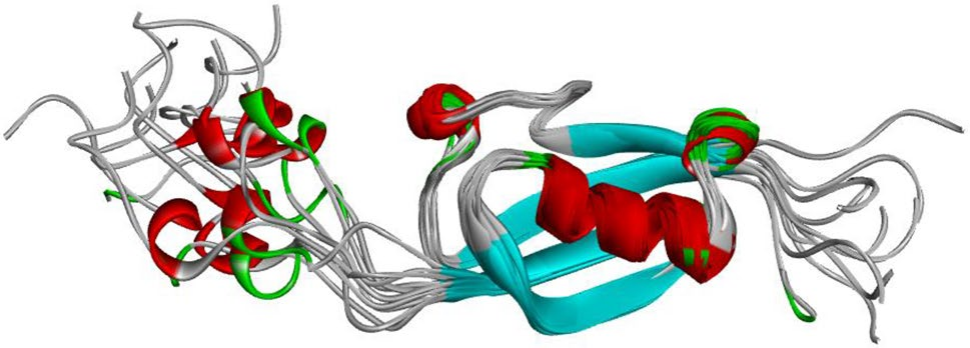
The superimposition of 10 conformations of SUMO1_HUMAN from PDB (PDBID = 1A5R)^59^. The folding types of segments are colored, such as red for alpha helix, blue for beta strand and grey for loop.

SUMO1_HUMAN is a well-known protein with conformational flexibility, the region in middle part is relatively stable in fold, conversely the N- terminal and C-terminal are intrinsic disorder regions^59^. The characteristics of intrinsic disorder are visually displayed by the superposition of 17 conformations of 7 structural data from PDB in Fig. 4.

**Figure 5:**
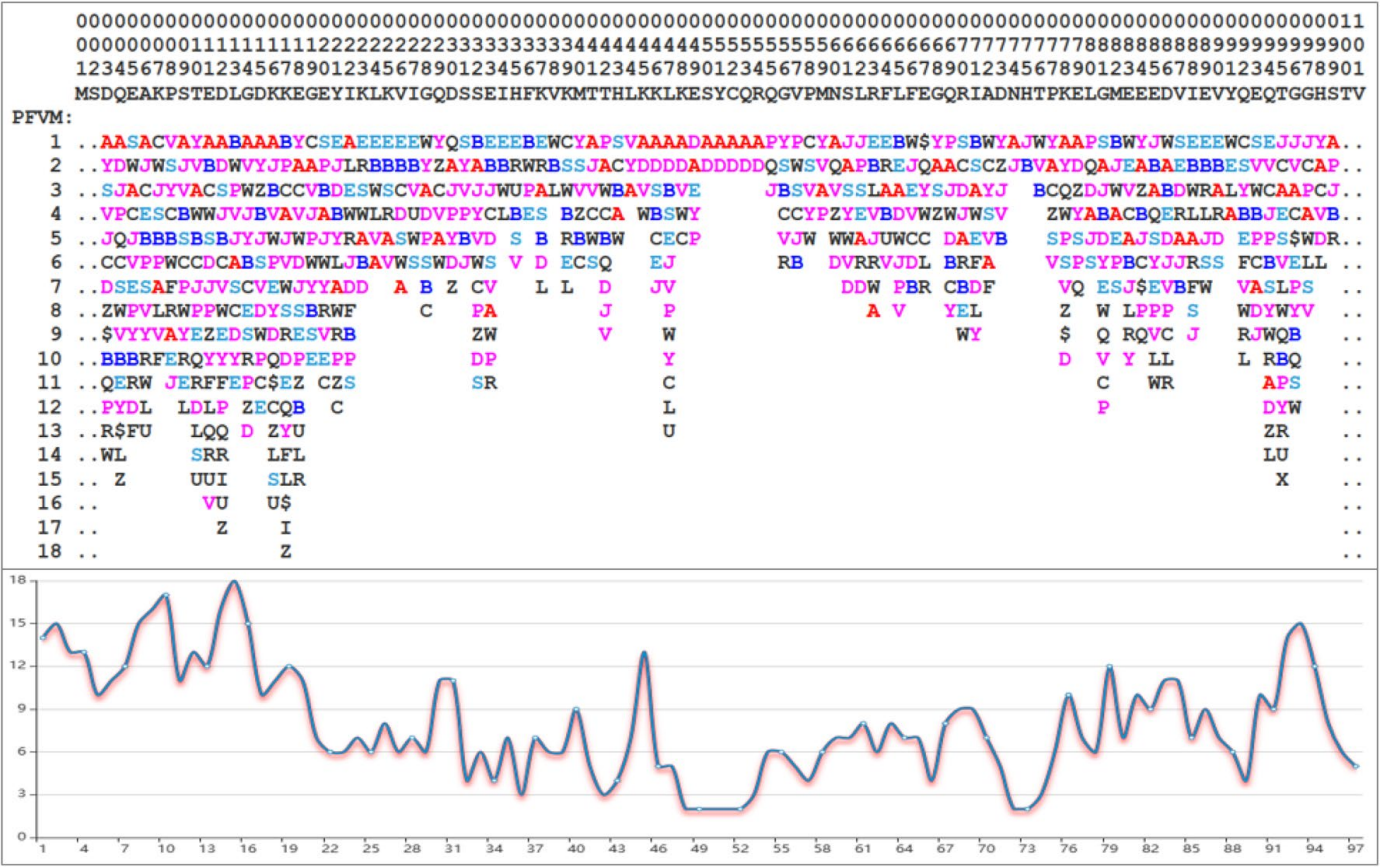
Protein folding variation matrix (PFVM) of SUMO1_HUMAN^59^. The top section is the residue position number, the amino acid sequence and PFVM. In PFVM, the PFSC alphabetic letters on each column represent the folding shapes of each set of five amino acids, and the folding variations for entire protein are completed along the sequence from N-terminal to C-terminal. The PFSC letters are marked by colors: red is for typical helix; blue for typical beta strand; pink and light blue for folds with partial helix or beta strand; black for irregular folds. The bottom section is a curve which displayed the variations of numbers of folding patterns which is every number of each five columns in PFVM.

**Figure 6:**
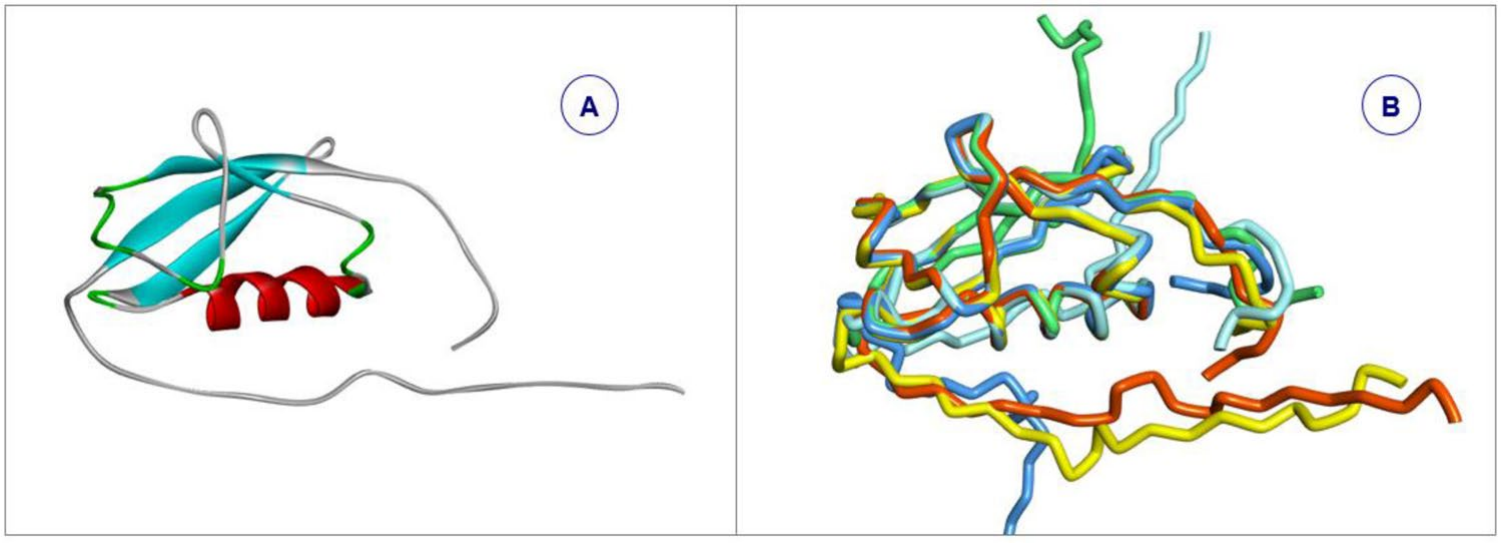
The 3D conformations constructed from SUMO1_HUMAN PFVM. The 3D conformation image of PFSC string on top of SUMO1_HUMAN PFVM of is shown on (**A**). The superposition of five of 3D conformations is shown on (**B**) which each PFSC string is obtained by replacement of PFSC letters from same column in PFVM.

According the sequence of SUMO1_HUMAN, with extracting PFSC letters for each five amino acids from 5AAPFSC database, the PFVM is obtained which revealed the folding variations along sequence (Fig. 5 top)^59^. The features of intrinsic disorder for entire SUMO1_HUMAN protein may be immediately glanced up in PFVM while the local folding patterns are exposed by PFSC letters in each column for corresponding set of five amino acids along sequence. First, the degree of disorder can be assessed by fragments. For examples, the section with the most disorder is shown in the region of 1-22 in sequence; the disorder is reduced in the region of 23-49; the section with folding ordered in the region of 50-55; the intrinsic disorder is increased in the regions of 56-73 and 76-101. Furthermore, the trend of degree of disorder along the sequence can be displayed by the curve in Fig. 5 bottom, which shows the fluctuation of average number of five columns of PFSC letters in PFVM. Second, the PFSC letters on top rows may expose the possible secondary structure fragment. The PFSC letters with red or pink color is for alpha helix, and the PFSC letters with blue or light blue for beta strand. With PFSC letters on top two rows, it is apparently that the region 44-54 trends for alpha helix and the region 23-27 and 32-37 for beta strands. Third, the possible various folding conformations for entire protein may be constructed from PFVM. For example, the PFSC string at top row in PFVM, as PFVM-01, is one of the most possible conformation for SUMO1_HUMAN and its 3D image is displayed in Fig. 6A. Sequentially, any PFSC letter in PFVM-01 can be replaced by other PFSC letters in same column, and multiple PFSC strings are formed which represent different conformations. The superposition of five conformations of SUMO1_HUMAN constructed from PFVM is displayed in Fig. 6B. These characteristics from PFVM provide rich information for folding variations and construction of conformations, which will promote better understanding the intrinsic disorder in SUMO1_HUMAN.

**Figure 7:**
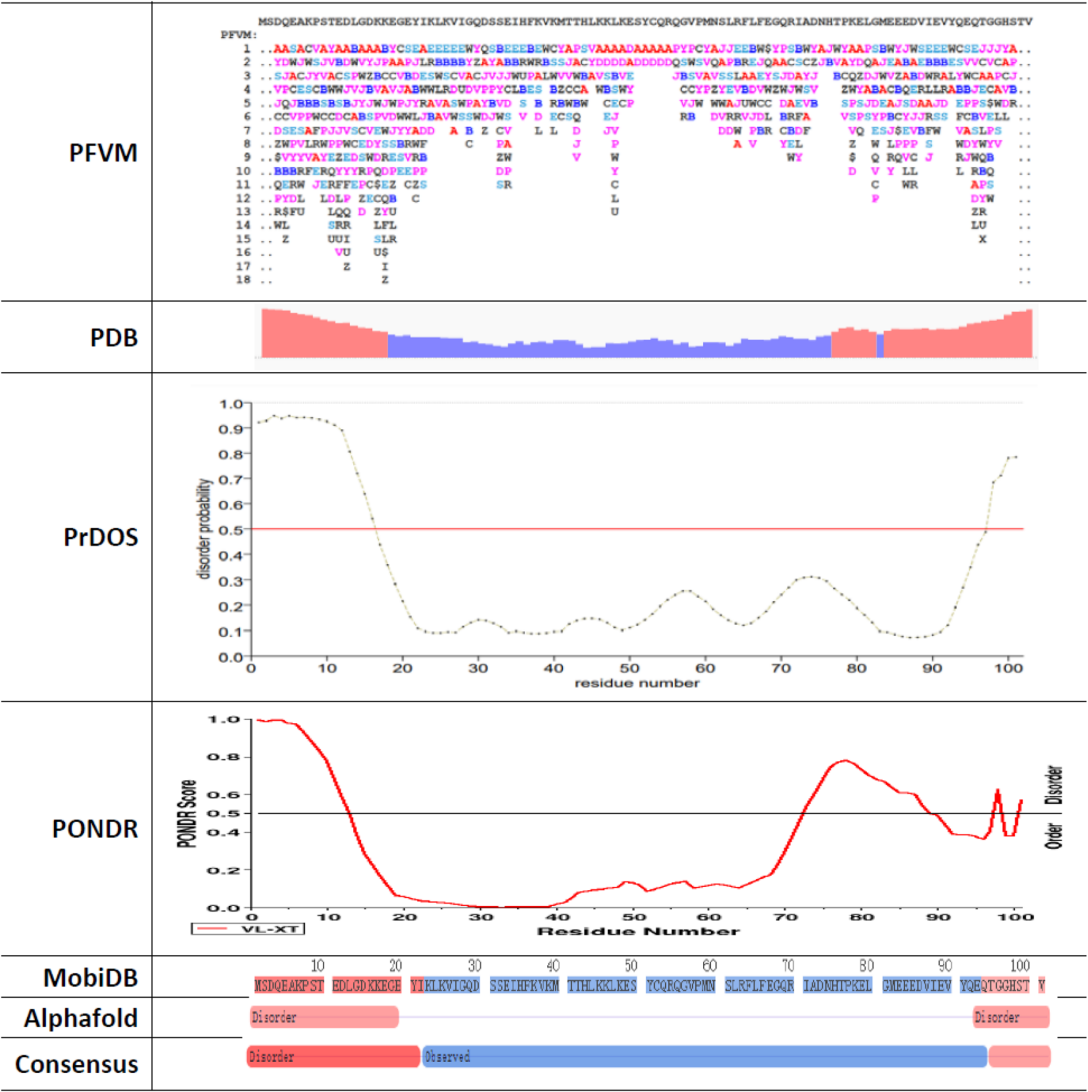
Comparison of various prediction approaches for intrinsic disorder of SUMO1_HUMAN. The folding variations by PFVM is displayed on top, then PDB, PrDOS, PONDR, MobiDB, AlphaFold and other approaches are displayed below separately.

The PFVM result of SUMO1_HUMAN can be compare with other protein intrinsic disorder approaches. The comparisons with PDB, PRDOS, PONDR, MobiDB, AlphaFold and other consensus are displayed in Fig. 7. Generally, the trends of fluctuations of protein intrinsic disorder for SUMO1_HUMAN in all approaches have overall agreement, i.e. the N-terminate and C-terminate are intrinsic disorder regions and the middle portion is relatively in order for conformation. The MobiDB, AlphaFold and other approaches only defined the disordered regions; the PDB, PRDOS, PONDR offered the degree difference of disorder in different regions. However, the PFVM provides the local folding variations in PFSC code in each column to illustrate the protein intrinsic disorder along sequence, which explicitly reveals the disorder region, degree of disorder and possible folding patterns. In addition to predict the disordered regions, the PFVM clearly provided the possible folding patterns in PFSC, and can further construct various possible 3D conformations for entire protein.

### Aβ42

**Figure 8:**
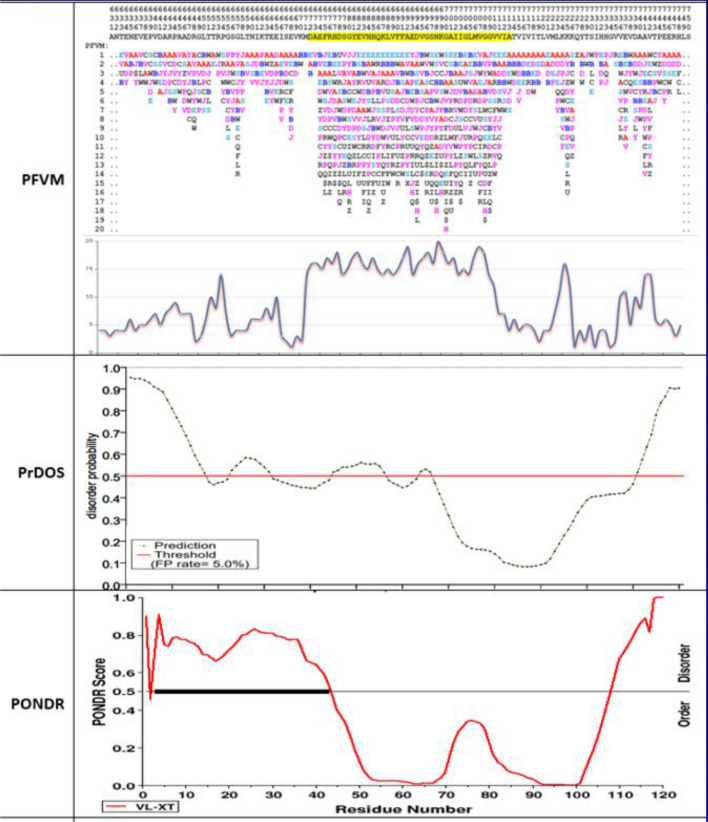
The prediction of intrinsic disorder for region of A4_HUMAN (631-750). The folding variations by PFVM is displayed on top; the PrDOS perdition on middle section and the PONDR prediction on bottom. The region of amyloid beta 42 (Aβ42) is marked by yellow color in sequence.

Amyloid beta 1-42 (Aβ42) is a peptide with 42 amino acid residues that are derived from amyloid-beta precursor protein (APP), which is cleaved by secretase to yield Aβ42 in a cholesterol-dependent process and substrate presentation^60^. The Aβ42 molecule can aggregate to form flexible soluble oligomers, which may trigger misfolding conformations and lead Alzheimer’s disease^61^. In aspect of genomic structure, the Aβ42 is a motif of A4_HUMAN protein of APP, it locates at region of 672-713 in sequence. The PFVM for fragment of 631-750, which contains the region of Aβ42, is displayed in Fig. 8. It is apparent that the region of 672-713 of Aβ42 with yellow color mark has higher folding variations in PFVM. Thus, the PFVM explicitly exposed the Aβ42 with higher intrinsic folding order. Furthermore, the PFVM is able to expose the folding feature of Aβ42. The PFSC string on top row of PFVM for Aβ42 has 28 letters as “B”, “E” or “S” (with blue and light blue color) among 42 PFSC letters, which shows two-third of Aβ42 originally trending beta strand folding shape. Also, the PFSC strings on top two rows in PFVM have most beta folding shape with blue and light blue color. However, the PFSC strings on third row and below else increase alpha-helix PFSC letters “A”, “V”, “J” and “D” etc. (with red and pink color). Thus, under certain situation, the folding conformation of Aβ42 is able to change into the alpha-helix fold from the beta strand fold, which misfold causes Alzheimer's disease. Therefore, the PFVM does not only reveal the protein intrinsic disorder, but also may assist to understand how the intrinsic disorder involve the disease with conformation change. In addition, the PFVM has dissimilar result of intrinsic disorder with comparison of results from different prediction approaches. For example, both PrDOS and PONDR approaches predicted that the region of 672-713 had relative stable folding changes while both N-terminal and C-terminal had higher disorder (Fig. 8). Conversely, the PFVM indicated that the region of 672-713 had higher intrinsic disorder and both N-terminate and C-terminate were relatively stable. With further observation, for region of 672-713, the disagreement of disorder for Aβ42 between PrDOS and PONDR approaches are found, i.e. for region of 672-695, the PrDOS is relative disorder, but the PONDR is stable order. With local folding variations of five amino acids, the PFVM provided more reliable prediction for intrinsic disorder. The PFVM does not only provided the illustration for possible changes of folding patterns, but also offered more sensible intrinsic disorder information for Alzheimer's disease. The Aβ42 is one of neurodegenerative diseases which related IDP. The neurodegenerative diseases involved with intrinsic disorder in many proteins. The PFVM of 20 proteins related neurodegenerative diseases are displayed in supplementary document.

### PAGE4_HUMAN

**Figure 9:**
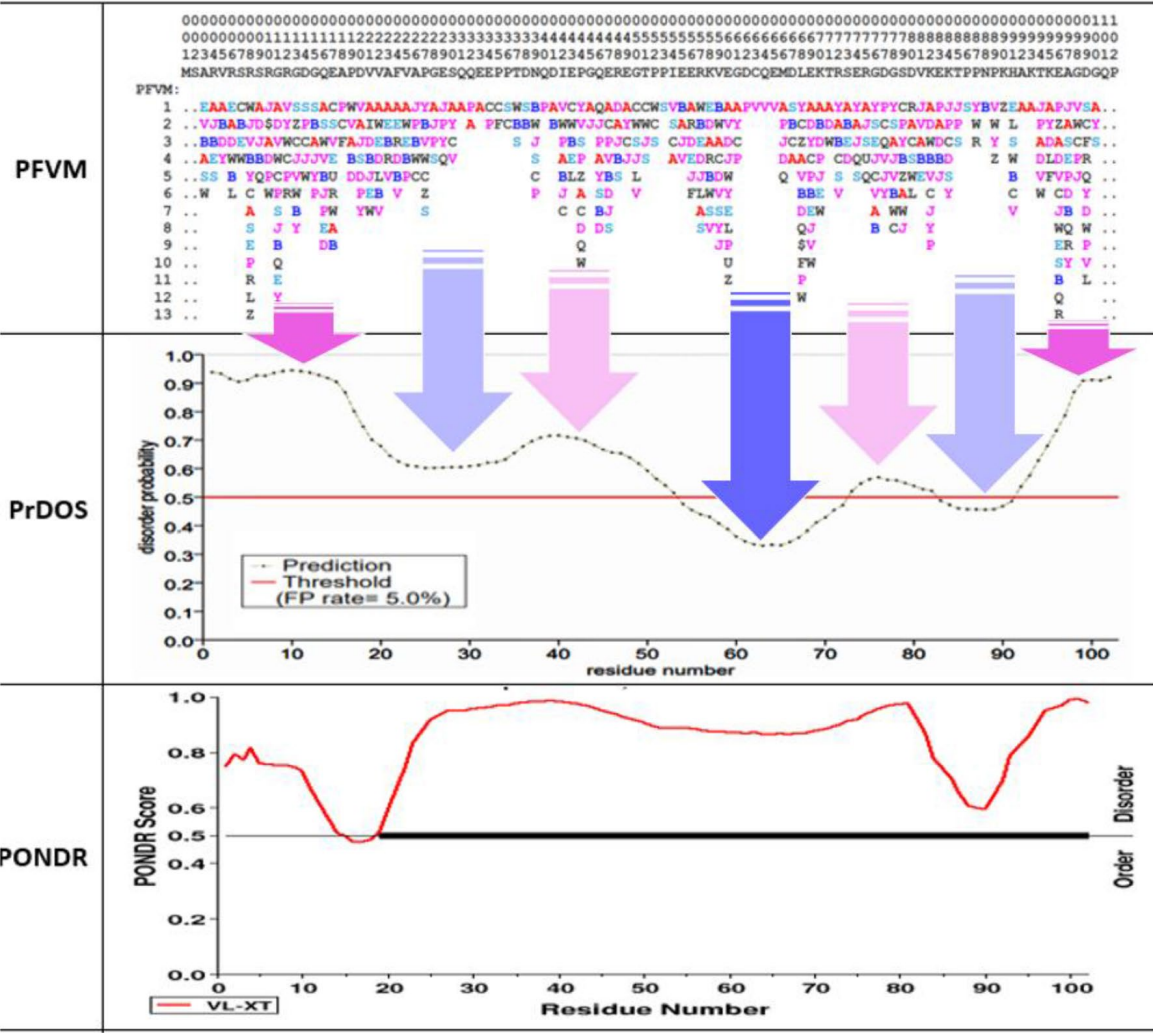
The prediction of intrinsic disorder for Prostate-Associated Gene 4 (PAGE4) protein. In PFVM, the amino acid sequence and the position number are listed on top, and the folding variations with PFSC letters in colors for each of five amino acids are displayed in each column. In PrDOS or PONDR, it have 0.5 threshold remarking intrinsic disorder. The arrows show the overlap agreement of disorder regions between PFVM and PrDOS. The pink arrow is for the region with highest disorder and light pink for higher disorder. The blue arrow is for the region with relative stable order and light blue for reduced stable order.

Prostate-Associated Gene 4 (PAGE4) is an IDP that lacks a unique 3D conformation, comprises a large fraction of the human proteome and plays important roles in numerous cellular functions^62^. Its sequence has the amino acids enriched in polar and charged (~ 53%) and < 10% of hydrophobic residues such as Leu (L), Met (M), Phe (F), Trp (W) and Tyr (Y), which cause PAGE4 intrinsic disorder. Much progress has been made in understanding PAGE4 structure and biological function. The PAGE4 is a remarkably prostate-specific tumor antigen that is upregulated in prostate cancer (PCa), and it is a stress-response protein that is upregulated in response to a variety of inflammatory stress^63,64^. As such, PAGE4 is a novel therapeutic target for treating and managing PCa, and so the revelation of its intrinsic disorder is important. According the protein sequence of PAGE4_HUMAN with 102 amino acids, its PFVM is obtained and displayed on top of Fig. 9. The local folding variations for PAGE4_HUMAN, based on five amino acids along sequence from N-terminal to C-terminal, is explicitly indicated in PFVM. The Protein DisOrder prediction System (PrDOS) approach predicted the disordered regions for PAGE4 with using amino acid sequence disorder probability, and its disorder is shown by a curve on middle section of Fig. 9. The PrDOS assessed the degree of disordered regions for PAGE4 according the scores larger than threshold (0.5). Overall, the predicted disorder trend between PFVM and PrDOS is in concert and harmony, and the arrows in Fig. 9 showed the comparison of disorder regions of PAGE4 between PFVM and PrDOS. The arrows with pink color for higher disorder; the light pink for weak disorder; the blue for higher order region and light blue for weak order. Thus, the results of PFVM and PrDOS provided overall agreement of prediction of disorder regions for PAGE4. The result of Predictor of Natural Disordered Regions (PONDR) approach for PAGE4 is shown on bottom section of Fig. 9. The PONDR® VL-XT algorithm merged three predictions together, including variously long disordered regions, trained on X-ray characterized and terminal disordered regions. The PONDR predicted that PAGE4 was complete disorder due to the score for almost regions are above threshold (0.5). However, to compare with region against region, the trend of PONDR is different from either DrDOS or PFVM. So, it should be further studied what causes the difference. Nevertheless, the PFVM does not only predict the disorder regions and the degree of disorder, but also provides the possible folding patterns with PFSC letters in each column. With PFVM, more intrinsic disorder features can be observed. First, the number of PFSC letters on each column showed the disorder degree for five amino acid residues of PAGE4, and the combination of more columns together for wider region can well display the disorder trend. Second, the feature of secondary structure of PAGE4 is exposed by PFVM. For example, the top row of PFSC string in PFVM indicated many alpha-helical fragments (red color “A” and pink color letters) with some beta-strand fragments (blue color “B” and light blue letters), and showed how the secondary structure is distributed along sequence. Third, each letter in PFSC string on top row can be replaced by other PFSC letters in same column which will produce various folding conformations for entire PAGE4. Therefore, the PFVM may provide a theoretical basis for the in-depth study of biological function and pathogenic molecular mechanism of PAGE4.

## Discussion

### PFVM advantage

The PFVM does not only indicate the locations of disorder regions in protein, but also reveals what possible folding patterns will be occurred. Any native protein with biological activity has folding flexibility in some fragments with different degrees. The region of disorder is able to be predicted by various methods and the degree of disorder may be specified by some methods, but the possible folding patterns almost have not been explicitly defined or annotated yet. The X-ray Crystallography or Cryo-EM can determine the disordered regions based on unassigned segments with indeterminacy of atom position, but cannot provide specific folding information. So far, most of computational approaches and database for IDP only predict the disordered regions, but do not provide folding patterns for the region. Even recently developed AlphaFold approach only can predict the disordered regions depending on lower confident regions in protein with lower local distance difference test score, but cannot define what possible folding pattern will be. The revelation of possible folding patterns is a challenged task for IDP study and protein folding object. In addition, how to unambiguously present local changeable folding patterns and multiple protein conformations with visualization is another obstacle. The PFVM provides an integrated means to expose the disorder region, degree of disorder and folding patters for protein intrinsic disorder. The PFSC letters in each column of PFVM are wisely utilized to display the change of local folding patterns, and then possible conformations for entire protein can be constructed by these PFSC letters. Also, the PFVM may provide integrated information to understand how the intrinsic disorder impact the biological function in structure.

The PFVM provided a universal approach to examine the protein folding. The proteins may be classified into either intrinsic disorder protein or ordered protein, and some of IDP approaches adopted a threshold value to determine protein disorder region. Based on five amino acid residues, the PFVM provided very detail information of local folding variations along entire sequence. Therefore, the PFVM avoided to take threshold score to adjust disorder region, and evaded to classify proteins into two categories as either order or disorder, which well reflexes the nature of protein conformation, including the region, degree of disorder and possible folding patterns.

### Disorder and protein folding

The PFVM provided a novel approach integrally to study both protein intrinsic disorder and protein folding problem. The IDP is focusing on the intrinsic disorder and related biological functions and pathological syndromes, which is part goal of protein folding problem. The protein folding problem is focusing on to predict protein structures with possible multiple conformations, which contains the folding variations for intrinsic disorder protein. To acquire comprehensive conformations for any protein is an extreme difficult task, and many methods have developed for protein structure prediction^65^. Based on artificial neural networks with deep learning, recently developed AlphaFold is able to predict accurate protein structure and may define the region for intrinsic disorder, but it cannot clearly provide possible folding patterns for IDR. However, the PFVM provided the folding variations along sequence, which can predict an astronomical number of folding conformations for a protein while it can explicitly reveal the intrinsic disorder for a protein. With digital alphabetic code, rich folding information is concentrated within a PFVM matrix, which is a good starting point for the IDP research and the complete prediction for protein folding.

### Folding change and space orientation

**Figure 10:**
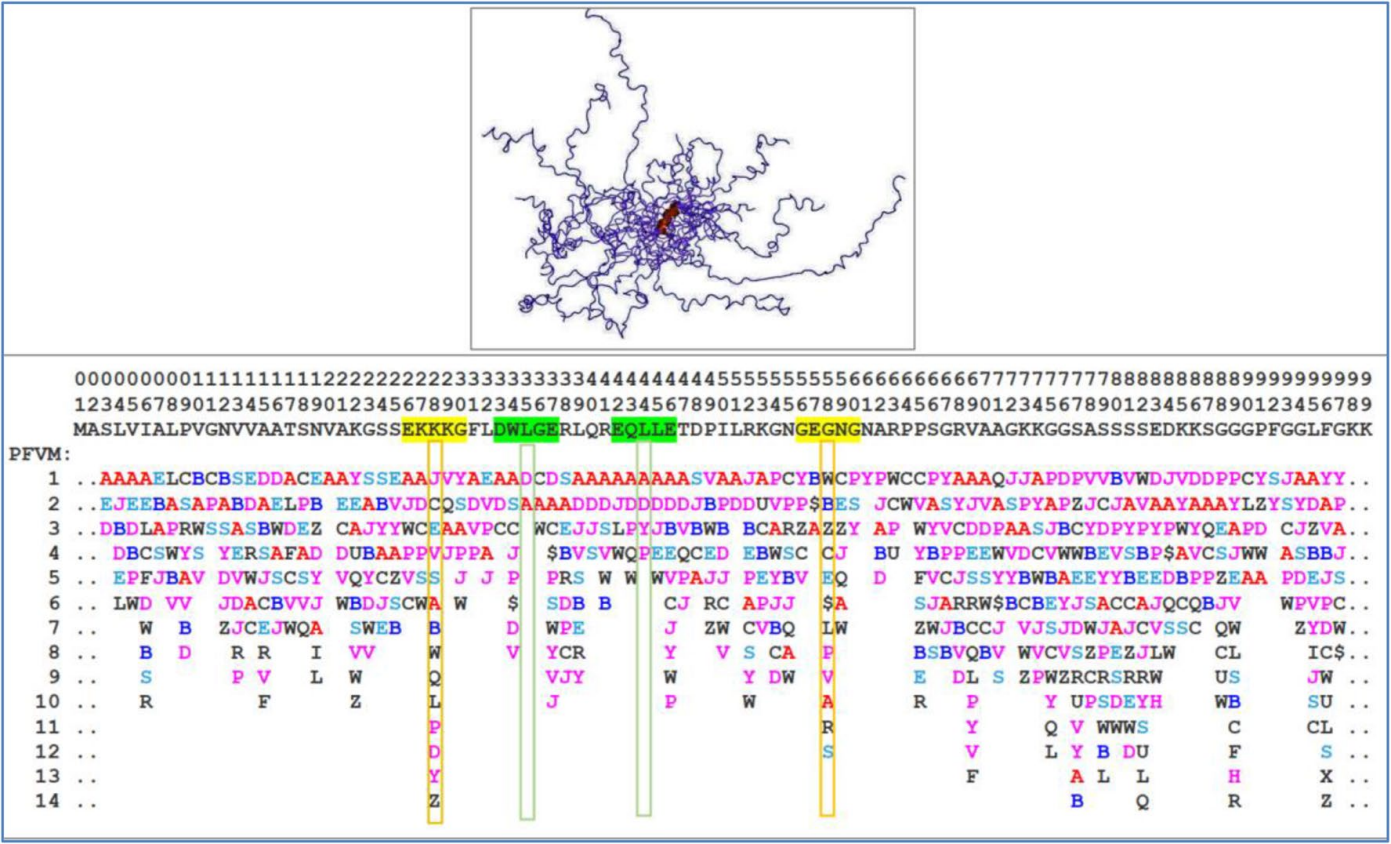
PFVM of protein Q8GT36_SPIOL (Thylakoid soluble phosphoprotein TSP9 protein) and images of its 20 conformations with largely flexibility. The PFVM is obtained from its sequence. The PFSC letters are marked by colors: red is for typical helix fold; blue for typical beta fold; pink and light blue for folds with partial helix or beta; black for irregular folds. On protein sequence, the green color marked five amino acids has similar folding shape on the column in PFVM; yellow color marked five amino acids has different folding shapes on the column in PFVM.

The protein intrinsic disorder may be displayed by either folding difference or spatial orientation difference. First, the folding difference in IDP is able to be revealed by the replacement of top PFSC letter with other letters in same column in PFVM. During replacement, the folding shape may have either similar fold or dissimilar fold. Specially, a larger change may involve different folding types. For example, an alpha helix is changed to beta strand or irregular fold etc. Second, the spatial orientation difference in IDP is easily to be observed by visualization if 3D structure is available. For example, the thylakoid soluble phosphoprotein TSP9 protein occupied larger geometrical space for transformation of multiple folding conformations (Fig. 10 top). However, it is not every residue in protein to make equal contribution for fragment with larger turn, and only some of residues play a key role for protein fragment with larger topological turn in space. The PFVM is able to expose these key residues because the PFSC letters on each column have the information for local folding change in protein (Fig. 10 bottom). Some of local folds are similar in same column, which does not turn the fragment too far in space. For examples in Fig. 10, the PFSC letters in column corresponding the residues of “DWLGE” in position 33-37 are “D” and “A”, which have similar folding feature of alpha helix; the PFSC letters for “EQLLE” in position 42-46 are “A”, “D”, “Y” and “P” have similar folding feature of alpha helix. In these cases, the replacement of PFSC letters in same column would not cause a larger turn in space. In contrast, the PFSC letters for “EKKKG” in 26-30 are “J”, “C”, “E”, “V”, “S”, “A”, “B”, “W”, “Q”, “L”, “P”, “D”, “Y” and “Z”, which 14 different folding patterns include alpha helix, beta strand and irregular folds; the PFSC letters for “GEGNG” in 56-60 are “W”, “B”, “Z”, “C”, “E”, “S”, “L”, “P”, “V”, “A”, “R” and “S”, which 12 different folding patterns also include alpha helix, beta strand and irregular folds. In these cases, the replacement of PFSC letter in same column may cause a larger turn in space for conformation transformation, i.e. the end of fragments may be turned far away due to the folding shapes much different. Therefore, the PFVM is able to reveal both folding change and spatial orientation, which will help to study the relationship between IDP and biological function.

### More applications

The PFVM may be utilized into basic research of protein folding and promoted the bimolecular medicine discovery because the PFVM is able to provide local folding variations as well as multiple folding conformations for various protein systems. Since genetic code development, over 250 million protein sequences have been known, but so far only less than 0.1% of proteins have the given 3D structural data in PDB and about only about 4% of proteins with computational simulation calculations. Recently developed AlphaFold is able to well predict the 3D structure from sequence and establish its database, but it is limited by a static status of protein structure. The PFVM does not only reveal local folding variations, but also it is able to construct a massive number of folding conformations for any protein, which is significant for the prediction of native protein structure and the protein folding project. What is more important, the alphabetic digital description of folding patterns with PFSC code in PFVM made an astronomical number of folding conformations to be simply stored in a matrix. The PFVM may be widely applied in several aspects. First, the comparison of protein conformations can be carried by PFVM if the sequences are known, and by PFSC string if the 3D structure is given^66^. Thus, the conformation comparison will not be limited by need-to-known 3D structures. Also, the protein comparison and homology study may be integrated with inclusion both sequence and conformation. Furthermore, the high-throughput screening protein database to discover the proteins with similar conformation is to become possible with PFSC and PFVM. Second, the PFVM has ability to expose the difference, which is caused by either protein mutation or species protein differentiation^67^. The protein mutation or species differentiation normally involved the replacement of some residues in sequence, and in most cases the protein 3D structures are unknown before change or after change. Also, it is very difficult to distinguish the structural changes by substitution of few residues with using experiment measurement or computational simulation. However, for substitution of residue in sequence, the PFVM is able easily to reveal the difference for protein mutation and differentiation, and clearly display the difference in matrix. Third, the PFVM is able to provide the lead information for peptide and protein design, especially for bimolecular drug discovery^68^. Currently the methods of gene recombination is able to create new protein in biotechnology, medicine and research with rational protein design. The goal of protein design is to obtain new protein sequence with novel function, so the optimized design achieves new protein folding with a specific conformation or has certain flexibility in conformation with specific activity. With integration both physicochemical property and folding patterns from PFVM, new protein will be easily optimized for directional design. The PFVM is able to provide significant lead information for peptide and protein design, which will benefit the research, such as vaccine, antibody and hormone etc. Therefore, the PFVM provide a new means for biological molecule research and medicine development.

### PFVM server

The PFVM server to predict protein intrinsically disordered can be found at a link in http://www.micropht.com/. User can enter any amino acid sequence as input, and the PFVM will be output as result on screen or save a file as output.

### Supplementary Information

Supplementary Information 1.

Supplementary Information 2.
