# Supplementary material for: Prediction of folding patterns for intrinsic disordered protein
Source: Sci Rep. 2023 Nov 21;13:20343. doi: 10.1038/s41598-023-45969-5 (PMC10663623; doi:10.1038/s41598-023-45969-5)
Supplement: Supplementary file 1 — Supplementary Information 1. [file 41598_2023_45969_MOESM1_ESM.rtf]

PFSC Illustration


The diagram of 27 Protein Folding Shape code (PFSC): Three blocks represent three regions 
of pitch distance; the nine vectors in each block represent the nine folding shape patterns; each 
vector is simultaneously represented by a letter, a folding shape pattern and an arrow. The 
vector characteristic is represented by an arrow line, which the initial and terminal points 
represent the N- and C-termini for the PFSC vector respectively. The ''a'', ''b'' or ''*''at each end of 
vector indicates the folding features similar to a-helix, b-strand or random coil respectively.


Reference
²	Yang J. Comprehensive description of protein structures using protein folding shape code. Proteins 2008;71.3:1497-1518.
²	Yang J. Protein Structure Fingerprint Technology. J Bioinform, Genomics, Proteomics 2018: 3(2): 1036
²	J. Yang, W. X. Cheng, X. F. Zhao, G. Wu, S. T. Sheng, Q. Y. Hu, H. Ge, Q. S. Qin, X. S. Jin, L. S. Zhang, P. Zhang. Comprehensive folding variations for protein folding. Proteins. 2022; 1- 22. 

============================================================================================================================================================================================================================================================================

>sp|P05067|A4_HUMAN Amyloid-beta precursor protein OS=Homo sapiens OX=9606 GN=APP PE=1 SV=3
MLPGLALLLLAAWTARALEVPTDGNAGLLAEPQIAMFCGRLNMHMNVQNGKWDSDPSGTK
TCIDTKEGILQYCQEVYPELQITNVVEANQPVTIQNWCKRGRKQCKTHPHFVIPYRCLVG
EFVSDALLVPDKCKFLHQERMDVCETHLHWHTVAKETCSEKSTNLHDYGMLLPCGIDKFR
GVEFVCCPLAEESDNVDSADAEEDDSDVWWGGADTDYADGSEDKVVEVAEEEEVAEVEEE
EADDDEDDEDGDEVEEEAEEPYEEATERTTSIATTTTTTTESVEEVVREVCSEQAETGPC
RAMISRWYFDVTEGKCAPFFYGGCGGNRNNFDTEEYCMAVCGSAMSQSLLKTTQEPLARD
PVKLPTTAASTPDAVDKYLETPGDENEHAHFQKAKERLEAKHRERMSQVMREWEEAERQA
KNLPKADKKAVIQHFQEKVESLEQEAANERQQLVETHMARVEAMLNDRRRLALENYITAL
QAVPPRPRHVFNMLKKYVRAEQKDRQHTLKHFEHVRMVDPKKAAQIRSQVMTHLRVIYER
MNQSLSLLYNVPAVAEEIQDEVDELLQKEQNYSDDVLANMISEPRISYGNDALMPSLTET
KTTVELLPVNGEFSLDDLQPWHSFGADSVPANTENEVEPVDARPAADRGLTTRPGSGLTN
IKTEEISEVKMDAEFRHDSGYEVHHQKLVFFAEDVGSNKGAIIGLMVGGVVIATVIVITL
VMLKKKQYTSIHHGVVEVDAAVTPEERHLSKMQQNGYENPTYKFFEQMQN


PFVM
     00000000000000000000000000000000000000000000000000000000000000000000000000000000000000000000000000011111111111111111111111111111111111111111111111111111111111111111111111111111111111111111111111111112222222222222222222222222222222222222222222222222222222222222222222222222222222222222222222222222222333333333333333333333333333333333333333333333333333333333333333333333333333333333333333333333333333344444444444444444444444444444444444444444444444444444444444444444444444444444444444444444444444444445555555555555555555555555555555555555555555555555555555555555555555555555555555555555555555555555555666666666666666666666666666666666666666666666666666666666666666666666666666666666666666666666666666677777777777777777777777777777777777777777777777777777777777777777777777
     00000000011111111112222222222333333333344444444445555555555666666666677777777778888888888999999999900000000001111111111222222222233333333334444444444555555555566666666667777777777888888888899999999990000000000111111111122222222223333333333444444444455555555556666666666777777777788888888889999999999000000000011111111112222222222333333333344444444445555555555666666666677777777778888888888999999999900000000001111111111222222222233333333334444444444555555555566666666667777777777888888888899999999990000000000111111111122222222223333333333444444444455555555556666666666777777777788888888889999999999000000000011111111112222222222333333333344444444445555555555666666666677777777778888888888999999999900000000001111111111222222222233333333334444444444555555555566666666667
     12345678901234567890123456789012345678901234567890123456789012345678901234567890123456789012345678901234567890123456789012345678901234567890123456789012345678901234567890123456789012345678901234567890123456789012345678901234567890123456789012345678901234567890123456789012345678901234567890123456789012345678901234567890123456789012345678901234567890123456789012345678901234567890123456789012345678901234567890123456789012345678901234567890123456789012345678901234567890123456789012345678901234567890123456789012345678901234567890123456789012345678901234567890123456789012345678901234567890123456789012345678901234567890123456789012345678901234567890123456789012345678901234567890123456789012345678901234567890123456789012345678901234567890123456789012345678901234567890
     MLPGLALLLLAAWTARALEVPTDGNAGLLAEPQIAMFCGRLNMHMNVQNGKWDSDPSGTKTCIDTKEGILQYCQEVYPELQITNVVEANQPVTIQNWCKRGRKQCKTHPHFVIPYRCLVGEFVSDALLVPDKCKFLHQERMDVCETHLHWHTVAKETCSEKSTNLHDYGMLLPCGIDKFRGVEFVCCPLAEESDNVDSADAEEDDSDVWWGGADTDYADGSEDKVVEVAEEEEVAEVEEEEADDDEDDEDGDEVEEEAEEPYEEATERTTSIATTTTTTTESVEEVVREVCSEQAETGPCRAMISRWYFDVTEGKCAPFFYGGCGGNRNNFDTEEYCMAVCGSAMSQSLLKTTQEPLARDPVKLPTTAASTPDAVDKYLETPGDENEHAHFQKAKERLEAKHRERMSQVMREWEEAERQAKNLPKADKKAVIQHFQEKVESLEQEAANERQQLVETHMARVEAMLNDRRRLALENYITALQAVPPRPRHVFNMLKKYVRAEQKDRQHTLKHFEHVRMVDPKKAAQIRSQVMTHLRVIYERMNQSLSLLYNVPAVAEEIQDEVDELLQKEQNYSDDVLANMISEPRISYGNDALMPSLTETKTTVELLPVNGEFSLDDLQPWHSFGADSVPANTENEVEPVDARPAADRGLTTRPGSGLTNIKTEEISEVKMDAEFRHDSGYEVHHQKLVFFAEDVGSNKGAIIGLMVGGVVIATVIVITLVMLKKKQYTSIHHGVVEVDAAVTPEERHLSKMQQNGYENPTYKFFEQMQN
Predicted Results:
   1 ..VAJAAAAAAAAEEAAABESZASBVDAJAJSAEEEWYAPESEWSBVJWYVRLCJYPPCVWSBVEDPAAAAAAADDPVAJEBVPVASAPCSSREAJBBWZAAAJDJWBBEEWRWSBABEPSDACAAAEWWCAPSBEEEBBVAJEWCYAAAAAAAAADDAAQYPSSVJELAVJBJCZDJBBJEEEEEWCAAJCWAJPBVVAQYYJAADABWPYYDJAWDQYAYAAAJEDADAAAAAAAEBAASVAJVJVVJSCSAEAAAYYVVAVAAJAARBDAAEPPPEEJAYAAAADAAPCPAADBWYWVPSASWRWSBBSAVARLRWJBAYAAPCYASWVPYAAAADDYBCSAADSAAEDWPCSAADPVASWCCYYAVPYAAAVYAABWVVAPPAAAAAAAAAAAAEAAAAAAAJAAAAAAAAAAAAAAJWCSAPABEAAAAAAAABDAAAAABAWAVAAAAAAAAAEAAAAAAAAAAAAAAAAAAARASVCCCVDAAAAAAADAAAAAAAYAAAAAAADDAAADDCVDAAAAAAAAAAAADAADEAAAAAAEAAVBBJVAAAADAAPDAAAAAAAAAAAPAAAAAAAJAVBAEWVPCAVPWWAYASWVEAAABACYWZAJWYAAJAVDPPDWYAAPBYAEVAAVCSCBAAAVAYACBWAWSPPYJAAAPAADAAAABBEVBJEBUVJJEEEEEEEEEEEYJBWSEWSEEBEBEVAJEEEAAAAAAAAEAAAASZAJWPEPJREBWAAAWCYAAAAAAAAAQZWSWSVAAAAAAJB..
   2 ..AVADDDDDD B  DDBYBBVP JAAJPWCPBYAB   DBY   SJDQ PSV SVQAXJ YC VBAJZWB   A JCDAREWJADACYPRBEAV LRCYPYVLBWSCSA BSSB BAWAPSWBYDSBJCYYJEEBBBEAJ PWLAZ D WDDDD AAY JDJLAWS ERJSSWP$AAAVARABRB SBVASAPPVWBYSACSWVV JW  WAPBELWPSVBPVDAAAVAJWDDRDDADDJVBYPASSSQJBEBADRECPRYDAWVAJSAJQSDWAAAAWBCABCJVADDAAACDBPCSJEJBWB   BAVWWYPSW DRQWLPJYJAPJBCJADDDDAAJCWRCVAAJDAEA VBDSBBSWBBSBZADAWZJBDAADDABAWZVJVPDDDDDDDJDDAJDDDDCDA DDD  DDDDDDJSABAYSDYABEDBDD SDAAJYWDYADCDADDDE DDVDDEDDDVDDVDCDDDDDDDVJADDLJBBSAEPQSDJDADBJDDJDADDDDDDDAADJBJQJYAPDBDDDDDDVDCEE AWD DDDSABDAAAVADDDDAWJYADVDDDDD WE AYJDDD P B   L BWZDDEAACJDJEADEBCACSBPYEQAWDYABY  J JBPCWCVJVABJBVCSSVCDCSAVAAAEJRAAVASJDBWZAEVEBW ABVEBEEPYBSBAWRBBBWAVAAWVWSVCSBEAEBBVPBAABBBDEDEDADDDYB BWB BA SBEBDDJEWZDDDDS DDDDSCJBBSDJBEWSAJ..
   3 ..CBPEBEBE  W  PYSAPWJV SPYBADSJD DD    C     BAP J W BZJVAP  W PVQYDR      BR DDAEAEWCDSWCEVDD WE VSJ  VBDSYB  EB  EVYDFRJJBJRABVZSWLRRA A B  A D    LS WE VP   JACBAA  EACAPWQVZYWCAWW    VCVBCWYAJAAJDAAAYB     CSSE VAVAWVSBWEJECYVDQEWBBBAYDAJZACYBYPYYBCBYEDQCEAWJLYWEEPAA ESEEEJYDESCSDWV  JB DSAWVAPY EBR   J SAJ B    DD VVSVAJWAAAAD      AJ CSSWDSJDWD  CVVAJCSCCVPEDVJBVDEPDD LDEBAYYADD    J VDEEDB    D$C       YJ YWWYBPWACADWJDEW   D DWDZYBDWJAWYP EJ   D AD   D PDJDSSCC JWRDDJCBWWS CDDDD DLS WDJCY D     JVE  DV BPC$YCVSB E    YAB  L     CQCEEPVWSWJJ EJWD JCRPJE  PB CEVBBP D V   W SDWVAADDVAEAPSJCWDEWYA  PEEZVDB      CZJJBAW UDP$LAWBJYJVYZVPVDJ PVJWSBVSBEVDPBDCD  B AAALVAVABWVAJAAAVBWBSVBJCCJBAAJSJWYWADDEWDBBED DEJPJC D L DQ  WJYWJECSVSSEFD   QZJECDCEWDVJVJBA..
   4 .. DWPJBEV  J  BSPSWEBQ  YBDDJP         J       $   Y PC YPD  V JWYBYV      ES PSO CDSBYBSJW B  ES  YB  CABJ          AYEEEWCSVWPY$  R DD   E  R         JY PB   ADB     DP CVVYYBWESDB     YPE VYASSJBIVWWBJJ     PZJQ   CJ  DWVVBBWVPBYWBRJCPJBDSWWWBWABAAY$CJJVV SWZDJDBBJSVJ WBDDDDAESZWDPDE  DW RBSA JS   D    R RCS D       BJY  PJBY  V      ZP VBY BDQBVY  RY V RBRP SPCCECWCJCWB WEPYJVA Y       PPCBBD    SV         B WPCBRSDCJYVDAY     J E$SBD ZSSQ DW VD     JB     BBWZJBY  BLWWEVPPSSR       BW  D L          W       WZWSWD        SBW  D     PJEVWEYB VPC BCBA VDE  J  J   VCE E J     R  BYBJD V ZSB WBDEWWBBZ  SRBEBW       SCV LDC BY YWWJWEDPCDYJBLPC  WWCJY VVJYJJDWS     EBSWRJAYRVUVARDJBSAPEASCBBAASUWVAEJARBBWDEERBB BPEJZW W C PJ  ACQESBBPWCW CW    ABJEFJWJEJDBYVP..
   5 .. YCV SWJ  L  VJJ DLSY  CCRSC                  Y      A SVA    SSVSB       S  SVD SRCDBJBVY W   W  QS    PP          QWRAVADECPC V         A            BC      WWJ      B   Y PPDJPBL      WY   CDVWJWPBCP P      DRY   WZ  WDEBDWECYEEZCSWWJPPBAJVZAYDSBJJWWWCYJ Y  PDPYR   P  JJJJWV WJSJCBW      V C VD          WDY         AWW  E E   P      PV BJJ RCPSA   U  W  DE  EBVBWJAWCBCP   C  BB           VJ                 V V  JDJSJWQ  SB     P PJBDL CCBB  C  P      W      WVBELE  V D J JCP         SB  E P                  S Y EJ        P    B     EBWRD EE YV  RSDV PEC  B  Q    DJ            VPJ J C LBP YALDBVPLV  BPCVQ        PAC SSA       D AJSSWPQJSCB  YBVPP    BVERCF     DWVASBCCWDBPBVUSAJBEBSAPVSWJDVBADABVDSVJ J DW    QQDY   E     SWVCYRJBCPR LC     W AE  CYWVJBWW..
   6 .. EYC   B      PE   W   BPEWP                  V      S  ZW    WYDDW       R  EW   YYJSVJ L        C     VW          RBLJSDVBESS P                                F      W     S JCEJS      YS   W  YSDBVRC        BCW   JW   JCCSVBBDC JDEYVCVCCCCB RP WC RSVBLW  B   EQP       LWWWC   VDERE       Y L DA          CJP         JCD    V   C      UY F$W E  RB      S   J  WJZYYS  PJ     J  CD           WL                 Y B  PPAVBVW  RP        VEC  HZVE            Y        WB W  Y J I AE          PC    Q                       P        W    A     WSJBB    CY  LBV  CP   P  S    SV            ESW B J BPZ B SLECL    JYL $        YJW AVZ       E   BW DWYWJL  CYJAS    EYWFER     WSJDASWEJYSLLPSDDCDWPJCBWJVPRDPDRDPSSRSD V       PWCE         VP BBSAJ Y   B     C LL  VPDPPVDS..
   7 .. CBB   L      ZR   C   SWZBV                  J         BY    Y WZ        C  RA    B     V                          B  C R RWDE                                  A      E        A F       SD      EP WP S        CZS   AB   E DRYJSWJ LJ SJVCYYWSE VJ ZP AVJCYB      BWD       RRRRR    RRE          E PB          JPV         PQP        R         APB J          Q   L  JRJ PE  R      R  DS           SS                 P C  QSC ZBC  WR          S  JYPD                     EV J      S EY           J                            W        B    C     DDVYJ    B   SD   WB           YP             V      DWD J W RSD    RB  C        APS CRB            Y VDEPSS   CYBV       P Y     ZVDJWDPDAAWJSSPLSDJDYCPAJPBRVWDYSLWCFWWS         YBVA         CR SPDL            P DV    EWSDSV..
   8 .. JEJ   W       W   Y   WEVCQ                                  Z BP        W   B          C                          U  B   VJRL                                                    C       JP      C  Z$ V        WA    BP   P W  PRSV RE VSWSEZDVS C  A  WJSLDJ       BS       VBBBB    EBU          Y BC          AVB         SYQ        S         EVD L          Y   V  VUS CA  W         JW           Y                  Q J  DCE  ES  P           V  SD S                     RW Z      Y BJ           V                                     Q    Y     LPF      J   WE   BL           ZY             A      VCY Z   LRJ    CV  P        ZVZ FW                CQ      DBW        V B     YDPVBWSVVJLRVJZPYVFVDDYVYADCJSCCVUSYJJ            SWJ         JS JWPV            D R     RBDCEE..
   9 .. SSW           C   P    RSV                                     JV        Q   C          R                             W   CDJA                                                    S       DC          S          JB    U$   S P  SJQL VV PYY VWEBC D  U  PDYPSS       CE       AYYY     JPS            QQ          P D         W B        Z         PWZ C          C   A   W   L  Z         P                               S P  VEV  LV  C               P V                      P P        YW                                                 V           CR      S   J    Q$                                  R$      F     Z   W            J                  W      L E          D     SCCCDYDPDSJBWDJVJULSWPJYPYFDULVJWJCBYV            VBP         LY L YF              Y     YCYQCR..
  10 ..   L           D   A    SWY                                     Z         L   Y                                            PB                                                      Y                   E           E    ZC   C    YPBP    QDE  JLPY E  D   PDRWZ        L       YCCC     Y               R            E           H                  YY  P          E   P   A   V                                            W Q   W       L               E                          R         Z                                                             $D               ES                                          D     U   S                                      S C          J     PRWQPCESYLDYDWVULYCCVEDDRZLWFJURPQEELC            CPQ         RA Y WP                      CWPC..
  11 ..   S               $    JP                                                                                                 WY                                                                          Q                SE        DWCS    ZIS  LYEZ U      EIV P        C       CLLL     V               V                                           W   W              W   D   Y                                              S                           H                                                                                                  LL               LY                                          L     V   Y                                        Q                CYYSCUIWCRRDFYRCPRUUQYQZADYVWPYPCIRDCP            YEV          V   CS                      LEUD..
  12 ..   Y               Q    VC                                                                                                 YP                                                                                           YV        L EY     Z   R FQ W      LZ  C                F        L               Z                                           L                  D   V                                                                              V                                                                                                  WS               SW                                                W                                            F                JZ$YYEQZLCCIPLIFUZPRRQZEZUPYLZSWLSZRVQ             QZ              FY                      UR L..
  13 ..                                                                                                                                                                                                                        E         R LZ           L         R   R                P        P                                                           D                  Y   C                                                                                                                                                                                 Y                YR                                                Y                                            L                RPQPJZBRPPYSYQYRZPYIUL$LI$ZLPYQLFYQLP              S               LR                         Q..
  14 ..                                                                                                                                                                                                                        I         U                        Y                             Z                                                                                  Q                                                                                                                                                                                 R                Z                                                                                              R                QQIZZLUIFZPCCFFWCWSLC$ERDQEFQCIIUPUZW              L               VZ                          ..
  15 ..                                                                                                                                                                                                                        K                                                                                                                                                                                                                                                                                                                                     V                                                                                                                                 $R$$QL UUFFUIW R XJZ UQQEUIYQ Z CDF               R                                           ..
  16 ..                                                                                                                                                                                                                        X                                                                                                                                                                                                                                                                                                                                                                                                                                                                       LZ LRH  FIZ U     HI RILHRZZR   FII               U                                           ..
  17 ..                                                                                                                                                                                                                                                                                                                                                                                                                                                                                                                                                                                                                                                                                                   Q R  IQ  Z     Q$  U$ I$ $   RLQ                                                           ..
  18 ..                                                                                                                                                                                                                                                                                                                                                                                                                                                                                                                                                                                                                                                                                                     Z   Z         H   H QU      H$                                                           ..
  19 ..                                                                                                                                                                                                                                                                                                                                                                                                                                                                                                                                                                                                                                                                                                                   L     $       $                                                            ..
  20 ..                                                                                                                                                                                                                                                                                                                                                                                                                                                                                                                                                                                                                                                                                                                         H                                                                    ..


>sp|P10636|TAU_HUMAN Microtubule-associated protein tau OS=Homo sapiens OX=9606 GN=MAPT PE=1 SV=5
MAEPRQEFEVMEDHAGTYGLGDRKDQGGYTMHQDQEGDTDAGLKESPLQTPTEDGSEEPG
SETSDAKSTPTAEDVTAPLVDEGAPGKQAAAQPHTEIPEGTTAEEAGIGDTPSLEDEAAG
HVTQEPESGKVVQEGFLREPGPPGLSHQLMSGMPGAPLLPEGPREATRQPSGTGPEDTEG
GRHAPELLKHQLLGDLHQEGPPLKGAGGKERPGSKEEVDEDRDVDESSPQDSPPSKASPA
QDGRPPQTAAREATSIPGFPAEGAIPLPVDFLSKVSTEIPASEPDGPSVGRAKGQDAPLE
FTFHVEITPNVQKEQAHSEEHLGRAAFPGAPGEGPEARGPSLGEDTKEADLPEPSEKQPA
AAPRGKPVSRVPQLKARMVSKSKDGTGSDDKKAKTSTRSSAKTLKNRPCLSPKHPTPGSS
DPLIQPSSPAVCPEPPSSPKYVSSVTSRTGSSGAKEMKLKGADGKTKIATPRGAAPPGQK
GQANATRIPAKTPPAPKTPPSSGEPPKSGDRSGYSSPGSPGTPGSRSRTPSLPTPPTREP
KKVAVVRTPPKSPSSAKSRLQTAPVPMPDLKNVKSKIGSTENLKHQPGGGKVQIINKKLD
LSNVQSKCGSKDNIKHVPGGGSVQIVYKPVDLSKVTSKCGSLGNIHHKPGGGQVEVKSEK
LDFKDRVQSKIGSLDNITHVPGGGNKKIETHKLTFRENAKAKTDHGAEIVYKSPVVSGDT
SPRHLSNVSSTGSIDMVDSPQLATLADEVSASLAKQGL

PFVM

     00000000000000000000000000000000000000000000000000000000000000000000000000000000000000000000000000011111111111111111111111111111111111111111111111111111111111111111111111111111111111111111111111111112222222222222222222222222222222222222222222222222222222222222222222222222222222222222222222222222222333333333333333333333333333333333333333333333333333333333333333333333333333333333333333333333333333344444444444444444444444444444444444444444444444444444444444444444444444444444444444444444444444444445555555555555555555555555555555555555555555555555555555555555555555555555555555555555555555555555555666666666666666666666666666666666666666666666666666666666666666666666666666666666666666666666666666677777777777777777777777777777777777777777777777777777777777
     00000000011111111112222222222333333333344444444445555555555666666666677777777778888888888999999999900000000001111111111222222222233333333334444444444555555555566666666667777777777888888888899999999990000000000111111111122222222223333333333444444444455555555556666666666777777777788888888889999999999000000000011111111112222222222333333333344444444445555555555666666666677777777778888888888999999999900000000001111111111222222222233333333334444444444555555555566666666667777777777888888888899999999990000000000111111111122222222223333333333444444444455555555556666666666777777777788888888889999999999000000000011111111112222222222333333333344444444445555555555666666666677777777778888888888999999999900000000001111111111222222222233333333334444444444555555555
     12345678901234567890123456789012345678901234567890123456789012345678901234567890123456789012345678901234567890123456789012345678901234567890123456789012345678901234567890123456789012345678901234567890123456789012345678901234567890123456789012345678901234567890123456789012345678901234567890123456789012345678901234567890123456789012345678901234567890123456789012345678901234567890123456789012345678901234567890123456789012345678901234567890123456789012345678901234567890123456789012345678901234567890123456789012345678901234567890123456789012345678901234567890123456789012345678901234567890123456789012345678901234567890123456789012345678901234567890123456789012345678901234567890123456789012345678901234567890123456789012345678901234567890123456789012345678
     MAEPRQEFEVMEDHAGTYGLGDRKDQGGYTMHQDQEGDTDAGLKESPLQTPTEDGSEEPGSETSDAKSTPTAEDVTAPLVDEGAPGKQAAAQPHTEIPEGTTAEEAGIGDTPSLEDEAAGHVTQEPESGKVVQEGFLREPGPPGLSHQLMSGMPGAPLLPEGPREATRQPSGTGPEDTEGGRHAPELLKHQLLGDLHQEGPPLKGAGGKERPGSKEEVDEDRDVDESSPQDSPPSKASPAQDGRPPQTAAREATSIPGFPAEGAIPLPVDFLSKVSTEIPASEPDGPSVGRAKGQDAPLEFTFHVEITPNVQKEQAHSEEHLGRAAFPGAPGEGPEARGPSLGEDTKEADLPEPSEKQPAAAPRGKPVSRVPQLKARMVSKSKDGTGSDDKKAKTSTRSSAKTLKNRPCLSPKHPTPGSSDPLIQPSSPAVCPEPPSSPKYVSSVTSRTGSSGAKEMKLKGADGKTKIATPRGAAPPGQKGQANATRIPAKTPPAPKTPPSSGEPPKSGDRSGYSSPGSPGTPGSRSRTPSLPTPPTREPKKVAVVRTPPKSPSSAKSRLQTAPVPMPDLKNVKSKIGSTENLKHQPGGGKVQIINKKLDLSNVQSKCGSKDNIKHVPGGGSVQIVYKPVDLSKVTSKCGSLGNIHHKPGGGQVEVKSEKLDFKDRVQSKIGSLDNITHVPGGGNKKIETHKLTFRENAKAKTDHGAEIVYKSPVVSGDTSPRHLSNVSSTGSIDMVDSPQLATLADEVSASLAKQGL
Predicted Results:
   1 ..CCVAALEBVCCDWPCDSWPSAPJWSJWAAAVVDA$SPWDYAAAWJWEWRWYQSWPPYAPSWYJJAJCWWAASBAWSAAAQECCAPAAAAPYWAASCYPWAYADPJBAACYAZWJAAQSAJWPAWAPPWEASQYAAPJBPCYSJVDWAAAPBYAASSCCCDYAWAAAPWWARPCYEZAJPDWPYAAAAAAAJAJSAAJJSCBVPBQEWRWCAPZAAAJVPPYVDAACYAACCJAZYJZAYJAJYWAAAAAAABAPYCCSSYJAWBPSVVDAAVVVPCCCYAJCCPSWVEDYQJAACCYAEBBEVEWWCDVARADAASYAAWCAAAJYJSVWPPYAAJCCPAYZDJAAAAPCCSWAJWCWCCC$CPSDWCWAAAAWAAAAVPZQBFAWYJAJJDRBPBBAPAAAASPSAWCSVPCBYVJDCBPABSCPCSEWWCCCAPRAAJAAVDBWPJVVPAVAEWAVJPQYPJADAAEPPACCYYJPAAYABESWCJVPCCCCAJCSJPEWCCEPJBWVVBWJYAPYAPSJYPAYCBJLCCCAWBCESABBEAECCJCCCAAVCPAAASPSWWBCAAAAAAAVAAAAAAAAJBVJVWCEEAAAJAABBAJBJVJBPAVPASEBWBVYPCEEEEJBCBBVAAJEEEBWSEASBBBBBJYLREEDBEVEEEZVASBEBEAASBAJBEBBBVJZBJEEEVEEEBBAWAAAAABDQSWSAAECBBSJW$YBCVAAJPDJAZYPBDWAAAWBBAADAAAJACYEAAAA..
   2 ..PB      A  WVWZRARVZ A Q VB     Z CCVYQSDWPPS YPSVPPBAV VJYJAVAAVAWDCYCAAPCADJDYZPAYYDDDD V  VBWVCYBADAQAYWJWBPYAVYD YJSCWZZWUYBSDDPAJJAVECPSAEYA DDP CA WCAJWJ CEADD WSVYACYBBY$$WZ WDDDJCJ DAWA DJ  A JSAQPWPJJVJVYBDDAAABAYAVJPPDWWSVCASWY WQYP  C  DDDJAVJVJWBAAYCRVAYBWAEDASCVSPFVPPDWCPAJVA AACYJSA A DBBSA YSSW DA DR DJPAPPDPVABYPAZZCJPY CJJYQWYYJJSVP BDA SYASYC WRJEACVEJDD REDACPPYAJPCCVAABAY YPWJEPDCW  DVP JJSEVJAVWVS WVBAYAW   WFCCV DPVSEAJVQZYWYVYDJABPCZJSVZEASBCWCPPZ   AJYASJAYPDYCCWVSFPPFCABWCWSB YAYUYCCVSJCBPWYAPCSAWCVCSPWRBYPJAEEEBBBWBCSWVCJAADVVVBWCPBS JVDPWVPYWESVBDWSACWYYBBYAVVBASESAPAAWAPVDCCQJASJBWQVCSJASVBWBWWABBBB  VSAWWAE EEWQSZSBCEERYBAWECDAAC CWSBEBEQBRCWQBESEPBBEAALEEDDVJSSD YBAAABDAWCCBEYCBZP JJ VCDCAVDPAVD  SVAESASVVPWAZADPZQ..
   3 .. P         P  RA PCJ J   SE       JPAAAWVCBLC JCECZJJJJ  SBREBCVPLEAYDPYDJYEJWWV YYJ PJ       PVQWJZDV JPSSYPAVVDBV  ZBBBABAE SAAWCESDDDPSWSADDSW  S  WD PVVABP VYEPP BZSZBAZASAPPYV  VSPD D EVDY  Y  P WZYJAYVS   CPDSWSYJWDBCY WSJYS C YAAB CDSL  D  JSRSVJWCABCJJAJSASCL VJPDD AAJSAC Y YCDAAZ USSVWWE B A  RB Z YE    CC JDQBBRJVDCJSABC$JBVJ AYPSAVZVCSJWF SSD VCVBZ  YCEBJ  ZD A DDWEJABADPJ ADQBJ Z ZABVPJPVL  VPB   J SAWBJS  ECACZB    SS J   SCCAVSPEAZEJYJBWLJAACPVDEPSJWYAIJES      VPPSJJASSJAAJWWWWYVSBPSW  AJS CJVPCPJVJCJSVYDVBSPVVJ SCESBWJWASPCP PBEWWVPEADBJECW     JVJJDWWYVJLDJDCLLSCPPWWDRWJVDJJVCZEAWDWYSDCWCAPYVJWYBBCJBWCWAAEJESV  WYJACJ   WYWCYBCFYSJQSSVBPPV B  YWAYJAYA FVJPYEBALLBBB  WB QVDJE  SCSR BBBSSDBVYPDJ    AAEWYJAJJAJ  JSESDEYEDSDSEDC BP..
   4 .. V         J  DW JYV      V       AVJJPAWVWJY B CBW$YYW   JEPWPBJSBVLZS WWJCBDPA JFD CS       EYAJCSZC DSAJVFJBCVAD  ARWA CBB VSL AACWBQCCAJRCVA   J     DYBWZV  WSY   CYVSY DA$VCA   SBWS C JWCS  P  C  ECW$SAA   BVVWBBZQDSCWD  B JE S JJC  AP W  J  CBCCJYCWZVWBCWPACJRY YDWSA JJWLCJ V  V DC  $CWC VV   S  BE   AS     W   AVVJYW PPWJS   DQL VZCV BVSSBADL AEP  ZSWS  CWWAS   S      BWVJSPEC VJDDV V AEJARCCWP    W      D JSY   YSJS     P  A   WSJBEAAJLSJWWC BREYSJCCARZW  ZVAYJW      D AVABBV SFSPAC  WBJCBF   DYJ JUASVBVCSJEEAZCDP  BYS  AP SVDARACJ  VWRSJWY VJCD BV      SVVJBAV$WYEVBJPWCWAYPJJSBWQBPYWVSSD SY$WZSSJVECACZWFYBAAPLASDYVDEC  Y DBJB   SVCAPAV VUBSJC AA      VCJAVSPJ AACVBPVBA AJD  L  JDBCV  AVYE E L WAVJVRP     WWABEWLSY W   RLDBCVPWAPDPBV DD..
   5 ..           B  SJ UAB      P       VBYPVDBD YB P F BSVB    AZBAFYRQPBASY JVAVYEBD  RP YW       WBPAEEJQ VYDVCJWWACYZ  DV   RJJ CDB B WY  WYYVVLAC   E      ACSRA  DJ    R WVD VR  Q$   AWV  P BDP   W  J  WJASPYV   JJS JW  Y DSZ  V  P Y  VP   A Y      VSVSBYZP ECDV BSBWE JV EB  PAVZ     A PI  JPVS YZ      AV   JR     E   $WDB Y SWCBV   ZSW JCS$ EDDVVCY  PBY  BBJR   J CB          JZWAVSVQ PQVWW   C SB DJYD           W AB     YWE     V      A EJCWCYWPBC Q LSRDPWABWSB     SWSV        DBPR B WVPW J   CCJLP   BDP PWEYB WZ SZPJSYJS  F R   V VBRSSCD   A BBV S  SJW FH      WBDPJCBCPDJBPPSAJSZSSPBDYVPCWVDWRWB VCADPACWBWAYAEZAPVBWCADESQWJWA  C  JV    C APAER BWPWAD J$      BVCVCD L  YAYDCADW SDS  A  D  B   JP V      JSBSCV     CJBEVPYV  B   Y WEPCDJBBBALJ WJ..
   6 ..               S EWA      A       BAB J JE AF S L E AP    EVJPS WW C BV E PYQLJZ  WW J        LSZYSRP  AEPBBSV B P   Q     CS BJW W  Z     WWPB    P      JWPEE   R    F  JV CJ  AC    ZS  V  E$   D  B  $BDCCLB    BY PC  C EB   W  F    BD   W         PRDS    VD B CECBW PL BC  ESWW     R  S   WZB JB      LL          Y   C ZS S  AZSJ   Y   BPAC A ZB WA  EC   APPB   V VE          SBEUZBSB WY CY   E VW WEDE           C QP     JDW     B        VRSPYACRAV Z A SCWBWDBAD     YCWY        V R    RJYL V   SYSEV   VWD WEJBW BE F VBV SE  P       WCSVWWW   B FAS W  WEY JP      CY WSJDWY VPVYDJQVCVZCPWPSEEBPP CCE  DCPRBPBYDSDPAJRSWCC SSCVBDPVP  D  LP    J PVC S RLWCDJ YZ      DY S$P    DPAWJPSD J V  Y  P  E   CW L      CDCBJJ      SFSSCEA        BVWRWBCCEDSS ES..
   7 ..                 XJ       J       SEQ Y P  CR W     PV    RBQ   EP S C  V  B RQ      Z        RJ SBW   SWZCE   D C   C     D  WPD          B RR           DPBSQ        V  XW P   Y     QC  W   V   B     AVZJJCP    S  ZP  V  V   Z  J    C               B W    Y  S J WVZ BP JJ  YLEP     W      Y R PR       R                C     C CW   C   YVZP C CP QJ  JR   SWVE   A  D          R DYQVYS BP S    U CC BW Y             RV     PBA     Y        $DW  BYAYA D S DWD BZ B       SBB          B    BLBA D   EWAS    $PV ZY     T B CSJ  F  W       CE DCRJ     LEB Z  ZW  L       J  SYBSBZ CQZ BSYAJJFSWCSEKJVEC DDV   BLSDYEWVPQYPSJAPDD  VYEPPZPW     VS    R ZJV W CDSBWW VY      SD  P     QYSCWSC  Y    D     V   RY B      EPZJV$      YWYPU$C         R D PEEWBC  JV..
   8 ..                 BB       R       YYW Z C  EW C     WS    CDV   S    V  S     R      E        JA B Y   Y CDW   J D   P     P  AR           D              FR YS        B  YZ S         PQ      Y   E     QZCWA         YY         A  L    Q               P         P   EAA    C    DAS     E      V   A                         S       YY   V    WW  P JQ BP  YW   VE V   B             W Y PC Y    V      DZ L                LA     WE      Z        DPB    WSS $    JB VA D       BVP               ESJF Y   YQP     S B  V     W R YWW  J          R$ L VV     SRE    CS              DSZ F RS  CEBPQAVZCEEYDPQRL YV    QQWZBP$ VS$SLYDSRY  FJJSS Q      ZB       WW   S CA V  D      ZJ  Y     S$CVAYV       Z     D   W  D      IWAW W      B  BAJE         L B YWSFJJ    ..
   9 ..                 A                Z   S Y  B  L     C     F Y        W        Z      S        CL       B JZS   S     W         C           Q              WY VW        J  P$           C       J           VDB         EZ         C  R    W               D         C   REP    P    YJB     J      Q   B                                 QF         B  S  W D    J    J     E             Y   DJ             PS Y                EY     VF                YU    CC$ A    BR Y  Y        A                  WV S    UV     W              BC              LJ C  E     YD     EL              VZP B F   EP ZWCAALYJ WVSCQ  Y    VYYJQVD EZSJDWLJW   R PC          D              AJ    Q      P         ZSWAYCW             H      W      PASA Y      L  LQCL           E  V  SW    ..
  10 ..                 V                         F  V           D          J               W        V            P                   E                              Y        Q               E       S           YYL         QQ         J  B                    W         E   Y      W     PJ     B                                             L         D     Y      Y    Y     F             C    Y             YY                         QS                SY    BUB      Q              I                          EL                    WF              PP J  S      J     YP              CDC C L   FR  $EJVRJ  YWYWE       Z A$Z J J ICE VL    Y CD          Y              DD           C          IEQRDR             R      J      VC             W W            P  Y  V     ..
  11 ..                                                          S          E                        A            L                   V                                                       V                     R         S$            V                    E             F            UE     I                                             Z         V            P    R                   F    R              D                                           L     JPE                     L                          RI                    UQ              YY           Y      Y              Z   R Q   QV  UFEBVP      S         JWD P   RIA Q       RW                                                 RL VWY             W      P      YY             C                          ..
  12 ..                                                          W          P                                                         L                                                       Y                               VD                                 F                          Y                                                                                L                   P                                                               Z     LQD                     R                          VR                     Z              A            Z                         V U   VZ  BRRR                  QYV     D$B Z                                                          D   Z                     C                     J                          ..
  13 ..                                                          L          Q                                                         F                                                       L                               KE                                 Y                                                                                                           D                                                                                         ERL                                                AY                                    F                                        W       SIY                   $       WQV                                                            W                                               R                          ..
  14 ..                                                                                                                                                                                       R                                                                                                                                                                              F                                                                                         DLR                                                ZF                                                                                      Q                    B       BUF                                                            B                                                                          ..
  15 ..                                                                                                                                                                                       T                                                                                                                                                                              U                                                                                         UDZ                                                 H                                                                                                           F       L I                                                            L                                                                          ..
  16 ..                                                                                                                                                                                       U                                                                                                                                                                                                                                                                         H                                                  O                                                                                                                   Z R                                                            Z                                                                          ..
  17 ..                                                                                                                                                                                                                                                                                                                                                                                                                                                                 I                                                  U                                                                                                                     Q                                                                                                                                       ..
  18 ..                                                                                                                                                                                                                                                                                                                                                                                                                                                                 Z                                                  D                                                                                                                     U                                                                                                                                       ..
  19 ..                                                                                                                                                                                                                                                                                                                                                                                                                                                                 F                                                                                                                                                                                                                                                                                                                ..
  20 ..                                                                                                                                                                                                                                                                                                                                                                                                                                                                 X                                                                                                                                                                                                                                                                                                                ..


 >sp|P04156|PRIO_HUMAN Major prion protein OS=Homo sapiens OX=9606 GN=PRNP PE=1 SV=1
MANLGCWMLVLFVATWSDLGLCKKRPKPGGWNTGGSRYPGQGSPGGNRYPPQGGGGWGQP
HGGGWGQPHGGGWGQPHGGGWGQPHGGGWGQGGGTHSQWNKPSKPKTNMKHMAGAAAAGA
VVGGLGGYMLGSAMSRPIIHFGSDYEDRYYRENMHRYPNQVYYRPMDEYSNQNNFVHDCV
NITIKQHTVTTTTKGENFTETDVKMMERVVEQMCITQYERESQAYYQRGSSMVLFSSPPV
ILLISFLIFLIVG

PFVM
     0000000000000000000000000000000000000000000000000000000000000000000000000000000000000000000000000001111111111111111111111111111111111111111111111111111111111111111111111111111111111111111111111111111222222222222222222222222222222222222222222222222222222
     0000000001111111111222222222233333333334444444444555555555566666666667777777777888888888899999999990000000000111111111122222222223333333333444444444455555555556666666666777777777788888888889999999999000000000011111111112222222222333333333344444444445555
     1234567890123456789012345678901234567890123456789012345678901234567890123456789012345678901234567890123456789012345678901234567890123456789012345678901234567890123456789012345678901234567890123456789012345678901234567890123456789012345678901234567890123
     MANLGCWMLVLFVATWSDLGLCKKRPKPGGWNTGGSRYPGQGSPGGNRYPPQGGGGWGQPHGGGWGQPHGGGWGQPHGGGWGQPHGGGWGQGGGTHSQWNKPSKPKTNMKHMAGAAAAGAVVGGLGGYMLGSAMSRPIIHFGSDYEDRYYRENMHRYPNQVYYRPMDEYSNQNNFVHDCVNITIKQHTVTTTTKGENFTETDVKMMERVVEQMCITQYERESQAYYQRGSSMVLFSSPPVILLISFLIFLIVG
Predicted Results:
   1 ..DJJJBWWDAEBEEACBVAAJAJPCCYAJADZAVZWCYQYJJYAAAEPPCCJRSYPYPSVWCYPYPSVWCYPYPSVWCYPYPSVWCYPWACELSEESJCCSVBCJBVBEBEEAAADAAESBJAJCSJWRWCSWCSCSBBWYJVAADAAAADAAAAPSVJLRWCCYAYJVJVAAAAAAAAADDAADDDADAAAQYJEEWZAAAAAAADDAADAAAAAAADAAAAAAAAJRCAWEBJCCYBBADBAEDADAA..
   2 ..A   E  WDDA  ZASABJA$ABSFPJV A VS PJRCACCFVSSP    BAYSWWAAWQPSWWAAWQPSWWAAWQPSWWAAWQPSWCCJVWBVJBVWWYJCBSSBEBEBWDEEAQPBEEVSAWABLECSRLECBBEEBVPYDDADDDDAJVDDJCYPEEEFVSWPSYCYDDDDDDDDDAADDAAADADDDPSBBBCYDDDDDDDAADDADDDDDDDADDDDDDDDA VPBADAPQSSEBAAWA DABB..
   3 ..Q   W   BBE  EVAQDSBDQWBLQ W P JE B SWBEABWPY     CWRCCZWQJAYCCZWQJAYCCZWQJAYCCZWQJAYCC  AWJEBWAWSBCBWVBEEJVJAVEZCEJSABACYEVYWSSLJCCBJAEWSVCCZJRVPPSBWDPWJWWWCRSPSSADAABWZZJBEPZBL ESYEJVEQSWEBJCPWLAJWE B   JBEB EB  JJVQEVCJPJBPS WSJ WDBPBJADEECD SW E..
   4 ..J       EAV  Y EJS LQSJJ V C Z SL S  J PBWPV      SJDRVSCCS$WRVSCCS$WRVSCCS$WRVSCCS$WRV  EBCJSACCJPBCESEWJACSSJSDDBYJSAWWDWAJPEBVDVBVWWRSJJPVAPSYQZJJV D S VSVWYLWBBPVCPPCYEYZ QJE BBJVY JJEBJWZASSWVVV       EW   W  QV WJBV JQJCC ADA EVFSCVJEV DS     ..
   5 ..W        J      P  DWZVV B P   WY    A   SJY      VBZB CJJCS B CJJCS B CJJCS B CJJCS B   SJEVABEBBSREJECVCSJCWACYSSVECWV$JPDBDCCAYBASBSCCPSBYS PZCCYVQ J   BAAFBBLYJVZPEBAVPV   EW WR BP RWJECQCVWLAB C       J        B  BZJ  SWQW YED JWJ VY  B  R     ..
   6 ..                W  EY AW C F    B    L   CCW      $CJ  J DBZ   J DBZ   J DBZ   J DBZ     VPSRJVWEELVWLWWJSVWVVUJJVPDLJDPACBQCERFDAFEWFEJAR ZWQ $QZYZPB B   YCBAUCJWPBJVWSWSVP   Z  LW  S BBZPVPDPIVRS P       S        Z  I    BPJV  JE ACS     J        ..
   7 ..                D  PZ C  W      W    D   VL       WYQ     QJ      QJ      QJ      QJ     IAYWQLJAPJWPPRVYACSWJCLPJVWWPVSDZCSESBLBWAPYPVWRV   B J$YE QP     DJ$BXYEFVJQYAAPBBQ          V S BQWJYZYQJ  Y                   L    PQSY  WR SQA     S        ..
   8 ..                B  V  S  Z      P        AS       YVV      C       C       C       C     PIPAWCVSLEESSYPCWWLAPBWBBWPBWJJYWSYIIFWFBEDJVFLL    J ZC    C     JBDZCVBRRSCBJYBE$Z          $   VYSS$WARC  $                   R    YC$B     V E              ..
   9 ..                   W            J        J        PP       Y       Y       Y       Y     BYACPPPYYFFYFJRLYPPRLQISYCECVCUBVV$WRDDEFPSALR F    C BS    J     ADZDPQR CCDWSVDJW           C   WJQVABEAF  Z                   S    VVBD     C R              ..
  10 ..                                A                  E       B       B       B       B     UCVYYRLRVR ARFYRPYAYRLYCWJCVRPDZBYJ A VP YFL L V    D CW    S     PPSCJA  WEWQULJPY           W   QRLCEJVFP                      V     EWP       W              ..
  11 ..                                Z                  S       R       R       R       R     HQBPLSRLR  LAPLPLLYPYPRVLUUYLQYPPDE C QJ WVR D        Y           R WYAD  EFSZCEE$F               PZYYW CJY                      Y     SEE       Y              ..
  12 ..                                                   D                                     WZIFCIYPA  FVLUARRRICSVWRRSIFRRQEQP L AS LRF          E           Z  JVJ  FLBEZIQC                CSBZB  US                             VQ                      ..
  13 ..                                                   L                                     YSOLFQ  U  Q A$UUFULIYBRPLBDYYCIQZZ   UY DU           V              SW   ZQEDDRSW                 CP S  CV                             YZ                      ..
  14 ..                                                                                         RUFDRY     R  FFFIF UFPIIYRUDLQSR$U       I           W              $D    RFUI                    VZ I  YI                             ZI                      ..
  15 ..                                                                                         DDRQI      U  QIIDQ ZDULFQFOUIZE$R        J                                YR Q                       U   O                             UR                      ..
  16 ..                                                                                         FLUUD         Z   Z  HHFUZI$ F LLU                                                                    V                                                         ..
  17 ..                                                                                         LR ZU                RFHZIHF   H I                                                                                                                              ..
  18 ..                                                                                          F                     U  LR   U                                                                                                                                ..
  19 ..                                                                                          H                     Q  $Z                                                                                                                                    ..
  20 ..                                                                                                                   Z                                                                                                                                     ..


>sp|P37840|SYUA_HUMAN Alpha-synuclein OS=Homo sapiens OX=9606 GN=SNCA PE=1 SV=1
MDVFMKGLSKAKEGVVAAAEKTKQGVAEAAGKTKEGVLYVGSKTKEGVVHGVATVAEKTK
EQVTNVGGAVVTGVTAVAQKTVEGAGSIAAATGFVKKDQLGKNEEGAPQEGILEDMPVDP
DNEAYEMPSEEGYQDYEPEA


PFVM
     00000000000000000000000000000000000000000000000000000000000000000000000000000000000000000000000000011111111111111111111111111111111111111111
     00000000011111111112222222222333333333344444444445555555555666666666677777777778888888888999999999900000000001111111111222222222233333333334
     12345678901234567890123456789012345678901234567890123456789012345678901234567890123456789012345678901234567890123456789012345678901234567890
     MDVFMKGLSKAKEGVVAAAEKTKQGVAEAAGKTKEGVLYVGSKTKEGVVHGVATVAEKTKEQVTNVGGAVVTGVTAVAQKTVEGAGSIAAATGFVKKDQLGKNEEGAPQEGILEDMPVDPDNEAYEMPSEEGYQDYEPEA
Predicted Results:
   1 ..AAAAAAAAAAACRAAAAAAJAQPAAAAASABCQARBEEAAAACQCADAAAAAAADAAYAAAEAAAAAEBVSEAAAADAAVVAAAAAAAAYJEAEYJBAASCAVJCVAYCAAAJJBSSVJJAJAVPCSPQYAJBVBY..
   2 ..VJDVCJJVBDQAAEWWDDRAVAASVDDQAVAYYYAEBBVVBJYYABADEDEDDSRRJADDBAWBVYPBEAADPEWEAJEYAPRJBBDDDAPBEVZBDQVJSBJPJBYQWDBVABCPJYSBVVJJWYJAZSVBYBJC..
   3 ..DCJQJPWSDZPSSBECJPSDPCSEDCJDYWEADVSARWEBEWADSREEWBDBCJASSDBVEVEYPDBAAEJJDREDBVJBSWYYJRJLCJEJJWVVSJWBBVQWSSSPYVEBSPVBBWVAJADEBSVVWVSAJCWB..
   4 ..BDCDWWCBPJYJJRDSEJDCWWYCWBBJJDJVASBWAZSYDEVAJFBWYEBREVYDDBVBJBSJ$SSSDDCPESJ VBBWEVJCZCVJEQARBYBCVBCYVJASBWVJESWDVWWVCBYVWCWSAWWJBPJVVJPS..
   5 ..CEVCYIDYJPDVFDBDWQWSYSEDEVPPBPSZPWJDSAYDJPZPVJVBCJSWBPSWBZJSSDDPUCJRSWWABDD  LWADJPWDYBEPVCVPDJSAVBCJWWVWYBWPYJCBVSJESACBBEACVBYACCPAACR..
   6 ..JVWJBCQDCQSPEVJEBCELJBDJLWSVP WBVJWLPSBPVBBVPLWU RLEVDPEWCSWVJLWYPEDYBPYWC   WCZUCDSVJWBBPSSWAPACDJPWZCAECCAABSSWAEEWCBWSSYPJFPCYJYSWESW..
   7 ..PWEEDDSJQWCWPWCPSYJVBJWWCEZBV DEWPZSDU WWCEWWSSS PVSFCBJP YR  VZSLZJW DSVW    FCWLLVWSPVZWWYCBWPESPAPCBBPADCJRQWYSJAPJWPCWSRLRCBJABWSWEZ..
   8 ..  PPLRPRSVBDDCPBVBBWCVBBBYC$D PSZDVJJJ ZCDSZDCCP SYCP CBV     B  VRPC BBJJ    RECBWPEW WVDDALCSWJWEVYSSCVEJSVWVPCCYRV CSEEVB BAWCWRYCPAV..
   9 ..  YRSEBCVCVYCLYFCSCY  JVRJE   CJ$EDCWC CYVJ$YEJ  YWY  VCY     C  WYVR LZRL     FPDFBRF  SCBCSSEQPYIWAYEELPPBBCCQDYAC  DEYPBC EYSVBW$PSVJ..
  10 ..  ZW BVE SZ BSLYLVV   VYSPQ    DBBPPYV JP DB W     L  EV      F  BDWX ER B     UYSSD E  J VWVJARWELZEEPYR ZUSPDYE LW  PYD$PL L $D P R  P..
  11 ..   Y SYZ  J LJSLRW    C JRV    PJ$YRQY  S PJ                     EW    W       L$E I    R YPYPCYYUYEDPULY WVLEPEP DL  Q PDCW   E$ Q E   ..
  12 ..     VZP  W V URF       P W    WKRCQVD    WK                     JC            PZY O    Y  Z     C F  Y   EERJ JQ RF  Z  YFY   LS       ..
  13 ..       W    W VVP       Y       C  VL      C                     ZF            D   Z    W          L      QF F RZ  D      L    ZU       ..
  14 ..               JY               U  Z       U                                   I        L          R       L       U            P       ..


 >sp|Q16143|SYUB_HUMAN Beta-synuclein OS=Homo sapiens OX=9606 GN=SNCB PE=1 SV=1
MDVFMKGLSMAKEGVVAAAEKTKQGVTEAAEKTKEGVLYVGSKTREGVVQGVASVAEKTK
EQASHLGGAVFSGAGNIAAATGLVKREEFPTDLKPEEVAQEAAEEPLIEPLMEPEGESYE
DPPQEEYQEYEPEA

PFVM    
     00000000000000000000000000000000000000000000000000000000000000000000000000000000000000000000000000011111111111111111111111111111111111
     00000000011111111112222222222333333333344444444445555555555666666666677777777778888888888999999999900000000001111111111222222222233333
     12345678901234567890123456789012345678901234567890123456789012345678901234567890123456789012345678901234567890123456789012345678901234
     MDVFMKGLSMAKEGVVAAAEKTKQGVTEAAEKTKEGVLYVGSKTREGVVQGVASVAEKTKEQASHLGGAVFSGAGNIAAATGLVKREEFPTDLKPEEVAQEAAEEPLIEPLMEPEGESYEDPPQEEYQEYEPEA
Predicted Results:
   1 ..AAAAAZAAAAACRAAAAAAJAQPBWAAAAAACQARBEEAAAQYAVEJEEAAAAADAAYAVADDCSAAEWEASPSZAAAVAAAAYBDDSBJJWYAAAAAAAAJWPABAAVPASBPBBZDCCYCWAAAAEJY..
   2 ..VJDVCCDBPZQAAEWWDDRAVASSAJPDDRJYYYAEBBVVJAS$JAEBPCEEEJRRJAD  AADCPSBEASBCAYBDDPCDCCWA  BACEPZVDVCDDDWSBDSADDAAC WAPWYYW SPYDDW WBC..
   3 ..DCJQJYZS JPSSBECJPSDPCA VDDVPSSADVSARWEBRDZQSBAVSFBSZPASSD$  SRJECDAAUJCJJADLCJDJJDAJ  EVAVBBJ W   JDACARVW YJP VYJSVEF  ASVB   WB..
   4 ..BDCDWJER YYJJRDSEJDCWWR BPJYJDDVASBWAZSYWPJDA DRARWDWDYDDBV  BUYVWFDBYWJSD$VJEQPFPWVP     BJSY P   SVPSSVEP D   UJRC AP  JV W   PS..
   5 ..CEVCY  E CDVFDBDWQWSYSY PW ZQWBZPWJDSAYD  VPY CJWDSLY SWBZ   V $WYRRSBPAWBC EPWJBDBCS     WCV  D   HCCYCB Y     ASV  JJ  W      SR..
   6 ..JVWJB    DSPEVJEBCELJBC C  BCEWBVJWLPSBP  WVC  SCSV D PEWC     SBRVWUDVEQCR BBYVRE BW     P C       JEFWD B     YVW  CS  B       W..
   7 ..PWEED     CWPWCPSYJVBJV D   YJPEWPZSDU W  BYP  W  L   BJP      AQDE    VYVB VZAYS  DV               SBL   C      BC  P   E       Z..
   8 ..  PPL     BDDCPBVBBWCV  Y   BBVSZDVJJJ Z  LBW     J   CBV      W EW    Z PJ WVBB   EC               BLP   J       S  B   S       V..
   9 ..  YRS     VYCLYFCSCY    J   SCYJ$EDCWC C   UD         VCY        SZ          SCS   SD                RE   V          S           J..
  10 ..  ZW      Z BSLYLVV     S   VV DBBPPYV J              EV         JJ          JSE    L                WJ   Q          V           P..
  11 ..   Y      J LJSLRW      E   W  PJ$YRQY                           OB          RE     Y                FR   R          W            ..
  12 ..          W V URF              WKRCQVD                           VC          YU                      UZ                           ..
  13 ..            W VVP               C  VL                            ZL          W                       V                            ..
  14 ..               JY               U  Z                                         L                                                    ..


>sp|O76070|SYUG_HUMAN Gamma-synuclein OS=Homo sapiens OX=9606 GN=SNCG PE=1 SV=2
MDVFKKGFSIAKEGVVGAVEKTKQGVTEAAEKTKEGVMYVGAKTKENVVQSVTSVAEKTK
EQANAVSEAVVSSVNTVATKTVEEAENIAVTSGVVRKEDLRPSAPQQEGEASKEKEEVAE
EAQSGGD

PFVM

     0000000000000000000000000000000000000000000000000000000000000000000000000000000000000000000000000001111111111111111111111111111
     0000000001111111111222222222233333333334444444444555555555566666666667777777777888888888899999999990000000000111111111122222222
     1234567890123456789012345678901234567890123456789012345678901234567890123456789012345678901234567890123456789012345678901234567
     MDVFKKGFSIAKEGVVGAVEKTKQGVTEAAEKTKEGVMYVGAKTKENVVQSVTSVAEKTKEQANAVSEAVVSSVNTVATKTVEEAENIAVTSGVVRKEDLRPSAPQQEGEASKEKEEVAEEAQSGGD
Predicted Results:
   1 ..DAWAAJESDAACCAPAABAJAQPBWAAAAAACQVBEJWSBAJYAVADAJAESYADAAYAEAAAAAAAAEAAEAEDEDEEAAAAAABBEEVJJEAYAJJWSYVSAJYAAAAYAAAAAAAAAAA$..
   2 ..ADQPDPREADQAJCADDAWAVASSAJPDDRJYY W   YJBPADADASSJAYAJRRJADJDJBDDDPEALCVEAEAAAAZDEVPDVEBAUYBABVYABPVAJVDWZPSYSASRSDDVDDDDDW..
   3 ..  D SLAVWCPS$VVWJWPDPCA VDDVPSSAD     EAVAZBBBCEEBVJZPASSD$  YPLCYBBBEYLDBACCDWYWRDDV RAVWWASEDVPAERJ Y CCCWCCDDDDEECYEVPWA..
   4 ..  Y VBL VWYJFUJZ D CWWR BPJYJDDVA      YEDDQES BDWBASDYDDBV   WVVVDDDWSABWBYEJBDPDYEJ DDWAPPBDADDP YE    $  DPPCVBWWWVLSVBC..
   5 ..  B Y S EEDVPJEE   SYSY PW ZQWBZP      C VJRY  W CWED SWBZ    DCPCSWWVDSVDVWYPDJSJ JZ S BQAS WRSL  WV    V  BJVWSEBZECJY VP..
   6 ..  R   D LPSP BSS   LJBC C  BCEWBV      R WSS   Y SDBV PEWC    EJEWV SB J RWDZSPEJB  C    SSC SSCC  CS    Q   EEJCY JB P  JZ..
   7 ..      W  RCW WCB   VBJV D   YJPEW         V      EPP  BJP     JWB E UD P V JV CVCV  B    JCR VBWF  ZW    A    QBE  LJ B  Y ..
   8 ..      B   BD DBV   WCV  Y   BBVSZ         W      PCC  CBV     S J W VJ C   S  R L   Y    EVW  E Y  AB    P     VJ  RP Q    ..
   9 ..          VY $Y    Y    J   SCYJ$                VFZ  VCY       Q   PS D      S Q        CB     Z  FR          ZY  VS V    ..
  10 ..          Z  ED         S   VV DB                DLW  EV               W                 DE         U           P   Y W    ..
  11 ..          J  Q          E   W  PJ                Y I                   B                 PL                     B   D      ..
  12 ..          W                    WK                  R                   U                 YZ                         L      ..
  13 ..                                C                  $                                     B                          R      ..
  14 ..                                U                  D                                                                U      ..
  15 ..                                                   Q                                                                       ..

>sp|P42858|HD_HUMAN Huntingtin OS=Homo sapiens OX=9606 GN=HTT PE=1 SV=2
MATLEKLMKAFESLKSFQQQQQQQQQQQQQQQQQQQQQPPPPPPPPPPPQLPQPPPQAQP
LLPQPQPPPPPPPPPPGPAVAEEPLHRPKKELSATKKDRVNHCLTICENIVAQSVRNSPE
FQKLLGIAMELFLLCSDDAESDVRMVADECLNKVIKALMDSNLPRLQLELYKEIKKNGAP
RSLRAALWRFAELAHLVRPQKCRPYLVNLLPCLTRTSKRPEESVQETLAAAVPKIMASFG
NFANDNEIKVLLKAFIANLKSSSPTIRRTAAGSAVSICQHSRRTQYFYSWLLNVLLGLLV
PVEDEHSTLLILGVLLTLRYLVPLLQQQVKDTSLKGSFGVTRKEMEVSPSAEQLVQVYEL
TLHHTQHQDHNVVTGALELLQQLFRTPPPELLQTLTAVGGIGQLTAAKEESGGRSRSGSI
VELIAGGGSSCSPVLSRKQKGKVLLGEEEALEDDSESRSDVSSSALTASVKDEISGELAA
SSGVSTPGSAGHDIITEQPRSQHTLQADSVDLASCDLTSSATDGDEEDILSHSSSQVSAV
PSDPAMDLNDGTQASSPISDSSQTTTEGPDSAVTPSDSSEIVLDGTDNQYLGLQIGQPQD
EDEEATGILPDEASEAFRNSSMALQQAHLLKNMSHCRQPSDSSVDKFVLRDEATEPGDQE
NKPCRIKGDIGQSTDDDSAPLVHCVRLLSASFLLTGGKNVLVPDRDVRVSVKALALSCVG
AAVALHPESFFSKLYKVPLDTTEYPEEQYVSDILNYIDHGDPQVRGATAILCGTLICSIL
SRSRFHVGDWMGTIRTLTGNTFSLADCIPLLRKTLKDESSVTCKLACTAVRNCVMSLCSS
SYSELGLQLIIDVLTLRNSSYWLVRTELLETLAEIDFRLVSFLEAKAENLHRGAHHYTGL
LKLQERVLNNVVIHLLGDEDPRVRHVAAASLIRLVPKLFYKCDQGQADPVVAVARDQSSV
YLKLLMHETQPPSHFSVSTITRIYRGYNLLPSITDVTMENNLSRVIAAVSHELITSTTRA
LTFGCCEALCLLSTAFPVCIWSLGWHCGVPPLSASDESRKSCTVGMATMILTLLSSAWFP
LDLSAHQDALILAGNLLAASAPKSLRSSWASEEEANPAATKQEEVWPALGDRALVPMVEQ
LFSHLLKVINICAHVLDDVAPGPAIKAALPSLTNPPSLSPIRRKGKEKEPGEQASVPLSP
KKGSEASAASRQSDTSGPVTTSKSSSLGSFYHLPSYLKLHDVLKATHANYKVTLDLQNST
EKFGGFLRSALDVLSQILELATLQDIGKCVEEILGYLKSCFSREPMMATVCVQQLLKTLF
GTNLASQFDGLSSNPSKSQGRAQRLGSSSVRPGLYHYCFMAPYTHFTQALADASLRNMVQ
AEQENDTSGWFDVLQKVSTQLKTNLTSVTKNRADKNAIHNHIRLFEPLVIKALKQYTTTT
CVQLQKQVLDLLAQLVQLRVNYCLLDSDQVFIGFVLKQFEYIEVGQFRESEAIIPNIFFF
LVLLSYERYHSKQIIGIPKIIQLCDGIMASGRKAVTHAIPALQPIVHDLFVLRGTNKADA
GKELETQKEVVVSMLLRLIQYHQVLEMFILVLQQCHKENEDKWKRLSRQIADIILPMLAK
QQMHIDSHEALGVLNTLFEILAPSSLRPVDMLLRSMFVTPNTMASVSTVQLWISGILAIL
RVLISQSTEDIVLSRIQELSFSPYLISCTVINRLRDGDSTSTLEEHSEGKQIKNLPEETF
SRFLLQLVGILLEDIVTKQLKVEMSEQQHTFYCQELGTLLMCLIHIFKSGMFRRITAAAT
RLFRSDGCGGSFYTLDSLNLRARSMITTHPALVLLWCQILLLVNHTDYRWWAEVQQTPKR
HSLSSTKLLSPQMSGEEEDSDLAAKLGMCNREIVRRGALILFCDYVCQNLHDSEHLTWLI
VNHIQDLISLSHEPPVQDFISAVHRNSAASGLFIQAIQSRCENLSTPTMLKKTLQCLEGI
HLSQSGAVLTLYVDRLLCTPFRVLARMVDILACRRVEMLLAANLQSSMAQLPMEELNRIQ
EYLQSSGLAQRHQRLYSLLDRFRLSTMQDSLSPSPPVSSHPLDGDGHVSLETVSPDKDWY
VHLVKSQCWTRSDSALLEGAELVNRIPAEDMNAFMMNSEFNLSLLAPCLSLGMSEISGGQ
KSALFEAAREVTLARVSGTVQQLPAVHHVFQPELPAEPAAYWSKLNDLFGDAALYQSLPT
LARALAQYLVVVSKLPSHLHLPPEKEKDIVKFVVATLEALSWHLIHEQIPLSLDLQAGLD
CCCLALQLPGLWSVVSSTEFVTHACSLIYCVHFILEAVAVQPGEQLLSPERRTNTPKAIS
EEEEEVDPNTQNPKYITAACEMVAEMVESLQSVLALGHKRNSGVPAFLTPLLRNIIISLA
RLPLVNSYTRVPPLVWKLGWSPKPGGDFGTAFPEIPVEFLQEKEVFKEFIYRINTLGWTS
RTQFEETWATLLGVLVTQPLVMEQEESPPEEDTERTQINVLAVQAITSLVLSAMTVPVAG
NPAVSCLEQQPRNKPLKALDTRFGRKLSIIRGIVEQEIQAMVSKRENIATHHLYQAWDPV
PSLSPATTGALISHEKLLLQINPERELGSMSYKLGQVSIHSVWLGNSITPLREEEWDEEE
EEEADAPAPSSPPTSPVNSRKHRAGVDIHSCSQFLLELYSRWILPSSSARRTPAILISEV
VRSLLVVSDLFTERNQFELMYVTLTELRRVHPSEDEILAQYLVPATCKAAAVLGMDKAVA
EPVSRLLESTLRSSHLPSRVGALHGVLYVLECDLLDDTAKQLIPVISDYLLSNLKGIAHC
VNIHSQQHVLVMCATAFYLIENYPLDVGPEFSASIIQMCGVMLSGSEESTPSIIYHCALR
GLERLLLSEQLSRLDAESLVKLSVDRVNVHSPHRAMAALGLMLTCMYTGKEKVSPGRTSD
PNPAAPDSESVIVAMERVSVLFDRIRKGFPCEARVVARILPQFLDDFFPPQDIMNKVIGE
FLSNQQPYPQFMATVVYKVFQTLHSTGQSSMVRDWVMLSLSNFTQRAPVAMATWSLSCFF
VSASTSPWVAAILPHVISRMGKLEQVDVNLFCLVATDFYRHQIEEELDRRAFQSVLEVVA
APGSPYHRLLTCLRNVHKVTTC

PFVM

     00000000000000000000000000000000000000000000000000000000000000000000000000000000000000000000000000000000000000000000000000000000000000000000000000000000000000000000000000000000000000000000000000000000000000000000000000000000000000000000000000000000000000000000000000000000000000000000000000000000000000000000000000000000000000000000000000000000000000000000000000000000000000000000000000000000000000000000000000000000000000000000000000000000000000000000000000000000000000000000000000000000000000000000000000000000000000000000000000000000000000000000000000000000000000000000000000000000000000000000000000000000000000000000000000000000000000000000000000000000000000000000000000000000000000000000000000000000000000000000000000000000000000000000000000000000000000000000000000000000000000000000000000000000000000000000000000000000000000000000000000000000000000000000000000000000000000000000000000000000000000000000000000000000000000000000000000000000000000000000000000000000000000000000000000000000000000011111111111111111111111111111111111111111111111111111111111111111111111111111111111111111111111111111111111111111111111111111111111111111111111111111111111111111111111111111111111111111111111111111111111111111111111111111111111111111111111111111111111111111111111111111111111111111111111111111111111111111111111111111111111111111111111111111111111111111111111111111111111111111111111111111111111111111111111111111111111111111111111111111111111111111111111111111111111111111111111111111111111111111111111111111111111111111111111111111111111111111111111111111111111111111111111111111111111111111111111111111111111111111111111111111111111111111111111111111111111111111111111111111111111111111111111111111111111111111111111111111111111111111111111111111111111111111111111111111111111111111111111111111111111111111111111111111111111111111111111111111111111111111111111111111111111111111111111111111111111111111111111111111111111111111111111111111111111111111111111111111111111111111111111111111111111111112
     00000000000000000000000000000000000000000000000000000000000000000000000000000000000000000000000000011111111111111111111111111111111111111111111111111111111111111111111111111111111111111111111111111112222222222222222222222222222222222222222222222222222222222222222222222222222222222222222222222222222333333333333333333333333333333333333333333333333333333333333333333333333333333333333333333333333333344444444444444444444444444444444444444444444444444444444444444444444444444444444444444444444444444445555555555555555555555555555555555555555555555555555555555555555555555555555555555555555555555555555666666666666666666666666666666666666666666666666666666666666666666666666666666666666666666666666666677777777777777777777777777777777777777777777777777777777777777777777777777777777777777777777777777778888888888888888888888888888888888888888888888888888888888888888888888888888888888888888888888888888999999999999999999999999999999999999999999999999999999999999999999999999999999999999999999999999999900000000000000000000000000000000000000000000000000000000000000000000000000000000000000000000000000001111111111111111111111111111111111111111111111111111111111111111111111111111111111111111111111111111222222222222222222222222222222222222222222222222222222222222222222222222222222222222222222222222222233333333333333333333333333333333333333333333333333333333333333333333333333333333333333333333333333334444444444444444444444444444444444444444444444444444444444444444444444444444444444444444444444444444555555555555555555555555555555555555555555555555555555555555555555555555555555555555555555555555555566666666666666666666666666666666666666666666666666666666666666666666666666666666666666666666666666667777777777777777777777777777777777777777777777777777777777777777777777777777777777777777777777777777888888888888888888888888888888888888888888888888888888888888888888888888888888888888888888888888888899999999999999999999999999999999999999999999999999999999999999999999999999999999999999999999999999990
     00000000011111111112222222222333333333344444444445555555555666666666677777777778888888888999999999900000000001111111111222222222233333333334444444444555555555566666666667777777777888888888899999999990000000000111111111122222222223333333333444444444455555555556666666666777777777788888888889999999999000000000011111111112222222222333333333344444444445555555555666666666677777777778888888888999999999900000000001111111111222222222233333333334444444444555555555566666666667777777777888888888899999999990000000000111111111122222222223333333333444444444455555555556666666666777777777788888888889999999999000000000011111111112222222222333333333344444444445555555555666666666677777777778888888888999999999900000000001111111111222222222233333333334444444444555555555566666666667777777777888888888899999999990000000000111111111122222222223333333333444444444455555555556666666666777777777788888888889999999999000000000011111111112222222222333333333344444444445555555555666666666677777777778888888888999999999900000000001111111111222222222233333333334444444444555555555566666666667777777777888888888899999999990000000000111111111122222222223333333333444444444455555555556666666666777777777788888888889999999999000000000011111111112222222222333333333344444444445555555555666666666677777777778888888888999999999900000000001111111111222222222233333333334444444444555555555566666666667777777777888888888899999999990000000000111111111122222222223333333333444444444455555555556666666666777777777788888888889999999999000000000011111111112222222222333333333344444444445555555555666666666677777777778888888888999999999900000000001111111111222222222233333333334444444444555555555566666666667777777777888888888899999999990000000000111111111122222222223333333333444444444455555555556666666666777777777788888888889999999999000000000011111111112222222222333333333344444444445555555555666666666677777777778888888888999999999900000000001111111111222222222233333333334444444444555555555566666666667777777777888888888899999999990
     12345678901234567890123456789012345678901234567890123456789012345678901234567890123456789012345678901234567890123456789012345678901234567890123456789012345678901234567890123456789012345678901234567890123456789012345678901234567890123456789012345678901234567890123456789012345678901234567890123456789012345678901234567890123456789012345678901234567890123456789012345678901234567890123456789012345678901234567890123456789012345678901234567890123456789012345678901234567890123456789012345678901234567890123456789012345678901234567890123456789012345678901234567890123456789012345678901234567890123456789012345678901234567890123456789012345678901234567890123456789012345678901234567890123456789012345678901234567890123456789012345678901234567890123456789012345678901234567890123456789012345678901234567890123456789012345678901234567890123456789012345678901234567890123456789012345678901234567890123456789012345678901234567890123456789012345678901234567890123456789012345678901234567890123456789012345678901234567890123456789012345678901234567890123456789012345678901234567890123456789012345678901234567890123456789012345678901234567890123456789012345678901234567890123456789012345678901234567890123456789012345678901234567890123456789012345678901234567890123456789012345678901234567890123456789012345678901234567890123456789012345678901234567890123456789012345678901234567890123456789012345678901234567890123456789012345678901234567890123456789012345678901234567890123456789012345678901234567890123456789012345678901234567890123456789012345678901234567890123456789012345678901234567890123456789012345678901234567890123456789012345678901234567890123456789012345678901234567890123456789012345678901234567890123456789012345678901234567890123456789012345678901234567890123456789012345678901234567890123456789012345678901234567890123456789012345678901234567890123456789012345678901234567890123456789012345678901234567890123456789012345678901234567890123456789012345678901234567890123456789012345678901234567890
     MATLEKLMKAFESLKSFQQQQQQQQQQQQQQQQQQQQQPPPPPPPPPPPQLPQPPPQAQPLLPQPQPPPPPPPPPPGPAVAEEPLHRPKKELSATKKDRVNHCLTICENIVAQSVRNSPEFQKLLGIAMELFLLCSDDAESDVRMVADECLNKVIKALMDSNLPRLQLELYKEIKKNGAPRSLRAALWRFAELAHLVRPQKCRPYLVNLLPCLTRTSKRPEESVQETLAAAVPKIMASFGNFANDNEIKVLLKAFIANLKSSSPTIRRTAAGSAVSICQHSRRTQYFYSWLLNVLLGLLVPVEDEHSTLLILGVLLTLRYLVPLLQQQVKDTSLKGSFGVTRKEMEVSPSAEQLVQVYELTLHHTQHQDHNVVTGALELLQQLFRTPPPELLQTLTAVGGIGQLTAAKEESGGRSRSGSIVELIAGGGSSCSPVLSRKQKGKVLLGEEEALEDDSESRSDVSSSALTASVKDEISGELAASSGVSTPGSAGHDIITEQPRSQHTLQADSVDLASCDLTSSATDGDEEDILSHSSSQVSAVPSDPAMDLNDGTQASSPISDSSQTTTEGPDSAVTPSDSSEIVLDGTDNQYLGLQIGQPQDEDEEATGILPDEASEAFRNSSMALQQAHLLKNMSHCRQPSDSSVDKFVLRDEATEPGDQENKPCRIKGDIGQSTDDDSAPLVHCVRLLSASFLLTGGKNVLVPDRDVRVSVKALALSCVGAAVALHPESFFSKLYKVPLDTTEYPEEQYVSDILNYIDHGDPQVRGATAILCGTLICSILSRSRFHVGDWMGTIRTLTGNTFSLADCIPLLRKTLKDESSVTCKLACTAVRNCVMSLCSSSYSELGLQLIIDVLTLRNSSYWLVRTELLETLAEIDFRLVSFLEAKAENLHRGAHHYTGLLKLQERVLNNVVIHLLGDEDPRVRHVAAASLIRLVPKLFYKCDQGQADPVVAVARDQSSVYLKLLMHETQPPSHFSVSTITRIYRGYNLLPSITDVTMENNLSRVIAAVSHELITSTTRALTFGCCEALCLLSTAFPVCIWSLGWHCGVPPLSASDESRKSCTVGMATMILTLLSSAWFPLDLSAHQDALILAGNLLAASAPKSLRSSWASEEEANPAATKQEEVWPALGDRALVPMVEQLFSHLLKVINICAHVLDDVAPGPAIKAALPSLTNPPSLSPIRRKGKEKEPGEQASVPLSPKKGSEASAASRQSDTSGPVTTSKSSSLGSFYHLPSYLKLHDVLKATHANYKVTLDLQNSTEKFGGFLRSALDVLSQILELATLQDIGKCVEEILGYLKSCFSREPMMATVCVQQLLKTLFGTNLASQFDGLSSNPSKSQGRAQRLGSSSVRPGLYHYCFMAPYTHFTQALADASLRNMVQAEQENDTSGWFDVLQKVSTQLKTNLTSVTKNRADKNAIHNHIRLFEPLVIKALKQYTTTTCVQLQKQVLDLLAQLVQLRVNYCLLDSDQVFIGFVLKQFEYIEVGQFRESEAIIPNIFFFLVLLSYERYHSKQIIGIPKIIQLCDGIMASGRKAVTHAIPALQPIVHDLFVLRGTNKADAGKELETQKEVVVSMLLRLIQYHQVLEMFILVLQQCHKENEDKWKRLSRQIADIILPMLAKQQMHIDSHEALGVLNTLFEILAPSSLRPVDMLLRSMFVTPNTMASVSTVQLWISGILAILRVLISQSTEDIVLSRIQELSFSPYLISCTVINRLRDGDSTSTLEEHSEGKQIKNLPEETFSRFLLQLVGILLEDIVTKQLKVEMSEQQHTFYCQELGTLLMCLIHIFKSGMFRRITAAATRLFRSDGCGGSFYTLDSLNLRARSMITTHPALVLLWCQILLLVNHTDYRWWAEVQQTPKRHSLSSTKLLSPQMSGEEEDSDLAAKLGMCNREIVRRGALILFCDYVCQNLHDSEHLTWLIVNHIQDLISLSHEPPVQDFISAVHRNSAASGLFIQAIQSRCENLSTPTMLKKTLQCLEGIHLSQSGAVLTLYVDRLLCTP
Predicted Results:
   1 ..AAAAAAAADAAAAAAAAAAAAAAAAAAAAAAAAAAPBCCCCCCCCCCCPYCWCSAACAVPVCCJWCCCCCCCCCWCAAAVWAADACYAAACVDJYAYSAABAARADAAAAVAADJCCAAAAAQAAAAAAAAAAJYJZADAJAABWYAAEAAAABAAADDAWCAJAAAAAASAAA$SPSWAAAAAAAAYAAAAAJWWYAAAAAABBAAAAJBWQAPYCVAAAAAAAAAAAPJAAEDAWCSVPAVAAEEBAAAAAAAAAAPVJZPAAEAYAAAAAEAAAAPWDAAAWAAAAAAADAABJBDVAAVAASBAEAAABAAEAAAEAAAAADAWSBAJWPYPABEPAAAACCVAZAAAAEBAADJEAAAAPYPYDAVVJDAAAAAAAAACCCYAADAAAAAAAWDAAAAAAAPAVJPSJYYCWAAADDAUPAESVSVAAWVWSJPPEAUVCAAAAAJJYYEYAVASAAPADAAZWAADWYPAAAAAAJEWBAJAYWBADALWCYYYAAABAAAAAVPAAAZAPVSDCJBAAAAAAAYLAAASBPCVBRALDVPQAJBVCVSJABVAWJAYPPSAPAWWYWYBBAJBBYSYQYAAPSBWYEPWJACAVAAJABWSAYVAYAADAAVAAAAAAWAADADDDJPPYPYJAAPCEABEAAAAPEAJADVWWWERWASBAEAAABJCDAAEEAAEDAEEAAABCJYSSEEWCBPAJAEBBAAAAAAEAALAAADJCDAAELAECSWSVPSYPJCDAAARVADAAAJAWSJVADWACVABAJPPEAAAAAAJSAEBVAPAACAJADEVSSBEBYAAWBAAAADADDAYAABAAEAEPAPADAASADAAAAVAAAPBBBADAADAAEAAJYEEBBAAAAAAAAAABBVEBAADAAAAAAAAAYPDPABAAAEAAAAADAADDEBAAPEAVJVAAVDAAAAAAPABCWADEWEJWPBASPCASBEAACAJPEABAADAPVVLSCSAJSEBRAVREPDYPEAPABSAJVAWWAAVAAJAEAAAYPVJAJVWAAEELAAAAAAAAAAAAPWYADJABCAEBYACSSAAAAAJAYYEAEJJAAEAAAADAYEAARJBWPAAAAAEABWAJAEAAAWCWBBAJSAVAZAAPJVAEAAEAJAAAAJCSVJBBABDAADAADAAADEAAAASJPAAWJBWAABAAAPCAQALWBCVBYAEWWSBACJBAJRAWPSWPVJZZSAAAASAWAAWJJCRSBVYZJVAAPAAAPCSYWAASAAAAADEAAAJEBABBBAJVBJAQVCAAAAASADAAAADAJAAAASWCDAWAAAAAAAAAEWPAJVAAAAAAAAAAAADD$EWJCWDAPVAWBQPVAZAQAAAAAAABVBVCAPJEDDQZAASSABAAAAAAAADAAAAAEADADAAVACWADDAAAEEVAYJDVDAWACVBADAJPYEAEAEDAAAJJAABDAAAJDBAVSAAAAAAAEAAAAAAABWDECAABVVAAJEEDWABAAVJBJRAYYVRDJAAADBSJVAAAEADEEYDWAJJDAAPVPCAAAAVWEABEVAYCAAJDEECAAJABAADABAAEWEEPVAYJAAAADAJDAEBBAAAAADAADVJDAAEAAAAAAPAADAAQVEEAAAAAAAAAJEDAAAAASVJBALJVSAQAAJAAWWASAPSYAJWCVAAAAAAEJJWBCJAAAAARSAAEWAWSAAAAADEVJEJAAAEAEAYAACEEEAVAEJBRDJWAJAWY$YYAEBPAAJWQAARAAAJCYPAAAAAAAAAAAAABAASAWAAABBEWCYAAEEAEYVAAAAADAAAAAAWAJVAJAESCAAAAAAJEWCSDZJCPDBWAAJBDABAAAAWAWVABABAAAABAABBYJJEYAAAAAAAPPCAABJAEYJBAAPVAJAJCPJAPAAAAAPYAVAJAAAFAAAAAELAAAACDJAABVAAAAAEAADJAWADAREBAJCSVPADAAADWYAVAAJAEBEAADEARSVAPCPYAAADAAAAAWDABEVAACEEEAADBWAASAC..
   2 ..DWDDDDDDADDDVDDDDDDDDDDDDDDDDDDDDDJBVBSSSSSSSYJVCCWJ CWCWCAWCSS JBSSSSSSZYFYBDDWB V B CVDSBZAZPDAJ  J  S AJJEEAJPAVPYSDDDSPCEBVDDBDDWPVAYJJJAVDAVA QADDVDADD   CPACEDDBBCQCBDWQWBDASEDDBD EADDDJWAAB      BJEBV YAEJAECASYJJDDDJDDDESLCD S EJYAAAYJVDAAAEWDDDEBDJBYWPYA BAFASSJDJA  DBSYSD  A  B SDDADDABLRBDPJJJAABBVDDEBWBDEVADDJDQAEAJWBEDAZEEPAADDBEFVSJVWJSWBJDDAEAC       Q DEAABDDDDBDDDBWSCVDADWJDBUCAADDSWYEPDDPDYYAZWVRDBDEADVCSJYRCJVJJA  AEJRDASADDPPCYAVBAZJ VEBCCEJVRVAJSEUAAJDEEJVBCPYYBBWCASABC S  VEDBWWV JCADJJWVVYRYVQYPVSDDDWJVPVDEECAWYJF W JYP AACVJWBWJBQYVPPAAZJBBCBJPJ ADEDWAPPP VDWZEEA  V JASAYDVSECWJVADVCDWBWRDDDD DADJAP   P  CAJVCBSEABSPS$JVCY$ PJW  PW APPAEPJYJPAVASDDBBEAAVAAEDEAWCPVABCCBYAVAEWEADDDCLEAP ASDDASSACCJPEAEABESSRAABVJEEDYECADDBAWAJPYDAVYBSBAESVAA V  DBDACS YSJ  A RDCVJAJSBRV DPWWDBDAJZBVPYEAEER VA ADADDA   EDPEPDDJEAEBABBABBBCYP ABAAWVDDDDDDDRAEAADRDADWDDESQ YPV  E VDWSDBDDDADDAEBEJDUACA YC EADDDDDDABDWSDSA Y  YAJVCBDBDADJPCAAAEAEB  V  VCSACBRDEADWJ JYAJBEWVAYDA BVAVDASJAEAJD DJBAJBLLDCADQ    DDEDEDDLW     BAD JW CWCAEEJYVDV  B  EAE AEESJADCAD AYJVAC JDDDBAAPAVADYBPSVJADA SF YDDABWEVCSDVD  VCAAAAARASA DDABVYDDEAW B BDYAVJQCYCCYEDDQJDJZVJ YAWWJD BVVARPCYVASJBBWCCSWYWCVCDJAJJY YAAPSJJWJAVQJZCJV APAVADBBWSDDBERD  PBEBWEADWBVPVACADSWDDADADDPEADADDBWAVA EY DEQYED DAVWJ  D P JD DJDDDAAAPLABAAY AYAADJWSVERSCDDDJVAAJJABWAB     DWA S BDDDDWVAD DD  WAYYPWJWAFDAADEDA YDJSAJBWEWDYJE WAAB E  BADRPVAEBAADDSS EC JD JJEDCAVDPJJBJABWD V SWWVDAPAULDE DAAAABDZE EAYJYJAWCSJ  BADABBAWBJ  EB WEASY V AAA JAAQSWRDAWBAWYCSWDE ADDRVAEA CAVAAYPDDAWAPZWEEBDVQBADDA AADWAE DBV A DAQSPAAA BBVVSDVVDSADDWDP    BA YADPDBADPAADASBCVDWCY EVEDV APPLE AJ JDEER  AA AAEDDBDEWABJ JVYDEAPAJDBACVSABB WSADLDADASCS PAWYSDA PJWSJDBBWCADE DEBBDD$PDBEBJJDAJDSAEBV    AJ BADDQDDDAE   D DV A  DBAWDJDVVEALYDWJCLSSEEZDZBAVYS BSDBDCABDBD    DDBAAVVBAV    DDJVC D  AVJVVJDYJZJ $PYJBJADDDDDB S  APDDCSCSVEAV    S  D S VD   AEBA VL AEBAEP VAAA ACVDAARSADEPSRAD DAYDARYWDBW V  ADD DDAAJWJ EWJACBBBEEAWDAEW..
   3 ..VDV VWVYJBEJDJYQ SSSSSSSSSSSSSSSSSPECJWWWWWWWZSBJRYY   SJVYJJWV YJWWWWWWW  SVJJCC D D VYCBVYWSVYZP  A  A   PDDJWJQ WVD   BUSDSW BDL JAAP$CPYD EWA   B BDBW Y   QVDSCBBEDDDDDBDJYJCYJJVP E  ZJRBPR JE      WAASD B DBJ AWW  V  WSQBPDBWA  A RAW BJQY SPBDBDE JD BDVVB SS DRCZYCSEEB   E  JV  D  D  EBEECJAWCAR AWSVEEABBRJDDAVDDDVSYED DVBCJWADCQLSPY VJSWWCDYDDDDAA EBABW       A EASEDES QDW WFXWSCBWSEYEZQDCZCJCCEDYACDYCCPEPYFYDVAPPJYJLR WCDSVB  EWAAEBWYVVDDPPBBAC B PUCPADEWESVRVAYPJDWWYPPRFBVJV PB DPWJ Y     EADY FSDA VSBCBJRBAZYDBEVJSWAYECJBPWSAPB   B$W ZYAYPBSPPCJB WVEWCYJWALZAS JVAAEPJS   J EAVB  S WDJPJQEDPPYYA WJVW S E J P JCCBQD       WSADSASLDEVDDDYJVC CP   EL VS VCZVW WVBJCV A  REDDPCLBDESWPJARBSVVDPBBASEJPDB R  PDBEBCVV VA CDAPPCYJAJY W   WDWWEE D CPY Z  DPAAEEDPA B E  EDAP R WJA    PWAYBWAZ SA VAJ BJWYDAACVJCES C BJ  Q       SV JY CQYSWJEEDBEEALVY DV  JWBJJVJYJEJSEBADBYVCSSDB  DAW  S  YPACEVSWRJ SBRDSBVBJJ WB W YEJYVEEEVPAEAD      VJBABEEDJZSPE DDECD      YBWWEEAWEEAE C  WSYJCCABS JY QSBDDBJBBS VASVVY PWDBBA      BCBRECV     D       ZVEDSPWDAY  A  WW  DLDCDBEJW   SEBBD YJWADEECWDDEWPADYSSJB D  VYJYWYDAJJAPB    YWBYDSWVD   JDBSVJDBA    AA BDDWWPBSYCEWAASAJE CDPVBV  EAJDVWV CYPVECB YPPAVDDPYJDAD DVBYCBVBAVYWDY B   JVD LERJBVJEAA   BWAEAADQBWPADJDJERDE BEBVCCPBVWWCDDDA  PA SDPDDB   AAP      R   DVJCJYQCYSADYP ECJPAS JBSCCY EBPBJQSWWVCBA      R  W EVEYPDJC  J   DJZA PBP RE EYDEB WJAAV JVSYAWPP JYJS    J  BVASDEWJQJCC AY  V BDD JBPWDDRDBJAAA Y AAAEPBSBAA L B    EBVS PV PEDJAVAA  DBBBAAZJPD  AE VJWAV B     ADBYBBZR AD AVDBCAV V  EDJSS DYDDQDJBJBVDA AADDECBDB  J B      BEE       BC   SPCDDWPDWA C Z      R   CJAWVBWJDESDPCRASBAV DYDWD  AB W SV CRLAA     JCRBEEJBAEAV DWJABBSBSWABJWB SA  D AA BPQBJV EPJACSW DPJFEVJYYVJJV JSDDWBWDZDVDDBRCDJY ASA     A ASA PYZE     E  W P  VARAVSCD RPSZ CZUPRVLWYCYYJECA DW D PYEEEA    AEDEWA  B      B B  W  BWAJYACJW    YSACVVVVEQJA     CSVWVSBDDJW    A  V J B      D   A  WARDB  CY   ECJJ ZDJYDQEAEB E A  AWSAAB Y   CC      AP  VPYASEDBDEVB BA..
   4 ..BPW CB   JWQPY   WWWWWWWWWWWWWWWWWWWYABBBBBBBW SF B    WPWWCYZ   ABBBBBBJ  JYPCSS     ZEWWAACYAEDA         ECS ESS  ZB   CAV  E WES D  WPSAV  BD      EBWD     W VDAVEDEPPAEWCWCCYDPVWJ     VSWWE PP      LDJDB Z AAD  VJ  C   C JSLEJW  D DPA   D   JLEWEB  R SBRBA V   BPPJJVCQ    S         E  UWVQYSSESWB BY DJDDDCJDEBW   WEVDBP J WVWBPEBLIR    V BYYWCPB     WEVW          QUBRSBW VW   J YVYJVBCSVEV$JBVCDDDCZESQSDBSVJAPQE BVEAADPB L SPBD  VRWBBQPZJYWYDQYCQB S DPWVJJWEDAQDB SVSVVDVVJPJJWSA VS RSCE W      D   RWBW  C DCSZZY$AEWYW ECJRBWBWAJYWCS    VC DPDDEEWDV Y  JAJ JDVCJJBDW Y SWJDAR     BDDW  C BVDZ EPJ VCCD YA   V J Y   P  S         BWSVVCAJWDJBYC B   JA    B EW WJCY  ACJPB     BBWRDDBDEPASYEP EWJDYSCL LJBQBD V  EEE WB Y DW DPBB BBCVZW B    EAJYB E  V     PDDLD  DY   L  JW     J      SJBDUYBV WD  LA WPEPEYWDA BJJ A A            Y AZ JASJADW E WWW DZ J   VPJEE EZPB  PWREEZWBJEJV   Q      ZDDBWSBEBE JPD  SJWDD BJ B CSSWEBBSELZ JB      YASVEWAJRCA P RBRD       WW DLBRAYBD     BLLASDVVB  E  BPVWDDWER ADABPE ACF AW      DEDSYJB     V       AYBCZQJ         Y   WBDECVAL   AAED  P  SEDUVREVWESCWJVDBS E  AE D BPFQRWDA    VDCBEAPRE   W DJEWBEB     B DPBSSAE VWSBLVDJWP VWJJ B  JYYVW E VVS YBF B ASPCJCPW     BVLEY EW BEWQ     W S V W DBWDJD   ASRDVDV PAES PJPYEBJ DVPWBDDEEECEYBJY  VD JBWVWJ   D D          S  S WPJABEL   $VYSB   QPWYD  SDYCBP SYEW       A  A  WJBEJPW  S     CS J V  R  JCCZ CB CB ADJRB  S V CA    S  CEPCJDDB   P WL        DWDBEEDSDEEBB    DYP DVS   R      AEW  S  DDE  RV   EDVEWJ SJ   SD  SSRB D     S D AABJ JA  CYA       DJBAL J JPCSDSPEB V BDAE  DEE             B       WS    DWYJBJYAC          V   V JVSW BEBCE JEDYEBW    B      C    SJDDB     DDB PREABDDA YPWBDWDDD DJRP  D   E  B Y YW Z SDAVJW   Y ZW SPSZ VD  WECLEEEBSAYEYB PEP  RB           JVYB        Y     SDDYEWW  EVP  SYYPAADDWVPVBED J    BCDSD     JV DQB                 EYPPSSRPY    ZVCDBJYJCECQ     S WDWWDBB F           R      E   D  BSBAY  J    DW S AWWLVVD$ A B     AEJJC     VW       B  RQBBWSEAWBYY  S..
   5 ..JBS  J    PVZE   PPPPPPPPPPPPPPPPPB  VVVVVVVVB WR E    J B       VVVVVVVP  ADV  Y     SDBEDP  JP           SPB BBV   C   DYY     Y     ZQDVD  SL      SJEL     J PJ WWJ V  S ZPVW CCYCS     RY BD  A        WRW D W E      S   D EJQWAB     E    W   BWWDBW    VPSJJ Q   SJWCPZS                  B BBZPEVBYW SZ ZSSVWEBAWC    BBYPWW B  EZCCBSVBE      S ZB R                    BBPBJ B W      JRSW  DBWQZVEJ PJSZQCCEUCZFBADSYB  VWWQSZWC   BDSE  BYBSWWYDSJY ZDZJWV E YVDJDSASVYCSY VCDEYBJYBDB SWC AP B EW        U   YBY     JAA  JCDWVSZ V BEWSPAVRJBAC    CY RSBSAL JW P  DWL VPAJEAVSA V BEDQY      ARB   Y  YWJ LBL LBB  CZ   W S E                YAY JEJR RBWJ  S   SC    C JY ESY   JYABV     SSJWJ E JQYCLRJ PV  SERY WBEEW     JCY E     B  YPV  CB DS A    ACBCS V        $EWCW  BC   B  BE     Z      EEJZAQWY AC  VC PWFSWWQJZ SLB   R            P P   DAPC B R DDD S$     BDVBW  CVD   YSWLE EPCPQ   B      PRLJD VBJP YW   JQCBC  W A S BEBCD ASV B        BJSJAJVCY    BJ P       J  P J YDVB     VCVLDEBWV  A  Y ZSRRJDV W DD S YV  Q       JW CBDJ             BDCJWYV         B   B BBJYVB   DDPV     B WBDSBWCSVB APVW     WV S D JSZBY     PSZJFJEEY      EJ RJV     Z SBJ PBJ A WDWBC DS APBC    SPZE  C  WY ARL W CDYYPYVB     PWVVW JE APSB       C       BVW     D PLPJ CJWB YPWLBES PYCELYJS DEJ  V   J  CWD BR     S          C  W  JA DVE   DPBWV   SDVJP  Q JWJR PS Z       C  E   SVSQDV        DV Y       SBBD B   W  BCSJ  D P          EBSBRWLW   A  E        WCJE BEEWDLSP    JSD S     W      SSE  J  VSP  AD      WJY  C       BCBD           JJYP EV  D I         UJB   SJDWYCWVJ   CR    E W             W       A     JB WV  BB              Y SEDV DSRJV WYBJPPS    V      Z    DVJSE     CRW JDBL   Y CYBWVJJZ  LRBL      W  J D VD D  BEW E   S DB  WAA WW  DWRBRVB VWCYAJ  RB  DS           DPRR        C     WBBPVJY  DA   E  ABRPAPDWEWDJ      J WAR                            DBYRA SVC     ABVSYSBJJSS     W EJDJJE  A           S             YE C   D     L B PBBVSAVB         CPWS      JP       R  CYCSAJ WJDDJ  E..
   6 ..EJC        RYC   JJJJJJJJJJJJJJJJJC   PPPPPPPF FV L    B S        PPPPPPS  BEY  F      SPCJS  DS           D   DD    J   WEE     J     DVWEE   E      WPV      S  B EPP Y  V YBF  B RJW         S  S         E      L          F WESYVD              WDJPJI    EVWAY C   JSDWDEB                  J  PBER F        J QJES E    CCRQS     FRAEY$YSJ      L WP                      ADDJP V Y      BBD   BD DDFPW  EE BJYJAQSRD VHAJ  JBYPZPZW   ERCW  PJDDRCC ZEB  AD EJ W JCVYWLSB BR J CSWS PBBCWA ABS CY E           V   BF      WEB  P WZCR  J  FCQ  WCLZ E    SA BZZWBY CC D    Q YWDVBP  P W CV VV      D S   B   CY WSY ALS       E   Q                CP  WJDD VAC   W   VV    R LC   A   V PCD     DWBBS   LY  CBW L    BS  VIPJS     BBW C         LY  W  VV F     YD Q           VLE   W$   P  RC            AVVAWCY  CJ   E LCJ PESWD V P                  Z    DEV S V S Y P      EBESB  RWJ   REVJW DY RD   J       SVEV YCE  VR   WS$ZB        YQ  J  JC          CWWSVSBB        B       A  B C S ZC       WC S  E     W  BCBDS    EU L CY  J          JLVC             S DWB B         R   D RVPSWV   VSA      P CPYDJSSVCJ BAEE     J  B A  Y V$     U$DP ECC       A  W D     E YSP CJD R CSCW  EW SYAR    $WDS  J     JJE C SY ZSVDP     YE YC  P ECVP       B       PW        CEJ  ACJV CYDRUPC ESWLWVQ  PJW  Y      BRE  E                P  J  UY  Y    SJDCJ      LS    WVYA            V      RWYSBE           B        VWP A   L   AEL    B          S DEW  E      J        PSWQ  S   Q J     CJ       A      D P  Y   VB  BY       VW  S       PYWS           PPEW  Y  W V          Q    YZVBVE SC   JW    P               Y       E        P   SW                WSRY S J B EBWVC B    J           EYBYD     SES   RR   B Z PJWR    S VR      Z  R V DA P  S D B   C EC  SVP     R EEW C EJQSCE  SV  AJ            CW               RWPSC J       C  DQW S CASALP      S  JS                            PDBBP JCS     JWWWZBEWSBC     E SLQYFS                            DW J         D E SJYPPWYP          JV                W  LAVJCW L  J   B..
   7 ..LVY        SBQ   EEEEEEEEEEEEEEEEEE   AAAAAAAV JY S      Y        AAAAAA   VJ   L      B DW   SZ           V   VQ    V   EWJ              WS          PSJ         E JS  S  Y B      BSE            Y         W      S             CWC R               J R      JCJSC A    DEBWBJ                  S  RWW  W        V  PWR J     SBVV     YDPVZAARW      E AS                      SJECW Y Z       WW   JE PWPVC  R  SD PWAVVY BIJR  SCVBJC A   P A   YASJVPA ECS  VS SW V WRYDERBJ EE P BBZC JDCSAP RDY SA F           E   SL      QSY  Z J D      JJY  E V  V       Y  J   BS Z          LS    C    $             J   PS BWP S W       J   V                D   Y  W AWJ       Y       BB   R     WWW      J P     J  JY       V   DL BJ     WRL V         W       J S                    BJB   AW   S  V             B  C B   EZ   P CDP  JVYJ   V                  S    PWL       R B      YLZ S  P S    WBWC  B SJ           YJF  ES   PL    DPPY        PV  S  AB          D D P  S        J       B  E L             B    W        PE CC     Y       L          PV Y             F WBV P         D     WYSB     B J      R  SSESJPDJS PCJS     P  C C  P  J      VJ  VSY       B            PEC VVP D   BY      JSP    BJ B  Z     ZAS R V    JWD     EP  E    S$CD       A                 JDS  SPY  Z SSCLL WBJY  B  CR          EQJ  S                   V  C   D     ZPEP      WW    DED                      C SB                    S W E       B                   C Y  L      W         DBJ  V   R W     JY              V         S  W         C  D       R                SE     Z Y          B     CWEWR PD   PJ                            V        R    J                 LES V S R SV BA E    Y            EP P     BW         U B SPF     W YA         E   PC Q  V L        D  CZR     V   S Y YY  DS   J   Y            WP               JJY D R          YYS J  C  SY                                        RSA  BW      BYEYBR  RWD     B Y YEPR                            VJ             Y  ZCZCDC           VS                S  E JDDC        L..
   8 ..CC         WE    VVVVVVVVVVVVVVVVV    JJJJJJJ  P  J               JJJJJJ   P    P      C LS    J           W             JB                            WL         R Y   W  Q E        C            C                P             QCV S               R         W ZE R     JQBCV                     V D  E           V W       W  C      Y Y PRY       R BC                       WYYV            B   SV YBQLS  Y   V WJV ZC AUCS   EBWDB     J Y   SLCLJDD PW   CV VS   CWJBSBPL J    PDBW SCW$   CPP  J                  D      BJC  $ S Y      SS                   P   V             PV    P    R                 EC CIB Y         F                    Q   D    LDR               DD   Q     YVP      C C     U   W       D    R RE     CJV           J       Z                       PR                            J D          E B  SY     Y                  Y    R D         R      D S L           SR     C           CPQ  CJ    J    AJY         EC  V  EF          Z E    V                F                  S              L L      E       P                           J  LY S         P     ECZJ     E Y          W  RQBW   EWC     C  E P            BY    D                     VE E R W   E$      SEA    UB    P     SPV   J    EBE     RY       WJBJ                         SCV   SC  S  VD    CS                  W    W                   Y  E   F      CJY      ZV     SP                      J WQ                    F   J       D                   W    R                JSP  B           Z               W            D         L                           VY                  Y     S  BV  Y   SS                            J             F                 Y       W VW Z  J    C            PC J     EV           Q VR         C         S   JP W  W J        S  A J         L Q CC  E    D   D             C               LE    B              P  E  UE                                        SWC  PS      WDYCQ   BEE     D    F                               D                  CWYJ           CE                   D LWFF        R..
   9 ..PY         YQ    CCCCCCCCCCCCCCCCC    EEEEEEE  Y  P               EEEEEE   W    E      W P     V                          D                            E            S   J  W V                                      R             RPJ                             WP P     R VYW                     U    J                     J  J      E B RZC         PQ                       YCFY            F       YJRV  B     YYZ JV SWDZ   JUFEI     Q L   WSRW SB R    W  CP   QJPW CVP R     JEY YWQW   JEZ                     E       VE      E      W                    R   S             FC    Z    S                 VD JAE                                    P     QV               S    J      YR      V S     V                S WR     FWS           R       B                       RD                              P               CC     C                                          C   P                              JRS   R    A     V          VJ  C   R            Y                                                       W                R                              PD                 FIEW       D              BQY   Q V     D  J Z            YP    F                        A S C    F       CS    YD    S     V     W     S      WU       CLPV                          F     $  W  ZJ     Y                                                  W      S         Z     YE                      W  S                        L       L                                         P Y              D                            F         P                           C                   V     $  C   Z   DI                                                            $         AF C       Q            CW       LY           S DC         S             CQ    J C        V  V W           W  L  F    W   P             J                V    P              R     VW                                        ZCD  EB      PE EE    RY     R                                                        B$Z           L                    S SRRV         ..
  10 ..WE          W    YYYYYYYYYYYYYYYYY    FFFFFFF  A  Q               FFFFFF   Z    J                                                                      L                Q    J                                                      P                              S W     S YD                      W    V                        Y      P   DJD         J                          PE            J        LY             PW   EE    SLVV       D   UVEC JE Y       DD    ASL PCY W     WY   ZDY   EVF                     Y       WF      P      Z                        E              R    D    W                 B  SCW                                    R     LY               Y    S       J        J     A                  L      SY                    Q                        W                              V               FE     Q                                          S   V                                         S     Y          RP      Y                                                                                     W                              SR                 LSV        S               JD   W L     E  L              Z     J                        R   S    L       L           W     C           Z       C       FFZW                          S        D   W     E                                                         V               PZ                         Y                                R                                                          P                                      S                           D                   P     B  Z       LL                                                                       L P       $            B        PB           W  L         B             ZV      E                               W        C                                   C              B      F                                         VE  W        L I     V                                                               JSW           Q                    Y UZVD         ..
  11 ..YS               BBBBBBBBBBBBBBBBB    YYYYYYY  E  R               YYYYYY        R                                                                      F                Z    P                                                      R                                B       RP                      S                                    V   VD          L                          Q             E         D              E   VP    Q BW       E   Q YV YJ         JY    BZZ VL         C     E   F W                             RD                                                          S    E                 L  VU                                           R                             Y        V     D                  S      Q                     $                                                       Z               VJ     W                                                                                              S          W                                                                                                                             P                   W                        PZ   D P        V                    B                            F    P       Q           R     L           C       R       PZ $                          R        T         V                                                         E               DC                         Z                                                                                           B                                                                                      C        R        P                                                                       P E       S            Q        YJ                                      SJ      F                                                                            S              C      I                                         DS  V        S                                                                       RE            Y                      WPJL         ..
  12 ..                 QQQQQQQQQQQQQQQQQ    LLLLLLL                     LLLLLL        Z                                                                      R                                                                            D                                        ZW                                                               W           Q                          S             P                        Z         C WE             F  EQ         PL    D   F          L     L                                   ZP                                                               J                 Y                                                                                            L                         Y                     C                                                                       B      Z                                                                                              R          Z                                                                                                                             J                                                 Y          W                                                 L    S                   F     W           E       S                                     Y                                                                            R               LL                                                                                                                                                                                                            Z        S                                                                                U W                    W        VL                                      BZ      R                                                                                           E      L                                         F   Y                                                                                FZ                                     L          ..
  13 ..                 FFFFFFFFFFFFFFFFF    IIIIIII                     IIIIII                                                                                                                                                            L                                        E                                                                                                                                              L           HY             L             RR    Y   U          R     D                                    W                                                               C                                                                                                              S                                               D                                                                       R                                                                                                     Q          C                                                                                                                                                                                                                                                 Z                                     R                                                                                                                          Z               FW                                                                                                                                                                                                                                                                                                      Z                      Z                                                        Z                                                                                           V                                                L   Z                                                                                                                                  ..
  14 ..                 ZZZZZZZZZZZZZZZZZ                                                                                                                                                                                                                                           L                                                                                                                                              Q           IL                            U                         Q                                                                                                                                                                                                                   Z                                                                                                                                                                                                                                        I                                                                                                                                                                                                                                                                                                                                                                                                                                                                                                                                                                                                                                                                                                                                                                                                                                                                                                                                                                       R                                                                                                                                      ..
  15 ..                 LLLLLLLLLLLLLLLLL                                                                                                                                                                                                                                           I                                                                                                                                              R           L                                                       R                                                                                                                                                                                                                                                                                                                                                                                                                                                            U                                                                                                                                                                                                                                                                                                                                                                                                                                                                                                                                                                                                                                                                                                                                                                                                                                                                                                                                                                                                                                                                                                              ..
  16 ..                                                                                                                                                                                                                                                                                                                                                                                                                            U           Q                                                       Z                                                                                                                                                                                                                                                                                                                                                                                                                                                                                                                                                                                                                                                                                                                                                                                                                                                                                                                                                                                                                                                                                                                                                                                                                                                                                                                                                                                                                                                                                                                                                                           ..
  17 ..                                                                                                                                                                                                                                                                                                                                                                                                                                        R                                                                                                                                                                                                                                                                                                                                                                                                                                                                                                                                                                                                                                                                                                                                                                                                                                                                                                                                                                                                                                                                                                                                                                                                                                                                                                                                                                                                                                                                                                                                                                                                                                   ..


>sp|P54259|ATN1_HUMAN Atrophin-1 OS=Homo sapiens OX=9606 GN=ATN1 PE=1 SV=3
MKTRQNKDSMSMRSGRKKEAPGPREELRSRGRASPGGVSTSSSDGKAEKSRQTAKKARVE
EASTPKVNKQGRSEEISESESEETNAPKKTKTEQELPRPQSPSDLDSLDGRSLNDDGSSD
PRDIDQDNRSTSPSIYSPGSVENDSDSSSGLSQGPARPYHPPPLFPPSPQPPDSTPRQPE
ASFEPHPSVTPTGYHAPMEPPTSRMFQAPPGAPPPHPQLYPGGTGGVLSGPPMGPKGGGA
ASSVGGPNGGKQHPPPTTPISVSSSGASGAPPTKPPTTPVGGGNLPSAPPPANFPHVTPN
LPPPPALRPLNNASASPPGLGAQPLPGHLPSPHAMGQGMGGLPPGPEKGPTLAPSPHSLP
PASSSAPAPPMRFPYSSSSSSSAAASSSSSSSSSSASPFPASQALPSYPHSFPPPTSLSV
SNQPPKYTQPSLPSQAVWSQGPPPPPPYGRLLANSNAHPGPFPPSTGAQSTAHPPVSTHH
HHHQQQQQQQQQQQQQQQQQQQHHGNSGPPPPGAFPHPLEGGSSHHAHPYAMSPSLGSLR
PYPPGPAHLPPPHSQVSYSQAGPNGPPVSSSSNSSSSTSQGSYPCSHPSPSQGPQGAPYP
FPPVPTVTTSSATLSTVIATVASSPAGYKTASPPGPPPYGKRAPSPGAYKTATPPGYKPG
SPPSFRTGTPPGYRGTSPPAGPGTFKPGSPTVGPGPLPPAGPSGLPSLPPPPAAPASGPP
LSATQIKQEPAEEYETPESPVPPARSPSPPPKVVDVPSHASQSARFNKHLDRGFNSCARS
DLYFVPLEGSKLAKKRADLVEKVRREAEQRAREEKEREREREREKEREREKERELERSVK
LAQEGRAPVECPSLGPVPHRPPFEPGSAVATVPPYLGPDTPALRTLSEYARPHVMSPGNR
NHPFYVPLGAVDPGLLGYNVPALYSSDPAAREREREARERDLRDRLKPGFEVKPSELEPL
HGVPGPGLDPFPRHGGLALQPGPPGLHPFPFHPSLGPLERERLALAAGPALRPDMSYAER
LAAERQHAERVAALGNDPLARLQMLNVTPHHHQHSHIHSHLHLHQQDAIHAASASVHPLI
DPLASGSHLTRIPYPAGTLPNPLLPHPLHENEVLRHQLFAAPYRDLPASLSAPMSAAHQL
QAMHAQSAELQRLALEQQQWLHAHHPLHSVPLPAQEDYYSHLKKESDKPL


PFVM
     00000000000000000000000000000000000000000000000000000000000000000000000000000000000000000000000000000000000000000000000000000000000000000000000000000000000000000000000000000000000000000000000000000000000000000000000000000000000000000000000000000000000000000000000000000000000000000000000000000000000000000000000000000000000000000000000000000000000000000000000000000000000000000000000000000000000000000000000000000000000000000000000000000000000000000000000000000000000000000000000000000000000000000000000000000000000000000000000000000000000000000000000000000000000000000000000000000000000000000000000000000000000000000000000000000000000000000000000000000000000000000000000000000000000000000000000000000000000000000000000000000000000000000000000000000000000000000000000000000000000000000000000000000000000000000000000000000000000000000000000000000000000000000000000000000000000000000000000000000000000000000000000000000000000000000000000000000000000000000000000000000000000000000000000000000000000000011111111111111111111111111111111111111111111111111111111111111111111111111111111111111111111111111111111111111111111111111111111111111111111111111111111111111111111111111111111111111111111111
     00000000000000000000000000000000000000000000000000000000000000000000000000000000000000000000000000011111111111111111111111111111111111111111111111111111111111111111111111111111111111111111111111111112222222222222222222222222222222222222222222222222222222222222222222222222222222222222222222222222222333333333333333333333333333333333333333333333333333333333333333333333333333333333333333333333333333344444444444444444444444444444444444444444444444444444444444444444444444444444444444444444444444444445555555555555555555555555555555555555555555555555555555555555555555555555555555555555555555555555555666666666666666666666666666666666666666666666666666666666666666666666666666666666666666666666666666677777777777777777777777777777777777777777777777777777777777777777777777777777777777777777777777777778888888888888888888888888888888888888888888888888888888888888888888888888888888888888888888888888888999999999999999999999999999999999999999999999999999999999999999999999999999999999999999999999999999900000000000000000000000000000000000000000000000000000000000000000000000000000000000000000000000000001111111111111111111111111111111111111111111111111111111111111111111111111111111111111111111
     00000000011111111112222222222333333333344444444445555555555666666666677777777778888888888999999999900000000001111111111222222222233333333334444444444555555555566666666667777777777888888888899999999990000000000111111111122222222223333333333444444444455555555556666666666777777777788888888889999999999000000000011111111112222222222333333333344444444445555555555666666666677777777778888888888999999999900000000001111111111222222222233333333334444444444555555555566666666667777777777888888888899999999990000000000111111111122222222223333333333444444444455555555556666666666777777777788888888889999999999000000000011111111112222222222333333333344444444445555555555666666666677777777778888888888999999999900000000001111111111222222222233333333334444444444555555555566666666667777777777888888888899999999990000000000111111111122222222223333333333444444444455555555556666666666777777777788888888889999999999000000000011111111112222222222333333333344444444445555555555666666666677777777778888888888999999999900000000001111111111222222222233333333334444444444555555555566666666667777777777888888888899999999990000000000111111111122222222223333333333444444444455555555556666666666777777777788888888889
     12345678901234567890123456789012345678901234567890123456789012345678901234567890123456789012345678901234567890123456789012345678901234567890123456789012345678901234567890123456789012345678901234567890123456789012345678901234567890123456789012345678901234567890123456789012345678901234567890123456789012345678901234567890123456789012345678901234567890123456789012345678901234567890123456789012345678901234567890123456789012345678901234567890123456789012345678901234567890123456789012345678901234567890123456789012345678901234567890123456789012345678901234567890123456789012345678901234567890123456789012345678901234567890123456789012345678901234567890123456789012345678901234567890123456789012345678901234567890123456789012345678901234567890123456789012345678901234567890123456789012345678901234567890123456789012345678901234567890123456789012345678901234567890123456789012345678901234567890123456789012345678901234567890123456789012345678901234567890123456789012345678901234567890123456789012345678901234567890123456789012345678901234567890123456789012345678901234567890123456789012345678901234567890123456789012345678901234567890123456789012345678901234567890123456789012345678901234567890
     MKTRQNKDSMSMRSGRKKEAPGPREELRSRGRASPGGVSTSSSDGKAEKSRQTAKKARVEEASTPKVNKQGRSEEISESESEETNAPKKTKTEQELPRPQSPSDLDSLDGRSLNDDGSSDPRDIDQDNRSTSPSIYSPGSVENDSDSSSGLSQGPARPYHPPPLFPPSPQPPDSTPRQPEASFEPHPSVTPTGYHAPMEPPTSRMFQAPPGAPPPHPQLYPGGTGGVLSGPPMGPKGGGAASSVGGPNGGKQHPPPTTPISVSSSGASGAPPTKPPTTPVGGGNLPSAPPPANFPHVTPNLPPPPALRPLNNASASPPGLGAQPLPGHLPSPHAMGQGMGGLPPGPEKGPTLAPSPHSLPPASSSAPAPPMRFPYSSSSSSSAAASSSSSSSSSSASPFPASQALPSYPHSFPPPTSLSVSNQPPKYTQPSLPSQAVWSQGPPPPPPYGRLLANSNAHPGPFPPSTGAQSTAHPPVSTHHHHHQQQQQQQQQQQQQQQQQQQHHGNSGPPPPGAFPHPLEGGSSHHAHPYAMSPSLGSLRPYPPGPAHLPPPHSQVSYSQAGPNGPPVSSSSNSSSSTSQGSYPCSHPSPSQGPQGAPYPFPPVPTVTTSSATLSTVIATVASSPAGYKTASPPGPPPYGKRAPSPGAYKTATPPGYKPGSPPSFRTGTPPGYRGTSPPAGPGTFKPGSPTVGPGPLPPAGPSGLPSLPPPPAAPASGPPLSATQIKQEPAEEYETPESPVPPARSPSPPPKVVDVPSHASQSARFNKHLDRGFNSCARSDLYFVPLEGSKLAKKRADLVEKVRREAEQRAREEKEREREREREKEREREKERELERSVKLAQEGRAPVECPSLGPVPHRPPFEPGSAVATVPPYLGPDTPALRTLSEYARPHVMSPGNRNHPFYVPLGAVDPGLLGYNVPALYSSDPAAREREREARERDLRDRLKPGFEVKPSELEPLHGVPGPGLDPFPRHGGLALQPGPPGLHPFPFHPSLGPLERERLALAAGPALRPDMSYAERLAAERQHAERVAALGNDPLARLQMLNVTPHHHQHSHIHSHLHLHQQDAIHAASASVHPLIDPLASGSHLTRIPYPAGTLPNPLLPHPLHENEVLRHQLFAAPYRDLPASLSAPMSAAHQLQAMHAQSAELQRLALEQQQWLHAHHPLHSVPLPAQEDYYSHLKKESDKPL
Predicted Results:
   1 ..XWWJSAEVAAESCEAAAJVCYQAAAAAJAWBPYSYBJBYAAPZPPVAAYBAAAAAAAAAJVWBVWCSYEJADAAAYAVASJSVACSSWYVZAAPSCADWCAAEAABQYAEYWZQSADJVAFYPVAVVJVBCAYJJYJVYESCRAJCJAVCAAVSWASWPCCSPWCCAPWCCAPAAVVCAAWEEWYAJJBADAZBPCPJSVAVAAAWCYAPCCJSYAVPBQZZSWAAEWCSWCZVJAPZVAAPIYZPCAPCPSCSWCEBSWSZJAJASPCVSBVAWWSVAVASWCCPCCJVVPJJBVBAPVCCSAAAAVAAJAVSCYAJAPBVCYAAPCWVEEJARCAESWCSCZJRWCADWJVVVJZCCCBEAPCCCAAEEVYAYJJJVYAAAJBJJJJJJVSPJYASYAAAASAPCPAJWCSCAEEBVVVCCYWPCSWPBSYPRAEEPCCCCCJJAAAAJJSJPCSVCPCCSPVJAAVPLCCWSESBBBUYAAAAAAAAAAAAAAAAAEEADAJJCCCYAAWZCWJQVAZBQEEAAACSJVDAAJJCAPCYWYACACZYSABLADAASWCJFCSJCAVJAPWJAJEQJPPJDAAYJVAPCYAAYWWCACSCSCJEYDVAVDCAEAAEDAVCAEJWDDJPCYCCCJCYWECYCVJAVAEEJWSAPCYAPWSAJDJSCYSCSCYCCYCVPYDCAWSAJBAAPRWCCCSVWZAJJSCCCCCCVCCBWPSCSJEJBAJAVYAAAARJEAPBPWCAYPSCCCCEBASPWAPVVVAAAARRAADAASSSVAVAVJBEWSAYAWJAAAADAAAAACAADAAAAAAAAAAAAAAAAAAAVAAAAAVAAAAABDBAAAAPAAAAAWPVAVAACDSPPVAWBAJAADVBBBASCYJPCAAADAAAASAAAASSYJAPDSYBSWCAVJVSYAAPSPVJVBEAWAPVAAAAAAAAAAADAVAAACWYAJSEBYSADCACQYJCCCAWJWAYBVSWBAAPSPCYAPCYYYYPVAVPCCAAAAAAAAAWYDAWWCYVAAAAAALAAAAAAAAAAAWYJYAAAAAAAVBQSBSSBYYSAAPDEBAAPAADAAWAAASJCBCBAVWSPAPSAADJYWAYAAJCJVACCSVAAAPBAADAQDABCAABJACVJESWAVWSWAAAAAAEAAAAAAAAAAAAAVABWWVAJVJJABCWCVJABEDDCACAAAYP..
   2 ..   CV A  VJPABDDJPCWS VDWJD$ZSJCVPCABEJJPQDJEADWAE DDCDDBDDAJPS VJZD PPPDEBAJYVRRCA F AJA YDCACBC BYBJADYSWSPBVAA$VBVSY EVJ Q  WCPVSAVEBAAW DPCJADAJAPPWCRA CVB SCVCRARCC  J    CVJEP  AVSWEEYA E C J CA LD  BWJWWSJ CS  YYPPUPJRJAJ VBW WWVCPAVDAPWYAVDY  C VAPBJRLAYWBWPAWS WJCSABZAWAPBBBVAJSCAAJ  E VEJCWACJPPCBPJEWBBPWDCSBACJZDSCS Z  WP YQAPPWCJYYJPRRCBC  WSYWAEDAYJVB     YSPBAAAPAYDEAJAAAAAAPAVPP WCCEDWYSY Y Y   JJADC     V CSVJCEYVJAV    YSSS  LPEBAAD W $ SJ YWJSSZYZ J  RE JAJW   DDDDDDDDDDDDDDDD    DVPYYZCVJSDBVWDABWE      W BYAVVCVA CDCFCYAWV S S JDPCJACYAP CCWPAAVBAABYYJWCYA   CP  QPCJW V VWWY FRABBARSAVADAEBAADJPZCAZAAYJPCPJ  VP WJ JUASWDBCWCYVJS DWC CYAPCJWYAAEWSW WYAZPJVCYDCYD $SPSWSCYVCYACCAWPWASYWSEVCCDAEZADDA PABERDAPY JRCCY  BCEWWWSDJEBBC   ADEVR  DVAVY    CYCA W BCYZYBEDSDDCJVDDWADCEDDDDJBDDYVDDDDDDDDCYDDDDCYDSVDDAWECYDPQSSD  D   JCSWZB  Y ESWAESARECQDJWJAWSPBDAYDYDAPY     ES AB R BZCWAPJWJDRYJBVSADCPJJBDDDDDDDPBDBADAEDPAASJWEWEZADVAD WSBVEJBJSC   QVSSDSWV PSJSW     YDJWYSWDDDEEDDPCDAPBV  YLCWDDDEDDDD DDCVDJCQBVDCDDEDWBV YA  W   DRAWP BJ  QADDAJBVJAB WCCSAJBCJDJ J SVWSJBPYBCVWWY   Y DDD D ADPAYDAABSYSVWPCACAVD SRD   JCVDDDDDDDDJ B   YCPCSALESCBZYYAV  DDSZEZVW..
   3 ..   A  D  YABPSJVVWWV  YBDDJABCCWRAPJAYEBWJYBAYVBWS J PJBPW DWJ  AWQQ YCACBCRSBJC A  R JCJ VYDWW   A WWWCSWPJJJACYSCPBW  B W    AWWBVVABVPWE  YERYBYBWWS SE   SJ  VCF WS    B    YPSWA  J  A YQ  P     Y       VSB B  JV  JVVYCAAEEVP  A  BBYYBCWYJBPVQSVS    YJDSCEBWEDCCJBA  CSSWJPCYCBCCLSYCSV  U   V YDA SYBDJWSEW PDPW SJPCRWYPCSC R     Y A PCCVYW DEVSDAJW  BRBSSSSJCSYW     W VEVVVCPVCDVPVVVVVVCVSAV CADZ JCWC A     SPBAA     W  YCEZWCWAEE    SWWW  WDBVBPJ C W B   AVYYYJA S  D   WVV   SSSSSSSSSSSSSSSW    YEAPSW JCPCPS WYLPJ      S C CWYAEW  W  ZWJJ      EEAW EPSC  YYAYPVJJJPSPZVBA D   VY  YL YC S   S  BEBWEJJJBCSSDDEDEEAJDZ BVWZC DWD   L AP LYC  W LYPVW W JCS DWECBPCV JWPWF YCCSCADP JASV ZCJRPAYCCRDZBVSPSSYYAJBYAFPWEAPEP    VCPCW WB CVWBJ  YVWV BA BCEAY   J   J  BJCYS    YEVJ V CVVAVJFPJV ASDVJCDBPAVJ BDDB DDJSSVSVS DAJSS DAJVECSJAAEDYDA$JE  C   YPY     J  YDVDWEWWFYYC  CJWSVWBZSCJCWS     VC    W DVYCVBBADJAEASWACAJVVCY VRPVSYEDCDPJDQVZJCV FBVCRCPAPC BAWWWYSPBA   DQEZEBCW SADAP       YAAVDRJRBBEEBPCPJSC    BDRJEAJ    VJDEEBSASSBVSWDJDEA     C       R  P    B JBYJRCW A DYDWYSVBB A R CPWWCDSBSJAZ   E JJW Q   VWCJ$ PRAAABSRSAYQP  E     JEVBYEPSYED      WSYEV YBJWW P    A ADJBAJ..
   4 ..          BJYYC PCAJ  JVHEPDYDPBCQSREWBDYWWAB $SCL W  BRDB WC   CAJA  WVJDDVVAPY D  E V P  V      P JBDPJVJWWSS CPY JP  L Y    DBJE DEWSYQZ  S BBZEVSBC  A   BW   JL  Y    P    S CPL     B V   B             ZCJ W  WP  CSCBACPZWWS  V  YYDZAJYZBVBJJ YJ    BSAVLBYCJPJYWV   BWYJSCBQQYJEJAAJPJ        AJW PPJ EC S  BQWJ BPAEVCSW V  F     C V CLBPRP P   SWSB  JAEBYYAYVLPE        SBBBSVDPZPVBBBBBBSJD    BPD CWD  Z     Y JCS         Y YSAABS     WBBY   WSWWS    C V   BAAACS     E   VWE   WWWWWWWWWWWWWWWJ    JB WWJ SD  SB AJYJW        A SPCDYB      L$P      P WD USRQ  R FCWPBAPVFV W       SC   W DS        CACFLPSVEAWBBBWWYJPSVY YJVCS VJ    W CW PZP  S V   B   YLP  AWV L   Y BYL  UWCJRJ  YB   AWSJBYJUJYQYWRJA PPVPPYVBWWVBWA A     YZSE CZ SCJSS  WWJP JB EACDV   Q      EDYQ     BRYY E AYC$JCSJBJ SVPEWVWEJVSS W  E  YCW      VDCW  VDCCDJJY JBWSW BYB  P   BV         ZVBJEJEJWP W  D BDDEJDPD W V     BW    A ASEAZJ VSBWZWYBDSZSCZBA SJV  WDJBY  YDJDWD   W RV YBB    CSPSJY      CJAB AY JR C        BDDABBYVDDJCJAACEC     DQJW$WE    WSJLVPAPPBJD YB JYW     J       A       E VPSW  J   BAJAE$DWE E    ZBRS PSJS W   D A E       SVW CWWRPD VY D E        SP SVYSBJWB         BW  VPES V      DYV SS..
   5 ..          SV AP WYP   S VCBPD A DJVSLVAWZ$ADJ JPVA    CCJV BA   D  E  YYSRSJLJDA J  S E C           DYJSZA$FBP  JVZ AC  R B     YCA QB  V L    DPEPS VJ  C   PC    V       W    A  DJ     D C   W             SQ  A  BJ  LCYQPQSDBPV  P  AACAYECBWCDP  ZC    CV RPCJDVAVPCJ   PPABVJREPPSA DBYWW        CW  YWW  B A  ASAY CSVLWFWS Y  W     J   $QFBVB S   WFPV   CSZVJCPZWSJ        VPPPZWZJVWYPPPPPPZCA    Z Y YVP        $ SBE         A WCDE       AVVZ   BYDE     F J   JYBBJ      S   JSA   PPPPPPPPPPPPPPPP    CU  AP CP  JY ZPPSV        W JSWVPJ      CP       A EY B V   B YVYQPEBEWW                CV        RB RWRAWJEB SVJLZWBBYR APZ W  A      JB WWW  V P   S   PJJ  B   Z   B C    A AB    P     YABEBWAPSPSAWBB YWWCBEPS JYCCW C     S DV    WA     CBBY SC DBW J   E         W      CD    EDPCSPYSCS WWBJPDJ W W  Y  Z   S        PS    PSQPVWW LW PV YCR       Y          JEBVQACS     Y AWJSCCCV J       IV    L LDWPSE   ZCCB ERYCDJBSD E    SBEWE   YZJPP   L SW  ES     YYVPB      P CV YB VV J         VSJSVVJJLWBWBZS         SECBC     YBJBQPZC YW Z   JS     S       B         QWCY  P   SCSCWVAA  R    VCJW CY F C   S S V       DYP WABWJJ Y  J R            C WL BS         RE  WDJY S      W Y  B..
   6 ..          C  PS   S   D CYEWE S SY PCCWYBABRS YES     EES  PS      P  VJYFESWE D      W B           PDSVDECVDW  VWP QB  C S     ELS J   W V    WEQCY YW  V   C             Y    B  ZV     P J                 BZ  P      BE AJYYPCCA     JCZWCSEPZJC   W        WV VRABWVYW   LEJY SYCYZVV FJ LF        WS  AEY  J    V JC VCQREJBB C  Y     S   DYLYJV V   PPC    EWYBVWZWC P         WWWAJSQWSCWWWWWWAWJ      R LDC        B YFJ         J   V        LPPB   ERUD     Y     LZDPD      A   YEJ   JJJJJJJJJJJJJJJB     P  LS Y   DC YCWAA        Y PJSBA       J        S  Z P     E SEZZWVDWYZ                SY        EF SSBBBWWE JWDBBCS W    I Z  B      LV  B   R A   C   SSQ      E          P VS    S     ACLSWBP $W$PAWS AEBBEJAJ Y DBY E     V J             EPS  D WLY P   D         D      LU    S WJCRA DB  DJB YE V B  J  C   Y        WY    WYDSPPP WD VC  BC       B          LPSDV PR       V C PF   B       SP    E   PBYA   LDWE  W YEBSYS B     WSPS     Y        C  JE     BBWCC      J DJ BR WW           $JPEEBEWRPQSDJ          C PDR     EPDLWDJD  P     SE     V                  DPS        YEAYWL  S     ES  WR   J   $ P         V F JBCDYA W                       V             ASZB          B  A..
   7 ..          R  VV   Y       V   V WB Y QVPJCSSC BJ       JY  VY      B    QPWEER        B D           CPPWBJVLSA  PZJ RA  A A      S      D B     SAVC SY  L   R                      E       R                 PA  V      ZW WSVLYDJC     PP QSWSSCWQ            CW PEQQEAVY   VC P EEUJJW  YS  R        SY  VZZ       D C    D LY Y W  B     Z   Y AS Y     VYE    FF ZWJSE            SSSYZPSYDASSSSSSYPY      B   E        P CWQ         Q   B         AA     V C     B     VW VP      B    AQ   EEEEEEEEEEEEEEEC     W     $      VDJBS          WYJWC                     R     Q DDB  CVBCA                W         L   VYYPYSY   S SSY        B  V      P   P     J   Y            S          S BW          FVWJFES VCBSBEJ VZ SARDP B WYD         E              LB  R  Y      W         B       E       VZYD P    SC J    P  E  P   P        CP    CPYCQ S  J  Q  PS       W          PCVJS L        Y   SW           U         VSDC    E    C  PRYV  P     LW                S  QY      JQWS        RS EC B            CRFR S LWCJ $S          P RJ      WSBSVY W  R     AQ     Y                  CJB         VJEYR  B     VP   W       R Y             V JBDE                                       RYAJ             C..
   8 ..          L  LY           S   I $D   $FRRV V  U        SE  SE      U    WVZPPW        P              VB CDACVR      LY  J D             S S     ZVRE AV                             C       Z                 YV  Y      A  VFBEBRU      SV SEDB QSA            YE EB SPBQP        YFWZ                     B         S E    W AS A J  J           EA       B A     J   PCB            CCCJCWYJBECCCCCCJZB      J   J        Z  SV         W   J         JJ     J V     Q     YC E            CS   VVVVVVVVVVVVVVVS     Y     P      JSCYP          BZBPW                           V JJC  LYCE                 R         V   DSCEDY      WB            E          $     R                F            JA          B  FRL  WVCZDP  B  DF ZC    PL         F              SJ  W  W      B                           DC V       B    Y  V      Q        BQ    BQJR        S  R                     LLB S        R    V           Y         RDCD    B    Y   S    W     JV                   WL      VZV         Y   A D            YFBC   PPSW J             BY      ZWWCR                                      ED         DSJCC        LY   F       Z               S PEWC                                        BY               ..
   9 ..             JU           W   W B    DDSDZ             VR   P              WBX        Y              CL VR  CC      E   S               Z C     V UP ZB                                                       AP         E  DWDQC B      C  DWLD D V             S AJ YSED          WJ$                     J           Y       P E    V           R        E       R    WD            EEEBSBZBESEEEEEEBEC      L            V             B   Z         EE     D P     R                     PU   CCCCCCCCCCCCCCCV     S     Z       QQ C          YCDE                              B E  W SJ                           W   C EYP       CR            Z          E     D                             PQ             L     J  LR  J  ES QY    $S         Y              V      P      L                           AB E       S    J  C      B        SB    SB          Z  V                     PRC                                      YPS                  Y     S                     W       $Y         J   E Q            J D    S BP L              Q        CRD                                      DL         B$LBE        R            B                 SPBF                                        AS               ..
  10 ..             RW           C     F     LVE               V   B              ZCS                        U     EF                            J       ZD EE                                                       JW         W  SYR S R      D  JVBJ V Z             Y ZV CDRB           PD                     L           S         F                V                     VJ            YYYE CESQDYYYYYYED       S                          E   P         FF     W       V                     Y    YYYYYYYYYYYYYYYE                   ZR Y          U$ER                              L J  Y YR                               J FDQ       JV            Y                B                              V             A     $   Y  L  LZ  E    DV         B                     R                                  ER Q            Z  S      W        ZW    ZW             W                     WYR                                      JRW                        U                     D       RE         P   L                E      VS                S        P                                        W          LQP F        S            P                 E SR                                        EP               ..
  11 ..             U                  Z     SCL                   R               DU                        V     I                             R       BW JZ                                                        $            CBZ V S      L  ED L E                  F VIZ$           $                                  I         L                                       R            QQQQ ERLCWQQQQQQQB       V                              W         YY     C                             R    BBBBBBBBBBBBBBB                    BF R          VEPS                              V S  S QV                               P DRR       VY            D                W                                            R         I     Z   L    S          P                     V                                  SP W               I      Z         Z     Z                                   Z W                                      QF                         V                     V                      P                R       V                                                                             P                                           R Z                                         FV               ..
  12 ..             W                        CEQ                   D                C                                                            A       FL                                                           D             V  J H      Z  BJ                      L EL Z                                              L                                                 S            LLLW JWPL LLLLLLW$       W                              $         LL     L                             L    QQQQQQQQQQQQQQQ                    US Z           B L                                   F LD                               U P C       PP            F                F                                            Y                   D                                     F                                  V                  R                                                          R                                                                   Y                                                                                                                                                   Y                                                                                       L                ..
  13 ..                                      P S                                    F                                                                    D                                                                          E  Q Q         VL                      P UY E                                                                                                             RRR$  VQR RRRRRR$Y                                      E         II                                   D    FFFFFFFFFFFFFFF                     V D           Q U                                   Q ZL                                 W         U             L                S                                            V                   X                                     J                                                                                                                                                                                                                                                                                                                                                                                                                                                 ..
  14 ..                                      Q                                      L                                                                    W                                                                          I               R                        XR F                                                                                                             ZZZD  XRU ZZZZZZDR                                      F                                              Z    ZZZZZZZZZZZZZZZ                       L             Y                                   R  Z                                                                                                                                           U                                                                                                                                                                                                                                                                                                                                                                                                                                                                                       ..
  15 ..                                      R                                                                                                           Q                                                                                                                   Z$ R                                                                                                             DDDR      DDDDDDR                                                                                      Q    LLLLLLLLLLLLLLL                       U                                                    P                                                                                                                                                                                                                                                                                                                                                                                                                                                                                                                                                                                                                                   ..
  16 ..                                      U                                                                                                           S                                                                                                                    Z S                                                                                                             FFF       FFFFFF                                                                                       U                                          $                                                                                                                                                                                                                                                                                                                                                                                                                                                                                                                                                                                                                                                                                        ..
  17 ..                                                                                                                                                  L                                                                                                                    O                                                                                                               III       IIIIII                                                                                       F                                          F                                                                                                                                                                                                                                                                                                                                                                                                                                                                                                                                                                                                                                                                                        ..
  18 ..                                                                                                                                                  $                                                                                                                    F                                                                                                               UUU       UUUUUU                                                                                       I                                          H                                                                                                                                                                                                                                                                                                                                                                                                                                                                                                                                                                                                                                                                                        ..
  19 ..                                                                                                                                                  H                                                                                                                    H                                                                                                                                                                                                                      $                                          Q                                                                                                                                                                                                                                                                                                                                                                                                                                                                                                                                                                                                                                                                                        ..
  20 ..                                                                                                                                                                                                                                                                       Q                                                                                                                                                                                                                      H                                                                                                                                                                                                                                                                                                                                                                                                                                                                                                                                                                                                                                                                                                                                   ..
  21 ..                                                                                                                                                                                                                                                                       U                                                                                                                                                                                                                      O                                                                                                                                                                                                                                                                                                                                                                                                                                                                                                                                                                                                                                                                                                                                   ..
  22 ..                                                                                                                                                                                                                                                                                                                                                                                                                                                                                              X                                                                                                                                                                                                                                                                                                                                                                                                                                                                                                                                                                                                                                                                                                                                   ..


>sp|P10275|ANDR_HUMAN Androgen receptor OS=Homo sapiens OX=9606 GN=AR PE=1 SV=3
MEVQLGLGRVYPRPPSKTYRGAFQNLFQSVREVIQNPGPRHPEAASAAPPGASLLLLQQQ
QQQQQQQQQQQQQQQQQQQQETSPRQQQQQQGEDGSPQAHRRGPTGYLVLDEEQQPSQPQ
SALECHPERGCVPEPGAAVAASKGLPQQLPAPPDEDDSAAPSTLSLLGPTFPGLSSCSAD
LKDILSEASTMQLLQQQQQEAVSEGSSSGRAREASGAPTSSKDNYLGGTSTISDNAKELC
KAVSVSMGLGVEALEHLSPGEQLRGDCMYAPLLGVPPAVRPTPCAPLAECKGSLLDDSAG
KSTEDTAEYSPFKGGYTKGLEGESLGCSGSAAAGSSGTLELPSTLSLYKSGALDEAAAYQ
SRDYYNFPLALAGPPPPPPPPHPHARIKLENPLDYGSAWAAAAAQCRYGDLASLHGAGAA
GPGSGSPSAAASSSWHTLFTAEEGQLYGPCGGGGGGGGGGGGGGGGGGGGGGGEAGAVAP
YGYTRPPQGLAGQESDFTAPDVWYPGGMVSRVPYPSPTCVKSEMGPWMDSYSGPYGDMRL
ETARDHVLPIDYYFPPQKTCLICGDEASGCHYGALTCGSCKVFFKRAAEGKQKYLCASRN
DCTIDKFRRKNCPSCRLRKCYEAGMTLGARKLKKLGNLKLQEEGEASSTTSPTEETTQKL
TVSHIEGYECQPIFLNVLEAIEPGVVCAGHDNNQPDSFAALLSSLNELGERQLVHVVKWA
KALPGFRNLHVDDQMAVIQYSWMGLMVFAMGWRSFTNVNSRMLYFAPDLVFNEYRMHKSR
MYSQCVRMRHLSQEFGWLQITPQEFLCMKALLLFSIIPVDGLKNQKFFDELRMNYIKELD
RIIACKRKNPTSCSRRFYQLTKLLDSVQPIARELHQFTFDLLIKSHMVSVDFPEMMAEII
SVQVPKILSGKVKPIYFHTQ


PFVM

     00000000000000000000000000000000000000000000000000000000000000000000000000000000000000000000000000011111111111111111111111111111111111111111111111111111111111111111111111111111111111111111111111111112222222222222222222222222222222222222222222222222222222222222222222222222222222222222222222222222222333333333333333333333333333333333333333333333333333333333333333333333333333333333333333333333333333344444444444444444444444444444444444444444444444444444444444444444444444444444444444444444444444444445555555555555555555555555555555555555555555555555555555555555555555555555555555555555555555555555555666666666666666666666666666666666666666666666666666666666666666666666666666666666666666666666666666677777777777777777777777777777777777777777777777777777777777777777777777777777777777777777777777777778888888888888888888888888888888888888888888888888888888888888888888888888888888888888888888888888888999999999999999999999
     00000000011111111112222222222333333333344444444445555555555666666666677777777778888888888999999999900000000001111111111222222222233333333334444444444555555555566666666667777777777888888888899999999990000000000111111111122222222223333333333444444444455555555556666666666777777777788888888889999999999000000000011111111112222222222333333333344444444445555555555666666666677777777778888888888999999999900000000001111111111222222222233333333334444444444555555555566666666667777777777888888888899999999990000000000111111111122222222223333333333444444444455555555556666666666777777777788888888889999999999000000000011111111112222222222333333333344444444445555555555666666666677777777778888888888999999999900000000001111111111222222222233333333334444444444555555555566666666667777777777888888888899999999990000000000111111111122222222223333333333444444444455555555556666666666777777777788888888889999999999000000000011111111112
     12345678901234567890123456789012345678901234567890123456789012345678901234567890123456789012345678901234567890123456789012345678901234567890123456789012345678901234567890123456789012345678901234567890123456789012345678901234567890123456789012345678901234567890123456789012345678901234567890123456789012345678901234567890123456789012345678901234567890123456789012345678901234567890123456789012345678901234567890123456789012345678901234567890123456789012345678901234567890123456789012345678901234567890123456789012345678901234567890123456789012345678901234567890123456789012345678901234567890123456789012345678901234567890123456789012345678901234567890123456789012345678901234567890123456789012345678901234567890123456789012345678901234567890123456789012345678901234567890123456789012345678901234567890123456789012345678901234567890123456789012345678901234567890123456789012345678901234567890123456789012345678901234567890
     MEVQLGLGRVYPRPPSKTYRGAFQNLFQSVREVIQNPGPRHPEAASAAPPGASLLLLQQQQQQQQQQQQQQQQQQQQQQQETSPRQQQQQQGEDGSPQAHRRGPTGYLVLDEEQQPSQPQSALECHPERGCVPEPGAAVAASKGLPQQLPAPPDEDDSAAPSTLSLLGPTFPGLSSCSADLKDILSEASTMQLLQQQQQEAVSEGSSSGRAREASGAPTSSKDNYLGGTSTISDNAKELCKAVSVSMGLGVEALEHLSPGEQLRGDCMYAPLLGVPPAVRPTPCAPLAECKGSLLDDSAGKSTEDTAEYSPFKGGYTKGLEGESLGCSGSAAAGSSGTLELPSTLSLYKSGALDEAAAYQSRDYYNFPLALAGPPPPPPPPHPHARIKLENPLDYGSAWAAAAAQCRYGDLASLHGAGAAGPGSGSPSAAASSSWHTLFTAEEGQLYGPCGGGGGGGGGGGGGGGGGGGGGGGEAGAVAPYGYTRPPQGLAGQESDFTAPDVWYPGGMVSRVPYPSPTCVKSEMGPWMDSYSGPYGDMRLETARDHVLPIDYYFPPQKTCLICGDEASGCHYGALTCGSCKVFFKRAAEGKQKYLCASRNDCTIDKFRRKNCPSCRLRKCYEAGMTLGARKLKKLGNLKLQEEGEASSTTSPTEETTQKLTVSHIEGYECQPIFLNVLEAIEPGVVCAGHDNNQPDSFAALLSSLNELGERQLVHVVKWAKALPGFRNLHVDDQMAVIQYSWMGLMVFAMGWRSFTNVNSRMLYFAPDLVFNEYRMHKSRMYSQCVRMRHLSQEFGWLQITPQEFLCMKALLLFSIIPVDGLKNQKFFDELRMNYIKELDRIIACKRKNPTSCSRRFYQLTKLLDSVQPIARELHQFTFDLLIKSHMVSVDFPEMMAEIISVQVPKILSGKVKPIYFHTQ
Predicted Results:
   1 ..SJAVVPAEWCCCYAEJE$ZAAAAAAAADAAABWVRCYBWVAAAAJWCYAVAAAAAAAAAAAAAAAAAAAAAAAAAAACADJBVADAADWDYQSPZJAAARWYJAPEBEWZAPBVAPSABAADJBVBAAASSCYAPAAAABVBWSAAPCCCYWVAJAAWCYJEAAWQSAPVAJWAEWZAAAAAAAAAPYAAAAAAAAAAAAACAYAQPEAAAAQSLCYAJVYADCAWYDAPPAAPADADAJAEAWYADYDAAAAAAWYDJBAUYSBBESACQSWCYSVSCFZVVSJWALWJCYAAAAQAVWJJAJAEEWSAYAJJWQAPAPAEQCZBJJAAAAJVVJACWCVJEJAAWBAAAAAAAAADAAAJASBVAAAACCCCCCCYCSSSJAAAAVYCABVSSAAAAAAAAAWVCBAACEAAQABCYYPPAJVAWAAJYSSWAAABYAYAJVCAVCPPCCCCCCCCCCCCCCCCCCCYWAJSAWASYVAEWYSAAJAVAPPVSERBADBJSVVJAWCRSYCJCCSSSQVJJJJJJVJVJJVBBJAEAAPJAAJLCBCAAAWWWEWERJBSWVEWCSWAWWSACYAADDAADBAAQSAPVABEVAPYJWSVAJDWAAAJVAAEDAAADAASEBYCBADAAAPAAVAAAAASAACSJWPCPJAAAAABABAAJVDYERPSVAAAAAAAAPSWSEBWSWSVAPCSWYAAEAAAAAAAAADAAAAADDAAAAAYAAAAPCYAAAEAAADDAAAAAAADAAAAAADDDPCYAPSBWCZAJBBWZAAADAJVJVAAAAAAAAAAAAADDPSBWYDAAAAAAAAAAAAJWSVPCCAJVAAAAAAAAAAAAAAAAAAAAAJVJBAAAAAAAAAAAAAAAAAAAAAAAAADAAAAAADAAADPSBBWCYAAAAADDDAAAADAQYAJWCSBVP..
   2 ..PDBSBWJVJJ B CVDWWY CWDDED ABSDAC S ZSJYBQDDACZJJWSDBEDDWDDDDDDDDDDDDDDDDDDDDP YB ADADDAQACPPYY DVLCCCECRAEBAYD SBJYYYJDD B  A  Y  ZVWBSDBDAPYPCYJVQWB AJYAJPJSVBPDSPWY ZWJPARZC VVVDDDDDPY  R DDWDDCDDDDVBJEPVADDDDAAPSVJADAJEACPZVDVAYCJSADADSCA ABYZSASDDDDYYZAAADAW   B DADYJWAAJWV    CAAECCAPADJP ASAAVPSWVCBBY WQASPACZYAPS YAQSAWJJDAWASEEBVYBPE WA SVSDDDVDDASVE DB CJDDWPSYSSSSC    ADBEYYJWFSA  D WDDDD  V PDYDABVDYEZAWDAWPBCWADEAE  J WJWZWPSWDV DQYQAAAAAAAAAAAAAAAAAAAJVYAYBAWA AVBJ JJDAEYBAASYPWCV    CAAWAASYCSP  W  AB      ABWCSJ  BWYJWAAJJBWRV D       LVDPYYSBEJWV VS BEZDDAAEDAADJVADJABWAEQEV  ABWA A VPP DDDADEDVBPYJCCSZDABVDJVSYBBYDCASPWVPVWSWBS PDCEEEBCPPA JAABYDSBBDDDJWCCJSWBBBB S WCJADDADJDBDDZSCAY BDEAADDLPPCYJCJJBZDDDL DEAA  WDDDBAJEYDVDVPJVWZPAA BPVVADAAADDDA PYAADDJ DDVJPCYDQPAJBECWAY DDDDBEDDCREBCYVAAYABVDJVDDSDP DVPWWSDDYPBASPVDSWDDDBDDDDDDPPSW DBSDDYDADWVRDDBB VAJWAEEWZDDDDDAASBBCBAJAARBBSB $Y..
   3 ..ES BAADB   W PW  SA WV  DL JVJESP Y     JDJZVJSSSBWBEDBEBSSSSSSSSSSSSSSSSSSSY     J PSSJV ZCAB   ZWSPZCJESAWBVS WJ    AYV D     Z  Y VJDWPJJQSCRVSASS  CAQSV PBCAAEDASD YCSVJBBZ SYWJWCCS    D  EBSY WEBPYESJJBWRWWVJBWZPZBADV PVVWWBJCCVAC   BAJW VASAABDWCVY  V PSJCP   S  SJZCVSJALW    RV SW YJVSCV BYBSPWVDPR  V  YCBB WDVBRC ABCEWVVQCWBPAJWPSAAAA PE YBPBZJDY WJER    ADCEPWYSWWWW     EERDDBSV AS  V BEBEJ    AAVPJVWEAPAJPZJSJWSCYCDVJ  E PDEBPWEPSW CJAYVVVVVVVVVVVVVVVVVVVPZSBCDBLR J CY AZYLCAVW CBACYS     CZB JBC WV     P       EEPBBA  V ZDDJEQEAEBY W       SBQCVPASVSBS YB EWV  E DECDVDZBJVY  BY A    EYZ   SQ    A  B  DQCBSEEARSDDPA$EBDEPPYJWSAYARJ JSV  EYABSVPBWZ AS AABCSDJCEBBYAYBEPYJP   Y ADWEDJDJJVJYDDJB D BVE  WRBVJVPSBWVSY A  DE   D YEE D DJDEAJVWVSDCE  BWD EEZD      LW     S  DDDD D  A WBZBD    EDBEEE BABEUPJZPWYJYBJED J JDCLCDJB WPVCVYYDD Y DWELVSVDDDV PWCYBDR  BWDEEAS BBWBSSLVA    BBEEJWSYBLPDVSEB    ..
   4 .. A EPCPD     DP  C  DB     PP  DE W      YSLPPWCCPEESBEBVWWWWWWWWWWWWWWWWWWW        BWWV  AJYW    EF  QYAJSVJA  JP    DZ  W        P JWESCBSAPY  DWWP   YDVS BVASDJRSCR CYCWLEV  YBD JJBZ       BVW    WCAVCBEABYYJYPV PAYY$PD DSAEPEUSDSCR    CED DVCQBJWLE P  B BRZ S      DACPBVECCF    VY BF VADJSY EBYRZACPDW      PYD DYUYWA DSAWEJEWWYEYVBRJRDSDP BS CJRCJCWV PBCJ    BPWBC$ WBBBB     BCW E P  WD    DWJ W     JZCBW SCWPBACWJCCJDCPZPL     ZASEEDBB    VZPPPPPPPPPPPPPPPPPPPAPZPARECC W W  C JWDB C WAWJ Y     WSD SC  BW     D       PAL  W  E  VC S  VBFJ B        E E  CRPYCY  P W    J B   PCACSBD  S  S    YVB   BV    B  J    AAWWPJVVQBQ WJEEWBJDD WBBFSB AYR  VBWC D WAP B  VBRWJJV SWCZJAASAVS    S BV  JBBBV WDWJE  W   Y   SJADBS  E    D   R   Q   D E      Y    EJJ  JYY WS         E     B  C WZ      JE YW     SDSBB   EWAYYCQA PWWS   D  BDDVWWW QSYABSZJJ    JBC BSWVR  RCWBJP   DDWBVWD YSQEWWJA     EEB DCPDSDJOWPLE    ..
   5 .. Q YSY       VR  R  VD     YW  JJ B       CCDQVQPADRDSSWDPPPPPPPPPPPPPPPPPPP        QPPP   ADZ    DE   PCBCYRD  YW     S           W PRCBDCCBDA   BVB   DZPP ARBVBVBJPJ W WZ S   JJ  PSPJ       WDP     JQ ASBWPB BEYJ DW  J C Y$CSE EWQ YF    DDC EQP VWVBS C  F ZE         VPJBSCCRP     WS R  SSEBWZ WVWE ZJAC       SPL VSWJVW S S$PDCDJVYSPPBAWCVBS CY VSVWVV    CL     SYEJEQ AVVVV     PB  W D   P      V       PCSYA C CDSCQCASPDJVJVWB     LY BSL      JAWWWWWWWWWWWWWWWWWWWCBCUWEJYY   V  D ZBVS    DVF D      WE B   YC              DB  Q  Y  EE    YPYW          S J  JAFBEA  J      C     YPYJBWB     B    RPP   JB    S  S    BWAZAW BSES PBPYDJZVP YSZSYV CZP     D E SYC C  WWSP EW B  PY D   A    V C   BEP S ZQCBC  E   B   YCBS V       B                    V    VYB  V E AW               C            LJ        WC VD   RCCZDV$P   CC   S  JECDJRE  YCJCC ZL    BWE JRRYB  WD V     ERVR  E  QZJEVB       W  VSJSEBWSJEAF    ..
   6 .. Y LCV       B   E         BE   B         PPLVYZYCVVWR SCJJJJJJJJJJJJJJJJJJJ        JJJW   WJC    VJ   DSDRCVB  R                  J BABEEVDJ B    YE   W$YB CYPCCWVDJ     A J   B   VVW        SCJ     WJ BYYCS  EJUW YC  Y B JQY J YDV SD     LL  CB ZEL   J  W YP         JBVRA D Y      C     BZCBJ P S     E       W V YACCZL W JBBYWYVBAWBSSC JCC   L WWDVYF           DEJPJJ LPPPP     RP  B     U      E       YB WJ W YWPJWSCB W ZQWSS     WD CB       CJJJJJJJJJJJJJJJJJJJJVCVCLVL     Y  Z CC C    J             E    Y               E        L       L             U  DDBPRD  V      S      Q$PC S     C    S      D    V  D    W D W  EJL   DS VV BQ JPJDQA BCB     J W  J  D  YDE  V  C  AP J   P    B Y   SQC W  PVPS          CWEC J       J                    B     R   W W UL               W                       R      WPW$ SS    D       EJE BEP  VSDWZ  S    E W WEJZ   CE       JBP   R  W YC V       P  W  EJEYZSRC     ..
   7 .. P WQS       S   F         V    S         WJY BA JFJ V VEEEEEEEEEEEEEEEEEEEE         EE    $ J         L   AD                      L LCRJJYFW       J   ZP C E  WJ WQB     Y     E    Y         VEE     YD WPCJ   SBVY FB  S   SW  A WEJ  L     WB     WPB         D         P ASF R        D     WRWDB   D     W         E  J SBJ P P SZPVLZJJZ AS ZWJ     LQJSPR            BVSVZ  AAAA     WS  V            Y       WD SQ B SYFZBYVV P  SYD      YL IR       D SSSSSSSSSSSSSSSSSSSSADYESD     L    LZ J                  D                    J        W       E                PJCVAP  A      F       PWE       V    U      J    Y  L      E B  URW   WR  Q J  QYCV   DVC     W C  S  V   ZC  Z  R  VD L              VVV D  JYQL          D D  R       W                    A     B     C  I                                       S       BYW  Y            P B  B   W E A  E    P B YYB    BY        PB         J P       L     JRWCBBWS     ..
   8 .. W DWB       W   D         W    D         VEE PV RJW   CJVVVVVVVVVVVVVVVVVVV         VV    V               DS                      S CSJCWPEY       L   CS D    DV Q       F     D    B         CJV      Z VLSY   LWWP  J  U   EY  R BJP  Z            DZ          J         B B Y          W     ESYPS   P     Y         J  B WCB B Z CPBPPCSCW FY  DV     BD Y               BWB   JJJJ     DR  P            P       SJ DZ F VVLVSVZL Y  YJB       P RJ       Z YYYYYYYYYYYYYYYYYYYBYFSZWC     P    VD D                                       A                S                YC  PZ  L      R       D V                           W        Y  CCS       S P   RWB    DD     L    C  Y       Y      Z P              C W E    SY          B W  W                                           R                                       V        JD                    L   C Y D  V    C   QBS    $P                   R Y              W UPCCV     ..
   9 .. E P D       E   V              I         QWC  P SY    JSCCCCCCCCCCCCCCCCCCC         CC                    LP                        EXWUYSL             V Q    YW J       Q     Z    P         JSC        EUUS   PSC   E  W   V   B ABB  B            RY                    W              R       Q     C               S    VDD     YSDSYRCDY H   YW                        LC    EEEE                      W       ZW PY P JCQ $E      ZBE         SZ       W EEEEEEEEEEEEEEEEEEEW $FJJP     S    SE P                                       C                W                 L  Y   R      B       J W                           C           JPY       W       C    EJ     S    Z                   W              F F C                     Y                                                                                   F        QV                        L Q J       S    WE    E$                     Z                DEE       ..
  10 .. B A L       Q   A              A         YYB  W EB    YYYYYYYYYYYYYYYYYYYYY         YY                     C                        Z YZVQP               W     Y P                  W         YYY        HCDO   VZ    R            SU   W            E                                    I       $     J               A    EJQ     DBSZSEUB       Y                        SV    FFFF                      S       CS V    QSR V$      ESQ         V$         BBBBBBBBBBBBBBBBBBBI PVDP            V                                         U                                  W                     W Y                           R           L$        D       E                                                     S P                     Z                                                                                   L         J                        I B P             V    LV                                      VLP       ..
  11 .. V   Z       Z                  V         BVW  $ F       BBBBBBBBBBBBBBBBBBB         BB                     Q                             WR               Y       E                  E           B        LZRZ    C    S            CV                J                                            E     R                    FSR      CYCEPPE                                      YYYY                      C          E    ZJV  B      RLC          I         LLLLLLLLLLLLLLLLLLLQ QZP             Y                                         R                                                                                                  YE        E       Y                                                       U                                                                                                         Q         S                        J R Q                  Q                                       SVY       ..
  12 .. C                                        ZBS  D         QQQQQQQQQQQQQQQQQQQ         QQ                                                    Q                                          Q           Q        PWW     L                 DY                L                                            P                          LEU      EREBDQI                                      LLLL                      Q          R     FW  D      WPL          Q         RRRRRRRRRRRRRRRRRRR$ REV                                                       H                                                                                                   L        C       W                                                       Y                                                                                                         W         B                        R P W                                                          ZJD       ..
  13 .. Z                                         S             FFFFFFFFFFFFFFFFFFF         FF                                                    W                                          U           F        UV      P                 R                                                                                         QL       RF  SRL                                      IIII                      R                 Y  F      VQR          V         ZZZZZZZZZZZZZZZZZZZZ  Q                                                        S                                                                                                            L       O                                                                                                                                                                                                    Z                                                               CF       ..
  14 ..                                           R             ZZZZZZZZZZZZZZZZZZZ         ZZ                                                    V                                                      Z         R                        Z                                                                                          Y       L    LR                                                                L                    Q      XRU                    FFFFFFFFFFFFFFFFFFF   W                                                        Y                                                                                                            R       Q                                                                                                                                                                                                                                                                    WI       ..
  15 ..                                           F             LLLLLLLLLLLLLLLLLLL         LL                                                    Y                                                      L         D                                                                                                                           $    HZ                                                                F                    U                             UUUUUUUUUUUUUUUUUUU                                                                                                                                                                                 R                                                                                                                                                                                                                                                                     L       ..
  16 ..                                           Q                                                                                                                                                                                                                                                                                                 I                                                                 I                    Z                             QQQQQQQQQQQQQQQQQQQ                                                                                                                                                                                                                                                                                                                                                                                                                                                               ..
  17 ..                                                                                                                                                                                                                                                                                                                                             F                                                                 U                                                  OOOOOOOOOOOOOOOOOOO                                                                                                                                                                                                                                                                                                                                                                                                                                                               ..
  18 ..                                                                                                                                                                                                                                                                                                                                             D                                                                 Z                                                  HHHHHHHHHHHHHHHHHHH                                                                                                                                                                                                                                                                                                                                                                                                                                                               ..
  19 ..                                                                                                                                                                                                                                                                                                                                             X                                                                 H                                                  XXXXXXXXXXXXXXXXXXX                                                                                                                                                                                                                                                                                                                                                                                                                                                               ..
  20 ..                                                                                                                                                                                                                                                                                                                                             Z                                                                                                                    $$$$$$$$$$$$$$$$$$$                                                                                                                                                                                                                                                                                                                                                                                                                                                               ..
  21 ..                                                                                                                                                                                                                                                                                                                                             $                                                                                                                    IIIIIIIIIIIIIIIIIII                                                                                                                                                                                                                                                                                                                                                                                                                                                               ..
  22 ..                                                                                                                                                                                                                                                                                                                                             O                                                                                                                    DDDDDDDDDDDDDDDDDDD                                                                                                                                                                                                                                                                                                                                                                                                                                                               ..


>sp|P54253|ATX1_HUMAN Ataxin-1 OS=Homo sapiens OX=9606 GN=ATXN1 PE=1 SV=2
MKSNQERSNECLPPKKREIPATSRSSEEKAPTLPSDNHRVEGTAWLPGNPGGRGHGGGRH
GPAGTSVELGLQQGIGLHKALSTGLDYSPPSAPRSVPVATTLPAAYATPQPGTPVSPVQY
AHLPHTFQFIGSSQYSGTYASFIPSQLIPPTANPVTSAVASAAGATTPSQRSQLEAYSTL
LANMGSLSQTPGHKAEQQQQQQQQQQQQHQHQQQQQQQQQQQQQQHLSRAPGLITPGSPP
PAQQNQYVHISSSPQNTGRTASPPAIPVHLHPHQTMIPHTLTLGPPSQVVMQYADSGSHF
VPREATKKAESSRLQQAIQAKEVLNGEMEKSRRYGAPSSADLGLGKAGGKSVPHPYESRH
VVVHPSPSDYSSRDPSGVRASVMVLPNSNTPAADLEVQQATHREASPSTLNDKSGLHLGK
PGHRSYALSPHTVIQTTHSASEPLPVGLPATAFYAGTQPPVIGYLSGQQQAITYAGSLPQ
HLVIPGTQPLLIPVGSTDMEASGAAPAIVTSSPQFAAVPHTFVTTALPKSENFNPEALVT
QAAYPAMVQAQIHLPVVQSVASPAAAPPTLPPYFMKGSIIQLANGELKKVEDLKTEDFIQ
SAEISNDLKIDSSTVERIEDSHSPGVAVIQFAVGEHRAQVSVEVLVEYPFFVFGQGWSSC
CPERTSQLFDLPCSKLSVGDVCISLTLKNLKNGSVKKGQPVDPASVLLKHSKADGLAGSR
HRYAEQENGINQGSAQMLSENGELKFPEKMGLPAAPFLTKIEPSKPAATRKRRWSAPESR
KLEKSEDEPPLTLPKPSLIPQEVKICIEGRSNVGK

PFVM
     00000000000000000000000000000000000000000000000000000000000000000000000000000000000000000000000000011111111111111111111111111111111111111111111111111111111111111111111111111111111111111111111111111112222222222222222222222222222222222222222222222222222222222222222222222222222222222222222222222222222333333333333333333333333333333333333333333333333333333333333333333333333333333333333333333333333333344444444444444444444444444444444444444444444444444444444444444444444444444444444444444444444444444445555555555555555555555555555555555555555555555555555555555555555555555555555555555555555555555555555666666666666666666666666666666666666666666666666666666666666666666666666666666666666666666666666666677777777777777777777777777777777777777777777777777777777777777777777777777777777777777777777777777778888888888888888
     00000000011111111112222222222333333333344444444445555555555666666666677777777778888888888999999999900000000001111111111222222222233333333334444444444555555555566666666667777777777888888888899999999990000000000111111111122222222223333333333444444444455555555556666666666777777777788888888889999999999000000000011111111112222222222333333333344444444445555555555666666666677777777778888888888999999999900000000001111111111222222222233333333334444444444555555555566666666667777777777888888888899999999990000000000111111111122222222223333333333444444444455555555556666666666777777777788888888889999999999000000000011111111112222222222333333333344444444445555555555666666666677777777778888888888999999999900000000001111111111222222222233333333334444444444555555555566666666667777777777888888888899999999990000000000111111
     12345678901234567890123456789012345678901234567890123456789012345678901234567890123456789012345678901234567890123456789012345678901234567890123456789012345678901234567890123456789012345678901234567890123456789012345678901234567890123456789012345678901234567890123456789012345678901234567890123456789012345678901234567890123456789012345678901234567890123456789012345678901234567890123456789012345678901234567890123456789012345678901234567890123456789012345678901234567890123456789012345678901234567890123456789012345678901234567890123456789012345678901234567890123456789012345678901234567890123456789012345678901234567890123456789012345678901234567890123456789012345678901234567890123456789012345678901234567890123456789012345678901234567890123456789012345678901234567890123456789012345678901234567890123456789012345
     MKSNQERSNECLPPKKREIPATSRSSEEKAPTLPSDNHRVEGTAWLPGNPGGRGHGGGRHGPAGTSVELGLQQGIGLHKALSTGLDYSPPSAPRSVPVATTLPAAYATPQPGTPVSPVQYAHLPHTFQFIGSSQYSGTYASFIPSQLIPPTANPVTSAVASAAGATTPSQRSQLEAYSTLLANMGSLSQTPGHKAEQQQQQQQQQQQQHQHQQQQQQQQQQQQQQHLSRAPGLITPGSPPPAQQNQYVHISSSPQNTGRTASPPAIPVHLHPHQTMIPHTLTLGPPSQVVMQYADSGSHFVPREATKKAESSRLQQAIQAKEVLNGEMEKSRRYGAPSSADLGLGKAGGKSVPHPYESRHVVVHPSPSDYSSRDPSGVRASVMVLPNSNTPAADLEVQQATHREASPSTLNDKSGLHLGKPGHRSYALSPHTVIQTTHSASEPLPVGLPATAFYAGTQPPVIGYLSGQQQAITYAGSLPQHLVIPGTQPLLIPVGSTDMEASGAAPAIVTSSPQFAAVPHTFVTTALPKSENFNPEALVTQAAYPAMVQAQIHLPVVQSVASPAAAPPTLPPYFMKGSIIQLANGELKKVEDLKTEDFIQSAEISNDLKIDSSTVERIEDSHSPGVAVIQFAVGEHRAQVSVEVLVEYPFFVFGQGWSSCCPERTSQLFDLPCSKLSVGDVCISLTLKNLKNGSVKKGQPVDPASVLLKHSKADGLAGSRHRYAEQENGINQGSAQMLSENGELKFPEKMGLPAAPFLTKIEPSKPAATRKRRWSAPESRKLEKSEDEPPLTLPKPSLIPQEVKICIEGRSNVGK
Predicted Results:
   1 ..XZDDAAVAAPCSVAAASCVWACVWAASACPWCQVJVEBUPPLASPYJWPYJAPCCPAAQCYAJJARQBWYAYYEWWACADADJSSYCCJBWYAPCJDAAWCAAAVESCYCWSESJAESAACCDJJABVYEJBJUDSJJEJPSVAAYCSSPJSJEAAAAAAAABZWYAJWAAAAEAAAAAADPAAYAPPZYASAAAAAAAAAAAAAAAAAJAAAAAAAAAAAAAAAAVCSAWEWYAPCCCBSADEABBBAPJWPAAPAEBPYWVBEBEAWWJJJCCVABEERWCCAAWRBAAADBUPVSBCYWAAAAAYAAAAEAAAWAAAAAEZAAACAEDBCCSQZBDQDAAEQSPPEWPBYVAAPJEASWYJYJPVAVRJVQJBVABABCWYAAWWYWJABASADEVCBAABZVAVVPVAAVBBSCVABAAAAVVZEEEDCABAVBAPSWCQWPAAPRAAVAARSCSVASSQPDAAAYAAQYCWSYBABECWJBAAAABYACACAERQSACAPWEEESDVPEAASBEAEPACACSBVAJBVAAAAADEDJPVAAAAAADJSAAAAAAWYACPSYSECSQAPYJWABAAAQYCAAJYAAVAYAAAAAAAAAJVJWBBEVAWSVEVWRWYPCWBEBBEBEYPZASEALEEBAVEEJBBBABZJVJEWYJVAAPYDDVSWSBWARWYJYBWSBEWJAAAZQYPJWCWJSSBVAAAAAABAASASAASBVAAPAAAAASAVVVJEAAASZ$SCJSYSRDPSAACJYCAAJWWVCPYCWABASSCECCPJAEAAAPYAYWCSJWCYWAAACYSBSAAWWACYVY..
   2 .. J  SC  RW CBDJSPAYYWJYAVDYWSACRYS  ASVACASBSABVSCDBVVASJQ ZAJPWESEEPJPCAAJPDEJAVAVAVV SCCVVEWABBESASYDPJPVPWWCBCWY BAJJJSJ  R AEC SACARWEDD  AECPSBBYSYDVDDDWDDYSYJPEJ PSDEDAWVDJDDA SESDRJC SJ DJDDDDDDDDDDDDZDA DDDDDDDDDDDDD DYPVWJPCBJWWSS BD RB EAWBBCYP YBBJJB PWBEJW     JW SAPBWAY CBAS   CAP CAAE VAJDE DVEJSDADDDAVDDVJ$SJDP BV EPWYYAAZVQBCRYAYEBCS V  J AL EBSPJP APWYSZPAEAESBEEJWWBAPPAADVDPDA  EJDWEYASAWJAPCJEACPYDAJDDDWZV   B DJ AAJCCCSWJCCYAADEW J WSAWEAJWYA  DEVBJAAY VJBEWYSBWBVBBSVJRDWJ SAYWSVABAAAJA DADWWC DJBBWVWZJW PEBPBDDDJRJEV VDD   A CDDSP VCVWAWCSACSVAYAZABSEEWZCSBCWADPJJWVVEDDECDDEDPAJLRBEBVAJAEESBAEYSJBEEBABVCPPBWDBBAEEEYAYSWSWEWPCSBA P  DDAAABJPEYCCYEUABSBEWBCAVSWDYSCACYSWYBSZYBEDSSJPCYWWJJAPA  EDCY WYSA JVY DWAAQYBBCJW AQYCCVBAAJPPPYBPWAJSPAW    AWADPJDVJJAC CYYCPYWEVCJBAAJ     $SSAP..
   3 .. A  CJ   A YCB B B AJVCYSYEVJSBVVW  YWAHSE AYPCYVJQ WWQBS   WYBEBABYDWDWB$ Y JPBPSEB   VBJAPYJESSDBCWWESALC ZAPRJ    Y BBJ   D WWL DVQPJBBAP  W EAWWP YVCAJCVEEPQYSWCVZ  JBDBY JYVVB  CDA AVY WA SDVSSSSSSSSW PJJS SSSSSSSSSSWJ  JAWWDDSLCSCYBB J      RJVPSAJ CVSWCC BA WW       A DEBWCP  SC     JRE WDBL BED Y  RCDZED   BDVPDVAA BD EY QVYJVBWQPYWWASJBJSEL    P SB BCBYZ  PJYAWYAPARJ ECBBSCWY JJVJEWBYB  R SBQJBJSDZZDYAAY WS    VEAWY   E  S  DBAYA VYWRBDV BY R CWCYBRWAS    VEYVCSA CREABVAPACSELPEBS  S WJJJWES RWBYV  DPV     JVAYSYY  SCYDDB EAAAW   EB     RPVED EAAYVCJVCPYYV  A$RBADDDPPJEPVACDAEZPDBJCJPBWBWDAESWBEAYBJAVEAVYZAAVAYADCAAAJ BSEWBAWCABPEAAECCDW    C  JADJBAQBBWASBAEWJAEADRBBDDJAPPJWV$APEJCYCDDSDDAWBVYAWYJCY  B DZ YC   WS  LVYWCVJEDPC  C WSSPCJPDAJSSAJWBYDJC    JSBACWVBDBPP SVBECWPBWDDSDBS     BJJBA..
   4 .. Y  YV      ES   E VE$JVYEA  CPSBB  L  WRB   CSCJSP QRP B   PSASDDPS  WBSW   BWSQVSV    VPBA BBVABPVADJWB W LJSEP       A    E PAB E SWZ W A  Y RC Y  CZAPEVEDWVPJRSBR   PVBED D SE   DJB QY  B  BPPWWWWWWWWJ QPQW WWWWWWWWWWJP  WD CSABPV L W          RCEBJS ZJAESQ SC Y        V BJVCE   BV     PSJ YY W ZS  D  E  J B   DYBBWBUY E  VD A SVWWYBCEVPZAYAVAB       BJ W VCV  BYPEAC SDSY  FFCVY L SPYRP WW       YSWDJJARW BSV J     EVR B      C  VE  S YAASDED DA      BPEAPZ     D YPBP     LSBWLLRWPRSY   V DPABPSE BCJB   BC      CWY RVW   S YJE  YC B   JS     WBEBE BPJDSAYAJWWAP  PBAEJBEYZVPSVCBSBBBEYQS PDVEBAAPBWEAYYBBACYBBDPCAYSSJWJJWWYDR SBPSWWJBWSBCJEBJDA         JV  PR RZ  ESWPAEABAAESWJBYC$SESQJBCA P SBBEJE YCVVBWVSB  J  C QW   SE    DYAWPP WB  A PEAYJWB YLCCW BVPJEE    WAE DABSW ZD QACABRCRB PWVE      C  CC..
   5 ..            RJ   S JCAPDDPC  YL AC     D C   SA AV      Z   ZDLYVBAA  EVVB   A EDYCY    F DJ CRWEVRBV PBS B RFBFW              BCW X  YB        SD    PBBWYJJSBZJQWCSB     P   C D    EPR V   Y   W PPPPPPPPP VYF  PPPPPPPPPPP   CJ AJBR S J            SDCYVW SPDPWW CE S          WC JP   DJ       U Z    CJ  C  A  B     EQEE DQ      Q L ZCJCEESSPDCCCZBCL       CR D C    E ZD   CPCB  RS B  V VCDSS DC       CCY PAS V  JE L       S S      E   D  B EPSYSJ   B      EWWD B     P PEJC     J   CRWJWDWC     CYCPRCB FVSV   CS      YDB  AC     ZSW  SL     W        BW   VQVBJ CWL B    SEDWRBW$AWDEDEWWYDJEYJ J  JDSYYEVJCRDEVWSDAVJWJJPPAR DWAD QS ARWYLCDABCW  R W S         B   JA  E  SBZ DWSRLSDYCCPWD BSBBYC V B EJEBC  JPQCCPYY   A  D  D          JWJWR  V    SVP VBE BBVW  SEACVS     D  JBWCC VB   EWSVJZ   Y R         Y ..
   6 ..             W   R PDYACPBW  BY CY     E W   VP  A      D   DCSCWEJD  LSZ    P CYPR     A  S SSYRWYPY  C  R  LJYB              JZ  V            VW     CSJQPPBCECWEDJW     S     B    PCP $   J   C JJJJJJJJB  DZ  JJJJJJJJJJB    Q YRLA W S            EJWE D VCWSZ   P               AA    S               R  S  D  V      JCS WW            PYVWAWYEPBQJAR        R         Y      VRYP     C  P ZYBCA AV        BJ YCY J   Q S         D      V   W  E P YDVY          ACBB C       A VL         SVBVEEC      VUVSBYW SBZA   W       LYS  JP     V     S     B        SY   JZJPB BBS       P   R JCZBLSJDSDLBW C L  RJPZRPYWSSVPEEDJC V  $ RPD   PU  B Y YRSDBWVR     Y               WW     ACB ZJ  SCAC BDV  WBDJ   W W WL  E  SJCYZVP    D                D PZS  R     WF  E  DF A  C BW       E   PJWP BJ   VBY SY     D           ..
   7 ..                 W Z ZQP HO  ED WZ     Y F   BW  W          CPWR WWP    X      PJCW           D C EJB  D  Y  V                     A                    W IRSCVBDCJBYA     J     W    WRE S   D   V EEEEEEEEC  L   EEEEEEEEEEC       CYC                P SV Y  Y  B                   LB    W                  J  Z  Y       SJ  B            AVSVJACSWRPSS                           ZWD     J  J RSSBJ J            W   Q   S                  W   P  L B BJZ            DCR         C WZ          WC SW        VDVLDY YS             QEJ         W     W                   SCPWS WLJ       J     VWDRBWPJCSSPC W V  SPW SYACLA D YLBD    V  RW   SZ  C D JAPSLDSJ     P                P     P V     EDJE  S   AVJ    C   PW  U   W DVD     R                V  DW        DW     W  E             R   LE   JS   RV   S                 ..
   8 ..                 V     Z IB  RF        V         B           VFA JDR           WUZ            Y W DSD                              J                      V BYJYVPAPEF     Y     E     SW     V     VVVVVVVVS  S   VVVVVVVVVVS        SJ                   A    W                      RJ                       R     P       WR  C            C Z WBJYYV$WY                           V C     Z    B CVC                  Y   J                  A   S  D S DBC             F          $ I           DF CA        WEYA C C              VZV               B                    DBEE ZYR             Y AVDB BE   Z   D   S    SV D J  BZJ       J    YL  Q C  FCP S D     V                B     W J     R PW      DCY    H       W     PYU                      E  AY        BB     Z  Y                 SS    Y   L    C                 ..
   9 ..                       J JJ  WJ        Z         D           WRP P             JSQ                J E                              P                      W RZPW DPALJ     V     Y     Z      P     CCCCCCCCV  V   CCCCCCCCCCV        ZW                   J                            V                             D       JY  V            S P  VEZDWWC                            W V            P Y                                         L      F A  PE             J            P           Y  J         CPZD J D              W E               V                     CJV  PB             W   S  VR                PY J    YPP       L    D     F   RR J                        E     J         L       RPA    R             JPC                      R            P                           DP        W                      ..
  10 ..                       L LP   S                  O           QVZ               YEW                                                                          WVRC B E S     L                  R     YYYYYYYYE  W   YYYYYYYYYYE         V                                                                              E       LZ               Z D  Z BBEBV                            C W                                                        P                                                                 BCE   L                L               Y                     FY   D              S   F   P                C$       W        W    J     Q   VY R                        V     D                  DE                  FSE                                   Y                            C        J                      ..
  11 ..                         SR                      Q           BCV               QB                                                                              J U V                          C     BBBBBBBB       BBBBBBBBBB                                                                                         W       P                  Q  J VJFVL                            F Z                                                        Y                                                                 F F   W                P                                     SR   F              A   R   Y                JD                     V          V F                        Y     L                  QU                  LDS                                                                Y                               ..
  12 ..                         U                       Z           ZD                V                                                                                 V L                          E     QQQQQQQQ       QQQQQQQQQQ                                                                                         $                          R        Q                            J                                                                                                                            L V                    F                                      L   V                      F                                       Z          J I                        C                         P                   Q                                                                 L                               ..
  13 ..                         V                                    E                Z                                                                                   Q                          L     FFFFFFFF       FFFFFFFFFF                                                                                                                                                          L                                                                                                                            Z Y                                                           D                                                                             Q U                                                  R                                                                                     R                               ..
  14 ..                         W                                                     $                                                                                                              Z     ZZZZZZZZ       ZZZZZZZZZZ                                                                                                                                                          S                                                                                                                                                                                          F                                                                                                                                  Z                                                                                     Z                               ..
  15 ..                                                                                                                                                                                              Q     LLLLLLLL       LLLLLLLLLL                                                                                                                                                          U                                                                                                                                                                                          U                                                                                                                                                                                                                                                        ..
  16 ..                                                                                                                                                                                                                                                                                                                                                                                       Y                                                                                                                                                                                                                                                                                                                                                                                                                                                   ..


>sp|Q99700|ATX2_HUMAN Ataxin-2 OS=Homo sapiens OX=9606 GN=ATXN2 PE=1 SV=2
MRSAAAAPRSPAVATESRRFAAARWPGWRSLQRPARRSGRGGGGAAPGPYPSAAPPPPGP
GPPPSRQSSPPSASDCFGSNGNGGGAFRPGSRRLLGLGGPPRPFVVLLLPLASPGAPPAA
PTRASPLGARASPPRSGVSLARPAPGCPRPACEPVYGPLTMSLKPQQQQQQQQQQQQQQQ
QQQQQQQQPPPAAANVRKPGGSGLLASPAAAPSPSSSSVSSSSATAPSSVVAATSGGGRP
GLGRGRNSNKGLPQSTISFDGIYANMRMVHILTSVVGSKCEVQVKNGGIYEGVFKTYSPK
CDLVLDAAHEKSTESSSGPKREEIMESILFKCSDFVVVQFKDMDSSYAKRDAFTDSAISA
KVNGEHKEKDLEPWDAGELTANEELEALENDVSNGWDPNDMFRYNEENYGVVSTYDSSLS
SYTVPLERDNSEEFLKREARANQLAEEIESSAQYKARVALENDDRSEEEKYTAVQRNSSE
REGHSINTRENKYIPPGQRNREVISWGSGRQNSPRMGQPGSGSMPSRSTSHTSDFNPNSG
SDQRVVNGGVPWPSPCPSPSSRPPSRYQSGPNSLPPRAATPTRPPSRPPSRPSRPPSHPS
AHGSPAPVSTMPKRMSSEGPPRMSPKAQRHPRNHRVSAGRGSISSGLEFVSHNPPSEAAT
PPVARTSPSGGTWSSVVSGVPRLSPKTHRPRSPRQNSIGNTPSGPVLASPQAGIIPTEAV
AMPIPAASPTPASPASNRAVTPSSEAKDSRLQDQRQNSPAGNKENIKPNETSPSFSKAEN
KGISPVVSEHRKQIDDLKKFKNDFRLQPSSTSESMDQLLNKNREGEKSRDLIKDKIEPSA
KDSFIENSSSNCTSGSSKPNSPSISPSILSNTEHKRGPEVTSQGVQTSSPACKQEKDDKE
EKKDAAEQVRKSTLNPNAKEFNPRSFSQPKPSTTPTSPRPQAQPSPSMVGHQQPTPVYTQ
PVCFAPNMMYPVPVSPGVQPLYPIPMTPMPVNQAKTYRAVPNMPQQRQDQHHQSAMMHPA
SAAGPPIAATPPAYSTQYVAYSPQQFPNQPLVQHVPHYQSQHPHVYSPVIQGNARMMAPP
THAQPGLVSSSATQYGAHEQTHAMYACPKLPYNKETSPSFYFAISTGSLAQQYAHPNATL
HPHTPHPQPSATPTGQQQSQHGGSHPAPSPVQHHQHQAAQALHLASPQQQSAIYHAGLAP
TPPSMTPASNTQSPQNSFPAAQQTVFTIHPSHVQPAYTNPPHMAHVPQAHVQSGMVPSHP
TAHAPMMLMTTQPPGGPQAALAQSALQPIPVSTTAHFPYMTHPSVQAHHQQQL

PFVM
     00000000000000000000000000000000000000000000000000000000000000000000000000000000000000000000000000000000000000000000000000000000000000000000000000000000000000000000000000000000000000000000000000000000000000000000000000000000000000000000000000000000000000000000000000000000000000000000000000000000000000000000000000000000000000000000000000000000000000000000000000000000000000000000000000000000000000000000000000000000000000000000000000000000000000000000000000000000000000000000000000000000000000000000000000000000000000000000000000000000000000000000000000000000000000000000000000000000000000000000000000000000000000000000000000000000000000000000000000000000000000000000000000000000000000000000000000000000000000000000000000000000000000000000000000000000000000000000000000000000000000000000000000000000000000000000000000000000000000000000000000000000000000000000000000000000000000000000000000000000000000000000000000000000000000000000000000000000000000000000000000000000000000000000000000000000000000011111111111111111111111111111111111111111111111111111111111111111111111111111111111111111111111111111111111111111111111111111111111111111111111111111111111111111111111111111111111111111111111111111111111111111111111111111111111111111111111111111111111111111111111111111111111111111111111111111111111111111111111111
     00000000000000000000000000000000000000000000000000000000000000000000000000000000000000000000000000011111111111111111111111111111111111111111111111111111111111111111111111111111111111111111111111111112222222222222222222222222222222222222222222222222222222222222222222222222222222222222222222222222222333333333333333333333333333333333333333333333333333333333333333333333333333333333333333333333333333344444444444444444444444444444444444444444444444444444444444444444444444444444444444444444444444444445555555555555555555555555555555555555555555555555555555555555555555555555555555555555555555555555555666666666666666666666666666666666666666666666666666666666666666666666666666666666666666666666666666677777777777777777777777777777777777777777777777777777777777777777777777777777777777777777777777777778888888888888888888888888888888888888888888888888888888888888888888888888888888888888888888888888888999999999999999999999999999999999999999999999999999999999999999999999999999999999999999999999999999900000000000000000000000000000000000000000000000000000000000000000000000000000000000000000000000000001111111111111111111111111111111111111111111111111111111111111111111111111111111111111111111111111111222222222222222222222222222222222222222222222222222222222222222222222222222222222222222222222222222233333333333333
     00000000011111111112222222222333333333344444444445555555555666666666677777777778888888888999999999900000000001111111111222222222233333333334444444444555555555566666666667777777777888888888899999999990000000000111111111122222222223333333333444444444455555555556666666666777777777788888888889999999999000000000011111111112222222222333333333344444444445555555555666666666677777777778888888888999999999900000000001111111111222222222233333333334444444444555555555566666666667777777777888888888899999999990000000000111111111122222222223333333333444444444455555555556666666666777777777788888888889999999999000000000011111111112222222222333333333344444444445555555555666666666677777777778888888888999999999900000000001111111111222222222233333333334444444444555555555566666666667777777777888888888899999999990000000000111111111122222222223333333333444444444455555555556666666666777777777788888888889999999999000000000011111111112222222222333333333344444444445555555555666666666677777777778888888888999999999900000000001111111111222222222233333333334444444444555555555566666666667777777777888888888899999999990000000000111111111122222222223333333333444444444455555555556666666666777777777788888888889999999999000000000011111111112222222222333333333344444444445555555555666666666677777777778888888888999999999900000000001111
     12345678901234567890123456789012345678901234567890123456789012345678901234567890123456789012345678901234567890123456789012345678901234567890123456789012345678901234567890123456789012345678901234567890123456789012345678901234567890123456789012345678901234567890123456789012345678901234567890123456789012345678901234567890123456789012345678901234567890123456789012345678901234567890123456789012345678901234567890123456789012345678901234567890123456789012345678901234567890123456789012345678901234567890123456789012345678901234567890123456789012345678901234567890123456789012345678901234567890123456789012345678901234567890123456789012345678901234567890123456789012345678901234567890123456789012345678901234567890123456789012345678901234567890123456789012345678901234567890123456789012345678901234567890123456789012345678901234567890123456789012345678901234567890123456789012345678901234567890123456789012345678901234567890123456789012345678901234567890123456789012345678901234567890123456789012345678901234567890123456789012345678901234567890123456789012345678901234567890123456789012345678901234567890123456789012345678901234567890123456789012345678901234567890123456789012345678901234567890123456789012345678901234567890123456789012345678901234567890123456789012345678901234567890123456789012345678901234567890123
     MRSAAAAPRSPAVATESRRFAAARWPGWRSLQRPARRSGRGGGGAAPGPYPSAAPPPPGPGPPPSRQSSPPSASDCFGSNGNGGGAFRPGSRRLLGLGGPPRPFVVLLLPLASPGAPPAAPTRASPLGARASPPRSGVSLARPAPGCPRPACEPVYGPLTMSLKPQQQQQQQQQQQQQQQQQQQQQQQPPPAAANVRKPGGSGLLASPAAAPSPSSSSVSSSSATAPSSVVAATSGGGRPGLGRGRNSNKGLPQSTISFDGIYANMRMVHILTSVVGSKCEVQVKNGGIYEGVFKTYSPKCDLVLDAAHEKSTESSSGPKREEIMESILFKCSDFVVVQFKDMDSSYAKRDAFTDSAISAKVNGEHKEKDLEPWDAGELTANEELEALENDVSNGWDPNDMFRYNEENYGVVSTYDSSLSSYTVPLERDNSEEFLKREARANQLAEEIESSAQYKARVALENDDRSEEEKYTAVQRNSSEREGHSINTRENKYIPPGQRNREVISWGSGRQNSPRMGQPGSGSMPSRSTSHTSDFNPNSGSDQRVVNGGVPWPSPCPSPSSRPPSRYQSGPNSLPPRAATPTRPPSRPPSRPSRPPSHPSAHGSPAPVSTMPKRMSSEGPPRMSPKAQRHPRNHRVSAGRGSISSGLEFVSHNPPSEAATPPVARTSPSGGTWSSVVSGVPRLSPKTHRPRSPRQNSIGNTPSGPVLASPQAGIIPTEAVAMPIPAASPTPASPASNRAVTPSSEAKDSRLQDQRQNSPAGNKENIKPNETSPSFSKAENKGISPVVSEHRKQIDDLKKFKNDFRLQPSSTSESMDQLLNKNREGEKSRDLIKDKIEPSAKDSFIENSSSNCTSGSSKPNSPSISPSILSNTEHKRGPEVTSQGVQTSSPACKQEKDDKEEKKDAAEQVRKSTLNPNAKEFNPRSFSQPKPSTTPTSPRPQAQPSPSMVGHQQPTPVYTQPVCFAPNMMYPVPVSPGVQPLYPIPMTPMPVNQAKTYRAVPNMPQQRQDQHHQSAMMHPASAAGPPIAATPPAYSTQYVAYSPQQFPNQPLVQHVPHYQSQHPHVYSPVIQGNARMMAPPTHAQPGLVSSSATQYGAHEQTHAMYACPKLPYNKETSPSFYFAISTGSLAQQYAHPNATLHPHTPHPQPSATPTGQQQSQHGGSHPAPSPVQHHQHQAAQALHLASPQQQSAIYHAGLAPTPPSMTPASNTQSPQNSFPAAQQTVFTIHPSHVQPAYTNPPHMAHVPQAHVQSGMVPSHPTAHAPMMLMTTQPPGGPQAALAQSALQPIPVSTTAHFPYMTHPSVQAHHQQQL
Predicted Results:
   1 ..AAAACCCWYAAABAJBALAAAAJJWJAWAVCJBJDABCJPSPYJYWSJBVJWCCCCPYWCCYAWAACCCJYDEASBZACVZYABPWVWJYAAWAWVPYSBSEEDABAAECPYVPCYVCSCAWBVYABCAYCSAEBSAAJJCCYDJRBVDDPDECAYADWCWASBAAAAAAAAAAAAAAAAAAAAAPBCCWYAAAVCVACCBAAACYACCAPCJVVSBSAVJYJVYYBADDDVAPJVSCADVAQEWUUYAWSVDWEEAYSAABDDDDDAEBAABWPDBJJEBARWYCZDDYRAAEDPYCAADAWAAAAPPAJAADVWS$YAAAAAAAAECCADEEEVEWACYAAASEYAAEJAJAJAAEEW$YZBADBAADZAAZSAAASWAAAAAAZJJPDAABBVAAEEEBBYAYJCBEACYBYAEAAEBACYJVYSYVAAABAAAAAAAAAAAAUDAEADSPAAADZJJYYYYAAABLEAAACYYAQABBSSUPYASADBWYAJJDPAEAEEWDWJJAVCCWYCYAPYBLEJYJEPVWAABPVAYYPJQEDESQCJAVVVVSVBCAPCCVAADAJVYYBWCYCVAWZCWCVRCCVRJCACCRVAVWWCAYVCSWSEJCCCBVAAPCCEPBSAAYDJVVVAJYAVAJPREBJYAAADBJPCCAZABECSSAABLYQAPRVVAADVPWACPCVBAAPECCSAVJDCJJWBVPSBBACSAWYYWVYAAAWWWWVAAJSPCVJVAJJEJVWJBYAAAJAAAAAAADAWCJAPAAAAWWASAJPDBVAYASACEBCSECEVAAAAAAAAAWAWPAABWVAASAAJVAAAAJYPPQAAAAPAAADAAAWYCYJYVDAWJSVYSABZEJVJCJJSAWSYAAAAZVAAAPVYASYAYAARPSBVAVAEAVAYAJBBAAAAAAAEBAJVJVJBWDESBAABPPWVSWAYCBPBWSDAJSSVAAJAAAASWSEEWPYBSAWVVVVVSCSWCSYYACAPSPCYPPCLVAAAARAACWVJCSSDDAAAAACJAAALSAAAAPCCWWAECYAAAVVBAEJWYADJVAPCABADYSVVADJPPAJEEBACWPCAAAAACZAAJBVPAAAAJLBDEJJSVSAAAAAAAAPPBWBVDAJPAVEBA$AYYBAAAAAYWVJJAPSYVJVCCSSADWBQSBEPPPASJPPVCJWZSSSSSQAAAARAAAVCYABVABJDWYAJPWFYCAJWCAPJVPPWDJWYDAVAJADLRWSYVJAWSADEAWBVVWAPFCAREEAYBECWPYAJAAASSAEIIWCYAWAWAAAAAADBACWCCAEAAAYAAAAWYAAWAAAYB..
   2 .. DJJSBACCDWDWSAJBAEDDB  CCL PYPVCAAJCWPJYCCPRPRCWWPCWWZYWS JWSW VPSPSPZY  J YWSJAVDDJLYARAPDQDAYC V YJAABAPBVDJVAWWAYSBAJACYAQAWE   EQAJWJBPWW AP CD  WSAUEZDAE CP  DDDDDDDDDDDDDDDDDDDDJBVWSSASSD VYJZACVDJPVWAPCWVAAAEJAPPPAAPCWPPAAEAWWSBYJ$PWC WJ  PCPCYYJABWPZZED     ECAVWVAAAJ   RYAPCPJASCAWJBJCBSBSJEADDYC  CAJWYACJQWRDD D DE   D JBWBRADBV  JAAVPRAARBVAWWAJBPCBA JEBPWC P ASEDB YQDDDDLPSAA V WY JA  SCVJAPAEWWABSVPVCYAEYSWVA $ YEDJADDEDCDDJCDDDWASVYAB DWVALPAVVVZWEDJBBBDVAJBYDEALC BJVC V ECSD BASDAVWVSJP  JW JPFJWPWC R AVAAJES P BSDCZ ASWEW$WJB     W SVCZPWSWDAWP SAYPWCDSVPS CPSESWSECVVSW D Y  EWSYWBYWVPW  WAZEC  BAECJDA     J BWASPWSLWVWBBBVAAWBLCRSJWJCWJJWSZ$SCFB WEEJAZDAFSZ   CS SVD WVUYYEYWCCJECJY BVJA VVEDE   YVEPVWYSPCPCAJDEBDWCDDWWDDCYDJ AJPVVDSVJSJBZPAVPYAWAWADDYSRQBBAW AD BDDJVDDBJB SPCECYZWJ C RDJDAVQCASPVDAYVJAEDJYEAVAAAAJVBYCP J  CSSPVAWVJLAADDDCYPP BQWSDE DQSDCWEVZY  LP BSPDJSJJYBUDDDEP ACYAAAAABWCJABCSBV CJSECFC  CCVB C      ECBBBELB  E      PJCBBYWBWS SC  L  W YSDWBJDLS  S           P DS VPPDVWW BAQWWVY    JBVE Z AYW VVWAY  C  D     EABS DYB  DD  W   PSYACBDBPEECJ  AA          AEPSR JPYJEAABPBWJCVDDD  B ABDR V BWS BWYACCWPJB   Q  Y WPYCS       DDDADDDAJVZEAJJEABADAABCA D CZD$AAVWCCC DACPDE EAB BV     JS SB   L BVWDA  QSSB YAVVY     DB     JPYAYDDDDDAJW BSBEA  D      VWJL    A..
   3 .. JBWBYWS PLWA SPVED      AW   S  YPSWASWAYAWSCYW BBJSVW YC D WJ   J VSCA    JPVWPABA C CBVJSAYZAW C ZWBBDEBRYPCSY ZCAJCPESJ QYDJV   Z CPSCCDES  C E   AA PDC EA S   WSSSSSSSSSSSSSSSSSSSPEC YAJQD  ECYPYAJPEJAYVAPCSCBJAWWYCWBPAVA VWBWBD$QPPYZBAS PS  AYCBZPEDRBRCYV       ADPCZEIJ    AWCQSYBBBABB DSJ    ABVWV D    DDAWPAYCD     J      ARB BV A   EPSSC  WPAYSECDADQSJE AAJBAY S VDSEZ VDP W VACVY   S       A   VYABDB  ADZVBWSWRECJ Z AD SDWWDBDYSDDVS EBBWDQ  BBDBVYWZA AVDE JASE  ZASAQSJJ ESCE D A J  WC VWBBWZ Y  SB   CPVDSW E  WCCAYJ   W YBA VZA AWCSC     B  SWASSC S  V ZBSY SAWPJA SFCSPSCS JSPS   C  VC WBCBB      JDW     WYV B       VCQCSYEVSCSJDEWWB SSYADSJWAB  ARVCJBDY  DCBJBVSVWY   B       ZEAVSSJWADASPC PRAP CJDSV   CCDCBBVBASQPEVBJLSYVVVDCVCWDCS YS SSYVSVDWEB JYWV ACCV YPBBESDBA WZ CBCDD JEPS CEEPWVPRV   D SVCAWS PB JJS EWJP SCBZDPBYD PASB    PPYWRSCWBAC WVV$S D C CCCB WP VSBJEYD  BB  JDAAAWCVJEB CAB WSJDYCJSE  PDY  EC EPYVW L  S CC W      BLCVJPCE  B      RPBCWBAPBA LR  W  E A  QDE D               D PW BDDPCAJ DCEJZDB    EDYV    C  BSDDV  V        PBCV EAV  CW  L      YDJBCWAAAB   D          SC  S SWV L  EVDDAPY        WSY   P   JCSPJYPY S      R S WS         PYBVSVCPAD D PA VEEPJSLC   EYVQ P CVAW ZSVCPA  EC C      DE V      JYY E    E   B  A      I     PBCBCELJQPPSC CWECV  E      Z D      ..
   4 .. PDCWWPL  SVD   D V      Y    J  SQPJJAYVZSCB CS LSP BJ BV           CSC    AJYABCCS P BSWDEESPSY W  ADSECWVZVS J BJPPRSB W SDWPW     PBDWAYSA  W L      $ A  C Y   VWWWWWWWWWWWWWWWWWWWWWY VVDWC  P WWZDSJSAJDSJWYYVJZRLCCSESSCSC WESAEVUPWCVQVYP VB  JSYWP V   VPE        BSWDDLC     EDS$ASS  SVE S       DBJB      PJBEAWPAB            WAC AB      V  D  DJEJRDDWBAC    YVSS     WJBWW D J B BCVCV           D    DJR J  JQADC JZWBYC V  S DVYCJYP VCJWJ VLRYE   YDSEBQ B  VBSB  R B  Q J Y SB AVQS        SS W DASA V       PWZWJE W  SSQEZC   J ZSJ YAB DVZWW     C  YSJBPB Y  W CWW  ZYJCCC AJB WPB  SWWP   B   B CAAAL       PJ     PJB         ABCWEE PAABSESA P W VYVWCVBJ  PJSYUJ    SJWSDSWJBW            WWA  E FADBBV  AES BYB A   SDLBACZYBYC P APJBVZPYBSJ JVJ      Z BZDJS V Y    VDZB JVA W AUB BW DW Y  SJSP W WJYSSAY     DPBPSV CD P P WBYV CBSBSVWB  AVAA    WY LB BARJB SC BA      A$W PR BAVPW V  DC  PCPDWDDSDW  SWC PBPEJDC J  BVC  WW BWRSJ E  W $W        W RA  W           SESAJJJC  BF     S     EA W               A WE   LVPCP CBJCVSC    A          W V C            JWW AVW      S      DVDEWEPDJR              Y   Y     Y   YJSSAC        P A       CACVSVA  C          SP         RZUSJW BZJ   WS AQWYBEJ      C  D  BQ   VWA    WA                           W   J  Z            U ZYWVQQVVYA  PBWSB           Y      ..
   5 .. VWDJE B  DL    S J           W   YDVBVBBAVVC J  SAQ  P  A           WPJ    VC ECUJW B L SBYPBVJ  Y  BR  SEESAB W  BCA WH P BJPA      YYEBDAB   S S      V J    B   PPPPPPPPPPPPPPPPPPPPB   WYPY   W ZYPPDBC QVBRYSJPWPDYDVZAJYJWB JLJSJSAWYJSDACJ  V  QJA   A   WVV        DCDEWV      DVPA AC    J A       PSVW       IPBYCWVV            LSV  J      W     ECPDPJVUDPW     YVC     YVL A Z V S A YD            J     RS Y  PSPLD   BABP Y  R PE BP W WQW   YSV V   EJBVAW    WDCV  S    $ Q B  R  Y          VB B JCBC E           AA C   PSBA      JVP  B  BAPPA        JJBWJJ    A RZ   VEPWE  B J VJJ  BBVJ   W   P PJ V        YQ     LZS         CSJYLC ULPC RQJ S   W JE P     FJAYW    RSARCC WAB            DBP  B BESREZ  EBC WCV Y   WSJACLSASBJ B CBAWJWB  AY D$S           VP C      C J  W P L JCJ S          CY E ABSBVCP       S AW Y    D DSBB VRWC JDZ  VDBE    AC SE PYPCW  B DE      BSA YS Z DBC    FV  A  SJQVJEH  WVJ Y DBZVS    E    CY JCJLV P  J Q                           WRLSVSAE  EL     B        B               Q  L   VZ  V AEB L J                   D            R   B        Y      JSCSDAV VP              C              CJBDD        S S       P D PZJ  J          CY          JE BC E      R   YWCCV      Y  Y  JP    BD    B                            V   W               Z DSJ P PJ C  S SVD           E      ..
   6 .. YSBPS    VS    W B               ZBYYBCJWBQJ     EV  S  W           BWV    WQ BJJPC F   BWBBEDC  E      WJDBEA    ZB   I   DZVQ      DEBDQWP   R W        S         JJJJJJJJJJJJJJJJJJJC   BCSV     QSJSZYD ZJPWJZWSSYBAREADVB RE BBCBPYBYZW YY Y  A  VVB   S    J         R EYYJ      SBVS  R    Z C       CDYJ       QJSV JZE            SW   E      D     LYSWBLBJ JJ     DYJ     CZD V   E J D  Y                  SJ S  RWDBJ   AJPY    B     B    SQ   CWW     SLWLDD     J W       C Z J  F  A          EP   EDUJ L           CP F   BY        VA   D  PD$A         WYSEVW    B E    WW SA  P W YVW  W YV       S RF          EB                 DJWD A JCYV SC  Y   B  P S     WCPD     JB$BJE CJ             PSB  S ESJVW   SZF   W     BYBWWSWCVZY W  DPCC Y   P YC                      Y P    W   CJS J          DA     BJJVC       V BY J      YVDE  VJ   Y    PEJ    BR ZW E WFE    PV      VER  V   EVP        B  CV WPYV         WE      S    J  P W      B W                            YVJDVS    V     L                        V      WE      D S                                  S            Z       YEVVD  E                              PARJW        A         D W YAU  P                      SD    W           DW P            PW    PY                                 R                   C SLD B BC W  L VBJ                  ..
   7 .. ZPVVV    YE    P C                 PSESPQP       V                  E       $ CWSYZ E   E CJJJE  Q      LDJWQW    VS   S   P QS      EDVFVC                         EEEEEEEEEEEEEEEEEEEE    B D     $BSYBEP CPWSRBBWCSJEEDYCEE A  D EJLJDEJB W  Z  R             Y         S BPAR      W ZJ  A              LEPS        ZYB E S            YJ   L            VSRRD EL VS     JZY     DRW Y   W P J                     YC E  BJSWW   EVU     V     R     Y   S Y     VEYWJA     P L         P W  W  B          AW   PYR  S           VB B   WZ         J   $  VJYY         BBW YA    C B    B  J     A BYA  Y BY       D LL           S                 WDBP L YVDJ  R  R               WZP     BQUEAW RP             VCS  A RWWE    D B   Y     DJCS V  WD  C  QSZA       VZ                      S Y    Y   EDE P          WC     R YYB       Y YJ $       E J   V        WW     JZ BY          ZJ      DJD  A   AC         D  EY SDVD          S      W    L  S          Z                            AYLAB                                           CB      W                                    W                     VCYC                                 EELZZ                  W    D                                            BE              S      J                                 A                   S PV    SW L     DY                  ..
   8 ..  CL A    E     C W                 AZLVCSW       W                  Y         PVBWL J     P$LYP          LYC      DD   V     YY      JWCEW                          VVVVVVVVVVVVVVVVVVV     J B     VJBJLCV DBEBSVPYPEWVBJJRPU F  Z R WPVA R B  K  U                         SVEU            D              FCB         QJJ S Y            P                  V  W    L      PD      PYJ     B E S                      V P  DBJSE   YC            L     B     D     WPE SB     $ J           S     Q          CJ   S Y  A           Z      E          U       PEB         PVQ LF    Y D    J  Y     F  LF  A  L       E ZS           Y                 E EV J APQE  W  W               B       WRE PB A               E   C JV W    B L          WPE Y  CW  Q   VV        Z                       Z S        IS  Y           Q       CEW       P CA V       J     E         Y     LB C            B      WQP  C    L            $  YQWI          V           R  W                                       VFVCC                                           YY      P                                                          WYJR                                 WBWVP                  Y    S                                            C                                                        J                   V VE    YE        W                  ..
   9 ..  V             J                   EVWADDJ                          D         QSHZY       $D BB           SJ      EE   Y     ZB      OA L                           CCCCCCCCCCCCCCCCCCC     Z       CRLVWFR  CJFVFRDYRLDJ BYWC B  Y V D YV V S  Q                             JR             E              JLS         VCE V              B                  W  Z           W$      QCP       C Y                      A    ERLYV    S            S     P     P       J YV     Q R           D     W          PZ        B                  Y          E                   RPC AL    S W    P  B     L  AL  R  A       Q B                              J Y    DESA                     L       LW  V                      Y Y  Y    C R           RV    YJ  R   CP                                  V        PX  C           V       BJZ       W J  W       C     P         J     SV E            P       WY  W    W            P  B$SJ          W           S  V                                       JJ W                                            EW                                                                  JPY                                  PPW                   Z                                                 E                                                        Y                     WP              L                  ..
  10 ..  E             U                   LPCRQJ                           V         SD S        ZS QL           CD      LL         SV      WF S                           YYYYYYYYYYYYYYYYYYY             P DZE B  FYL J EE PSV EVFV E    F C JC F    W                              J                            V C         WUS                R                                  C      Z C       R                        $    Z BJ     U                        C          S     Z U           P                L         C                  $          R                    ZE ER      Q    A        R  ER  Z  E         J                              Z S    W  P                     R        Y  F                      R L                     S      R  Y   RY                                           VY  Q           D       D E         V          R     U         P     VE R            D       R        Y            W  PZ B          H              Y                                       WR $                                            ZC                                                                  WSV                                  CC                                                                      Q                                                                               Q                                 ..
  11 ..  Y                                 I$REZE                           Z         YR E         V S                     Z         CE       C V                           BBBBBBBBBBBBBBBBBBB             S WWP W  SR    BD YPF QZUW J      Y QR L                                   P                            W            LL                                                   P        R       Y                        D      C                               E          Z                   R                R         J                  D          W                     V R       $                R      R         Q                                     B  L                     A        A  R                      P W                     V      E  I    A                                           Y   R           J       E S                    Y               R     R               W       B                        Z             P                                                      D  Z                                            JJ                                                                  RZZ                                  QV                                                                                                                                                                                        ..
  12 ..  $                                 R YZ$B                                     ZY           L                                 JL       L P                           QQQQQQQQQQQQQQQQQQQ                $Q Y   L    ZL CCL WS          O  X                                     S                            Q            PF                                                            Y                                F      R                               J                                                                            Q          P                     Y         P                                 S                                     X  R                     E           S                      I                       W      F  S    R                                               Z                   W                                      U     Y                       P                        R                                                                    F                                               $                                                                     S                                  V                                                                                                                                                                                         ..
  13 ..  Q                                 S  $WV                                     R                                              ER       V Y                           FFFFFFFFFFFFFFFFFFF                LY     D     R  FP $B          S  B                                                                               QI                                                                                                    Y                               Q                                                                            V          I                                                                                                       R  U                     P                                  Q                              A  V                                                                                                                     Z                                                                                                                     L                                                                                                                     B                                  R                                                                                                                                                                                         ..
  14 ..  Z                                 Z  U                                       U                                              $$                                     ZZZZZZZZZZZZZZZZZZZ                 C     F        O  D           Z  F                                                                               FR                                                                                                    W                                                                                                                       L                                                                                                       S  I                     Q                                                                                                                                                                                          $                                                                                                                                                                                                                                                                                                                                                                                                                                                                        ..
  15 ..                                                                                                                              RF                                     LLLLLLLLLLLLLLLLLLL                 R     U        Q  R              U                                                                               H                                                                                                     Q                                                                                                                                                                                                                                  O                                                                                                                                                                                                                F                                                                                                                                                                                                                                                                                                                                                                                                                                                                        ..
  16 ..                                                                                                                               I                                                                                                                                                                                          I                                                                                                                                                                                                                                                                                                                                        Z                                                                                                                                                                                                                Q                                                                                                                                                                                                                                                                                                                                                                                                                                                                        ..
  17 ..                                                                                                                               X                                                                                                                                                                                          R                                                                                                                                                                                                                                                                                                                                        D                                                                                                                                                                                                                                                                                                                                                                                                                                                                                                                                                                                                                                                                                         ..
  18 ..                                                                                                                               Z                                                                                                                                                                                                                                                                                                                                                                                                                                                                                                                                   F                                                                                                                                                                                                                                                                                                                                                                                                                                                                                                                                                                                                                                                                                         ..
  19 ..                                                                                                                                                                                                                                                                                                                                                                                                                                                                                                                                                                                                                                                                   Q                                                                                                                                                                                                                                                                                                                                                                                                                                                                                                                                                                                                                                                                                         ..


>sp|P54252|ATX3_HUMAN Ataxin-3 OS=Homo sapiens OX=9606 GN=ATXN3 PE=1 SV=5
MESIFHEKQEGSLCAQHCLNNLLQGEYFSPVELSSIAHQLDEEERMRMAEGGVTSEDYRT
FLQQPSGNMDDSGFFSIQVISNALKVWGLELILFNSPEYQRLRIDPINERSFICNYKEHW
FTVRKLGKQWFNLNSLLTGPELISDTYLALFLAQLQQEGYSIFVVKGDLPDCEADQLLQM
IRVQQMHRPKLIGEELAQLKEQRVHKTDLERVLEANDGSGMLDEDEEDLQRALALSRQEI
DMEDEEADLRRAIQLSMQGSSRNISQDMTQTSGTNLTSEELRKRREAYFEKQQQKQQQQQ
QQQQQGDLSGQSSHPCERPATSSGALGSDLGDAMSEEDMLQAAVTMSLETVRNDLKTEGK
K

PFVM
     0000000000000000000000000000000000000000000000000000000000000000000000000000000000000000000000000001111111111111111111111111111111111111111111111111111111111111111111111111111111111111111111111111111222222222222222222222222222222222222222222222222222222222222222222222222222222222222222222222222222233333333333333333333333333333333333333333333333333333333333333
     0000000001111111111222222222233333333334444444444555555555566666666667777777777888888888899999999990000000000111111111122222222223333333333444444444455555555556666666666777777777788888888889999999999000000000011111111112222222222333333333344444444445555555555666666666677777777778888888888999999999900000000001111111111222222222233333333334444444444555555555566
     1234567890123456789012345678901234567890123456789012345678901234567890123456789012345678901234567890123456789012345678901234567890123456789012345678901234567890123456789012345678901234567890123456789012345678901234567890123456789012345678901234567890123456789012345678901234567890123456789012345678901234567890123456789012345678901234567890123456789012345678901
     MESIFHEKQEGSLCAQHCLNNLLQGEYFSPVELSSIAHQLDEEERMRMAEGGVTSEDYRTFLQQPSGNMDDSGFFSIQVISNALKVWGLELILFNSPEYQRLRIDPINERSFICNYKEHWFTVRKLGKQWFNLNSLLTGPELISDTYLALFLAQLQQEGYSIFVVKGDLPDCEADQLLQMIRVQQMHRPKLIGEELAQLKEQRVHKTDLERVLEANDGSGMLDEDEEDLQRALALSRQEIDMEDEEADLRRAIQLSMQGSSRNISQDMTQTSGTNLTSEELRKRREAYFEKQQQKQQQQQQQQQQGDLSGQSSHPCERPATSSGALGSDLGDAMSEEDMLQAAVTMSLETVRNDLKTEGKK
Predicted Results:
   1 ..JWPBBEBVVYJVAAADDDADJVPSVJWYSAAAAAAAAAADDAADAADDQSBWYAAADDAAAPYQWCCCYPYVECEAEABAAAAAQYJAEBSVSPYAYAAJBBSVAAJVSEEEEWZAJEEBEASW$ZPREEVAAADVPAVAWWZDAADAAAAAAAAQSBSBEBBVYSWSEWYAAAAAAABAWAAAAABSEAAPAAAAAAAABDAEECAAAAABAWWYAVDAJZAVAAAAAAAAAAAAEJBBVCVAPAAAAAAAAADAVAEBAAWCAWBWJWAYADWZAAAARABAAAAARAA$AAAAAAAAADJAADVYASBVRVAJSDVAQAPPAAADLAZACYAAAAAAAEBBCVAAADCAAAJYS..
   2 ..SVSSVBEQPVAPYDDAAADAAYDYAPSZADBDDDDDJDDAADDADDAA$LVAZDJDAADDJWSAJQEJVBSWWWYEADDDDDDD SPEAWBAAJVDADEBSWBYWWPWJDAAB $D BBEBWAVJYJSBBBCYDJECSASABYADEADDDDDDDDPCWEEBEWCPJBCSAZ DDDQYJAJA     WVBDECDDDDCDJB B  BAPWDDDASAQZJE VSVSADDDDDDDCDDZBABVJAQADWBDDDDDDSDAJAYAYBVA   E BAVBSBAYDDDDADJJED  SB AVDDDDDDDDAAYDAASB V  YJ  WWEVWDAYYDYWDWPSZDDVDDDEBWASADBDAACVWEV$..
   3 ..AABEWJSWJASAV    JYVQWJBDDCVCBSBJE  D SYQ  WB  QZAJPEYDE ZPPQJCVBBYSZQAJLSVVBEAV J   EBBDADDVCCBD BQEEW BDACPWDB  AZ  LRVERQCDAEWREWDJAWWCCDBCDYWDEEEJB EVP ASRJW EWAACLRJB WJWBPCVWY     CWABYDSWVJDW   J  LS BSEWCC JVQA EAJJYPPBCJSEDYCVEDASPQYPSASVYPJ BDPJESWJADWC     VPLCDJCAECJBSYAPD   A  J  JSSSSSSJBDVVPVJ    J   SJWWCADPDCAEZP VVW$D EBDCJ J BD WDPYEAJA..
   4 .. BAREPWBYBDB       JBASWEVJDDEEVWY  S YZL  CE  SAJYCVJV   VYDCBWPESWAYJCJ BBDSS  B     VBEEBEB SQ RARPC D YPAB    YY    JBDDY BWLUAPJPSC$YBBJAJ LV BBV  JBV YPA R AYCRPRA E B  SESWYC      BDJPYV  BW    Q  PB DJBEW  PCPD  BADJWB  VVBBRSYDJD VPPZJJDBVJS P EBBPCWP D      EVORRAEBYYSWDVWCV   C     SWWWWWWVSSBJQAS        VSCADBJWJYBYCA PJ    BEBA    EE V$ PBBQV..
   5 .. DEVAWVYBCLD        EQBVQSP BRWJC     BBJ  BR  JSBAJJS    CSPBVCAPPACJEFF   JWV  C     RSDU C     DEVVV V CY L    C     ADBA      DDZCPUZJWJDVV  S VSS  WSW  YB S DBVBFWB      DZBYCE      LQSCAJ  E     E  WJ VWZSV  AQWP  WYWSZC  C JWWWCWPS  YAJVCYJ C  J  EV JV         YY PJ LSW   BPDS    D     PPPPPPPP PJBWB         BBPYSJWBZBCAPD B     J RD    PJ  V SDPCP..
   6 .. PJWJQJPCPV         PPY   B PSD E     PWY  ES  BDCWBW        SWBSJRRWACAB     C  P     WW   J     SPJJP J  D A           VC       IBCEBBYZLEED   J RCW  CY   AU    SWCEE       P  DSP      ERVSJC        W   Y JCWBP  Z$Y   CBCWSV  Y CSBBD QW   BY Q      Q  QP PY         A  $  SVB   CBS     J     QJJJJJJW WLCSD         JYYPVVYVBVEC $ W     W SV     P  P DLDPY..
   7 .. E CLSAEAJY         DCC     W         JPV  S   PIEPVB        VESCWWPJZLBD              D    Y      SW   S                LP       WVVBQDARSPVS   R      V    FI    AL A           S V      A WWFY        A   D EB VD  $PZ   DPPCJ     RJJEJ V    ZS B          S B          P  A   PS   EC             EEEEEE  $WR J         L VJJZBCPJJR Q       P J      W     S  Z..
   8 ..   JPRQSWQ          RDW                V   J   VYWCYD        YJPVYAVD$PPR                               C                           W R QDJWY           B          ES L             B      Y PJ P            V SL CE  Y     REEBQ     WEE L      DC            H R          S  V   WJ   J              VVVVVV  B             Y SSPCCSVZPP Y                          ..
   9 ..   UYDC  W          VL                 S   L   EJPRDP        ARJYSBBQWBYS                                                           B W JBE U           F          UE               J        QD Z            W     J        EDVEY     PRS        SD            Q V          W      CC   V              CCCCCC  C             E FDBYQJCEWV B                          ..
  10 ..     VF                                J       WPVSE$          YEVFY CVD                                                              E   F L           P          PJ               S         B                    S        VWBDB     S          V             R                    L   W              YYYYYY  J               Z E ELQS F C                          ..
  11 ..      P                                L       $BYD A           R LD VWR                                                                    P           R          DB                         V                              CLPC                              U                    V                  BBBBBB                    Y S SW D J                          ..
  12 ..      R                                         HFE C             V  S S                                                                                S          J                                                         SY                                                     P                  QQQQQQ                    $        V                          ..
  13 ..      U                                         C F S                                                                                                              R                                                                                                                                   FFFFFF                    I                                   ..
  14 ..      Y                                         E   U                                                                                                                                                                                                                                                  ZZZZZZ                    L                                   ..
  15 ..                                                W                                                                                                                                                                                                                                                      LLLLLL                    F                                   ..
  16 ..                                                                                                                                                                                                                                                                                                                                 O                                   ..
  17 ..                                                                                                                                                                                                                                                                                                                                 R                                   ..
  18 ..                                                                                                                                                                                                                                                                                                                                 U                                   ..
  19 ..                                                                                                                                                                                                                                                                                                                                 Z                                   ..


>sp|O00555|CAC1A_HUMAN Voltage-dependent P/Q-type calcium channel subunit alpha-1A OS=Homo sapiens OX=9606 GN=CACNA1A PE=1 SV=3
MARFGDEMPARYGGGGSGAAAGVVVGSGGGRGAGGSRQGGQPGAQRMYKQSMAQRARTMA
LYNPIPVRQNCLTVNRSLFLFSEDNVVRKYAKKITEWPPFEYMILATIIANCIVLALEQH
LPDDDKTPMSERLDDTEPYFIGIFCFEAGIKIIALGFAFHKGSYLRNGWNVMDFVVVLTG
ILATVGTEFDLRTLRAVRVLRPLKLVSGIPSLQVVLKSIMKAMIPLLQIGLLLFFAILIF
AIIGLEFYMGKFHTTCFEEGTDDIQGESPAPCGTEEPARTCPNGTKCQPYWEGPNNGITQ
FDNILFAVLTVFQCITMEGWTDLLYNSNDASGNTWNWLYFIPLIIIGSFFMLNLVLGVLS
GEFAKERERVENRRAFLKLRRQQQIERELNGYMEWISKAEEVILAEDETDGEQRHPFDAL
RRTTIKKSKTDLLNPEEAEDQLADIASVGSPFARASIKSAKLENSTFFHKKERRMRFYIR
RMVKTQAFYWTVLSLVALNTLCVAIVHYNQPEWLSDFLYYAEFIFLGLFMSEMFIKMYGL
GTRPYFHSSFNCFDCGVIIGSIFEVIWAVIKPGTSFGISVLRALRLLRIFKVTKYWASLR
NLVVSLLNSMKSIISLLFLLFLFIVVFALLGMQLFGGQFNFDEGTPPTNFDTFPAAIMTV
FQILTGEDWNEVMYDGIKSQGGVQGGMVFSIYFIVLTLFGNYTLLNVFLAIAVDNLANAQ
ELTKDEQEEEEAANQKLALQKAKEVAEVSPLSAANMSIAVKEQQKNQKPAKSVWEQRTSE
MRKQNLLASREALYNEMDPDERWKAAYTRHLRPDMKTHLDRPLVVDPQENRNNNTNKSRA
AEPTVDQRLGQQRAEDFLRKQARYHDRARDPSGSAGLDARRPWAGSQEAELSREGPYGRE
SDHHAREGSLEQPGFWEGEAERGKAGDPHRRHVHRQGGSRESRSGSPRTGADGEHRRHRA
HRRPGEEGPEDKAERRARHREGSRPARGGEGEGEGPDGGERRRRHRHGAPATYEGDARRE
DKERRHRRRKENQGSGVPVSGPNLSTTRPIQQDLGRQDPPLAEDIDNMKNNKLATAESAA
PHGSLGHAGLPQSPAKMGNSTDPGPMLAIPAMATNPQNAASRRTPNNPGNPSNPGPPKTP
ENSLIVTNPSGTQTNSAKTARKPDHTTVDIPPACPPPLNHTVVQVNKNANPDPLPKKEEE
KKEEEEDDRGEDGPKPMPPYSSMFILSTTNPLRRLCHYILNLRYFEMCILMVIAMSSIAL
AAEDPVQPNAPRNNVLRYFDYVFTGVFTFEMVIKMIDLGLVLHQGAYFRDLWNILDFIVV
SGALVAFAFTGNSKGKDINTIKSLRVLRVLRPLKTIKRLPKLKAVFDCVVNSLKNVFNIL
IVYMLFMFIFAVVAVQLFKGKFFHCTDESKEFEKDCRGKYLLYEKNEVKARDREWKKYEF
HYDNVLWALLTLFTVSTGEGWPQVLKHSVDATFENQGPSPGYRMEMSIFYVVYFVVFPFF
FVNIFVALIIITFQEQGDKMMEEYSLEKNERACIDFAISAKPLTRHMPQNKQSFQYRMWQ
FVVSPPFEYTIMAMIALNTIVLMMKFYGASVAYENALRVFNIVFTSLFSLECVLKVMAFG
ILNYFRDAWNIFDFVTVLGSITDILVTEFGNNFINLSFLRLFRAARLIKLLRQGYTIRIL
LWTFVQSFKALPYVCLLIAMLFFIYAIIGMQVFGNIGIDVEDEDSDEDEFQITEHNNFRT
FFQALMLLFRSATGEAWHNIMLSCLSGKPCDKNSGILTRECGNEFAYFYFVSFIFLCSFL
MLNLFVAVIMDNFEYLTRDSSILGPHHLDEYVRVWAEYDPAAWGRMPYLDMYQMLRHMSP
PLGLGKKCPARVAYKRLLRMDLPVADDNTVHFNSTLMALIRTALDIKIAKGGADKQQMDA
ELRKEMMAIWPNLSQKTLDLLVTPHKSTDLTVGKIYAAMMIMEYYRQSKAKKLQAMREEQ
DRTPLMFQRMEPPSPTQEGGPGQNALPSTQLDPGGALMAHESGLKESPSWVTQRAQEMFQ
KTGTWSPEQGPPTDMPNSQPNSQSVEMREMGRDGYSDSEHYLPMEGQGRAASMPRLPAEN
QRRRGRPRGNNLSTISDTSPMKRSASVLGPKARRLDDYSLERVPPEENQRHHQRRRDRSH
RASERSLGRYTDVDTGLGTDLSMTTQSGDLPSKERDQERGRPKDRKHRQHHHHHHHHHHP
PPPDKDRYAQERPDHGRARARDQRWSRSPSEGREHMAHRQGSSSVSGSPAPSTSGTSTPR
RGRRQLPQTPSTPRPHVSYSPVIRKAGGSGPPQQQQQQQQQQQQQAVARPGRAATSGPRR
YPGPTAEPLAGDRPPTGGHSSGRSPRMERRVPGPARSESPRACRHGGARWPASGPHVSEG
PPGPRHHGYYRGSDYDEADGPGSGGGEEAMAGAYDAPPPVRHASSGATGRSPRTPRASGP
ACASPSRHGRRLPNGYYPAHGLARPRGPGSRKGLHEPYSESDDDWC

PFVM
     00000000000000000000000000000000000000000000000000000000000000000000000000000000000000000000000000000000000000000000000000000000000000000000000000000000000000000000000000000000000000000000000000000000000000000000000000000000000000000000000000000000000000000000000000000000000000000000000000000000000000000000000000000000000000000000000000000000000000000000000000000000000000000000000000000000000000000000000000000000000000000000000000000000000000000000000000000000000000000000000000000000000000000000000000000000000000000000000000000000000000000000000000000000000000000000000000000000000000000000000000000000000000000000000000000000000000000000000000000000000000000000000000000000000000000000000000000000000000000000000000000000000000000000000000000000000000000000000000000000000000000000000000000000000000000000000000000000000000000000000000000000000000000000000000000000000000000000000000000000000000000000000000000000000000000000000000000000000000000000000000000000000000000000000000000000000000011111111111111111111111111111111111111111111111111111111111111111111111111111111111111111111111111111111111111111111111111111111111111111111111111111111111111111111111111111111111111111111111111111111111111111111111111111111111111111111111111111111111111111111111111111111111111111111111111111111111111111111111111111111111111111111111111111111111111111111111111111111111111111111111111111111111111111111111111111111111111111111111111111111111111111111111111111111111111111111111111111111111111111111111111111111111111111111111111111111111111111111111111111111111111111111111111111111111111111111111111111111111111111111111111111111111111111111111111111111111111111111111111111111111111111111111111111111111111111111111111111111111111111111111111111111111111111111111111111111111111111111111111111111111111111111111111111111111111111111111111111111111111111111111111111111111111111111111111111111111111111111111111111111111111111111111111111111111111111111111111111111111111111111111111111111111111112
     00000000000000000000000000000000000000000000000000000000000000000000000000000000000000000000000000011111111111111111111111111111111111111111111111111111111111111111111111111111111111111111111111111112222222222222222222222222222222222222222222222222222222222222222222222222222222222222222222222222222333333333333333333333333333333333333333333333333333333333333333333333333333333333333333333333333333344444444444444444444444444444444444444444444444444444444444444444444444444444444444444444444444444445555555555555555555555555555555555555555555555555555555555555555555555555555555555555555555555555555666666666666666666666666666666666666666666666666666666666666666666666666666666666666666666666666666677777777777777777777777777777777777777777777777777777777777777777777777777777777777777777777777777778888888888888888888888888888888888888888888888888888888888888888888888888888888888888888888888888888999999999999999999999999999999999999999999999999999999999999999999999999999999999999999999999999999900000000000000000000000000000000000000000000000000000000000000000000000000000000000000000000000000001111111111111111111111111111111111111111111111111111111111111111111111111111111111111111111111111111222222222222222222222222222222222222222222222222222222222222222222222222222222222222222222222222222233333333333333333333333333333333333333333333333333333333333333333333333333333333333333333333333333334444444444444444444444444444444444444444444444444444444444444444444444444444444444444444444444444444555555555555555555555555555555555555555555555555555555555555555555555555555555555555555555555555555566666666666666666666666666666666666666666666666666666666666666666666666666666666666666666666666666667777777777777777777777777777777777777777777777777777777777777777777777777777777777777777777777777777888888888888888888888888888888888888888888888888888888888888888888888888888888888888888888888888888899999999999999999999999999999999999999999999999999999999999999999999999999999999999999999999999999990
     00000000011111111112222222222333333333344444444445555555555666666666677777777778888888888999999999900000000001111111111222222222233333333334444444444555555555566666666667777777777888888888899999999990000000000111111111122222222223333333333444444444455555555556666666666777777777788888888889999999999000000000011111111112222222222333333333344444444445555555555666666666677777777778888888888999999999900000000001111111111222222222233333333334444444444555555555566666666667777777777888888888899999999990000000000111111111122222222223333333333444444444455555555556666666666777777777788888888889999999999000000000011111111112222222222333333333344444444445555555555666666666677777777778888888888999999999900000000001111111111222222222233333333334444444444555555555566666666667777777777888888888899999999990000000000111111111122222222223333333333444444444455555555556666666666777777777788888888889999999999000000000011111111112222222222333333333344444444445555555555666666666677777777778888888888999999999900000000001111111111222222222233333333334444444444555555555566666666667777777777888888888899999999990000000000111111111122222222223333333333444444444455555555556666666666777777777788888888889999999999000000000011111111112222222222333333333344444444445555555555666666666677777777778888888888999999999900000000001111111111222222222233333333334444444444555555555566666666667777777777888888888899999999990000000000111111111122222222223333333333444444444455555555556666666666777777777788888888889999999999000000000011111111112222222222333333333344444444445555555555666666666677777777778888888888999999999900000000001111111111222222222233333333334444444444555555555566666666667777777777888888888899999999990000000000111111111122222222223333333333444444444455555555556666666666777777777788888888889999999999000000000011111111112222222222333333333344444444445555555555666666666677777777778888888888999999999900000000001111111111222222222233333333334444444444555555555566666666667777777777888888888899999999990
     12345678901234567890123456789012345678901234567890123456789012345678901234567890123456789012345678901234567890123456789012345678901234567890123456789012345678901234567890123456789012345678901234567890123456789012345678901234567890123456789012345678901234567890123456789012345678901234567890123456789012345678901234567890123456789012345678901234567890123456789012345678901234567890123456789012345678901234567890123456789012345678901234567890123456789012345678901234567890123456789012345678901234567890123456789012345678901234567890123456789012345678901234567890123456789012345678901234567890123456789012345678901234567890123456789012345678901234567890123456789012345678901234567890123456789012345678901234567890123456789012345678901234567890123456789012345678901234567890123456789012345678901234567890123456789012345678901234567890123456789012345678901234567890123456789012345678901234567890123456789012345678901234567890123456789012345678901234567890123456789012345678901234567890123456789012345678901234567890123456789012345678901234567890123456789012345678901234567890123456789012345678901234567890123456789012345678901234567890123456789012345678901234567890123456789012345678901234567890123456789012345678901234567890123456789012345678901234567890123456789012345678901234567890123456789012345678901234567890123456789012345678901234567890123456789012345678901234567890123456789012345678901234567890123456789012345678901234567890123456789012345678901234567890123456789012345678901234567890123456789012345678901234567890123456789012345678901234567890123456789012345678901234567890123456789012345678901234567890123456789012345678901234567890123456789012345678901234567890123456789012345678901234567890123456789012345678901234567890123456789012345678901234567890123456789012345678901234567890123456789012345678901234567890123456789012345678901234567890123456789012345678901234567890123456789012345678901234567890123456789012345678901234567890123456789012345678901234567890123456789012345678901234567890
     MARFGDEMPARYGGGGSGAAAGVVVGSGGGRGAGGSRQGGQPGAQRMYKQSMAQRARTMALYNPIPVRQNCLTVNRSLFLFSEDNVVRKYAKKITEWPPFEYMILATIIANCIVLALEQHLPDDDKTPMSERLDDTEPYFIGIFCFEAGIKIIALGFAFHKGSYLRNGWNVMDFVVVLTGILATVGTEFDLRTLRAVRVLRPLKLVSGIPSLQVVLKSIMKAMIPLLQIGLLLFFAILIFAIIGLEFYMGKFHTTCFEEGTDDIQGESPAPCGTEEPARTCPNGTKCQPYWEGPNNGITQFDNILFAVLTVFQCITMEGWTDLLYNSNDASGNTWNWLYFIPLIIIGSFFMLNLVLGVLSGEFAKERERVENRRAFLKLRRQQQIERELNGYMEWISKAEEVILAEDETDGEQRHPFDALRRTTIKKSKTDLLNPEEAEDQLADIASVGSPFARASIKSAKLENSTFFHKKERRMRFYIRRMVKTQAFYWTVLSLVALNTLCVAIVHYNQPEWLSDFLYYAEFIFLGLFMSEMFIKMYGLGTRPYFHSSFNCFDCGVIIGSIFEVIWAVIKPGTSFGISVLRALRLLRIFKVTKYWASLRNLVVSLLNSMKSIISLLFLLFLFIVVFALLGMQLFGGQFNFDEGTPPTNFDTFPAAIMTVFQILTGEDWNEVMYDGIKSQGGVQGGMVFSIYFIVLTLFGNYTLLNVFLAIAVDNLANAQELTKDEQEEEEAANQKLALQKAKEVAEVSPLSAANMSIAVKEQQKNQKPAKSVWEQRTSEMRKQNLLASREALYNEMDPDERWKAAYTRHLRPDMKTHLDRPLVVDPQENRNNNTNKSRAAEPTVDQRLGQQRAEDFLRKQARYHDRARDPSGSAGLDARRPWAGSQEAELSREGPYGRESDHHAREGSLEQPGFWEGEAERGKAGDPHRRHVHRQGGSRESRSGSPRTGADGEHRRHRAHRRPGEEGPEDKAERRARHREGSRPARGGEGEGEGPDGGERRRRHRHGAPATYEGDARREDKERRHRRRKENQGSGVPVSGPNLSTTRPIQQDLGRQDPPLAEDIDNMKNNKLATAESAAPHGSLGHAGLPQSPAKMGNSTDPGPMLAIPAMATNPQNAASRRTPNNPGNPSNPGPPKTPENSLIVTNPSGTQTNSAKTARKPDHTTVDIPPACPPPLNHTVVQVNKNANPDPLPKKEEEKKEEEEDDRGEDGPKPMPPYSSMFILSTTNPLRRLCHYILNLRYFEMCILMVIAMSSIALAAEDPVQPNAPRNNVLRYFDYVFTGVFTFEMVIKMIDLGLVLHQGAYFRDLWNILDFIVVSGALVAFAFTGNSKGKDINTIKSLRVLRVLRPLKTIKRLPKLKAVFDCVVNSLKNVFNILIVYMLFMFIFAVVAVQLFKGKFFHCTDESKEFEKDCRGKYLLYEKNEVKARDREWKKYEFHYDNVLWALLTLFTVSTGEGWPQVLKHSVDATFENQGPSPGYRMEMSIFYVVYFVVFPFFFVNIFVALIIITFQEQGDKMMEEYSLEKNERACIDFAISAKPLTRHMPQNKQSFQYRMWQFVVSPPFEYTIMAMIALNTIVLMMKFYGASVAYENALRVFNIVFTSLFSLECVLKVMAFGILNYFRDAWNIFDFVTVLGSITDILVTEFGNNFINLSFLRLFRAARLIKLLRQGYTIRILLWTFVQSFKALPYVCLLIAMLFFIYAIIGMQVFGNIGIDVEDEDSDEDEFQITEHNNFRTFFQALMLLFRSATGEAWHNIMLSCLSGKPCDKNSGILTRECGNEFAYFYFVSFIFLCSFLMLNLFVAVIMDNFEYLTRDSSILGPHHLDEYVRVWAEYDPAAWGRMPYLDMYQMLRHMSPPLGLGKKCPARVAYKRLLRMDLPVADDNTVHFNSTLMALIRTALDIKIAKGGADKQQMDAELRKEMMAIWPNLSQKTLDLLVTPHKSTDLTVGKIYAAMMIMEYYRQSKAKKLQAMREEQDRTPLMFQRMEPPSPTQEGG
Predicted Results:
   1 ..EW$YZCVABVVJWPAAAAQABEVJCAJWPCCYWSBPAFCYCYDAAAAAAZAAAASAABAABCABSCAPPABEBJAAEBEWCPAAAADPWAADAVYJCQSSSADAAAAWWBREAAAWACWVYAQAPYZSASSVSVPSAAEEEEEZAASBBEAEAAVBWCAAWAAD$WJBDAABEEDEAAJABAUEJSASVAAAAABAEAAASADAVSPSAPAWEAAJAAAAEABAAEBCAAAARAAEABABBAAWBBEAWJRSEEEEPYBCADJBSBCAAAASCJAYAPQVZJAAAAAAEADCQYSAAYAAAEEJADAAABBAAPEPAAJAAEAWJAAZQYJPAAAAABLEAABVJAAYEEAAEAAAAJAAAAAAAAAAVAVAAAAAAAAAADAAAAEAAWBBBADYAADEAAAVAAYVCYJJJZYAAAAPLESSVWYAAAAJYAABADAAAAAAJDCVVPDVEASVAAEAAYAVACWYBBJABVVEAAAEADAJAJRBBBAAAAEAAAEEAEAEEYWPJAAJAADAAEAEERBVZAEVAAEAAAADCCBBJESPBPDDDDDQQALEDAADABEAJABBWYAAJWEJJWAAAAAAAAABEBAADAASAAAWEAAAAAAAVJAEAAAAAAAAEBABAAAPAABAJBDRBBVBZWCCSCJAAVJJYAAADWEAEDPYZSAABACPVJAEDACWWSQSAAAAEAADBAADJ$EEBDASBEAAAEYDAJAAZVWDAYAAJAAAAAAAWAAAAAEAAAAADABEDWDSFJABAAADEADBJJSCAAAAAPPVYAAEDDAAAASAAAAVAAAAWAJADAAAAJDAJVWAVBBAAAPSJEVWVDADVDBAVBWPZACPVPBAAAAAJDAVASAAAAAPYSVJAADJVQEAAAWWVJPPBBPPVAAAAAAPPSBSWVEDDAVAAAAJVWSJSLRVPAAAPACJJJADDAAAAWAPCAAAAYYJYAPSZPAZAAADJAAJPYPAPWYBDAAJAAAAAAWJJJJSDWAYPEPYP$QYAAJEAWEEYPSAEDSQBAVAVABVAAESJJADAYQAVJPCVWWVJJDASCVWAQDAWSWBPCACACJAAAAAPBABAYAABACWCAAEWBAPSWJSVJYYEJJSBSJSAAWVAAVECVAJAAAYSCZCJVCPVAQEYCSYPVY$YASVVBZYASAVAPJAAABSRWWALBEWWYAJWCSWWAEAABEWRJICVBCCSSVYAAAAYAAAAVPCYJAPYYJJYAJWYBRCWPCPAAAAWAAAESBEADAAAWWBAEBEBAWAAAAAAJVACVAYASSAAAAERASWLBBAEEBWAEAAAAAQVDJAWAW$AAADVAAAAAWEEEBASAACEEVWWYSQSAAJAAAAADAAAAAAAAAAAAACDAAAVDDEWEEDAEAAADAAEEEAEEEAAAWAAEEBSDAJPPAJAAAACCBYAADBABWVPAJEAAASAAWYADJDAADEESEEEDVVAAAAABAAABPSPPPRWAADVSBJVRADDBBCSBALWRRRPJEBBAEABEEVDDEAADAAABBBDAYAAJPBYAAVWAAAWJASSSAWWAAAAACESDDDWWYAAAAARPEBBBAJEWSVQCEAEBAAAAWAJEABAEEDQAAABEAAAAAEESCAWEAAWACEWBBAABEACARJAADDDDPADPADCABAABYAABEBBDAW$JDAAVAJAAAAAAAAAAAAVAVYJJBAABDADEDAABARAAWESAEAAAADEAEAEAAALAASWWSEBVCCVJVSAAAASSAABSSAAAASAAAAAASBDDPPCABBBBAAAAADYPSJBVDQAAEBZPAWWAAVDERRAAEEAAPAAEAAABEAABAAAWSAAAEAYAAAJBAABECAABBWAADPBVADAAAWYAJAAAAAAAAAWPDJLAZQWJJADEEEAAADUAAPAWCCVAYAEVAJWAAADAAAAASERAJAUAZJAJADDJAAAADAAAAAVVVEAVAAAAEEWBAAJVJJWVABDAAAAAAAAAAAASAAAAAAAAARAAABJPAAWWASPPCCSEZW..
   2 ..DVAZ AP AWQPASYPJCASJBEPWVSYQWYPPWVAQYJCAA     S VD DJADE JBV WSDB    AAJPBVWAWVSQDBBSADPDSAD         EDDED  ABBEWDAD  YZSPPWZ ADAAAZWAVVZR     DVPWLBBBJWEW F  PJDEB B A  ABBBBBPABADYAPBDBADDEPSEDAJDDBEBEZJWCJJDAABEVDBDEAPSVDDAADDDDB WBEEEDEBZEAD D VS     YA P PVVJJV    YSCCACA Y S      C  DYJPPEAPRPJAAWEVDE     BACVPDDVDAVPBAPSYJ     EBABSDEEEB   SBABVDJYEDDDVQDBDDW$A EVEBDDVQJSJCJDYWE    D ADWJADBDDJDPCPA   E VRDBAB WEAYVPLDJSZDJA AJDDDCWEVSSSAAAAWAJJPBDDVVSDJ P A B    EWDREBD  R E  BDDBAVDDRA  DASW J DDADDAEDADABBDSAD B  B    PYBLJ  ASWS     DP  AVW CEA WS EAPVJJEBSEPAEBSEDDBEEA SJJ   ADDDPBBDDJD DPADWWDBDDDDDBEDEEDDASDDDQ$ SPS  $PWACAA VAVRADED   DDRJJW J D  WCABSAVAYAWJYDEB    EECBAACRPAAWAAABDEAEAYADJAJAAWZJY DDDDD DADPBBDADEDDREVASAAAR PBWEDD AD A   SRYDDD    D    SDEDAYPDDA    V S AD VVAAVA VDJ  EP SBARWBY  JW VDPJPJADDJAJEBDWYCA DAVADDEPDB AA VJCBZJADVJPEZD     BY JDEWPJ$BAJERB   A PECYAJ S S$ JJDVYYYAFVD     D JVBVCPDWZYZEVLASQDED J CDSJZAPZPCVJSDCRDDWCCJYBWWWQVPVEAESCYJCPEAADS  VSFSSARCPZABYWJA  BRSPV DZVDCSWSEPBCSBEVJPYV P PAAAW YBVPAAD    SADADADWAJR JVDUSYSWCVPCDP SAAPYJ BA  LDWJAWJY AVDVCWWYWP V JCSV   VWAWAVBAEBJWQPAEERBADPZWJ   J AC YVJCC W  BL WEVDAD JESLBYBYAJ$DVAJDVVYQYAQCC V  CSW ZEEBBJVJVDD A  DPEADWA      DAAV  ADEEDJQP VJCYP   DC DAQJJ BVWBAD B   BBJBVYAERC  BQDWZA  EVDBSDCVRBDDSARBABWJVEPPSDVDVDASDBBDJDDVVDDWAPCCWWAEB JBBEADVDAYDABB A    B  BDAEWADAADDA    WYABDJA JQA YJR SSPJSDAADA  E  WR       DDEEEADJVDYY YAS BDAJ WDEVEAQ   CCP B   J A   R  ABA  B D ELDAAEAEVDDW$  DWA ESDAAJ   W  VYBWPSCC     S B    JB    ECEVY E DA   D JDARB E   ABZSAABDJDJAB A  BBDAWA B   DWBE DSADW AA JBAVBVSDEDBAVVDEBSAZC YBAPDBEAD BWWPBBRBDDADQ AEEDDAABE A  WSAECL  EWD D AADAEDDEEW DAAAARVJAYAAYVDDYBEB V   BJE  D DDDZAAWBWBW     B  JD P  BW QJVSAAYWS  SJ  W  DWLB BJ  ABDBWBBDAVBD BDDDAEJYJDPWSBDBALDWEEDDAAWWDAD  B VAV    D     SACBYY    AAV DDDASVV CSJWPS EV JAL EDASDDSEEBVDAWBDREJ D  ADDDD        AAJADEDBCCB    $AWCUYRJWDDED DD DDDADPDDDDJDYDYDVYPS V       AVCPZ..
   3 ..AAC  W  SQPVYJCCDDPESSWWVYQSSPWSVCZS$CPVJZ     Y    P  WJ  ES CVRE    SPAAEDADAAEAEVDDPJAWBWV          BBBE  SADBEYD   CVJSD   CYJDWJA  BD      JWCEERDADYAE    ESJAW      ERAAJPDRPWEWCAJEEBVEVCDABDBWSWDAJAYJBVBBBBVJA D   BCDSAEEBE EA DABADEADB  E C A      Q$ J WWJBWF     Y YS Y C V      A  JJ YJD  ESBBEEAWEW     Y S A BAEF VDBACBA     W BSBEUS S   BEBEEVEZBE JPVSSBEADJ DDDDB E  A  DWVQ       ZYVABBDSYS  AYD   R DDJCCC EWWJEJDVDCVCDW SPBPSVDDAACEJCLSBRAPCDJJABBYA B J      DD L EW       VEEDBPS     BD     WR  YJVB  R EEAD  A       QVYPP            W  BBE EJE VB  JCZPWREBWEDBDDQVBDBBE RE    D VJJWDBEDS  WR AB D RBE  D DLEEQ BA AJ EJE  DJJJ S  W  ABR      AAVA$ Y    JSCDBSW$CEPYJEY       D WPQYBESEJEDEBDBUPCYVPEAVSBVVZ EJYEJ  BEDDEWD DBSWAPJ SCPA   DWWQ D  S   BW        J     VYVPDV  B    Z    F WD   D C B  DV JCSABSZ   D J   VWDCPCBASDY C P YSSD  BDJW  S  D PYAPWPWBAYA     CA DWCJW CYJAB D     CYRVCC   RE ADJQCWJWRE        PJS QDQP WBBRBYQPJB  A W ASCSWAC CAY ADSY DDVAWPAYAYJJOUOCSZDADVPD V  Z ACVPYEVBZPZJSW   ABCD W$SYYYABBJJ  VBPE A  V JDCP  SYADS V    YVPDWWEDEYV VYSSPJYSBB WJ  WZPBJZ CY  BS VE    DCSDEBSCAB B BSJB   CCD SCEBDCSJADVJWJADVB EC     SB S CW  C   B ESLAZ  WWCVSCCWRPDEJRDCWD CSZVSS     P   AJWCZEDSJY     ABEJ E       B B  SSBDYWDS   Y A   JW  WSBB EA EJR D   DDDWAAJRDA    VB     DRRRABPYASEABASVJPPPYWSAP PDJLVEEDEBWSSY JDESVJBABRJ R  BDBDCVSVBSA      D  EABJJY D$AZ     JVJACVB CJV JB  WD ACJDWZS  B   B         DDBCEDJEVP SVW J J  VAPAB A   JY  E         S  VL   R    W E AE B  VA   DY JC DQS      DEVCDWSE       D          AJBC  W BB   R DBDA      DJJDDD  BPEBA    DDJDEB R   RELD BAB         AEAEEWPPJBBJJJADBF ZAVDSEBWE E EYJEDDWJDCA DB BED J      CDJRB   B     D   BV D  VEZJCSRW$VJSJASZ JB  B   J V  P EBE DDAAVEY     D  B  S  C  APYDWEABP  DE     EE A DA  D SY DESD D  V  ECYAJWVW VV W S J V   DJYJ V  C WV            DWWAV    BDC W EWCDS SBSP V    SBE LE BEECBBAWSPQSJAS     BJEQ           DPCSBEAW     ASAQESEBE  D     E    DVBBVDYVABS  AY D       BYLEQ..
   4 .. CP  J  WYYICCJDZWDDCWBBYPPDAVZACBJBYA  PD          R  BP     YRA      D SV BEBBBJIDE  V BYJ           EEDV  JS DBJ    SB CJ     DWPAB           QAJSSEJEPWL    REPJ       WAREWVJCQDWEJVZBRDBJWDJLEBWBRVSCWDVCW EWD J       SECEBD EB    E DDBAUJD    W         S Q   S PW     W DV Q B Y          B C B  SBA BJWEJB     R Y D  BBC  JYJVP        SVEABW V   VDDFCCWBVB PYJWDERDQP BYWJV W  R  W ZY       DEBSDESWJC  WWR   Y JVBDLE CCBSSDEJBWDJYJ VV YJBVAYDJW SWWJBPYVASBWJE   C        R    CB       PB WDCB     EB      E   ZD      WDE          JASCV            A  SEC S   EA  SYFWBD CBBEDWCDELS  W DP      ESSJEEBVJ   F BS E   B     D RY P  VP BE   AESP V     DD        BACS       JSJ BCPPYQ  RZ       E V$ FARBPYDB EBLBEDS DRDDREASJ BYJCV  YSEEDVS YJEBRWV VBEE   SBJY R  C   JB              RCJJCB       Y      RE   P         WVBBP B   P     A VYVBEC S    Z   WJ  DBP   J    SCPJPQSASA      JD  RJPJ BC VL       YJSZDL   E  BB PJPZP R        WCJ J YJ ZVCJWCY PJ       W JYV  APB  YC   J $A BVYCWPACAAWQABSJVR B    LD EWBCCYDDSPB    V$   C CJASEWAE     W    Y DPYQ   DJVJ Z    BD EEZJVSWY    CEP AWA VA  A SLBW VV  CA W     SDWQAR     D PVPU   EJY  BJELWAQJJYP SJ CW PF     ES C S          V VJ  SSPSWVYZDDJSYDSJY   VPPZJ     V    LAAVP Y V     CDDB           A   JDJZDPW   A     SV  BA A  E SBA S   EJ SPCEV S     D       DBBUEBWRBDDCW PVB AVVVE EEWVBJSCJWBRCC  BWRDDEEEAW    D YJJ DYJA       W  DBSAAR PDY      AAPV D  D   ER  DY YPEJZ C      D          JDW EBLU   W  D S  YC ED W   P   R         B  B         D R D  W  AJ    P LY  S       BSDDW JR       J          D PJ         V VS B      J SEE   VVB D    AE RDW V    ARW V             B VJEYDEYBWAEPAR  YBWEA BB R B QCJEESWYE WW EB         D  WE   A         AE    WW EYBSYVPYV CWY         V      SE  EVJZAY      E  D  V  S  J JLSPD E  LV     BD   E   S  D LDBE    E  BWBBSDJL Y  D W A     B B  U  J Y             PEVDW    WBW C PBV   YYP  P     SJ BS RJRDLW B  BVC$B     WEJJ           ZYWBEJBJ     RWBESCDWB             SPEJ     V   WZ           SLV..
   5 .. QW  V   DDWPASSPBWJPAUEZJWPWSVJAYEJ P  V              JS     BE       B YY R CJYYJWW  R  DB            SSB   D RD     FP Y      BJDR             EARCSD D      JR P        S VDDCDESSBPYAJ JWSBLWRCSCVCEBJDJASA AVE W        R RSQ VJ         WVWE              C Y   C CA        W                  B W   CD SLBSB          E   PS  E V$V         D  Y  Y     JVBJBQJZ BE  JSB YD  EB   S  E  S J        JPJEWVECAD  YSZ      JWVB  RASBBECQPB SRS  D JRW YWPWY VDPEE SBJ PJWR   W              P       DJ EVSW                          P           B WWW            J  RAP B       WSCCPA WPCBQJJVYWE     B        EAVF BW   P VD S         R QU    L  Z    PA          Z        EBSY        VZ  QS DY  V        J EC AYVRDEWJ IWYAQVV SV J CJYV J$PJW  JBV JS  CERCW C JEZB    DVJ S  J   V               JDPCVC                   W         A EJE E           YZWSWB              WW         C SRJRYZBJ      SZ  SBDV SW  R       JVBASP      CE BSCP           CYP D SD  CV VDJ AF         QJC  Y J  PW   W WD CBJBSCCVCP CPEYZWS D    CW CB SJ QPV E    AY   J EQWJWYQP     P    D  SBV   SPYY J    JY SC WPW Z    JV  C S        AP$  B  DB        YBYP      W WJ W   SYS  PRDB VB SBZ PC W  SL     CR L              JV   BVJPEVPVSEBBVBVB   PS$ Y     B    WFEAB B       DAJA           C   EWSCVSC          J  CE W  U WLW A      CBSBB E             JA PQVCE E B  SCA ZB Y  VB BD RP CVCWW  EJ SSDSSB     S ECS  E            PWVYEE RVC      BB W P  V   BS  BP BV VB J      S          B   RLPC      S Q   Q  R B   B                D                Z  P     W PR  E       JD V   A       Z            CQ         E A  V      W BCV   E   W    LL VLE      DU  W             C  ASJFA PPSRSWS  C B J DW   D DD  B SWY  L            E  Y               W     V BWCAEWAWB D C                    J CSJS         Y  J  A  P PRBD  R          V            W E     R   DZSBVYS    S   L     S    E  S               SSPJJ    VS     E    VVD  Y     P  DB EBBJDC E  JJPER     EB V           EB WPDDB     ZCCDCBWSJ              B       D                WV ..
   6 .. YB      CAEVBWVWEJPERAAAQYZBAJC$ SV R  B               B      D       Y    D  C  SC   A  JV             WW      S      A V       V C             YRWWV  E      C  W        W SAWSZDEZV ZDW  E DVBJS EE CRVQQBYE VL  D          J    S         YD R              V     P S                                  D  DD B               J   V  AS         W  A         WPRCACR V   PCJ  Y  P    Y     P A        EZEBJWREZY  JJ       S JE   V VC V CP PWE  W V Y  E YD  EY   DES VSCY                  S       E  JCW                           Q             JS             S   L          L$SYRB F WYREEJ  V     S        Y LC P      LE W           SV    D                   V         WZ         Y   YB JF  S        R ZQ     VQJ    RDW        S DW WSVWB  DVQ WY  JWLDB W WPBF    FE     Y   W               BVSDZD                   V         V DDL R           P AW W               L         A YYVJECJR          EDCZ V   V       UDJSBS      R   AV$           SBY   VV  SY YBB CL         CRB    C  E         SCCSVSLRLW  WJ PSB E    WP WE WS WWE J    WA     WWZBV VC     V    A  VWJ   CSC  Y     J  P  EV      AA  J          WVV  D  FW        BCJB      A  P      A   E JW CY WC  YY     R     RY V               Y   C FJBZESYPVWSVQC    WA       C     JDDL P       BRP                YLLEYWY          D  LB      PS          DWPA               A   UDPY B L   SW LJ D  YW EE VV EE     VP WPJD S       JP   B            SLRDBB  EZ      SJ Z    P    V  EC PB ED                   Y   BWJB        W   W        W                                    Y       YE  P       SB B   W                    LW           B  J        DW    R        RP E Y       V  E             W  DWDEJ WSRWWJQ  E V W LR   V PS  S CZP                  E                     Y   JE  QBW Y J                      EJQA            A  E  V CV W  W          S                        JC VE                W       E                DYPP    E      Q    EJV        W     DRSYW  C  VEWPC     DW W           PS DSWRA     BLEBR  RP              S                        DJ ..
   7 .. JZ      BESQWVWYJVVWDYCPWEAJYSQB       S                                      P   J   Q  V                      L      C A         Y             R                         J W JYWJJB  D P  L SW W  SC    B$  F S   E          B               W                      A                                    J  LS D               Q   P  BA            W         CSPDPIS C   EJW          B     R W        SV  V WBQQ             S    B A  Z V   BD  C   J  S  J   B   CYY WCPF                          Q  RWD                           R              E                 W           B S S   A SCBB  P              A SR W       L             V     E                             CP         W   DJ $J  B          BD     J       V         J WS  CCBP    B C   VP JC S BJW     LQ     P   P                B W W                               C Y             C S  E               S            CSCCYDE          LWSE W           ZPVEE       S   BSQ           YW    CB  AJ AWR SS         YFQ    D            V PE AWYWJ  $P BCZ J     V  V     AY      CV     VPVCP B      C         C   VDZ           J  JP      IB  F          VWC     PY        ZJ J      S  B            C  WE  J  BV            A                     Y PRJ ACBVCECPSJ    $B       J      SYW         WVC                  CRPY           R  R       JR          EESS               E   AEJB W     AY  Y Z      W WE SC      S BWRC W       VY   R             IWS    Q$      V            E  J  ZD LE                   P   S SA        E   S                                             D       BJ  Z          E   B                                 L           EV    S         J              S             L  VBC D  A  V      J U  S     WW     ED                  V                         WL  DIE P S                      BQUC            Z  U  Y DE S                                       J EB                        A                ACVS    S      C    BPW        R     PS ZP     ZQYY      PS C           QW C YJR     C SAB  A               R                        FW ..
   8 .. D        CYSYZYVPCYA LDSEAVZJPDY       W                                      E          P                      C      D B                       B                           R U  W R  Q V          PL    PP  J Z   L                          S                      W                                                          S   Z  PC                      QWWRWSV     RR           C     L          W   Z  JWZ             P      C  W     PP  Y   D  P  R       VSV CZ C                          W   JR                                          J                 U           E   F   L  PPS  R              B WS Q                     W     P                             EV             PQ CR             SZ     L       W           C   BSPY          SS EY   PVY      Y     V   Y                    Y                                 J               B                  V            LQDVJ P          B  Y             QSP         Z   WBV           B     PA   U  EA            $ZS    L            E SP  ISI   BR WBJ C              QC      P      JABEL S      F             RWB           F   R      RR  B          CCS                E S         D               YS  R   W            W                     R W L CEQYJPECWS    J        W                    Z                  RWCB           F  P       CD          YDLD               P   YJZW           C          DJ PL        P P               S              DW    WJ                   P     CJ  P                         AJ            P                                             S       R   D              D                                 S           WB    W                        R                LRV R            S                 J                                             Z   JPY   $                      VE $            B  W  Z LU                                         P CC                        P                BJ$B           P    JC         L     Q   C     LASV      R  B           V  L VSS     S PRP  E               C                        IR ..
   9 ..          JAJVBJCSBLR  LQDVCVZDE                                                                                 J      J E                                                        C Y    Z          VP    VY    W                              L                      Y                                                                 JI                      RZ  CDW     WL           L                    S  RB              Y      P  Y     SC  S      J  B         W SB J                          J   LE                                          A                             L       R  S W                 V  V                             W                             YB              V PA              V                         Z   V             B  VJ   C              W   A                    E                                 A               J                               VCBBD            J  B              ZD         W   VDL           I     ZS   P  P             Z      P            P W   JWJ   VD YEV U               R      Y      PBPLR E      R              YE                      YC  R          SA                 V V         Q               DV                                         F     JB  RRBWBE    V        Y                    Q                  B              B          F           U W                    C E                        S VP        J S                              CR    C                    W      W  C                         WW            B                                             Z       W   $                                                                  Y                                         SVW S                              S                                                  S    V                         D            C       SW                                         V FP                        V                QEQC                 A                   V     YDES      S  E           Y  V  V        LJ                   J                        RS ..
  10 ..          SULZPBBVEWF  RBBCQIL B                                                                                        W R                                                        Z                 D     S     D                              J                                                                                         W                      UJ  VPC                                          VP              F      E        VR         B  C         F Y                             Z   SB                                                                                S  Y R                 C  W                                                           D               R  C                                            E                 S   E                  E                    J                                                                                 WESDV            P  D               W             ZZS           L          S                       V            R     Q Q   YV CYW                                ZCDY Y                     BW                       U  E          YE                   R         W               P$                                         J      J   SJYEP    C                             S                  P              E          L             C                    J $                          D         Y C                                    J                           E  J                         QL                                                                      C                                                                  P                                         ZLZ Z                              Q                                                                                 J                    WY                                           WQ                        Y                V CE                 Z                          CV       Y  P                 F        VS                                            VF ..
  11 ..            BIDR YRCD  SDCR C  W                                                                                                                                                                           C                                    P                                                                                                                PY  DYP                                           C              W      Z         F         C  L         C                                    J                                                                                V    C                 D  J                                                                           W                                               P                                                             S                                                                                 SRZP                Q               E             D                        W                       W            Y     S S   Z  RD                                 $LV  C                                              Y                                  U                          D                                         A          C RPZ    Q                             Y                                 S          R             R                    W Y                                      L                                    B                           Y                            VS                                                                      R                                                                                                            C S                                R                                                                                 Q                     J                                            R                                           WA                                             B          Y                 W                                                         ..
  12 ..            EEEZ  YBI  VJSX U  U                                                                                                                                                                           Y                                    Z                                                                                                                S   E Y                                                                 $         Y         L            P                                                                                                                          P                 R                                                                              Y                                               R                                                                                                                                                Y$L                U                             E                                                                   V V      SL                                 R    L                                              J                                  W                          P                                                    D LY$                                                                    U          V                                    R                                      V                                    U                                                                                                                                Y                                                                                                            R                                  V                                                                                 V                                                                  Y                                           BD                                             I                                                                                      ..
  13 ..            FRQ   L      ZB    V                                                                                                                                                                           R                                    Y                                                                                                                D   U                                                                                       Q            Z                                                                                                                                                                                                                           D                                               W                                                                                                                                                ZEQ                                                                                                                           IQ                                      U                                                                                                                                                                    FD                                                                    Y                                                                                                                                                                                                                                                                                                                                                                                                                                                                                              W                                                                                                               I                                             R                                                                                      ..
  14 ..            ZF$   S      IF    Z                                                                                                                                                                           X                                    C                                                                                                                J   $                                                                                       R                                                                                                                                                                                                                                                                                        Z                                                                                                                                                 Y$                                                                                                                           DR                                      R                                                                                                                                                                    R                                                                                                                                                                                                                                                                                                                                                                                                                                                                                                                                                                                                                                                                                    R                                                                                                                                    ..
  15 ..            DLU   I      LU                                                                                                                                                                                                                     Q                                                                                                                Y   S                                                                                       Z                                                                                                                                                                                                                                                                                                                                                                                                                                                                                                                                                                       FH                                      $                                                                                                                                                                    L                                                                                                                                                                                                                                                                                                                                                                                                                                                                                                                                                                                                                                                                                    U                                                                                                                                    ..
  16 ..            UD    U      U                                                                                                                                                                                                                      R                                                                                                                                                                                                            H                                                                                                                                                                                                                                                                                                                                                                                                                                                                                                                                                                       LI                                      H                                                                                                                                                                                                                                                                                                                                                                                                                                                                                                                                                                                                                                                                                                                                                                                                                                                                                                                                                                                              ..
  17 ..            $O           $                                                                                                                                                                                                                      $                                                                                                                                                                                                                                                                                                                                                                                                                                                                                                                                                                                                                                                                                                                                                                                    UU                                                                                                                                                                                                                                                                                                                                                                                                                                                                                                                                                                                                                                                                                                                                                                                                                                                                                                                                                                                                                                     ..
  18 ..            IU           R                                                                                                                                                                                                                                                                                                                                                                                                                                                                                                                                                                                                                                                                                                                                                                                                                                                                                                                                                                                                                                                                                                                                                                                                                                                                                                                                                                                                                                                                                                                                                                                                                                                                                                                                                                                                                                                                                                                                                                                                                                                  ..
  19 ..            H$           H                                                                                                                                                                                                                                                                                                                                                                                                                                                                                                                                                                                                                                                                                                                                                                                                                                                                                                                                                                                                                                                                                                                                                                                                                                                                                                                                                                                                                                                                                                                                                                                                                                                                                                                                                                                                                                                                                                                                                                                                                                                  ..
  20 ..            OH           F                                                                                                                                                                                                                                                                                                                                                                                                                                                                                                                                                                                                                                                                                                                                                                                                                                                                                                                                                                                                                                                                                                                                                                                                                                                                                                                                                                                                                                                                                                                                                                                                                                                                                                                                                                                                                                                                                                                                                                                                                                                  ..
  21 ..            RQ           O                                                                                                                                                                                                                                                                                                                                                                                                                                                                                                                                                                                                                                                                                                                                                                                                                                                                                                                                                                                                                                                                                                                                                                                                                                                                                                                                                                                                                                                                                                                                                                                                                                                                                                                                                                                                                                                                                                                                                                                                                                                  ..


>sp|O15265|ATX7_HUMAN Ataxin-7 OS=Homo sapiens OX=9606 GN=ATXN7 PE=1 SV=1
MSERAADDVRGEPRRAAAAAGGAAAAAARQQQQQQQQQQPPPPQPQRQQHPPPPPRRTRP
EDGGPGAASTSAAAMATVGERRPLPSPEVMLGQSWNLWVEASKLPGKDGTELDESFKEFG
KNREVMGLCREDMPIFGFCPAHDDFYLVVCNDCNQVVKPQAFQSHYERRHSSSSKPPLAV
PPTSVFSFFPSLSKSKGGSASGSNRSSSGGVLSASSSSSKLLKSPKEKLQLRGNTRPMHP
IQQSRVPHGRIMTPSVKVEKIHPKMDGTLLKSAVGPTCPATVSSLVKPGLNCPSIPKPTL
PSPGQILNGKGLPAPPTLEKKPEDNSNNRKFLNKRLSEREFDPDIHCGVIDLDTKKPCTR
SLTCKTHSLTQRRAVQGRRKRFDVLLAEHKNKTREKELIRHPDSQQPPQPLRDPHPAPPR
TSQEPHQNPHGVIPSESKPFVASKPKPHTPSLPRPPGCPAQQGGSAPIDPPPVHESPHPP
LPATEPASRLSSEEGEGDDKEESVEKLDCHYSGHHPQPASFCTFGSRQIGRGYYVFDSRW
NRLRCALNLMVEKHLNAQLWKKIPPVPSTTSPISTRIPHRTNSVPTSQCGVSYLAAATVS
TSPVLLSSTCISPNSKSVPAHGTTLNAQPAASGAMDPVCSMQSRQVSSSSSSPSTPSGLS
SVPSSPMSRKPQKLKSSKSLRPKESSGNSTNCQNASSSTSGGSGKKRKNSSPLLVHSSSS
SSSSSSSSHSMESFRKNCVAHSGPPYPSTVTSSHSIGLNCVTNKANAVNVRHDQSGRGPP
TGSPAESIKRMSVMVNSSDSTLSLGPFIHQSNELPVNSHGSFSHSHTPLDKLIGKKRKCS
PSSSSINNSSSKPTKVAKVPAVNNVHMKHTGTIPGAQGLMNSSLLHQPKARP

PFVM
     0000000000000000000000000000000000000000000000000000000000000000000000000000000000000000000000000001111111111111111111111111111111111111111111111111111111111111111111111111111111111111111111111111111222222222222222222222222222222222222222222222222222222222222222222222222222222222222222222222222222233333333333333333333333333333333333333333333333333333333333333333333333333333333333333333333333333334444444444444444444444444444444444444444444444444444444444444444444444444444444444444444444444444444555555555555555555555555555555555555555555555555555555555555555555555555555555555555555555555555555566666666666666666666666666666666666666666666666666666666666666666666666666666666666666666666666666667777777777777777777777777777777777777777777777777777777777777777777777777777777777777777777777777777888888888888888888888888888888888888888888888888888888888888888888888888888888888888888888888
     0000000001111111111222222222233333333334444444444555555555566666666667777777777888888888899999999990000000000111111111122222222223333333333444444444455555555556666666666777777777788888888889999999999000000000011111111112222222222333333333344444444445555555555666666666677777777778888888888999999999900000000001111111111222222222233333333334444444444555555555566666666667777777777888888888899999999990000000000111111111122222222223333333333444444444455555555556666666666777777777788888888889999999999000000000011111111112222222222333333333344444444445555555555666666666677777777778888888888999999999900000000001111111111222222222233333333334444444444555555555566666666667777777777888888888899999999990000000000111111111122222222223333333333444444444455555555556666666666777777777788888888889999999999000000000011111111112222222222333333333344444444445555555555666666666677777777778888888888999
     1234567890123456789012345678901234567890123456789012345678901234567890123456789012345678901234567890123456789012345678901234567890123456789012345678901234567890123456789012345678901234567890123456789012345678901234567890123456789012345678901234567890123456789012345678901234567890123456789012345678901234567890123456789012345678901234567890123456789012345678901234567890123456789012345678901234567890123456789012345678901234567890123456789012345678901234567890123456789012345678901234567890123456789012345678901234567890123456789012345678901234567890123456789012345678901234567890123456789012345678901234567890123456789012345678901234567890123456789012345678901234567890123456789012345678901234567890123456789012345678901234567890123456789012345678901234567890123456789012345678901234567890123456789012345678901234567890123456789012345678901234567890123456789012345678901234567890123456789012
     MSERAADDVRGEPRRAAAAAGGAAAAAARQQQQQQQQQQPPPPQPQRQQHPPPPPRRTRPEDGGPGAASTSAAAMATVGERRPLPSPEVMLGQSWNLWVEASKLPGKDGTELDESFKEFGKNREVMGLCREDMPIFGFCPAHDDFYLVVCNDCNQVVKPQAFQSHYERRHSSSSKPPLAVPPTSVFSFFPSLSKSKGGSASGSNRSSSGGVLSASSSSSKLLKSPKEKLQLRGNTRPMHPIQQSRVPHGRIMTPSVKVEKIHPKMDGTLLKSAVGPTCPATVSSLVKPGLNCPSIPKPTLPSPGQILNGKGLPAPPTLEKKPEDNSNNRKFLNKRLSEREFDPDIHCGVIDLDTKKPCTRSLTCKTHSLTQRRAVQGRRKRFDVLLAEHKNKTREKELIRHPDSQQPPQPLRDPHPAPPRTSQEPHQNPHGVIPSESKPFVASKPKPHTPSLPRPPGCPAQQGGSAPIDPPPVHESPHPPLPATEPASRLSSEEGEGDDKEESVEKLDCHYSGHHPQPASFCTFGSRQIGRGYYVFDSRWNRLRCALNLMVEKHLNAQLWKKIPPVPSTTSPISTRIPHRTNSVPTSQCGVSYLAAATVSTSPVLLSSTCISPNSKSVPAHGTTLNAQPAASGAMDPVCSMQSRQVSSSSSSPSTPSGLSSVPSSPMSRKPQKLKSSKSLRPKESSGNSTNCQNASSSTSGGSGKKRKNSSPLLVHSSSSSSSSSSSSHSMESFRKNCVAHSGPPYPSTVTSSHSIGLNCVTNKANAVNVRHDQSGRGPPTGSPAESIKRMSVMVNSSDSTLSLGPFIHQSNELPVNSHGSFSHSHTPLDKLIGKKRKCSPSSSSINNSSSKPTKVAKVPAVNNVHMKHTGTIPGAQGLMNSSLLHQPKARP
Predicted Results:
   1 ..ACAYAABAACSAAAAADASJZAAAAAAADAAAAAAAPBCCSBSAAPAPSSCCSSACPYAJPPYAYAAVWYAAAADYVDJWCJCCVYAAAAWWEEAWAAAAAPSWZQYJJABVAAAAACPVSBAEEEWAAACSBSSRPSSAAAARABVBEDDDAAWEACAADAAAAPADAVWVYYSYYWWCWJAEAWWCAPAAAQADPVEEACVAVWASABSPVBJWVJAAPCZAAAJBEPPSCVVVVVAACADACCCSSLEBABJSCDBRSAJAVBEAAAEAWCWPVBPEAAAPCJAJWWWWPCWWCWCBVAACABZPSCCCCYWYASWSAJLAV$VVAACBDAAAAESBVDAPSEBBBVAVVPCEVPCZJBVAPCAAAVAAAVAPYPAQWDAAADAAPDJADAAAADJVAVDCCSWVAAJPCCFCYVEAFWWWWJYPJYWWJRREWAEACCSBCJPVJJCWCCJPJJJAACPWSWEWCCVCBWCJWCCESFAVYAAAWYCQPRAJJASACAAAWQEEEVQAAAWWSDESRPYPYSVAACEDEWBVWCADWAAAPAWBDDADAAADDAAAPCWSBBPWEPAAAAJYAEWSYCSBEBWSBAAAAAAABAVPSSAJAZBSECVABPJYCYQBBCAAVEWAJQWJCBWWWWWWAYEBEAJJYPVWVAYVWVJSCYAASVYWWWAAAPAPAJBCDDACVSWVAAAAAJVAAWSCPYWSJDJSRVAEVEYVJJJJJJJJEAAJAAAAVAAJADWJPPCYVBJWCSEPPWSRLAEEDWAVAARVVLJPQCPWCSBSCYJAAAAAAAEBCWBJJAEEBEPADAWEEBAWSBJDJBPYAQQAJBYAABJARSCAAJVJVAAAAVSAVVSVSAJAWRVP$BBEBYZVPRWSSZYAVAAAAYAWBCSA..
   2 ..YJWAPJAVRPCVDDDDAQCPAEDDDDEYADDDDDDJBVBYCWB   V CCSYVBJFBWDCJWZJBYYBAACDDDCQAJBCSWSSZ JDQS  C JA SZDEBYCCDSBAPWZJJDCPA $YWDW  A   WBS  SW   P ESB  E    EECW  DEW  D   A AJDCCYAJBCYBWYAEBEPVCCSPC$PZEUJ SSSRAHYREAECJAJJAPDAJVDDDAJBW$EZ       AVA VYW EEC JJEWVELE  DYPDABDPAECY W ERRWDDLWAPV    ABSBWCSEZPBAJEPFJBWWWSVADWPC PEQYPAADP VAVCYJARSYA   BWJEYDJC  BSCWAE   JVYPJDEDJWSSA CYBABEDAV JJADASJBD PYJA YSBCCDYP WV SCPAPA     ZASAECYZAPCBABBW SVWWSVPYJ  AJ   YWJSCJSLC  W C JVPVSSBCWAASVJBZAAAYCYBPJYADPSB   LP    SCAJ R JAWJAYCWWDEBBJ  A AJDDJ DESA DAJJD  DD W CCJVJVCCBCERP J  AJJCCB  VEEEBEDBDEWBJEAEBJYJ CSSY$ABPBCAPPEPEALJBCAERWC       WAEWPAAVJYAWZQSJAEWSC S  W  AYDVAJAJAEE JJBBZV     YAYBVVCPYBAB PWAASBABDEAAAAAAAAAAY   PDD   YD JW  Y W AJAJ EAAWSV   JPBDDBD   C PYYC CSCPZBPJD Y  RE  CAAVCPSBWC B   APACS ZC$JQV  SPC CJABWSBS  CBAAPDBDAJEDCCSAEEWJEAYS  B Z PCSCYJBVDADJPDA BES P..
   3 .. ACWBDEWCWRWEWPBEVADYCBBSVDDPSSSSSSPECJZBP    D  WWWW PRJVPVS DBABCPVDDJSCAVPCVBRPWPC DEPD  V     CSVJRDPA CBJPADDWDRE AVA A  E    W   C      DB         DBB  PDA  J     JAJBW SPPFSCSES A VDZSJJRYBCC$V P JEQZCEABWDPVABPDEDBSYPYEVVQWYV       VP  W L JS   PSEJRAS  QPDWJSPDLLP    YDAVYVEJVC     CSCCVBAWU  RWUYAAJSSSVAZEDJY QADAJYBJB ASJWZDBBJ     VPCSASYE  SCABDS    AZD PSESJCQV  JVEDDJQY DPDBJYSD  WW B P CJ BV  A   BA DJ      Q JCSVS CRSJEYB J  CCPWBS       JJACSC SS  S D AYC WBALPWZBYBAADYCWJQPDBWBJJDC    Q     SEA   QQVABADZ VASVP     BEC  BAAV  V B        SWEYZRPSSLDB     YPSBA    SRVRDPVBABACVWDEPAA   CJ BRLSDJASAWDSDVJAJAASW       VVPBYVVJSJVCYVYPWPCRV       BDBESDDFPPB PP PBP     DVESECQACSZE ASJJYS PJBJVVVVVVVVJD            BB    B CES  J VP     SADASJB   P ZAA   W SVYDDJ     L  VBEDDAAUAD D   SYC Z A CBAC    Y VPDEPEEA  PWCBEJEVBBVJA BDPBRBSDCV       YCSC VACDPVRCJ YP  W..
   4 .. PVCWEDL JB  CJJBUYADDJJVYS BWWWWWWWWYAWW        BB   QV ZYBC $CJSSACSEC E BWRWEBCVWA  WYB  B     SVBCCZYP PSDAYY SPJS YA  S           F      SV         BPA              YYF  VSJLVV  D D   YRZSJBWWWJW    DDYEZVEAEVBYDYWBVSYCVVQAWSYJ         B    S LC   AAJAAR   AVAASJVJJQA     JDDSRAPPD      PPVSP CY  SEVDSBPPP AECW CV YBJQW WEW C YEBLD         WADJZY  L  S B    BDJ AP BBV W  PAJEWEPD CA VZBDS  AP W    L J        D VL         PVCC FSE WEF W   A ARC        PWWBR P   B W   J BCY  P CDDVPPPJEPP CCDEZVRS          PB    DSCB   V WRAQ        E   B E    S         PCACSRJ WWE        VS    RZDYVBCJJEFBYEBAE P   EW YPJVJPCAVB BSCCSPD V          CSCBBPAPC CAASPCLVS       SVJDJQBPBJS QB CW       DJFPYALBCPW C VFBJ QZJ BBBBBBBBWP            AY    C ELW  D QD     WV SBLA     JZ      JBWCES     P  YCPCPDCY   W   DVE Y    FBY      WDSAC W    ZVJCEWWWABYB E WVVCW AA        VVV JJJ  BBJD  C  J..
   5 .. BDSV JP BW  SEVSJIZPSVVCE  QPPPPPPB  VBJ         V   V  S WY VYWVEEYV V S EDSPRDLEBW    J  R     WPQWBAJS APEJD   BCD C   P                  BE         R L              C    ZVEPW     C   ADLV WAEAVA    ZPCJDSWVYYPCZDJWC  SW P DVVA              P  R   EBDWBW   SQJPWRY V       ABBV JDWJ      W EAL     B PAQCZVB CBJB BZ VVBWL   D D DRV           SJ QW   W    A    WV   B DEE D  VDWLB  J    J VBC   A Y    S          Q WW         V SA WBC  VJ C   W CSR        S ZJA W   E     B FZV  C EJWCDJCBCVS  EJJEBPY          EC    AWJS     SSDY        S          V         BSCVYLV F Y        WE    JBYEBEDRDJJWBBCLC S   Y  CEWFP SWYE E  YWWY E          JFVPPAVCJ  PPVEVBFR         WPWVECVVW VW AY        WWJA$VWJBC S BVCK EAP PPPPPPPPBC            CV    E WBB    BJ      B J        V$      RACYSB          BJJBEW       V P A              WCPJ C     PWJYDYSPJ      WEYV SQ        YBD AW   W E   J   ..
   6 .. S ZJ VU FE  BBEPPQSVVEEPW  JJJJJJJC   FP         P   E    Q  CPSDVSJJ B W UJWSPFSRJY    P  S     D JAJVAV WRRVJ   JER J   R                  JA                          V      LBP     J   VEPD ZYJBWB    CVJWPRDYACWVPS  S   B B AYAZ              Y  V   R YP P     YRPP  S       EPPZ DEYS        LJY     E JCC SJE BRPC AB CJV C   J W LBD           EC  A                  D WYP P   YYJV         DER   C J               B J          B AP  ED   L P   Y EWB        Y JPB C   Y     Y LRJ  J JEESJWSYAY   W SVDJQ          BR       W        Z                   W         JWR QWQ B C        PY     DBDRCE YSLZRDJW      W  WVVWZ JLRL W  PPDZ A           LEWW WBB  DDACAJYW          YEYVD    CD WV        CYSJDSAPYP Z P WV RWL WWWWWWWWCW            VP    R SVE    D       J P        YB      WCD             QBACWS         B C              BEVS P     SSYC BPE       A P  ZP         EA CD   C R   W   ..
   7 .. W  Y Q  SJ  FVYVZVCEJYYB    EEEEEEE   VR         A   L    A  SVPCZJSW P J W  ESW        Y  W     P PQW VJ SESES    SW Z                      V                           S       S          JJYY DJDSB$    PYPAYDJDJASSAZ  J   Q                        W     AE F      JDD          BYEJ WB           BD     D D J WQJ WSQ     S Y S   V J FDC           P   B                     AW Z      R         JPW     P               J Y            PJ  L    S V   R   E        $ AAD R         S PY     VWSRCVVDF    Y  YWE           V        R                            C          YW   A S J        Y        W YS W  DZVRD         J     EFBD J  VRYP J           DDSS   P   CBSLPBE           QPZS     Q JJ        LCDPPYQVDR   Y AW SBS SSSSSSSSDZ            PA    Y VYD    P       D W         S      BJP                JDV           E              VQDV R     WCL  JJ        L               LQ SS   D W       ..
   8 ..    C    AP   CPWWWYWBPPW    VVVVVV               J        P  WSZP ZDB S B A                L       Y   SY VC CP                                                          E       V          EFC  QVSYAC    SEWDBJ SPECEYR  W   Z                              BF        VB           CUCR YF            F       C R   L Z         W     C P WSW           A   P                     PJ                  ZW      C                              W                             Y Y              A     WB  VZWW         SB           A        E                            P               Y   V        E          JY S    YFV               I WY P  WEV  P           PJCC   R   JY RFE            UBW      V YC        SEWSWJEAEV     EP ZYW CCCCCCCCS             SC        V            Y Y         P      YSV                VRA                           WWD V     YPV  PY        S               P  W    E Y       ..
   9 ..    Q     V   SWCDZVRYWWE    CCCCCC               E        S  AW E  PC W R D                            BC YD D                                                           B                  FWD   ZRPCQ    YJSBCP ZRSEBSB  Y                                            QL           SS   B             J       W W               E           J               C                     SY                   F      D                                                            R                       S  SBJI         EQ                    V                            D               W            J          V  Z     S                L J  Y   YC  B           A EE   S    L  EJ             CJ        EP        PJBDJWJECY     ZQ  C  EEEEEEEEV              S                     Z B         V        A                W J                            $  Y     DYD            C                  Y    S V       ..
  10 ..          Y   QSJ$PBBWSSY    YYYYYY               F           PU F  BP Y V Z                            $   I S                                                           D                    F    UJQS    ACBRSC  SDYDCW                                               YQ            Z   V             S       Q                 S                                                                      R      Q                                                            B                       I  WY Q         Z                     Z                            E                            R          W        W                Q L       R  L           R YY        C  YW             SS        SA        DRRBYBSWJA         S  YYYYYYYYR              E                       C                  S                Y P                               A     EEW            D                  P              ..
  11 ..              RCUC$WSLCC     BBBBBB               Y           B  J  EQ                                      V                                                             Z                    Q    VZLY    BIIPV   WWQZW                                                E                                       $                 C                                                                      L      E                                                            D                       P     S                               C                            Y                                                L                V D          Y           J QQ        R  R              WQ        $E        ZVYQBEZFQI         R  QQQQQQQQ               U                       Z                  U                  C                               I     BDB            R                                 ..
  12 ..              WQRSDEJRQQ     QQQQQQ               L           J  Q  Q                                       W                                                                                  W    YDSP    UBV J   B L E                                                F                                       A                                                                                               S                                                            V                       R     V                               D                                                                                              Y P          R           V LL        U  Z               Y        ZI         DZZVR  LL            LLLLLLLL               Q                                                                                             L     ZL                                               ..
  13 ..              YRLYRQ PRR     FFFFFF               I              W  Z                                                                                                                          B    ALR      RE Q   L R                                                                                          S                                                                                               Z                                                                                                                          P                                                                                                           Z             RR        X                           L          LF$ZD  UU            RRRRRRRR               R                                                                                             U      R                                               ..
  14 ..               LY  $ ILL     ZZZZZZ                              Z                                                                                                                             E    B Y      SU       Z                                                                                                                                                                                                                                                                                                                                                                                                                                 D             ZZ                                    R          ZLUFF                ZZZZZZZZ                                                                                                                                                                    ..
  15 ..               FQ  H FFF     LLLLLL                                                                                                                                                            V             XL       D                                                                                                                                                                                                                                                                                                                                                                                                                                 F             DD                                    U          PCXLI                DDDDDDDD                                                                                                                                                                    ..
  16 ..               IZ    UII                                                                                                                                                                                              F                                                                                                                                                                                                                                                                                                                                                                                                                                 Q             FF                                                Q  Z                FFFFFFFF                                                                                                                                                                    ..
  17 ..               UI    ZUU                                                                                                                                                                                              I                                                                                                                                                                                                                                                                                                                                                                                                                                               II                                                   $                IIIIIIII                                                                                                                                                                    ..
  18 ..               Z      ZZ                                                                                                                                                                                              U                                                                                                                                                                                                                                                                                                                                                                                                                                               UU                                                   Q                UUUUUUUU                                                                                                                                                                    ..
  19 ..               H      HH                                                                                                                                                                                                                                                                                                                                                                                                                                                                                                                                                                                                                                                                                                   O                                                                                                                                                                                            ..
  20 ..                                                                                                                                                                                                                                                                                                                                                                                                                                                                                                                                                                                                                                                                                                                           U                                                                                                                                                                                            ..


>sp|P20226|TBP_HUMAN TATA-box-binding protein OS=Homo sapiens OX=9606 GN=TBP PE=1 SV=2
MDQNNSLPPYAQGLASPQGAMTPGIPIFSPMMPYGTGLTPQPIQNTNSLSILEEQQRQQQ
QQQQQQQQQQQQQQQQQQQQQQQQQQQQQQQQQQQAVAAAAVQQSTSQQATQGTSGQAPQ
LFHSQTLTTAPLPGTTPLYPSPMTPMTPITPATPASESSGIVPQLQNIVSTVNLGCKLDL
KTIALRARNAEYNPKRFAAVIMRIREPRTTALIFSSGKMVCTGAKSEEQSRLAARKYARV
VQKLGFPAKFLDFKIQNMVGSCDVKFPIRLEGLVLTHQQFSSYEPELFPGLIYRMIKPRI
VLLIFVSGKVVLTGAKVRAEIYEAFENIYPILKGFRKTT

PFVM
     000000000000000000000000000000000000000000000000000000000000000000000000000000000000000000000000000111111111111111111111111111111111111111111111111111111111111111111111111111111111111111111111111111122222222222222222222222222222222222222222222222222222222222222222222222222222222222222222222222222223333333333333333333333333333333333333333
     000000000111111111122222222223333333333444444444455555555556666666666777777777788888888889999999999000000000011111111112222222222333333333344444444445555555555666666666677777777778888888888999999999900000000001111111111222222222233333333334444444444555555555566666666667777777777888888888899999999990000000000111111111122222222223333333333
     123456789012345678901234567890123456789012345678901234567890123456789012345678901234567890123456789012345678901234567890123456789012345678901234567890123456789012345678901234567890123456789012345678901234567890123456789012345678901234567890123456789012345678901234567890123456789012345678901234567890123456789012345678901234567890123456789
     MDQNNSLPPYAQGLASPQGAMTPGIPIFSPMMPYGTGLTPQPIQNTNSLSILEEQQRQQQQQQQQQQQQQQQQQQQQQQQQQQQQQQQQQQQQQQAVAAAAVQQSTSQQATQGTSGQAPQLFHSQTLTTAPLPGTTPLYPSPMTPMTPITPATPASESSGIVPQLQNIVSTVNLGCKLDLKTIALRARNAEYNPKRFAAVIMRIREPRTTALIFSSGKMVCTGAKSEEQSRLAARKYARVVQKLGFPAKFLDFKIQNMVGSCDVKFPIRLEGLVLTHQQFSSYEPELFPGLIYRMIKPRIVLLIFVSGKVVLTGAKVRAEIYEAFENIYPILKGFRKTT
Predicted Results:
   1 ..VAAVWYWDPQAAWCBVWDPCEPSBEESJBSYYBRPCCCWASZJSAVAAAAAAAADADAAAAAAAAAAAAAAAAAAAAAAAAAAAAAAAAAAAAAAAAAAAAABVYAPAEYCYYAPCADEAAAAAEEAWCCYAPACBJBJVAPSAWCAYYSPCYABAAYJEYASVJEEEEWBDVPRBBVADBAAAJAAJEBBVAQAYPRBEEBVDPSWEBEAWYQYJBEEVJBVPZAAAAAAAAAAAAAAAAQYPSBAAWSBWSVJEEEELSWBPRBABAAAEBBJVDAJEBBVAQJVPSBBBBVDESAEAEEWYQBWEEEVJBVAYAAAAAAAADAAAAAAAJBV..
   2 ..B  BPCC SWPDCJRS ACPYWPSBWA E   AYSWWABC  AJBSEBRDDDDBAVADDDDDDDDDDDDDDDDDDDDDDDDDDDDDDDDDDDDVDDDEDDBJPYJD DJCVCCYWPZA D Y CAPJCWSZYAWREPW YJ BCASSCVCJWAJASYSBWWCAASPAAAECBPWVWEERAADDBAVJBBESYDDCVASEBAAYJCYPAAAEASSABE  AAJWAYDDDDSDDDDDEDDDDSPSYBVWDVJDBBWSWJDYERBYWSWBVVDRADWYAAVAAERWDDSAARAVAAECPABBBBAEEAVBSDBWAWRPADBDDDDDSAB VDWQJEW ..
   3 ..A  JYZA AUCJVPSA CBW  CCSAJ S    EYSBSCS   VDAPYEBESJDCRPSSSSSSSSSSSSSSSSSSSSSSSSSSSSSSSSSSJ BVCBDQ J WZVY  S$ VAPVJSV     DSWBPYWVSDJ VWE RP   C BSWVWSVDYJJBSSAWBDAALCWLSVSBS SBESEREEDESPADC PPDADY WBEASJVBJEDDEVAPP   WCPBLPP PVDWJSJEJJEBB BBWCDDSABACJ ABV  SE VBEASEDEEBED    PVLS QACYYBEEED QRVJWERDDARYAWBJASRSBVVJ WBS  YS J BWSY  ..
   4 ..J    BV JPDWPBZD  V   LWR        PCYP      YPCJDDEBY   JBWWWWWWWWWWWWWWWWWWWWWWWWWWWWWWWWWWQ CWBVVB S C CJ  AP WVVY V      EVYPBSB B F W V        CWCWAADPJDVCPBSD  W WUBBDWY   WJDEDVWWPPBAJSA  AJESA  SL Q  JRJRUBAPSE   D SECA  YWWES  YB RWJ EWSAABBDLJPA R Y  WB EA  EAQJBDAE     S A PZPBJJDWD  WAY AS BBCLPSRAWEVPJDRWE BE    D   DDDW  ..
   5 ..P    VY  YE DE W  W   RFQ        VAFS        DVJCSVB   BQPPPPPPPPPPPPPPPPPPPPPPPPPPPPPPPPPPY DBPWJ  W   PP   U  JWD        BRB  BP D P   L        EP  CYP WQBVACCE  Y DVP RJB   AYSBREJDQYP SRE  BELJD   V A  YDLBB  CVC     DAJ   WZVBB   V  E   VJ  SCJYP C         QE  CWEBSWSP     P E SBYDDCC     BJ  L JJVPSDBWDYCJBJWY  EL    E   JPYB  ..
   6 ..D    SD  BS JW    A   WJ         ZWRL        J VYLYC   CJJJJJJJJJJJJJJJJJJJJJJJJJJJJJJJJJJJP SCJPP  D           S A        SWA   J W V   C         V  BV  SVDJWLBB  C BBC AP    LAVJS BR J  LWR  YSP E   W C  CLDYJ  JDD     AIW   EBJJQ   D  V   PL  YR  S R         SJ  WJLPCR J     B   WP CZ J     CL     S EAJJVAPBAECU    W        E CL  ..
   7 ..     W   CB B                     RB         Y WSC     E EEEEEEEEEEEEEEEEEEEEEEEEEEEEEEEEEE   EVEL  V           D J         DC     J     S             B  VCWPRP    D C D EE    RDB   VU      W   P  V     P  ABR R  BWQ     Y     J YQY      S   EZ        W         W   DLUSJS       C    W  C S     YP     A WLPLL SDC FD             L P   ..
   8 ..          W S                     VJ           E W     P VVVVVVVVVVVVVVVVVVVVVVVVVVVVVVVVVV   JSRS  P           P           L      P     F             E  CPPWC       F    Q      W   PC                      DWW    ECA     Z        S       Y                       A    SJVYL       D    C    W     DW       BZRC  DRY YC             C W   ..
   9 ..          L Y                     BE           P R     S CCCCCCCCCCCCCCCCCCCCCCCCCCCCCCCCCC   PECC  C           W           Y            J             Z  D  L        R                Z                       C       Y     V        V                               J     WCV                        S        ZWC   JP                   V   ..
  10 ..          J                       IL             V     U YYYYYYYYYYYYYYYYYYYYYYYYYYYYYYYYYY   SYJR  E           Z                                      R  L  A                         P                       S       R              L                                     YWW                                  DE   B                    Z   ..
  11 ..                                  QA                   W BBBBBBBBBBBBBBBBBBBBBBBBBBBBBBBBBB   ZQYY  Q                                                  F                                                               W                                                      P                                  CV   U                        ..
  12 ..                                                       X QQQQQQQQQQQQQQQQQQQQQQQQQQQQQQQQQQ    WUW  R                                                  J                                                               Z                                                                                         JL                            ..
  13 ..                                                         FFFFFFFFFFFFFFFFFFFFFFFFFFFFFFFFFF     IZ  Y                                                  L                                                                                                                                                          F                            ..
  14 ..                                                         ZZZZZZZZZZZZZZZZZZZZZZZZZZZZZZZZZZ     S                                                      P                                                                                                                                                                                       ..
  15 ..                                                         LLLLLLLLLLLLLLLLLLLLLLLLLLLLLLLLLL     L                                                                                                                                                                                                                                              ..
  16 ..                                                                                                Z                                                                                                                                                                                                                                              ..


>sp|P14136|GFAP_HUMAN Glial fibrillary acidic protein OS=Homo sapiens OX=9606 GN=GFAP PE=1 SV=1
MERRRITSAARRSYVSSGEMMVGGLAPGRRLGPGTRLSLARMPPPLPTRVDFSLAGALNA
GFKETRASERAEMMELNDRFASYIEKVRFLEQQNKALAAELNQLRAKEPTKLADVYQAEL
RELRLRLDQLTANSARLEVERDNLAQDLATVRQKLQDETNLRLEAENNLAAYRQEADEAT
LARLDLERKIESLEEEIRFLRKIHEEEVRELQEQLARQQVHVELDVAKPDLTAALKEIRT
QYEAMASSNMHEAEEWYRSKFADLTDAAARNAELLRQAKHEANDYRRQLQSLTCDLESLR
GTNESLERQMREQEERHVREAASYQEALARLEEEGQSLKDEMARHLQEYQDLLNVKLALD
IEIATYRKLLEGEENRITIPVQTFSNLQIRETSLDTKSVSEGHLKRNIVVKTVEMRDGEV
IKESKQEHKDVM

PFVM
     000000000000000000000000000000000000000000000000000000000000000000000000000000000000000000000000000111111111111111111111111111111111111111111111111111111111111111111111111111111111111111111111111111122222222222222222222222222222222222222222222222222222222222222222222222222222222222222222222222222223333333333333333333333333333333333333333333333333333333333333333333333333333333333333333333333333333444444444444444444444444444444444
     000000000111111111122222222223333333333444444444455555555556666666666777777777788888888889999999999000000000011111111112222222222333333333344444444445555555555666666666677777777778888888888999999999900000000001111111111222222222233333333334444444444555555555566666666667777777777888888888899999999990000000000111111111122222222223333333333444444444455555555556666666666777777777788888888889999999999000000000011111111112222222222333
     123456789012345678901234567890123456789012345678901234567890123456789012345678901234567890123456789012345678901234567890123456789012345678901234567890123456789012345678901234567890123456789012345678901234567890123456789012345678901234567890123456789012345678901234567890123456789012345678901234567890123456789012345678901234567890123456789012345678901234567890123456789012345678901234567890123456789012345678901234567890123456789012
     MERRRITSAARRSYVSSGEMMVGGLAPGRRLGPGTRLSLARMPPPLPTRVDFSLAGALNAGFKETRASERAEMMELNDRFASYIEKVRFLEQQNKALAAELNQLRAKEPTKLADVYQAELRELRLRLDQLTANSARLEVERDNLAQDLATVRQKLQDETNLRLEAENNLAAYRQEADEATLARLDLERKIESLEEEIRFLRKIHEEEVRELQEQLARQQVHVELDVAKPDLTAALKEIRTQYEAMASSNMHEAEEWYRSKFADLTDAAARNAELLRQAKHEANDYRRQLQSLTCDLESLRGTNESLERQMREQEERHVREAASYQEALARLEEEGQSLKDEMARHLQEYQDLLNVKLALDIEIATYRKLLEGEENRITIPVQTFSNLQIRETSLDTKSVSEGHLKRNIVVKTVEMRDGEVIKESKQEHKDVM
Predicted Results:
   1 ..AAEBCAAAAACJAAVAPPBEAAVCVWJBJCYCREEAAAEWCSCCCSAEYBARAAAADPYBVDAPAAYAAAAAADSVAQASJSAABADEAPJYAAAAAAAAAAAAJPSCPAAAAADAAAAAADBEVAAAAYAAJCAEADASAAAAAAAAAADAAPAJJJSBAAAAJJAAAAAAJAAAALAAAAAVAJAAAAACAAAAADAAAAJAAAAAAAAAAAAAABEBEBEAWCVVAWAAAAAEADJDAAAAYPDDDAAAVAAAEEAAAVAAAAAAAVDAAAAYJAABQAVWADAAJJAWEBAAAAEYUAAAACASDQEADPAAAAAACEAAAAAAAAAAEWRAWDBAAAAAAAAAAAAJBAAAAABAEAEWAAAAUAAESSEWBWVVEQAABEDWDAADSVAYYCYCSBECAEEBWWREAWQYBPAAJWAAAJVV..
   2 .. DPVWPDJQD RDEPSAYE WJYBZCWAWW$WSBAWW CJWCWBSY BWABAQDYJAADRY  AJBAVD DDZAAADAEEAADDJEEAEDAADDDDDDDDDDWYY D YEJVDYADEBDDDSAWZVBDWBWWAAESDEWADD DDVPREVADDACYVAVEDDBVADVDDDL AYDVJAWDDBEADARDDJVAYDSDWAQDBJ PDDDSSBDEDDDD   WWWBVABBSJADWDVDAEA AWDDCBA   D VA  D BBSWACYVDDWJAABDDD VEJAABA DADVAAW B VDVYA AVCDVWSA SAW WDDJDB AADDDDDDYDDJIBBDAA ZDDCE EDDBDPAAEEDBDARADAADDDDASVAAEBBPVEWA DVP SBJWBPAAVBVAAYB VABBWEBCEBDZ SWAJDAB   A  ..
   3 .. BADVJYDPE FRJACJ   PWRWYAASAPZAJACDJ PPF SSVC JBVWBDJWDBEAWA  SDJECZ JV  BYRJVRER WEWADDA V EBYWBVVVEDDP C ZDPSVCS PDSSEASSABJJBSJJYRBDEA DJV VCDYDDJ VEV ZPDJAEEDDDAUEWWW D ZYEDDSCWWJ QJEWPYYDCE BZS E  DWBLDDDVDJCQY    AERWBWSYVPZDJDCWD  BC ESVD     SY    DJ CJWDPCCDSDERE E YP  WDC WB WCS  S DJJJS JQSYDBDD RBD BB EJC DVSZEPBWVJQSJDLPV  D   B J BSVBSDDDEPBEDDBDS  BJQYPJJB EWE  B LE  AAAD ABR EJJPSJ  PSABJAA  WB BJWDBWA      ..
   4 .. SCSBSEVDW CEWUYE   VYELJJPWPVPBWPPBD A   RYBV VADDDUPDCPC SW  WYP D      VD DW DD JSDWB     YEZBE   J P    AYQD LE   VJVEEJBDR  AP PEVBBR P W PJWQEZB WSC  YSBDPWEPPVB L D P BBWPEBJEBP DS VCCDVSR DJD     B W C  R  EJ    BVSD JCADCYBVJSS        E      BZ    LS JBDZYY CB WD  R  B  S D BE EPB    YBBVL QJB  AR  AD  AS VSD  SEV WSJDYPCSAAYC        Z PJF DJBBBDEYEBVYD  EWYCYCYJ AAS  P V   BDQ  EVJ AAEZBV  D DJSSP  YY PPBWW J      ..
   5 .. PDEAWP JC  YDY     JCBZCDSDEAVEEWSEE J     DB  VEEEJYVFVD YD    W S      DW    RL P RY      BSCVJ   C C    BCC   L   WVQBD$$SC  CB  DDAWB   Y J BVW L   W  B E VVJQS D     W SZDWLWSJCB  B BDWWPVB E C     S   V     BS    FLV   J PSJEWBBB               WD    RW VYPJDQ  P BE  Q  D  C W  R DED    RQDCB  PJ  D   B   J  DCE      BESECWBUJDJE        D  DC BEV PWC YVW    VCWPC  R R R  Q     RYE  Y S CZW  A  W RSVDE   C WAJEE C      ..
   6 .. YBAEC  C    SC      EPPSVBCBB JPDWCV F     RR  PFLVCSZRS  J     Y B       C     E S LZ      CWEES   B      DPS         BWPBW P  W   BR  C       YDC E   J  E A SB  B J       EDPES EDDS  C CSPBJRC         P         J      AW   Y WRPVPWJC               JP    S   PVEWV    LW  C  J  P      PSE    ZSIW    D  L   D   S    S      ECBBBOP PSBB        Y     EV  SEJ CW     SSPBD      A             B E JDP       J  CJ   $ VSEBQ P      ..
   7 .. LQJ D  S     Q      RJS  YP   LBL JP S     YP  CJ SV JB         C         B         B       PJRJ    V      JJ          J VLD E  D   S   W        EF P      L D BL    P       RERSC BVY   D  YRSBBD         V         V      PD     BB Y EYR               CQ    W   D PSP    SV  L            QD       WP    W          Y           JWEFWJV REEJ              WR  CQS Z      WVJJW      B             R Y RSV          RL     ZLSSP S      ..
   8 .. VVY B  V     S      SC   CR   SYR L         J  D  Y$ RS         E         J                 RYW     Y       W          P  RE D  E                JJ R      S W CJ            WJ JY  C    E   ZZ ZL         E         Y       A      J     D               Y           QZ      C  S            YW       PD    Y          L            JPCE Y  W P               P   SV        YE$D                         V D          EB     AVRV         ..
   9 .. WY  $        $      PL   RV   YA  P            E   H B          I         Z                 SL                         R     L  F                 L S      W   YS            YC V            D  YW                   W       J      D     F                           V       S                         Q    P          R            RCWP    C R               W   JY        RZBV                         W            YD     CCC          ..
  10 ..                     DF    Y    C  S                  P          V                                                            S  V                 S W           U            JI              J  W                    $       Y      E                                                                                   V            VRSV      D               Y               EW                         Z             R      ED          ..
  11 ..                     VS         Z                                                                                             V  Z                                            VL              E                       P       P      V                                                                                   D             VJS                      S               SZ                                       S      RL          ..
  12 ..                      W                                                                                                          P                                            UW                                                                                                                                         C             LZR                                      VL                                                          ..
  13 ..                                                                                                                                                                              C                                                                                                                                          E             Q                                        C                                                           ..
  14 ..                                                                                                                                                                              F                                                                                                                                          Q                                                      D                                                           ..
  15 ..                                                                                                                                                                                                                                                                                                                         Z                                                      R                                                           ..
  16 ..                                                                                                                                                                                                                                                                                                                                                                                Z                                                           ..


>sp|O00411|RPOM_HUMAN DNA-directed RNA polymerase, mitochondrial OS=Homo sapiens OX=9606 GN=POLRMT PE=1 SV=2
MSALCWGRGAAGLKRALRPCGRPGLPGKEGTAGGVCGPRRSSSASPQEQDQDRRKDWGHV
ELLEVLQARVRQLQAESVSEVVVNRVDVARLPECGSGDGSLQPPRKVQMGAKDATPVPCG
RWAKILEKDKRTQQMRMQRLKAKLQMPFQSGEFKALTRRLQVEPRLLSKQMAGCLEDCTR
QAPESPWEEQLARLLQEAPGKLSLDVEQAPSGQHSQAQLSGQQQRLLAFFKCCLLTDQLP
LAHHLLVVHHGQRQKRKLLTLDMYNAVMLGWARQGAFKELVYVLFMVKDAGLTPDLLSYA
AALQCMGRQDQDAGTIERCLEQMSQEGLKLQALFTAVLLSEEDRATVLKAVHKVKPTFSL
PPQLPPPVNTSKLLRDVYAKDGRVSYPKLHLPLKTLQCLFEKQLHMELASRVCVVSVEKP
TLPSKEVKHARKTLKTLRDQWEKALCRALRETKNRLEREVYEGRFSLYPFLCLLDEREVV
RMLLQVLQALPAQGESFTTLARELSARTFSRHVVQRQRVSGQVQALQNHYRKYLCLLASD
AEVPEPCLPRQYWEELGAPEALREQPWPLPVQMELGKLLAEMLVQATQMPCSLDKPHRSS
RLVPVLYHVYSFRNVQQIGILKPHPAYVQLLEKAAEPTLTFEAVDVPMLCPPLPWTSPHS
GAFLLSPTKLMRTVEGATQHQELLETCPPTALHGALDALTQLGNCAWRVNGRVLDLVLQL
FQAKGCPQLGVPAPPSEAPQPPEAHLPHSAAPARKAELRRELAHCQKVAREMHSLRAEAL
YRLSLAQHLRDRVFWLPHNMDFRGRTYPCPPHFNHLGSDVARALLEFAQGRPLGPHGLDW
LKIHLVNLTGLKKREPLRKRLAFAEEVMDDILDSADQPLTGRKWWMGAEEPWQTLACCME
VANAVRASDPAAYVSHLPVHQDGSCNGLQHYAALGRDSVGAASVNLEPSDVPQDVYSGVA
AQVEVFRRQDAQRGMRVAQVLEGFITRKVVKQTVMTVVYGVTRYGGRLQIEKRLRELSDF
PQEFVWEASHYLVRQVFKSLQEMFSGTRAIQHWLTESARLISHMGSVVEWVTPLGVPVIQ
PYRLDSKVKQIGGGIQSITYTHNGDISRKPNTRKQKNGFPPNFIHSLDSSHMMLTALHCY
RKGLTFVSVHDCYWTHAADVSVMNQVCREQFVRLHSEPILQDLSRFLVKRFCSEPQKILE
ASQLKETLQAVPKPGAFDLEQVKRSTYFFS

PFVM
     000000000000000000000000000000000000000000000000000000000000000000000000000000000000000000000000000000000000000000000000000000000000000000000000000000000000000000000000000000000000000000000000000000000000000000000000000000000000000000000000000000000000000000000000000000000000000000000000000000000000000000000000000000000000000000000000000000000000000000000000000000000000000000000000000000000000000000000000000000000000000000000000000000000000000000000000000000000000000000000000000000000000000000000000000000000000000000000000000000000000000000000000000000000000000000000000000000000000000000000000000000000000000000000000000000000000000000000000000000000000000000000000000000000000000000000000000000000000000000000000000000000000000000000000000000000000000000000000000000000000000000000000000000000000000000000000000000000000000000000000000000000000000000000000000000000000000000000000000000000000000000000000000000000000000000000000000000000000000000000000000000000000000000000000000000000000000111111111111111111111111111111111111111111111111111111111111111111111111111111111111111111111111111111111111111111111111111111111111111111111111111111111111111111111111111111111111111111111111111111111111111111111111111111111111111
     000000000000000000000000000000000000000000000000000000000000000000000000000000000000000000000000000111111111111111111111111111111111111111111111111111111111111111111111111111111111111111111111111111122222222222222222222222222222222222222222222222222222222222222222222222222222222222222222222222222223333333333333333333333333333333333333333333333333333333333333333333333333333333333333333333333333333444444444444444444444444444444444444444444444444444444444444444444444444444444444444444444444444444455555555555555555555555555555555555555555555555555555555555555555555555555555555555555555555555555556666666666666666666666666666666666666666666666666666666666666666666666666666666666666666666666666666777777777777777777777777777777777777777777777777777777777777777777777777777777777777777777777777777788888888888888888888888888888888888888888888888888888888888888888888888888888888888888888888888888889999999999999999999999999999999999999999999999999999999999999999999999999999999999999999999999999999000000000000000000000000000000000000000000000000000000000000000000000000000000000000000000000000000011111111111111111111111111111111111111111111111111111111111111111111111111111111111111111111111111112222222222222222222222222222222
     000000000111111111122222222223333333333444444444455555555556666666666777777777788888888889999999999000000000011111111112222222222333333333344444444445555555555666666666677777777778888888888999999999900000000001111111111222222222233333333334444444444555555555566666666667777777777888888888899999999990000000000111111111122222222223333333333444444444455555555556666666666777777777788888888889999999999000000000011111111112222222222333333333344444444445555555555666666666677777777778888888888999999999900000000001111111111222222222233333333334444444444555555555566666666667777777777888888888899999999990000000000111111111122222222223333333333444444444455555555556666666666777777777788888888889999999999000000000011111111112222222222333333333344444444445555555555666666666677777777778888888888999999999900000000001111111111222222222233333333334444444444555555555566666666667777777777888888888899999999990000000000111111111122222222223333333333444444444455555555556666666666777777777788888888889999999999000000000011111111112222222222333333333344444444445555555555666666666677777777778888888888999999999900000000001111111111222222222233333333334444444444555555555566666666667777777777888888888899999999990000000000111111111122222222223
     123456789012345678901234567890123456789012345678901234567890123456789012345678901234567890123456789012345678901234567890123456789012345678901234567890123456789012345678901234567890123456789012345678901234567890123456789012345678901234567890123456789012345678901234567890123456789012345678901234567890123456789012345678901234567890123456789012345678901234567890123456789012345678901234567890123456789012345678901234567890123456789012345678901234567890123456789012345678901234567890123456789012345678901234567890123456789012345678901234567890123456789012345678901234567890123456789012345678901234567890123456789012345678901234567890123456789012345678901234567890123456789012345678901234567890123456789012345678901234567890123456789012345678901234567890123456789012345678901234567890123456789012345678901234567890123456789012345678901234567890123456789012345678901234567890123456789012345678901234567890123456789012345678901234567890123456789012345678901234567890123456789012345678901234567890123456789012345678901234567890123456789012345678901234567890123456789012345678901234567890123456789012345678901234567890123456789012345678901234567890123456789012345678901234567890123456789012345678901234567890123456789012345678901234567890
     MSALCWGRGAAGLKRALRPCGRPGLPGKEGTAGGVCGPRRSSSASPQEQDQDRRKDWGHVELLEVLQARVRQLQAESVSEVVVNRVDVARLPECGSGDGSLQPPRKVQMGAKDATPVPCGRWAKILEKDKRTQQMRMQRLKAKLQMPFQSGEFKALTRRLQVEPRLLSKQMAGCLEDCTRQAPESPWEEQLARLLQEAPGKLSLDVEQAPSGQHSQAQLSGQQQRLLAFFKCCLLTDQLPLAHHLLVVHHGQRQKRKLLTLDMYNAVMLGWARQGAFKELVYVLFMVKDAGLTPDLLSYAAALQCMGRQDQDAGTIERCLEQMSQEGLKLQALFTAVLLSEEDRATVLKAVHKVKPTFSLPPQLPPPVNTSKLLRDVYAKDGRVSYPKLHLPLKTLQCLFEKQLHMELASRVCVVSVEKPTLPSKEVKHARKTLKTLRDQWEKALCRALRETKNRLEREVYEGRFSLYPFLCLLDEREVVRMLLQVLQALPAQGESFTTLARELSARTFSRHVVQRQRVSGQVQALQNHYRKYLCLLASDAEVPEPCLPRQYWEELGAPEALREQPWPLPVQMELGKLLAEMLVQATQMPCSLDKPHRSSRLVPVLYHVYSFRNVQQIGILKPHPAYVQLLEKAAEPTLTFEAVDVPMLCPPLPWTSPHSGAFLLSPTKLMRTVEGATQHQELLETCPPTALHGALDALTQLGNCAWRVNGRVLDLVLQLFQAKGCPQLGVPAPPSEAPQPPEAHLPHSAAPARKAELRRELAHCQKVAREMHSLRAEALYRLSLAQHLRDRVFWLPHNMDFRGRTYPCPPHFNHLGSDVARALLEFAQGRPLGPHGLDWLKIHLVNLTGLKKREPLRKRLAFAEEVMDDILDSADQPLTGRKWWMGAEEPWQTLACCMEVANAVRASDPAAYVSHLPVHQDGSCNGLQHYAALGRDSVGAASVNLEPSDVPQDVYSGVAAQVEVFRRQDAQRGMRVAQVLEGFITRKVVKQTVMTVVYGVTRYGGRLQIEKRLRELSDFPQEFVWEASHYLVRQVFKSLQEMFSGTRAIQHWLTESARLISHMGSVVEWVTPLGVPVIQPYRLDSKVKQIGGGIQSITYTHNGDISRKPNTRKQKNGFPPNFIHSLDSSHMMLTALHCYRKGLTFVSVHDCYWTHAADVSVMNQVCREQFVRLHSEPILQDLSRFLVKRFCSEPQKILEASQLKETLQAVPKPGAFDLEQVKRSTYFFS
Predicted Results:
   1 ..EAAAWCABAAAAAAAVCCSCCACVJCYPAQACAUYYAYWVSPCSAJAWWJAWBDDDJEBAAAAAAADBABAADAJLAAEDBAAVDVASAWESAYWSAAVYYCCWPPWWYAAVAYACSVVADAAAAWCVAAAAAAAADAADAABBVACAEPEAAAAAAAWWCAVJAADYDWAAAASCCAWWYWPCWWAAAAAAAACJZAWECBBBAWCCCAJJAAAJWAPDAJAAAAAEAAAAPWAAAAPAAAAABAWAAJAAABADJJYAAJAADAAAAAAQAD$AAAAAAABABAAQYBCYDJVAAAADAASADDYSWWVAAAAAAAAAAAAAAABAAAAABAAAEAZVAYAAAAAAEAADJVAJECCYCCCYCWSAAYAAAAAWSVYYJEWVWLCSACSAAAAAAAAAAAAAAAAAJEEWCBESPACWPCAAAAAAADAAAAAAAAAADAAAAAAAAAVPDCPAAAWSYAESAAAAAAAPWVAAAAABVJAAAAAWCCPYCDAADAAAAAAJBDAAAJBEAAASBWWJEAAAADDAAAAAAAAAAAAYPWPSWCCADAVYEQAWBAAAAAPCCPWABAAVAASAAAAAAADAEEWSVJPDVWWWPAJEESJJAEEEDVAAYPSECALWCJAAADAEAADABPBVJEYSEACPSSVPCCCSAJCYDCAAAAAAVWSVJEAEVABAAAAAAAAASCYAAAADAJAAAAAAWAJBELC$YWAAAARAAAAEACCCVAQSWCCCYAJACFCYESACYEAAAVSYAAAAAAAADAACDAAAAVAAYWAAAAADVAAEAAVAAJSEEEEELEWZAYJRWSVJVAAPYJPEAAAAAAAAASBSWSRWAJAPAAWABEEAADVAAAAYPCAAAACUAAAAAAAAAWAEDDPSDAASBVAAAYRBVAAAAAAAAAAAAAAVYAJVAAEDBAPSEBEJYZCBAAAAAAAAVEJZYAAAABPBWCVAPCYBESAEAAAAAAAAAJDAAVQAJSAVAAEBYAWWEBDAELDAAAABYAAABQAPAAABADAAAAAAAJSBAAADDAAEAEAADEAAAAADDJVPAAAAAJEAAAAAAABAAEAPBSWDJLYQAPSEESBBEEWAAKADQYVPJRADBBBBEAAWAAPCAVADAPDABAAAABAAWDVYAAAAAAAAAAAYSEEEEBB$YJLEWAVASAVAAAAAAAAAADSDAAPYVDADAAALEAAAPAAJVAJAAAAAAJAADAASCSCCAVAEVAAAAVAVAE..
   2 ..      SZQSYDJDP   ASJSVSVJZAVJQYVW  DESEAVBY YD  QYAW   SRAVWDSJWWAABADBAWACPCBBEJVEAASEPAS C VQSJFVZ SA    AVJBYS P   D DDDWAWAD       ADDASWEWSDVYAALWCBPDWSCEWSAADDA A  S  C   AJCS W  DDDDDDDDASYJJBDAAYBAPWPY    PAPWYAQABDEPDD   DBPDVPVWDDDBBEEPS PVD AEAEAA DASDAE JB DAJARDDEVEDEA SVBPAAWVAAADEDDA  DDAJZ  AYDDDDDCDDDPCDVSWDBDDEDADDEADYADZSEDDDDASDWWCBBCPVSVPWCEV EWADDDJDAEZASBBBWSEBEWBEBW DD EDDD  RBSPV  WBAEVWCRWCCQYDPED EAJCEDDRDDDJAB DD DEDBASAAAP WEQ JSWEJ VEDB JAVJESCAAADDDDJYDYACWEVCAYDDDCCVABJD AAAVDVAAUYAADDDD A DD BEDECPZVBWC RS SYADARBASPCDDDDDWW CCCADBA WASDDDESDA$JPLCSVVWA   WWCWWAVBE$BRJSVPZWABYJEASSVBDALADDAJAJVJEBZAYEVEDB   DSCBSJCAVZVSDEPYAAAVVJYAJJJDD DDDD PWC JDDABADDDVBDP      WSJCBCDABDBDAQASS DVYPSWWCCSPSCWSPBBJVJPCWCADDDEDDDDDA  AAJDC A WASDDDDDAEWZDQDPDDPABR LRERAYQAWS RBWYJJV AYAJDWDDDBDSVJECCSYZQJADVADEBABCBAJDEDADBSWDDAAEBDJDC DDADAAAJCSESAE  Z PWWYD D D  DDVDDDDAAYBBDWAA PAVVEBWCASV EBDDJDPPWAWEDWWJAEWPVADASDAVAVABWLJBYDJPAADDAVJBV WDDAWADL YPADAWEDBDDVRYBYYPDCDBYADADDDDDVDPCVEBVAADDDPABDAADB Y  APPYYSDDDAADPDWSDDA EVPBEEELSCSDSBRBWBVCJBABEREBWUCWZDB ESE  YVLDVCSJEDJ QQPADD SJDBAAAADJ  LSD    QABRDURWWA E  CYCAJAVD D  DDJ AAZB  SWABAVDDAABDDJBVPABADDDZDDAEBADEDWCWYJACWWDEDEAVB A..
   3 ..      WAWCEFDVQ     YPPYAPJEYLC ZY  JWCAVSJ   W    CE   EAESJWBDSDPESE WWCPAWJAEWWERWDDAJD  W ZPCPPPC BC    SDPJBB S     EBWSVJP         VSCR   AWAVSBJBDECE DSBLRCYVCV    D      VAVV      JESVCWWCCYFSWESWDJ SJ     BEBVS PDVSPBS    BDJBPVBBCV DEADA   WV PRJAWE  D  RB  D  PSBY JDEBE E EDSJSPVRVDWWBJWE  AW  A  S$CBJ  D   DD WCBSVEBDEDEEDYPJCPAEJWWRCBB BCSEWWWSCBWYBRC VCPPPV VEJWDPAJECCYAYDSAED    JVVC  DSOJD  C BSYVJVVBJWEEJB   YDJVE BZ  E    J JW  YAJPDE VBR PREYP  D D AYDEDBBDDVVJCBBPWA P$ABDEJPBSPWYDAB  SEBD BDJPADDLB J       YE VWPBSEY    Y    ADPWBDZCCRBC  VY D DE DDRYY DD B AV   CJY     VVCVCSAWRLBP WJVABWRBPBWCYW EFRWCPDJCCWAACBLSAJE      REWPVPACPDBWCSCBDABBBSSSV J SBJC J W  JVVDSBYPWZ A      SVBDEDJEEEDWDBQWB   CCLYASYVJVPSCCREB BCJPACAJWDEJVE     SSWD Y DZABWS ELADYA$ A Y E      L  PZBW B   B S DAPSPDWESWEWBVDBEBCYYS BDB SABWSEJVWSVD WBBPEDEDWSDJV JEBVJWVWV QJ    Y BYS          JVCYWJPSPY CJE VJ Y PQWSJA  CC WEDCRBJVCBEEDYACYWYJWB CDQDWBDDCDEDYP C DASPE AWEBEW J AAESCAAEDE WJEJBDVPDYYDECEJFVPYC B CS     $VJBVVJBEV C   DQARD    D DBPYEBD  YYZJ ASBWZWVWEADE VBSJESJ EYQSFAVD V    SBVBWW S  A DAVJC  VAPEDPD     CZ     AEWSBAEJZ  B  ZBVCEB          WLBJ  VACDPDYCBDVW A JB D VSVYV DWPWBWCBBPFCYJJ BDC EJD  ..
   4 ..      QCDYSCE D     VFWCZVAYWPJ E    BBYJD          A   BBWBBSDV BJWDS LV DYBSJA D SV BVV     CCPBB   WB    BYSAWE       VWBVSAW         PBBD      QYCRPBSDW JB FVBZWEB           C  Y        RQVVDBVZCAAVESV  FY     SBDCZ  YEEW Y     AAYBJYC     D D   P  DJWVV      BD  W  YPYJ SPBDJ D C DBDWFZWQBCD B   B   P  CAPEP         QYPRWSEJPE BBWCVDSSBBVVERDC QLBCEAJ LSJBWSY B V YP EDBBWVPSAPVA BPWJJJ      PW   RWDJ    DPRASBSPVVJSZD   JEBJJ D          BC   YEEED DJB SAB    S J CSSRSCDSSWL $EYBAV  FBYLPBJPPDEAVEE  BCRL D WVP BES         RW EDVJAAS    Z     ADVESJJJCCJ  JP V    CCJCC    E BJ   PE      BWBBJAELSW B  D   PSRSD WW  BSB WYBDBYSPCWCBWJWC       SC WWPPWREBWZBJ PSWVECV    YSE  R B   LPVDEZ DD B      AZAEQJSDD P   S     BJBFFVVY WJBR CVA AD CSWW BWBPYS     RDV     VRYQJ  EBBD Z S P R         WE B       W P ZBVBPWCDWC  Y ACP V  P   AR EBAPC J   AZDWRJJWVJSZ   VDBD PBY CY      ZQJ           PEBLWVBSA VPS SC A JBVCWE     SWQSPPCWEDJLEBEBSBVW V  CYBDEQSE VB W J   YWW EE VAP Y DJSEBE  WR DVJWWWY YRJWW QC SEJW A Y      ZJDR WS  W D   A SC       JVSDLWE  QJ Y  CF BBJJBWL  JSVCJBA APVAJ A  R    WYBS   B    J YS     QJYBY     WC     PYRBAB Y      JJDDB           BEEE  APEVJ VWJJD  D      RJSDJ PVDJVBPSESZPWEB  J  WD   ..
   5 ..      PYCDBLS J     SJAAYWCCJS$ P    CYPCA               DLESCJB JEDWR EB SRLYRR   WE $WW     PAYWW   AD    V WWCR        HJCDBJ         JFJ       JV SJYJVP QA BYD PBY           J           B WE WU$RRBWLZJ  Y      QCAJB  BDYQ       EDSJ CE       E      EYBDP             JY A   W   W A JWERLSYZJYC E          PSVJB         JWJEEVSRSJ JSDQDFY WV BC     P  ABS RWFSJF  W B  E   YS ASR BEJ VBEWDC      W    EC       DCEWCALABDR L   EWDBB E          SU   J  WB BU  VCL        EDBSBD  WEW P CSYB  PP V WCVJWDD JV  CR     PDV  S           B JEYPEFL    F      WJVYWPV E   RB Y    VJDWS            Y      EZD DCRB   E      JDDBL      WD PBECS P WAFADEBR       VE SY JSWYJBLEW YA ZDBP     VW        PYRPP   J Y      QAEBSVRBW            BV SRWA BLZ  AS  Y  JYDP JB CEL      W      DPSVB  WWEV D J S B          P J         S CZB BCVJB   A BV  A      D  RJVWS C   DYESSBBJDV     RSWP B   JP      VAC           WVS CWCWD DS  YW B  A Y$P     BRJYBYVDWSD VAJJF    W  BEYESVFW B         EB SR YVV B PDB DD     ADSEVJD AZCEJ SV  BVE Z           D L   E V      V       S J PV   AW R   R AAYLF B   WWSUDE PC WB    C    VSJ    W    Y D       PJW      DD      WYW           WJJV              V   BS S  E            WEBEQ  DYZ DJPVB  PVS  Z   Y   ..
   6 ..      EJPPWSB V      CFWDZP SVE $    S WWJ               S DVEEW  B  D DC  ZSDSU   AS RB      YVDZC   E       BPPC         VJJPR         QWP       P  ASJ JV EE  BE CJ                        W BS E PYWEYPVC  B      VWVPC  SP D       J ZS SD       Q      WDCSU             B  V         D CACCBWBB BV S           EWZR         PDEVQZ  Y  WRLEPRJ RD UV     S    B VFBEV   P D  Z   V  CC  SRW ASRYVE      L    WD       JFP EJYYYWB W   SPSR              D   D  BJ  A  ZJ         BJW  E   S  V EA     W   Z WCBPS PR  RY      ED  B           C WVDDVL     R      JBCJYSB V    J      YFEZ                    JEA BW     S      A CR       DS YSVPW B VD  CW B       DV BS SBJCYVRFZ BD AUYA     YB        WCYV    C S      JPRSDBES              E VBBW  W   ER  V  DWJS S  SBZ      B       CEPE   PCB C D V D          S           Y YDS CQJE    J  F  Z         DESCY V   EDLB EVVC      WJYB W   PV      JSL           CYV ECEAS EB     D  P JEQ     DVVASVYQYJP QSSLJ    S  YSPPCWR  J         SL  B SQD A L V WB     EQVP ZA  ES D V   RSD V                 D Z      S         Q W    WV C     WPPCC        P D WD BC         CWP         C S        EJ      BJ      CJL             VD              Y   D  B  J              J    LEB  AER   QBC          ..
   7 ..      JDR PVV        W $  R CWP B      JPY               W RCV C  C    PE  WCVL    PB E       QJWDA   J        SJA         EEYSY           W          BD  EB  L  DY JW                        Y JB P C JYSWAS  Z      DSJ    WL           P  WS              YSSBB             E  D         W W BEECEP  S             QJ             S J      PWVWAWV DW  W          L BJSJE   D E  J      EW       EABCB           D        CBB FBDSSBV     V RC              B      SS  D  D          $UY           PC     S     LWFYW  D          YB  V           R YYJEWJ            Y  APWF J    Z       LBB                    LPF RB     W        WJ       EW S W     J   VB A        P RC VFESL C   C  WWVW     CS        BW$B    E V        PYPSB               R  W R  R   WP     BBV     YP       P       EJCC   JJ    Y E Q          V           C DVW E YS       J  C         F RYP     V  C  D J      JRS  Y   YB       B            QBJ DEJC  YC          E       CLSJYCPASZV PJRRA       EDWZD               R  J D     W J SU     LL D E   LE S Y   JC                             F           B    JB D      UQEV        V   SB I           CS         B C        BE      F       RP              YZ                     Y                 W    BVC  EJW   CP           ..
   8 ..      ZSV D W        V Q  S BAS Y      SZB                 CD  E  V    SP  ED P       J       EWJLE   R         R           YCV                            J     W  YQ                          PY     CJJ EW         J S     W               J               B YE             V  C         L   VJS     Y                            Y Y      R SBQP      Y          Y FPAVS     R         QV       VVZPY                     SC   FB CC                       Y      V   L  Q          SZ            WD     R     JBEJE              J  Q           V SBSWDB            V  EVEL W            WCS                     LP WE               V       RJ Q S     S   YR J          ZE WLP   F   D  DCDY     EL        QSCY    S          S JEV               W  E C  E    W     SR       Q       R       B  P   YL    $ B            C           V BR  K B           W         V J       L  E  W P      YCJ  $   BW       E              W  L Y              V       PBUWV SJBCS WD E        WP CL                    W       R L       P V P   W         R                             J                US A      VD W        D    E E            A         V          CS              DS               I                                            PJ   B A   JD           ..
   9 ..      $VB R R        Y    W ZYV        UEC                  P  S       VQ  JJ         C       FUV J   Y         S            Z                             Q        BL                          S       SD J            C     Y               A               C                   Q         R    YY                                  D D        ZJ$       S            WYVLZ                           W                      A    J  LL                       R          C  W          DB            S      V     QVV P              S                BJC  S            S  WC W               Y                       R                  W       V  B P          S P          EB  CB   W   W  CPE      RP        U$PC    Y          L CL                F  I E  Y          RD       Z               V  W    P    C              J             JC  Q E                       W       S  L  P        BPC  E   U        C              P  D                        VJW Z  LPPR CR F         W S                     Z         V       A R                                             P                BC V         A        H    A U            E         W          P                A               Y                                             S   Y Y   S            ..
  10 ..      CWJ                 B   Y         D                   Y              BV         V       $Y  S                          Q                                      EP                          Y        C                                    V               F                   P              V                                   Q R        BVB                     A                                                          S  SJ                       V          S  Y           $                   C     EY  B                               PQ   Y                B                 W                       Y                             E L            V          FJ  DL           YH      WV          ZW                 LR                Y    B             VF                               S                   W             VW  V F                       Y          V           PEL  S            J              R  Z                        YQ  A  RRVB $  S                                 J         J       E S                                             D                II S                  Y    L Y            O                                     V               P                                             W                      ..
  11 ..      LES                     Z         B                                  DR         W       A   Y                                                                 RS                                   P                                    R               P                   S              F                                     S          C                     E                                                             UU                       S                         E                   J     S   R                                $                                      L                                                     J U            W          JR  RV           $                    $                 R                      L                                                                                WE  Y P                                               LZ               P                                              L  B LC R                                    R                 U                                                                S  W                  Z      P                                                                  W                                                                    ..
  12 ..      V Y                               $                                  PY                 B   Q                                                                 SR                                   R                                                                        Z              S                                                S                                                                                   ZW                       J                                             L     Y                                                                                                                                 V                         LU               Z                    R                 F                                                                                                             W                                                                V                                                 C QY                                                                                                                         C  I                         J                                                                                                                                       ..
  13 ..      Y $                               Y                                  S                  L                                                                     UV                                                                                                                                                                            W                                                                                                            P                                                                                                                                                                                                               PZ                                    Z                 W                                                                                                                                                                                                                                  RZ                                                                                                                         P                            L                                                                                                                                       ..
  14 ..        E                               R                                                     S                                                                                                                                                                                                                                                                                                                                                                                                                                                                                                                                                                                 V                                                                                                                                                                                                                                                                                         Y                                                                                                                          R                            $                                                                                                                                       ..
  15 ..        F                                                                                                                                                                                                                                                                                                                                                                                                                                                                                                                                                                                                                                                                                                                                                                                                                                                                                                                                                                                                                                                                                            X                            Q                                                                                                                                       ..
  16 ..        Z                                                                                                                                                                                                                                                                                                                                                                                                                                                                                                                                                                                                                                                                                                                                                                                                                                                                                                                                                                                                                                                                                                                         R                                                                                                                                       ..
  17 ..                                                                                                                                                                                                                                                                                                                                                                                                                                                                                                                                                                                                                                                                                                                                                                                                                                                                                                                                                                                                                                                                                                                                  Z                                                                                                                                       ..


>sp|Q03468|ERCC6_HUMAN DNA excision repair protein ERCC-6 OS=Homo sapiens OX=9606 GN=ERCC6 PE=1 SV=1
MPNEGIPHSSQTQEQDCLQSQPVSNNEEMAIKQESGGDGEVEEYLSFRSVGDGLSTSAVG
CASAAPRRGPALLHIDRHQIQAVEPSAQALELQGLGVDVYDQDVLEQGVLQQVDNAIHEA
SRASQLVDVEKEYRSVLDDLTSCTTSLRQINKIIEQLSPQAATSRDINRKLDSVKRQKYN
KEQQLKKITAKQKHLQAILGGAEVKIELDHASLEEDAEPGPSSLGSMLMPVQETAWEELI
RTGQMTPFGTQIPQKQEKKPRKIMLNEASGFEKYLADQAKLSFERKKQGCNKRAARKAPA
PVTPPAPVQNKNKPNKKARVLSKKEERLKKHIKKLQKRALQFQGKVGLPKARRPWESDMR
PEAEGDSEGEESEYFPTEEEEEEEDDEVEGAEADLSGDGTDYELKPLPKGGKRQKKVPVQ
EIDDDFFPSSGEEAEAASVGEGGGGGRKVGRYRDDGDEDYYKQRLRRWNKLRLQDKEKRL
KLEDDSEESDAEFDEGFKVPGFLFKKLFKYQQTGVRWLWELHCQQAGGILGDEMGLGKTI
QIIAFLAGLSYSKIRTRGSNYRFEGLGPTVIVCPTTVMHQWVKEFHTWWPPFRVAILHET
GSYTHKKEKLIRDVAHCHGILITSYSYIRLMQDDISRYDWHYVILDEGHKIRNPNAAVTL
ACKQFRTPHRIILSGSPMQNNLRELWSLFDFIFPGKLGTLPVFMEQFSVPITMGGYSNAS
PVQVKTAYKCACVLRDTINPYLLRRMKSDVKMSLSLPDKNEQVLFCRLTDEQHKVYQNFV
DSKEVYRILNGEMQIFSGLIALRKICNHPDLFSGGPKNLKGLPDDELEEDQFGYWKRSGK
MIVVESLLKIWHKQGQRVLLFSQSRQMLDILEVFLRAQKYTYLKMDGTTTIASRQPLITR
YNEDTSIFVFLLTTRVGGLGVNLTGANRVVIYDPDWNPSTDTQARERAWRIGQKKQVTVY
RLLTAGTIEEKIYHRQIFKQFLTNRVLKDPKQRRFFKSNDLYELFTLTSPDASQSTETSA
IFAGTGSDVQTPKCHLKRRIQPAFGADHDVPKRKKFPASNISVNDATSSEEKSEAKGAEV
NAVTSNRSDPLKDDPHMSSNVTSNDRLGEETNAVSGPEELSVISGNGECSNSSGTGKTSM
PSGDESIDEKLGLSYKRERPSQAQTEAFWENKQMENNFYKHKSKTKHHSVAEEETLEKHL
RPKQKPKNSKHCRDAKFEGTRIPHLVKKRRYQKQDSENKSEAKEQSNDDYVLEKLFKKSV
GVHSVMKHDAIMDGASPDYVLVEAEANRVAQDALKALRLSRQRCLGAVSGVPTWTGHRGI
SGAPAGKKSRFGKKRNSNFSVQHPSSTSPTEKCQDGIMKKEGKDNVPEHFSGRAEDADSS
SGPLASSSLLAKMRARNHLILPERLESESGHLQEASALLPTTEHDDLLVEMRNFIAFQAH
TDGQASTREILQEFESKLSASQSCVFRELLRNLCTFHRTSGGEGIWKLKPEYC

PFVM
     00000000000000000000000000000000000000000000000000000000000000000000000000000000000000000000000000000000000000000000000000000000000000000000000000000000000000000000000000000000000000000000000000000000000000000000000000000000000000000000000000000000000000000000000000000000000000000000000000000000000000000000000000000000000000000000000000000000000000000000000000000000000000000000000000000000000000000000000000000000000000000000000000000000000000000000000000000000000000000000000000000000000000000000000000000000000000000000000000000000000000000000000000000000000000000000000000000000000000000000000000000000000000000000000000000000000000000000000000000000000000000000000000000000000000000000000000000000000000000000000000000000000000000000000000000000000000000000000000000000000000000000000000000000000000000000000000000000000000000000000000000000000000000000000000000000000000000000000000000000000000000000000000000000000000000000000000000000000000000000000000000000000000000000000000000000000000011111111111111111111111111111111111111111111111111111111111111111111111111111111111111111111111111111111111111111111111111111111111111111111111111111111111111111111111111111111111111111111111111111111111111111111111111111111111111111111111111111111111111111111111111111111111111111111111111111111111111111111111111111111111111111111111111111111111111111111111111111111111111111111111111111111111111111111111111111111111111111111111111111111111111111111111111111111111111111111111111111111111111
     00000000000000000000000000000000000000000000000000000000000000000000000000000000000000000000000000011111111111111111111111111111111111111111111111111111111111111111111111111111111111111111111111111112222222222222222222222222222222222222222222222222222222222222222222222222222222222222222222222222222333333333333333333333333333333333333333333333333333333333333333333333333333333333333333333333333333344444444444444444444444444444444444444444444444444444444444444444444444444444444444444444444444444445555555555555555555555555555555555555555555555555555555555555555555555555555555555555555555555555555666666666666666666666666666666666666666666666666666666666666666666666666666666666666666666666666666677777777777777777777777777777777777777777777777777777777777777777777777777777777777777777777777777778888888888888888888888888888888888888888888888888888888888888888888888888888888888888888888888888888999999999999999999999999999999999999999999999999999999999999999999999999999999999999999999999999999900000000000000000000000000000000000000000000000000000000000000000000000000000000000000000000000000001111111111111111111111111111111111111111111111111111111111111111111111111111111111111111111111111111222222222222222222222222222222222222222222222222222222222222222222222222222222222222222222222222222233333333333333333333333333333333333333333333333333333333333333333333333333333333333333333333333333334444444444444444444444444444444444444444444444444444444444444444444444444444444444444444444444
     00000000011111111112222222222333333333344444444445555555555666666666677777777778888888888999999999900000000001111111111222222222233333333334444444444555555555566666666667777777777888888888899999999990000000000111111111122222222223333333333444444444455555555556666666666777777777788888888889999999999000000000011111111112222222222333333333344444444445555555555666666666677777777778888888888999999999900000000001111111111222222222233333333334444444444555555555566666666667777777777888888888899999999990000000000111111111122222222223333333333444444444455555555556666666666777777777788888888889999999999000000000011111111112222222222333333333344444444445555555555666666666677777777778888888888999999999900000000001111111111222222222233333333334444444444555555555566666666667777777777888888888899999999990000000000111111111122222222223333333333444444444455555555556666666666777777777788888888889999999999000000000011111111112222222222333333333344444444445555555555666666666677777777778888888888999999999900000000001111111111222222222233333333334444444444555555555566666666667777777777888888888899999999990000000000111111111122222222223333333333444444444455555555556666666666777777777788888888889999999999000000000011111111112222222222333333333344444444445555555555666666666677777777778888888888999999999900000000001111111111222222222233333333334444444444555555555566666666667777777777888888888899999999990000000000111111111122222222223333333333444444444455555555556666666666777777777788888888889999
     12345678901234567890123456789012345678901234567890123456789012345678901234567890123456789012345678901234567890123456789012345678901234567890123456789012345678901234567890123456789012345678901234567890123456789012345678901234567890123456789012345678901234567890123456789012345678901234567890123456789012345678901234567890123456789012345678901234567890123456789012345678901234567890123456789012345678901234567890123456789012345678901234567890123456789012345678901234567890123456789012345678901234567890123456789012345678901234567890123456789012345678901234567890123456789012345678901234567890123456789012345678901234567890123456789012345678901234567890123456789012345678901234567890123456789012345678901234567890123456789012345678901234567890123456789012345678901234567890123456789012345678901234567890123456789012345678901234567890123456789012345678901234567890123456789012345678901234567890123456789012345678901234567890123456789012345678901234567890123456789012345678901234567890123456789012345678901234567890123456789012345678901234567890123456789012345678901234567890123456789012345678901234567890123456789012345678901234567890123456789012345678901234567890123456789012345678901234567890123456789012345678901234567890123456789012345678901234567890123456789012345678901234567890123456789012345678901234567890123456789012345678901234567890123456789012345678901234567890123456789012345678901234567890123456789012345678901234567890123456789012345678901234567890123456789012345678901234567890123
     MPNEGIPHSSQTQEQDCLQSQPVSNNEEMAIKQESGGDGEVEEYLSFRSVGDGLSTSAVGCASAAPRRGPALLHIDRHQIQAVEPSAQALELQGLGVDVYDQDVLEQGVLQQVDNAIHEASRASQLVDVEKEYRSVLDDLTSCTTSLRQINKIIEQLSPQAATSRDINRKLDSVKRQKYNKEQQLKKITAKQKHLQAILGGAEVKIELDHASLEEDAEPGPSSLGSMLMPVQETAWEELIRTGQMTPFGTQIPQKQEKKPRKIMLNEASGFEKYLADQAKLSFERKKQGCNKRAARKAPAPVTPPAPVQNKNKPNKKARVLSKKEERLKKHIKKLQKRALQFQGKVGLPKARRPWESDMRPEAEGDSEGEESEYFPTEEEEEEEDDEVEGAEADLSGDGTDYELKPLPKGGKRQKKVPVQEIDDDFFPSSGEEAEAASVGEGGGGGRKVGRYRDDGDEDYYKQRLRRWNKLRLQDKEKRLKLEDDSEESDAEFDEGFKVPGFLFKKLFKYQQTGVRWLWELHCQQAGGILGDEMGLGKTIQIIAFLAGLSYSKIRTRGSNYRFEGLGPTVIVCPTTVMHQWVKEFHTWWPPFRVAILHETGSYTHKKEKLIRDVAHCHGILITSYSYIRLMQDDISRYDWHYVILDEGHKIRNPNAAVTLACKQFRTPHRIILSGSPMQNNLRELWSLFDFIFPGKLGTLPVFMEQFSVPITMGGYSNASPVQVKTAYKCACVLRDTINPYLLRRMKSDVKMSLSLPDKNEQVLFCRLTDEQHKVYQNFVDSKEVYRILNGEMQIFSGLIALRKICNHPDLFSGGPKNLKGLPDDELEEDQFGYWKRSGKMIVVESLLKIWHKQGQRVLLFSQSRQMLDILEVFLRAQKYTYLKMDGTTTIASRQPLITRYNEDTSIFVFLLTTRVGGLGVNLTGANRVVIYDPDWNPSTDTQARERAWRIGQKKQVTVYRLLTAGTIEEKIYHRQIFKQFLTNRVLKDPKQRRFFKSNDLYELFTLTSPDASQSTETSAIFAGTGSDVQTPKCHLKRRIQPAFGADHDVPKRKKFPASNISVNDATSSEEKSEAKGAEVNAVTSNRSDPLKDDPHMSSNVTSNDRLGEETNAVSGPEELSVISGNGECSNSSGTGKTSMPSGDESIDEKLGLSYKRERPSQAQTEAFWENKQMENNFYKHKSKTKHHSVAEEETLEKHLRPKQKPKNSKHCRDAKFEGTRIPHLVKKRRYQKQDSENKSEAKEQSNDDYVLEKLFKKSVGVHSVMKHDAIMDGASPDYVLVEAEANRVAQDALKALRLSRQRCLGAVSGVPTWTGHRGISGAPAGKKSRFGKKRNSNFSVQHPSSTSPTEKCQDGIMKKEGKDNVPEHFSGRAEDADSSSGPLASSSLLAKMRARNHLILPERLESESGHLQEASALLPTTEHDDLLVEMRNFIAFQAHTDGQASTREILQEFESKLSASQSCVFRELLRNLCTFHRTSGGEGIWKLKPEYC
Predicted Results:
   1 ..XASWCSPPRAYSWWADABWCBVZJZAAAAAVDVJPSYRAAAADWEAAJSPAAAPYZAABAAJCCWAPDABASJJALWWAJBBVAAAAAABAAASBEEAPASAAAJYAAAAVWAPADAAAAAAAAEAVAAAAAAAAAAAAJAVVAAAAEAAAAAAJVYAYSWYPSAJAAWAAJAAARASYAAAAAAAAACAAAAAAADBSPBBEEWWDAPWYAAAPCCWJAAAAAJJISSAAAAAAAAABFJAJECADAVACWYADSBJWPBAAAADVSAAAAAAAAAAEEAABAACJJBEAASAACSCWAACCCCVADAAVSVVPAADAAYAAAAAAADADAAAAAAAAAAYREWWAAJVPVYAAVASCAAJSBYAWYASAAPWPZAAAAAAAWJDCPASAABAZQVBSASEABVCZJACDWABBAYESABAAAPVWPJCAAAABAYCWVJCPJJAWSSAAD$YVZAADAAAAADJSAEAAJAVAAAAAWWJJVYDAYAAAWVJPEJYADBEAAAAADADJWAEDDDDDDDAAQYBJWZWSQSCWPCDSAAAAAAYJEAJADABQAPCPCBAEBZABEARWDYCERRRRADWAEEEEEEJCWADAVDJSSAAAAAJDAAAAVVABBPWSEEYEEAEEEAAQCDAYWBSWWAEEYASJCWAJVAAADAAEAAAAWPCDJBEJCASYZZDSAVSDBAAAAJDWBAAJWRAABEDDAJAWBCAUPAACACJVAEDSAEDDDDAAEWADJJZAAEAAJVPABAADASWCJEAYADBEADCAZVQABBEAAAWBVAAABABWQZSCBEAAYAAAAAAADQWYAAAWPWSAAWAJPCYAAAWZAJYJJCCEJVAEEBBAAAAABBBDDAPCEEDEPACADAAAADAAABAADEEEEWBBACAEADAAJWBAVDAAWWSVWEAAWBVEJEEAAREEBDAQVAPEBEWJVCJJVSPEEAAAAAASBYAJEAJEEABAAEAYAAABAAASAAPAAEDAJWAJJAAJVAAAADCPEVJVAAAJAAWJVJVASJAJPSYEAPPZAJADSSVYJBBBAWSBBBAYYJPSCCJCADWVYAVJEBPJDBSAAAVJJDAJADDAAPCJARAWVACDJVAAAYEJAPYAJAAYJSVEESWCAAAAAEAPASABSYQYJPSVSVBCCYCVAAAYAPAAWDSBJPCAAWADCAADADSAAAAWAVVVBYVBEEVEYADAAAAWAAAAYDQSPVJBWWAWWYEBCYBBJAAAAAALLAEJEBVYCAAJAAAQYVVAJEAAAAACDDEPWWARAWEPAAADWVWYAJEEDAEBASAAWAAAAAAAAAAVDAPJJAEBPWERRRRAASYWRWSYAJABDPAABAAWBPEEVVYVAVPCADZAAYBPAAWJAZVJWCAJY$DJAAAJAJCVWCACEAAAAADAJAAAVJBWCAAEAEZPVACADAAAABSYVAWJAJAAAEBBAAAARBWACVSCBJARAAAAADBABYZWPPPPWAAAAAPPAJABAEAAAYAAJJBWVP..
   2 .. JPPSAYAAJW  AB  S JCSAA D D DYQUWWCPBWWDDE BPEYVAJZESEDVDAEJAPACWWCWADA   P  VAACYWDDDDDAVDQYPVAD YADDWYSDSBWAAZADADDDSDDDEWBBDPCB BEDDDYPYEEARDDDAVPDDDDPBZDADAJAEVCDQBWDEDDC  C DSDWYDJCYJ   VCPDAUAWSJBBBLAVAAABYJJ JCYWBZYR     BD  YD DDDWYBPJ QJCEYSCADPWW A ABBDYCWASCDDDDDJJYPVVDCDDJ  S YCADJPJWSJCYVPVYWA YB AJ DCBDBZDPDDDBD BASJJDDDD  VAVVVPWDAW C   C CVYJWC BQPZBJYBJ CYDBBBDVPYWJVABAJSVVVPBWJBERWCCVWASYJADEPCSAAWCZ SJWBBPABJDDADJWSAYAYVPEA$AV$VPSC J A  VLWE W AEDQJ DVVJBYVYAWJABJYDBBAAWJWWCJJADDV D DEPV A        JE WRYACAAAPSCAAJDDDDDDAWWEACJBWYPBJ WWEAPPVAB  LABVB    SA E      WJJBAE ADARS VVJPYWDWS  D  A AUAALAJ B D  BRWE W    BBAZADRPYWYDJDADE   D E SA EBEUBWZ  AAEAA EDDDDD EEYDPCESCE AA ABPS  YJYELDQCAW ABDA      BESA SVDEBD AA BV LAEASYDZJPDAEBWAECYAA AAB SVJSPSDDSSAAUSWEE EWSDDDVD  YAPVJVDESA VJVVARSZYPJJY PJ    WWY S JEEDYS      DWSDBBAWVSEADREDW BDA E AS A EAY$VVBAJD PSVPAEWAYAJA BDBEAUPBWYSJSWSBVAAZJBEJC  WY  JVA DSDD DJE JW YAFBEADDDWZDDEAQSWA YAVDVADPAQWBWQSYDBV JSBAJ ADDDBDVBYYA JP EABAADDUAVJWJJ  Y P   EAWWAZBBZWAAWS R YSWWYBACYAYVWYPVDAAAWZABAAWYVEYWSEBADEA     DABBSVVYQWZSCBDAWPJYDDBDWYYCYJS AP$BWYAA W  AACJEEVDQSRCB PDWWCJADAJ  AB V  DDAE   V BA   BAWAVDESADW C JDP YPA     JLAJWRJPDEDDDB  D E W ASV ABDEPP AEPBWDDDJBAAAW VESD  JDDWVAACVVBDAADAADBDVCYDDDDDEDVVA P VAYRVACR      AWAEPCVEAPAJJPD V VRB    CCZPJSDSY   AA  AASJASPAVAAWZWCSJABBDACZPJADDEDBADAD D EAAVDDPWAEAYWSDASDDPEBB  AESABDEB  YDDDEA B APAAVEADJQYBAPWSEYB     DDDDDA D SES W$WQ S  ECA ..
   3 .. SYB  AJSWA  PE  J EJD Y       VBAYRJSJDCLY AWBWASPCWRAE  EDSVWVABYSSDEB   W  BDSWCBVVPWSEESWEERYV  JYJDAPRDD DDP  JWYCYYVEDBEASD$S JDEVJDWD A JY   DJSECBCWVWCAPBCRBAYSADSAECS  A PD  DRB SA    WDJESCEEESJAB   EVDSCW SA YJYEJ     C       BEP D P P  CVV C VYC B DWE ZAJBBR BS P ZSRCYVDCPA  E  DEQY VYJVPWAJDA    C BA JDAVJAJDPJQSE JBEDQ BSP  QWAQSVCBDA Y   S A DPDA VPAVVPE B WVVDDDCWVVAPWJYEDBAWWSP ZVCAJEBDQ YVADQRWDBBJYVP  LYASVYDDWWWCAPAJAVVYBRVA JV QD  E C  DWCW A BBYPP QQDDEDAPBYCYDZDSJV  ABBAJPABVSD   EJ J B        CS PSB$PZYPJPVDL B CSJVDSCJC AVSCSJP LEVJJASSL   VEAR     W        BWAE B EPYWB B  DAEBDD       EABEW B      JA Z      RWDYVASA   JP BEB     L  P JABSWC    ZBDJ V YPVA JYJJBPJBB     BEAR  J  SPVPP Y LEED      RADS BYJDA  SB RE JCVBPACURWREDA E WD   WDW JDAEWPS ADDDWAAS  JPAJEEQQ    AWVZZP P SPJUSSVVPDDPC       AP  A AWWEDE        QADEJA VVVYSDWA EEE P DJ W   PVJJEPBJ A  SPV JVJAC EBEAWCLSBCYPBPREWCCYDJAWE      ABD CD Y   A     VBRBEBEAEWWBYEDD      VA  B DBDDVC  PD AWVDA YL VAEDAPCP WJ VWCCVABAWDPYPA        WDB  YDCWYBPAB S  C  DSDBWBZ AVYSRCSDACZWLJVBAW PU A   Q     AJEEEAYVDCAPA BBJJZSJESEBPA$ B  JJQWAAYJ J  ZWYSVWAZJYESE J  Y DVJVP   D Y     V     W    DZCYCSDWVCJ P A E WCW     VBEEVSP WBJEBW  Y   S W   DR SCC JVAPDCCVDSQ RA D        SQVPSSYVRBBEYWJAZWYDBVJWECBDCY   BPPWEJB       RZUPCWZCBJSYYWY B ZYS     APWS JC     V  BPYBQAFBDDBQJAYWYVJSBWPSBPCPVJED S   Q PEBAV WJJY ADR YRVJBACS  LAWBEWB   BE JAW     Y PVEVB DS$WLPAEA     VCW PD J  WY  ZYA J  VAY ..
   4 .. PW   D EBV  QV  V SSC         YSCSDVJVBVSA W DZ$VCBBJBR  W EPJSPCAYBW E      WSESBC SSPBCWJYBJSV   DL CQVZVW  VY   JVSDEW CASEJBD  DCJBBB   B DP    ERWBEECCSJEJPW  EB$JV SC W  B Y    JD ZY     E JQPYALRADE   SZJJE  W  VYQDE     L       EAA     Y  WW    ARP C SDW DPAVPP YB J  DAYEWS SP     SV   CJBS ZSWA     P YP BBE PVYVJVJ C  VWP   BJ  $DSBJ SJZP     A B JCE$ ZWCCYAZ E  $CJJJJYJ$CABYPPPDWPA$Y EERBLSSBC J W  VCYVWYBAY  BCEJCJSBCJVEBQZSCPCSWSB  DY WJ       BB   R JDBWS WD WDABQYAVVWABVSP  CRVCSQE  E    J  S D        DV YDJJYCPCBBRE    E B$SBZD  P C D    JRDABYEW    ZP               LEEC    VVAC    AWDCSJ       B DBI         E V      SJWJ PA     Z SBD     A     DWYSF    BR D A V B   ACYCASZR     SW C  W  DJJJV D BRR       CBVR  WPJD  D  S  EEBEBZSY BSS     BA   RED  YLJ Y     BCYV   BBJEBBD     BB PJV S DSAPYCWAEWVAV       BA     LDWVB        VL ADJ   WP   B DBL          VYSPSWC     AJ  EP B  D AVDAVJVWDWLBWAPDDDAR BB       C     V   W     DL  DEWVBAPEBDB        B     AAEWB  S  LFCJD J   DPBCCW  S  YRDV RCYYLECVB        PLJ   VE  JJ V B     JEPJVJA PCWYWEBEPDQPRWSDBB  V J   J      YAJJD DPBDBD YWYBYVVBJLABVP S  VVAPQZJ     JZZCWDPSACBJS W    W SCA                 E     DDVWYPD  C Y B J BS      S DR FS YDB EY  B     J   ED   S   BABWS VA  WE B        BYPYE DAA ERDD JSBD W  ESZEEWD   WVSVPSP       CJYSBVQS  PVACP   $EV     YSC  W         PQVRJB EECJVPBEDBDPYZEA$DABVYBR  W     WSSYW AD D CPB SVELRSVC   VJESBW   DB           BWDZD  WWEVACWY     WEV BW        YJB     SJ ..
   5 ..      J C B  DD  E FVA         APYBJCDYC  B D PSBYSDSWUY     DSDSECPCR J      JBCYW   BQEDDWSPWAW    W BCBS    Y    VCPJW  WVJP YP   BPCSJ   R BZ    DCBSSWSB SCDZY  QVBC  PF J  W Z     E  B     S UCWAR  WS    CPVFU  B  DZBZP     P       SVJ     D   E    WC  J E   JQQYRW  P E  QHAW    Y     BC     VY  PSS     J  Z CSS WDZ QSP B  WCB       WZCPA ECBB       D  SAP A JASB  W  JSWWWVBSADQASSJYJDCYAZ Y L BAJSP P B  S W SDJJB  E PCAPJVQBDQVIYCZWSPVL   WC                 SVSVZ  P PSQSDZBDSPBP V   ES VB S  B    R  C          SU AYAY  DVVCJS    L S CPAB    E J    VPY   RA     D                ABS    Y EJ    YJBEV        D WZ          S A       SJV W      V EW      B      AVYP     V       W   VP YYVCS     PR       BA      WF        SC E   C W  Y  V  BWCJJB   LC      VJ         PA D     J$     VV BJ W     SS  YQ Y W Y PJZSDBBDD       LQ      VB J        BW WPD   Y    E  WD          Q BSJ D     BV  B  W      BWAPYPJBWJDVDJYPBA RV       A     Q         BS  W  CPCSJDVE        J      ZSPP  J  W YL  D   PWLJBA       EB  EWJYVR V        D A   PS  WY R J     BR RRS  VPCD JD BQDDSLBSSA  B C   P      SRPCW ZJJSCJ ADUCSDZJVBJSEQ V   WESJJB     PBA YSD CDLL          BY                 J      SCBVJB      C   SW        RU AC   W  E  E     V    E       SSEBJ BW  BC A         CCSB  PL JWLP R DR    BYSSBB    ACJAJRJ        CCWJAWB   E DJ   C E      VY             DDDS    P CB JBDBYVEBYJWBJECDJ          EBY BV A JEF  YLWJY W   PRRV                  Y S E  D VBDWVC      B  EJ        JZC     BP ..
   6 ..      S B        Y L           EWSVPZPPJ  V   JEYQWY Y A     LAREP AD         S RAJ   VJJSBYPAYB       V W            QBB  JLDD       W  P   W V      E JCDPW  WS B  W ES   L V  D J     W  V     R VHJJW  YE    LWWZ   E  LWPCD             PQD     J        ZE  P       PJ    R S  B W     Q      B     YB  YBR          EVJ CPP SQB S   DC       PP$AL JSYC          WY  C VDEQ     LPCCCQCBPLWJEJVWVSJDJ    W CWRWY V S  A S  RCSD  W VYWVREVSLBDVCUVJWWCJ   QE                  SVBD  S BWBDADCPCSCV     VW  L            Y           Y JVCW  JJYABB    P V BEBL    J B    AS    V      W                DDW      PF    BCJSB        F             V W       VSD        S VR             PEPB     Y           WD  DCJD     Q        CB                WJ J                DCAV           SV         D  J      P     C  CS Y      A  PA V   S C B$JYEZJ               BJ P        RR   V        J                YBW P     W   L  Y      ESELPJCERLESJSPV S  P             J         PJ     JJQZZQW         L       B W  W    AZ  S   W  VAB           WJCQSB          U E   WW     W        Y  ED  CZJP  V JUP EPD DJ  E S   Y      V UV  CVSVY    A  RECPPLQCY $   $ VVVD     SS$ DBC KBWA                             Y      BBDEBE          CB        J  EE      V             S        WSSB PJ  CD           WZ P  SQ WSWS E C     S V W     PBWBSBV        PBJRZBW   Q      P        JJ             SPPE      YC VCZYQAQSSASSYCPVS          VWB DC B  J   FBEWL R   WPVD                  D   W  Y LDCDSE      S  W         PBW     J  ..
   7 ..        Y          R           BAH ZEAB        AEWBP C       YB B   R         C   S    VVWQ VDVD         J            W    BDWW       B  S   L S      P EJB S   L      JY   R Y          L  $       BAVCC  DV     SEC      P D S             YWE              SL          C     C R  V D            R     Z   BFY           WL DWV  BS     LW       ELWW  V  D          BQ  J Y$J      AJVVVSJWCB Y RWBWYQPZ    F EQW V D E  Y V   DWV    YWSDZPY  JRYQPPSQASB   BB                  WC V  B CCE VS  B PZ      Y  D            A             VBDC   W YAR      E JYL     A C    DW                            B P      VR    QPLV         L             J         LVS          J               WVE                 J    YLV               S                 V                  LVCW           DW         E  V      S     P  VR        J   B     B    DVJPCP                S R        YJ   Y        P                PLY       E   P         JLYAZBZSURJR RWJ D                B         CR     WDSC RJ         D       J    C               EWF           S VJYV          C P   YV              A  A   FAS   P E S  SY EU                     AB$PW    E   YSCWC DV E   A AYDS       D SCZ DVJV                             A      PSSCCP          EV           J       D             I        D J  CE   Q              Y  DZ  BV  W S       Y       E BJAEY        ASVEJ R   A               YA             $$           SP  SPAYJYEWPYSPB          C   RB P       CPDP V   B YJ                  W        AEE  J                    RP        ..
   8 ..        J          W           JCV  AEE        CW Y  V       E  R   Y         D        Y  L  VLW         A            E     JL           W     Y        V   A   B      R    S P             Q       WEYBJ         CSR      B J C              CL              BV                E B  W Z            L         JL            J  SQ   EZ      S       U BD     J          VB  S SBL      BRSSSWSYJV E DYS EDC       VVY R   P                QYWE B  VW JBBYAC F    D                        W RYJ CV  J V          P                          CWES   Y QW       Q E S     V Y    PU                            L D      BZ    SV Y         W             C         ABW          W               PJ                       BP                W                 D                  WPY             B         B  Q            S  ZL            C     C    PWSQB                 V          EB   S                         CWV       J   S          V CALWCCAAJ ZZW                  E         SW     BSVV  L                 Z    E               PZ               B F            Y    A              W  D    BV   W Q $     YV                       D Q    V   BPDJD WW P   B CCB          B J EWDP                             P      VRYW C          J                    L             W        J P  EP                     Y   LB    Y       J       U CPW          SE SB     F               CL             C            W    ZVJLBYVWJBSP              YY W       PREV     C D                            J F  P                     S        ..
   9 ..        X                      RZ   DCS        QC    B       C  U   P         Y           C  CAY         R            J                        E        W   D                 B             W       QWBVD         DPV      C V                                CA                  C                 Y         W             F   J   P                 EJ     S           S  P $JC      CWPPPBEQ S S QD  JYL        D      C                UBBP E  YZ EYSEJL C    S                          Q V W   W            Y                          DAVP   B V        W         L W                                    J      C     ZS W                       Y          C                           AE                       WY                Y                 P                  YWZ             P         S               W  F             J            C Y                            AF                             DA        D   V            QJVVAAD W PJ                   P         W      Y BE                                         L                P P                 J              C       WB   C   C                              P A    C   SVESS ZJ W   Y EBP          P   V CE                                    FJJ  R                                                      R     D                     C    P            L       Y ZLY          BV YY                     BS             V                   CVLJIDBERW                 S         VW     S P                              V                        E        ..
  10 ..                               FR   S R         R    E       B  V   Q                     P  W C                      R                                                       D             Z       SJR L         R D      F W                                RB                  F                                         P                          L     I           V    BN       DYYYYEPC   R W   L          L      V                EESV J     VQWBLB W                                 S     P            E                          LL R   E                      V                                    V      D      Z Z                       B                                      BZ                       PW                                                     Z E             Q                         Y  Y             U            V S                            D                               $        Y   R            RCIBYVS Y VQ                   S                  LY                                         Y                A                   P              J       JE   E                                  E Z    P   W RV   S     Z YSC          R     QF                                    YPP  Y                                                            V                     Z    S            D         ESC          RP A                      DD             B                   UERRL SWYY                 V          J       W                                                       J        ..
  11 ..                               LY   $ D         P    A       W                                 J                                                                              R                     RP  P         $ E        $                                LJ                  U                                         E                          Y     Y                          EEEEPZE   D Z              P                       RL W P      RQLEE                                   R     R            R                          ZE V                          Z                                           F        $                       F                                      D                         E                                                                                                  R                                                         W                               R            D             SOPZPF   W                                                                                  S                C                                  D       DD                                      Y      L     W            LUW                                                       W                                                                Y                                                 R D           J Z                      ER             E                   LF  U R                    C                                                                          Z        ..
  12 ..                               U    B           L    L       S                                                                                                                                      $D  F         Q                                            P                  W                                         Y                                                           LRRRY$D   $ C              Y                       VF   S      W$R$O                                   Z     U            W                           P                            Q                                           L                                L                                      L                                                                                                                            S                                                                                                                     SEVY    Y                                                                                                   S                                  L       UQ                                             B                  IE                                                                                                                         Z                                                 V L           R V                      WZ             W                   PB    W                    H                                                                          V        ..
  13 ..                               W    Q                                                                                                                                                                I  V                                                      F                                                            L                                                            FFFFD$     V                                      AH   Z      EDZUU                                                                                  $                                                                                                                                                Z                                                                                                                                                                                                                                                   QQJ                                                                                                        W                                  P        F                                             I                  UL                                                                                                                                                                           D R             F                                     Y                   QD    F                    J                                                                                   ..
  14 ..                                    W                                                                                                                                                                Y  Y                                                                                                                   R                                                            LLLR R                                            ZI          IEFIZ                                                                                  Z                                                                                                                                                                                                                                                                                                                                                                                                    RU                                                                                                                                                                                                   R                  FZ                                                                                                                                                                           $               L                                                         FZ    H                    U                                                                                   ..
  15 ..                                    L                                                                                                                                                                $                                                                                                                      $                                                            $$$L U                                             Q            UZ                                                                                                                                                                                                                                                                                                                                                                                                                                                                                        $L                                                                                                                                                                                                                      R                                                                                                                                                                            Q                                                                         H     L                                                                                                        ..
  16 ..                                    R                                                                                                                                                                V                                                                                                                                                                                   III                                                             Q                                                                                                                                                                                                                                                                                                                                                                                                                                                                                         LI                                                                                                                                                                                                                      Z                                                                                                                                                                                                                                                      I     Q                                                                                                        ..
  17 ..                                                                                                                                                                                                                                                                                                                                                                                                                                                         O                                                                                                                                                                                                                                                                                                                                                                                                                                                                                                                                                                                                                                                                                                                 O                                                                                                                                                                                                                                                      R     U                                                                                                        ..
  18 ..                                                                                                                                                                                                                                                                                                                                                                                                                                                         H                                                                                                                                                                                                                                                                                                                                                                                                                                                                                                                                                                                                                                                                                                                 $                                                                                                                                                                                                                                                            Z                                                                                                        ..
  19 ..                                                                                                                                                                                                                                                                                                                                                                                                                                                         X                                                                                                                                                                                                                                                                                                                                                                                                                                                                                                                                                                                                                                                                                                                 D                                                                                                                                                                                                                                                                                                                                                                     ..
  20 ..                                                                                                                                                                                                                                                                                                                                                                                                                                                         $                                                                                                                                                                                                                                                                                                                                                                                                                                                                                                                                                                                                                                                                                                                 H                                                                                                                                                                                                                                                                                                                                                                     ..
  21 ..                                                                                                                                                                                                                                                                                                                                                                                                                                                         I                                                                                                                                                                                                                                                                                                                                                                                                                                                                                                                                                                                                                                                                                                                                                                                                                                                                                                                                                                                                                                                                                       ..
  22 ..                                                                                                                                                                                                                                                                                                                                                                                                                                                         D                                                                                                                                                                                                                                                                                                                                                                                                                                                                                                                                                                                                                                                                                                                                                                                                                                                                                                                                                                                                                                                                                       ..


>sp|Q16637|SMN_HUMAN Survival motor neuron protein OS=Homo sapiens OX=9606 GN=SMN1 PE=1 SV=1
MAMSSGGSGGGVPEQEDSVLFRRGTGQSDDSDIWDDTALIKAYDKAVASFKHALKNGDIC
ETSGKPKTTPKRKPAKKNKSQKKNTAASLQQWKVGDKCSAIWSEDGCIYPATIASIDFKR
ETCVVVYTGYGNREEQNLSDLLSPICEVANNIEQNAQENENESQVSTDESENSRSPGNKS
DNIKPKSAPWNSFLPPPPPMPGPRLGPGKPGLKFNGPPPPPPPPPPHLLSCWLPPFPSGP
PIIPPPPPICPDSLDDADALGSMLISWYMSGYHTGYYMGFRQNQKEGRCSHSLN

PFVM

     000000000000000000000000000000000000000000000000000000000000000000000000000000000000000000000000000111111111111111111111111111111111111111111111111111111111111111111111111111111111111111111111111111122222222222222222222222222222222222222222222222222222222222222222222222222222222222222222222222
     000000000111111111122222222223333333333444444444455555555556666666666777777777788888888889999999999000000000011111111112222222222333333333344444444445555555555666666666677777777778888888888999999999900000000001111111111222222222233333333334444444444555555555566666666667777777777888888888899999
     123456789012345678901234567890123456789012345678901234567890123456789012345678901234567890123456789012345678901234567890123456789012345678901234567890123456789012345678901234567890123456789012345678901234567890123456789012345678901234567890123456789012345678901234567890123456789012345678901234
     MAMSSGGSGGGVPEQEDSVLFRRGTGQSDDSDIWDDTALIKAYDKAVASFKHALKNGDICETSGKPKTTPKRKPAKKNKSQKKNTAASLQQWKVGDKCSAIWSEDGCIYPATIASIDFKRETCVVVYTGYGNREEQNLSDLLSPICEVANNIEQNAQENENESQVSTDESENSRSPGNKSDNIKPKSAPWNSFLPPPPPMPGPRLGPGKPGLKFNGPPPPPPPPPPHLLSCWLPPFPSGPPIIPPPPPICPDSLDDADALGSMLISWYMSGYHTGYYMGFRQNQKEGRCSHSLN
Predicted Results:
   1 ..JYQVCVAVPPCYBDYAAWAAYYSAESEZPAAAAVAAAAAAAAAAAAABADAWAP$ABAEBSVWVEAJYJYWYEVDBAASWPCAAAAAAJEWYJWSWRWSBADJYPSWSBJSWCEBVDPYJWBBWJYDJBJEWRAJVAAJAACCPLAJAJVADJAWAADAYAEAWYJBPAQAWJVAAJWAABEBYJPCVYAJPCCCCCPVWCYVCCAJYJPBDU$CCCCCCCCYZAAABPCFWVCSPPCSVJWCCCFFFCYAPSYVADABAAEBEAAAADAVDYPAADQAEESJWYSSSVS..
   2 ..DVUPPYVACZWAWLWBWBELWV JJJBAAJVPSAPDVDDDJJDDDSDA ADADYSB J EJYPR JCR W ZSEAJ  C JAPVDVYBEBCSAPBAJLESCZPSWRBCEWAVSBWAAJVWLEEECCACVBJBAWSYVYAWCSWEW VWSADAA  DBACV ADLVSSJY$J WZCDAVSJPBCWA WAWVPJWWS  JBVYCAWZYCJAAEBV  YSSSSSSCYSJD AF CYWYYCJ A SWSS   VWJAAJADAJWRBDAD   D DA ASD A D   P S    D..
   3 ..A AZAAYYVCSCD PJDABWAA  ABVVVSWCWYDWD EEBCWJWDBE   EWWJ    VYSCE SPZ    YDSP  P  DVDCPJJBSLZ$JPD  CAVQCVJE WYADAAAEEPCSBAR VBVWEWZZJEJBJDDDEPYSBA ADW  EB   VBSA JESCB VDCV  SJBWYYSEWWVB  Y J C SW  YC PSBPYJPVSJSSA  SWWWWWW  DDJ    ASYZJWY   CSWB     SVDSDWPELJWA A     ED SJ  Q E   A      A..
   4 ..Z VALZPSASA V  VEEDB S   AJEWYBQD YY  JRWDPPPRSP   DZAC    W PVA  AE    BBBS  B   JZQSZEAABRCB    V FS A   RWVEBBDRJ DWLJA RSWYPYYVAJBCABZZJFRBVE CPC   D   DYJ  B  WV WCPW  YWJB $C  REP      W PB  SW SVEV$PFCW WWY  WBBBBBB  Y B     CZCAA      BE     CEJA VEDSEJB       SE VA        Q      E..
   5 ..  ZYVBJBWBJ Y  PSL E     PWBCBJVJ C   S  SVEV       YQY    J B J  WS     W W  Y    WJWV SL  W     F J  J   V PBPE LD SZ ED BA  RCEBPS WBYVECVZDWB  Y        WPP  S  DA  JV     SV  P  SZ         YV  B  ZAJAPVWPB DEB  AVVVVVV            AQL      VF     VRPP PCP PL           W         D      R..
   6 ..  CJSCQEYV      B  J     WSJSZP$  J      B L        QVA    P   S  B      Y Q  Z    YEYS     P     R P  B   B BYCJ  B  I  V LW  SSWYS  AWCBLLY A D  $          Q  V  ZC  SA     W                 AP  Q   BYBJB SC      LPPPPPP            LVS      P      ZYVV JJV DE                     W      J..
   7 ..  DCYSWJLW      R  D     V WD     Q        V        CZZ    S   Y  E        V  A    BSBB     Q       W        ER P     J        A PRL  V SPPV  E               V  W  A   PJ     P                 VA      FP QS W        AAAAAA            PZY      A      RL D CS  SS                             ..
   8 ..  WBJWECE       C  V     Z CJ     L        W         $V    Y      L           D    QPDD     S       D        DV R                 WY    PSYB  J               Y  C  E   VY                       BJ      JR SW          JJJJJJ            VCE      J      YB   SB  CV                             ..
   9 ..   WWJDIU       F          Y      W        B         J     Z                  V    SBEC     B       B        CJ D                        JBF  V                  P      Q                        JE      PW             EEEEEE            WEJ      E       D    W                                 ..
  10 ..   EBPBUH       J          R                         C                        W     WJE     V                F                           WW   Y                  R      W                        LF      W              FFFFFF             SR      F       S    L                                 ..
  11 ..   QEECHO       Y          S                                                        LRW     E                                                                    Z                                Y      Z              YYYYYY             WV      Y       W    V                                 ..
  12 ..   SRUSRS                  P                                                        V       F                                                                                                     L                     LLLLLL             B       L       Z                                      ..
  13 ..   UDRZW                   U                                                        Z       Y                                                                                                     I                     IIIIII             L       I                                              ..
  14 ..   LFQIL                                                                                                                                                                                                                                   U                                                      ..
  15 ..   KIDLZ                                                                                                                                                                                                                                                                                          ..
  16 ..   IZ$UQ                                                                                                                                                                                                                                                                                          ..
  17 ..   $$F$P                                                                                                                                                                                                                                                                                          ..
  18 ..   RQIRF                                                                                                                                                                                                                                                                                          ..
  19 ..    OLHO                                                                                                                                                                                                                                                                                          ..
  20 ..    UXF                                                                                                                                                                                                                                                                                           ..
  21 ..      O                                                                                                                                                                                                                                                                                           ..
