# Supplementary material for: Prediction of folding patterns for intrinsic disordered protein
Source: Sci Rep. 2023 Nov 21;13:20343. doi: 10.1038/s41598-023-45969-5 (PMC10663623; doi:10.1038/s41598-023-45969-5)
Supplement: Supplementary file 2 — Supplementary Information 2. [file 41598_2023_45969_MOESM2_ESM.docx]

# SUPPLEMENTARY MATERIAL

All data generated or analyzed during this study are included in this published article and its supplementary information file. The supplementary information is stored in a file with rich text format (RTF), which can display longer sequence in a row, and then better exhibit the PFVM matrix. The name of supplementary file name is “20 Protein PFVM.rtf”.
